# Maternal education and equity in breastfeeding: trends and patterns in 81 low- and middle-income countries between 2000 and 2019

**DOI:** 10.1186/s12939-020-01357-3

**Published:** 2021-01-07

**Authors:** Paulo A. R. Neves, Aluisio J. D. Barros, Giovanna Gatica-Domínguez, Juliana S. Vaz, Phillip Baker, Chessa K. Lutter

**Affiliations:** 1 Federal University of Pelotas, International Center for Equity in Health. Rua Marechal Deodoro, 1160, 3rd floor, Pelotas, Brazil; 2 Institute for Physical Activity and Nutrition, Deakin University, 221 Burwood Highway, Melbourne, Australia; 3 RTI International, 701 13th Street, Washington DC, NW USA

**Keywords:** Breastfeeding, Equity, Maternal education, Infant formula, Breast-milk substitutes, Developing countries

## Abstract

**Background:**

In low- and middle-income countries (LMICs), low levels of formal maternal educational are positively associated with breastfeeding whereas the reverse is true among women with higher levels of formal education. As such, breastfeeding has helped to reduce health equity gaps between rich and poor children. Our paper examines trends in breastfeeding and formula consumption by maternal educational in LMICs over nearly two decades.

**Methods:**

We used 319 nationally representative surveys from 81 countries. We used WHO definitions for breastfeeding indicators and categorized maternal education into three categories: none, primary, and secondary or higher. We grouped countries according to the World Bank income groups and UNICEF regions classifications. The trend analyses were performed through multilevel linear regression to obtain average absolute annual changes in percentage points.

**Results:**

Significant increases in prevalence were observed for early initiation and exclusive breastfeeding across all education categories, but more prominently in women with no formal education for early breastfeeding and in higher level educated women for exclusive breastfeeding. Small decreases in prevalence were seen mostly for women with no formal education for continued breastfeeding at 1 and 2 years. Among formula indicators, only formula consumption between 6 and 23 months decreased significantly over the period for women with primary education. Analysis by world regions demonstrated that gains in early and exclusive breastfeeding were almost universally distributed among education categories, except in the Middle East and North Africa where they decreased throughout education categories. Continued breastfeeding at 1 and 2 years increased in South Asia, Latin America and the Caribbean, and Eastern Europe and Central Asia for primary or higher education categories. Declines occurred for the group of no formal education in South Asia and nearly all education categories in the Middle East and North Africa with a decline steeper for continued breastfeeding at 2 years. With a few exceptions, the use of formula is higher among children of women at the highest education level in all regions.

**Conclusions:**

Over the course of our study, women with no formal education have worsening breastfeeding indicators compared to women with primary and secondary or higher education.

## Introduction

To achieve optimal growth, development, and health the World Health Organization (WHO) recommends infants and young children are exclusively breastfed for the first 6 months of life, and thereafter receive nutritionally adequate and safe complementary foods, while breastfeeding continues for up to 2 years of age or beyond [1]. For the child, breastfeeding significantly reduces the risk of diarrhea and respiratory infection, malocclusion, and all-cause mortality, and probably the risk of obesity and type-2 diabetes [2]. For mothers, breastfeeding reduces the risk of breast cancer, and possibly ovarian cancer and type-2 diabetes, with further benefits for birth spacing and family planning [2]. Not breastfeeding is attributed to an estimated 595,379 child deaths (6 to 59 months) annually from diarrhea and pneumonia alone, and among mothers 98,243 deaths from breast and ovarian cancers, and type-2 diabetes [3]. Not breastfeeding also generates estimated economic losses of US$341.3 billion annually, resulting from higher health care costs, premature mortality and lost productivity [3, 4].

Over the past decades, substantial improvements in levels of formal education among girls and young women in low- and middle-income countries (LMICs) has occurred. In these countries, low levels of formal education are positively associated with breastfeeding practices whereas the reverse is true among women with higher levels of formal education [5]. Breastfeeding is a positive health behavior that is more prevalent among women with low levels of formal education compared to women with high levels of formal education in LMICs. Because higher levels of formal education are positively correlated with socio-economic status [6] a higher prevalence of breastfeeding among women with lower compared to higher levels of formal education has helped to reduce the nutrition and health equity gaps between rich and poor children within and across countries. WHO considers ensuring equitable access to breast milk for all infants is a key component of essential newborn care [7]. This is further reflected in the ‘nutrition equity’ theme of the 2020 Global Nutrition Report, which calls on governments, donors, civil society groups and others to develop equity-sensitive nutrition policies, programming actions and data systems in support of the Sustainable Development Goals [8].

Substantial investments in policies and programs to improve breastfeeding have led to progress globally [9]. This is reflected in the global exclusive breastfeeding rate (< 6 months), which increased from 33% in 1995 to 42% in 2018 [10]. However, assessing progress only at the aggregate global level, misses important nutrition inequities across and within countries [8]. For example, in several countries in Latin America where increases in breastfeeding have occurred, they have not been distributed equitably among different population subgroups. In Bolivia, Brazil, Colombia, and Peru the population subgroups whose children are most at risk for mortality and morbidity were least likely to show improvements in breastfeeding duration over a 20-year period [11]. Even where prevalence has not changed at the national level, differences in subpopulations have been observed. For example, in Mexico, although the prevalence of exclusive breastfeeding and any breastfeeding did not change between 1999 and 2006 for the population as a whole, both declined among vulnerable population groups, including indigenous women, while they increased among women with higher education and socio-economic level [12]. More recent data from the 2018 national survey from Mexico show at a national level significant increases for all breastfeeding indicators compared to 2014 [13].

One major barrier to achieving optimal breastfeeding in all country contexts, is the aggressive marketing of breast-milk substitutes (BMS) through health systems, direct-to-consumer advertising and other channels [14, 15]. Recent studies have shown an escalation in BMS sales worldwide, driven mainly by growth in highly-populated middle-income countries, especially n China and throughout South East Asia. This is occurring not only in the standard infant formula category, but also, in categories for older infants and young children, including follow-up and toddler formulas [16, 17]. The WHO has long maintained that these latter milks are unnecessary and unsuitable as substitutes for continued breastfeeding [16, 18, 19]. Despite the implications of the above for child and maternal health, and the scaleof change in global feeding practices underway, WHO and UNICEF do not include an indicator for the use of milk-based formulas in the current global monitoring system for infant and young child feeding IYCF [20]. Therefore, formula consumption is not regularly monitored as an indicator for assessing changes in worldwide feeding practices [21, 22].

Increases in levels of formal education among girls and young women are important for women for a myriad of reasons and something to be celebrated. At the same time, because higher levels of education are associated with lower levels of breastfeeding it is important to measure its effect so that policies and programs can be adjusted and breastfeeding protected, promoted, and supported. In this paper, we examine trends in breastfeeding indicators and use of formulas for infants and young children by years of formal maternal educational in LMICs over the past 20 years by World Bank income classification and regions of the world as defined by UNICEF. We also highlight a country from each world region for more in-depth analysis and discussion.

## Methods

The International Center for Equity in Health’s database (http://www.equidade.org) contains, to date, data from 411 national surveys from 117 countries, including a large body of reproductive, maternal, newborn, and child health and nutrition indicators. Drawing from this database, we analyzed data from nationally representative cross-sectional surveys periodically carried out in LMICs, namely Demographic Health Surveys (DHS) [23] and Multiple Indicator Cluster Surveys (MICS) [24]. These surveys are highly comparable in terms of sampling methods, questionnaires, measurements, and field procedures [25]. Both employ multistage sampling strategies to collect data at household-level, through face-to-face interviews with women of childbearing age (15–49 years) using standardized questionnaires administered by trained field workers. Information on IYCF practices for the youngest child less than 2 years of age in the household was collected using a qualitative 24-h food recall except for early initiation of breastfeeding which was assessed by maternal recall from the time of the child’s birth. When multiple births per women were reported, the youngest child was selected.

**Fig. 1:**
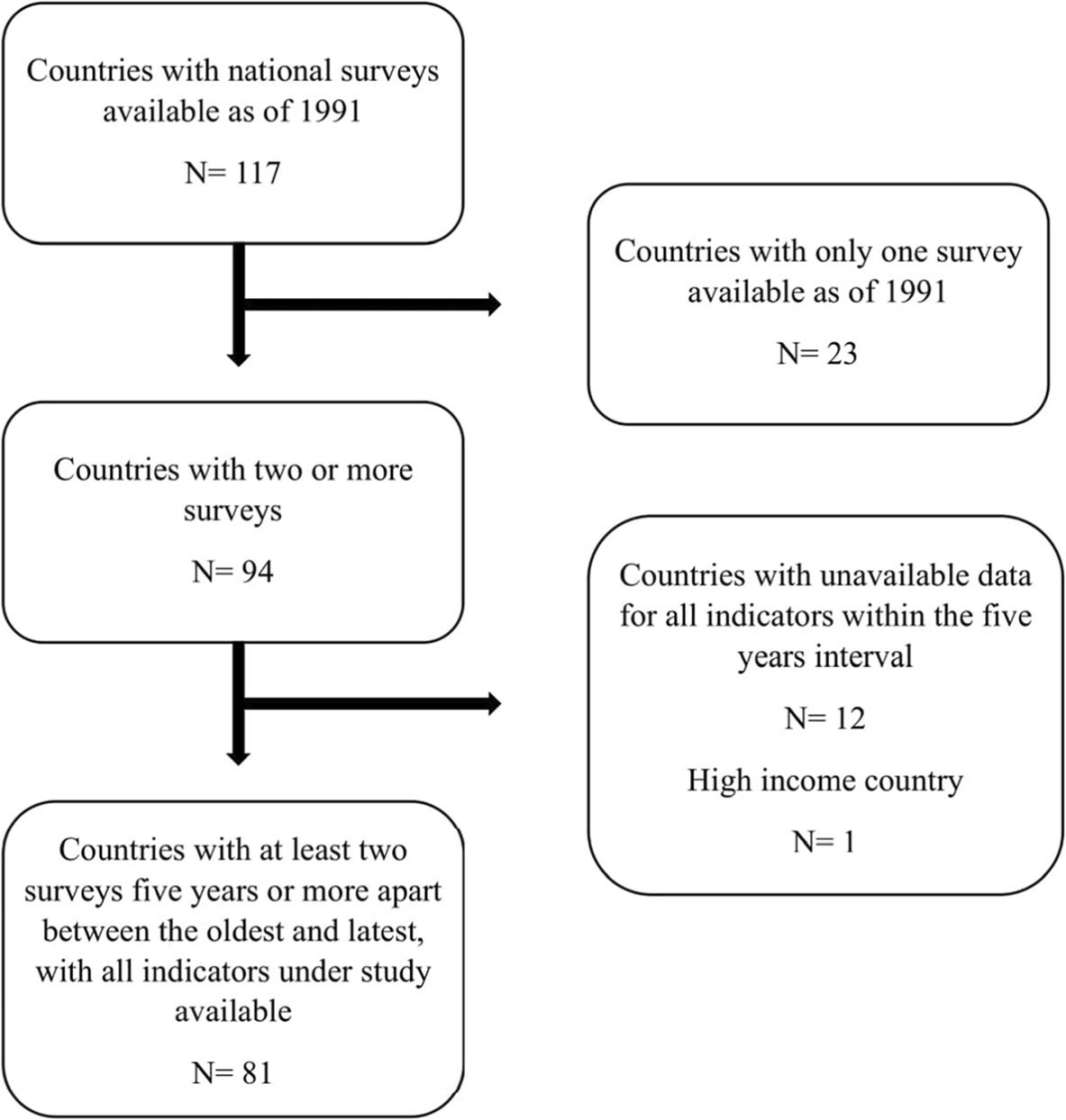
Flow-chart of selection of low- and middle-income countries in the trends and inequalities analysis by mother’s formal education

**Fig. 2:**
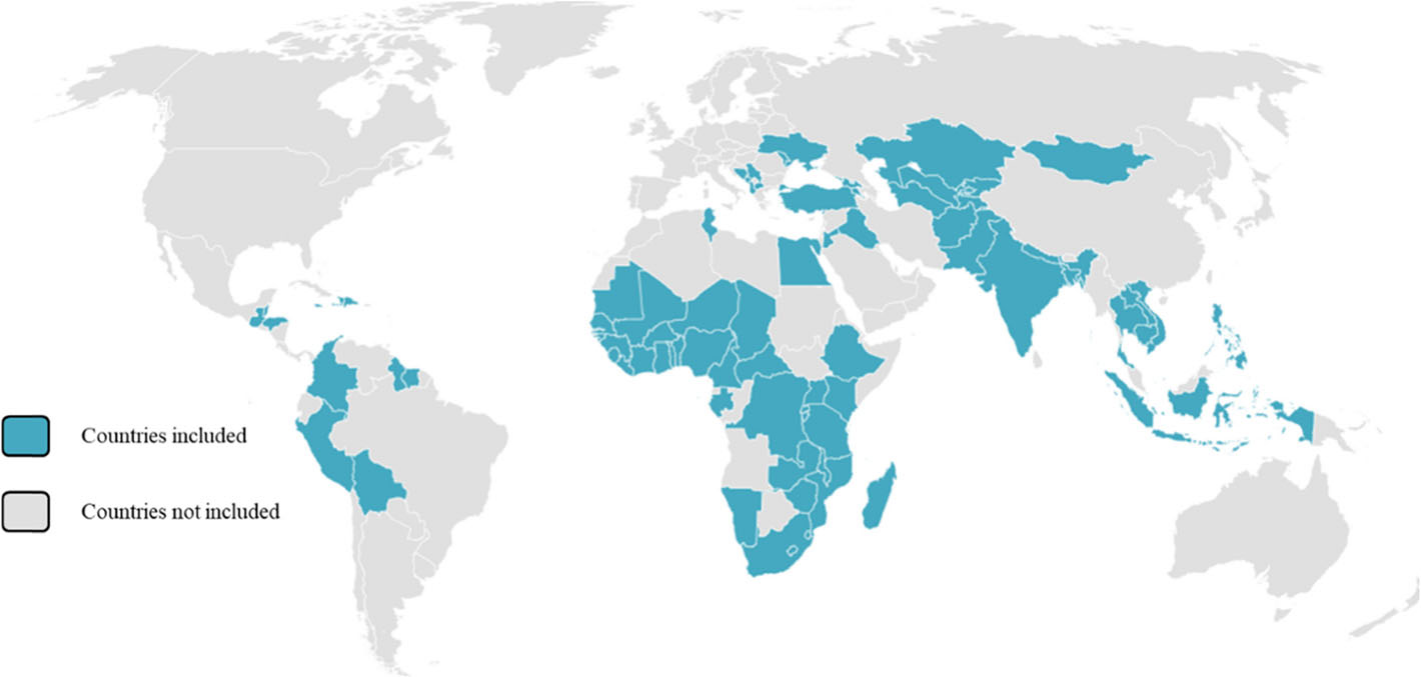
World map showing the countries included in the analysis

For the present study, DHS data from 1993 onwards and MICS data from 2005 onwards were available. Only surveys with the full set of indicators we sought to investigate, and with at least two-point estimates from surveys 5 years or more apart between the earliest and the oldest survey, were analyzed. Of the surveys available, 319 from 81 LMICs met our inclusion criteria (Fig. 1). Figure 2 shows a world map highlighting the countries included in our analysis.

Countries were grouped according to World Bank’s income classification at the year of survey implementation and by UNICEF’s regional classification [24, 26]. Supplementary Table 1 details the countries included in the analysis, their income and regional classification, and the sample size by age range. The cluster sample design of the surveys was taking into account when performing the analyses using the ‘svy’ command in Stata. Moreover; all analyses were weighted by the average population size of children in the year the surveys were carried out using the World Bank Population Estimates and Projections [27].

### Infant and young child feeding indicators

Based on WHO definitions, we calculated the following indicators: *early initiation of breastfeeding* (proportion of children in the last 24 months who were put to breast within 1 h after birth); *exclusive breastfeeding under 6 months* (proportion of infants 0–5 months of age who are fed exclusively with breast milk); *continued breastfeeding at 1 and 2 years* (proportion of children 12–15 months and 20–23 months of age, respectively, who are fed breast milk) [16, 17]. Focusing on milk formulas as the main type of commercial BMS consumed worldwide, we also calculated the following two indicators concerning the consumption of formula: *consumption of formula under 6 months* and *between 6 and 23 months* (proportion of children between 0 and 5 months and 6–23 months of age, respectively, who are fed formula). We checked the consistency and quality of our recalculated estimates by comparing them with the published figures in the DHS/MICS reports for the WHO standard indicators. Almost all differences were < 1 percentage point (p.p.), except for small discrepancies in exclusive breastfeeding when some foods or liquids were not taken into account to generate the official estimate in the report. Missing values and “don’t know” answers were considered as “not consumed”, following international standardized recommendations to deal with such cases [28, 29].

### Mother’s formal education

The definition of mother’s formal education attainment is country-specific and included in the raw datasets. For simplicity, we recategorized such variables into three education levels: none (no formal education); primary (7 years or less), and secondary or higher (8 years or more). Religion education-only categories were deemed as no education because no formal curriculum is associated with this type of teaching.

### Trends in IYCF indicators by formal education categories

The analysis was done for all countries, by income group, and by region of the world. Multilevel linear regression models were used to estimate absolute average annual changes (AAAC) in feeding indicators in p.p. using all data available; however, we show trends from 2000 to 2019 to depict changes over the last approximately 20-year period. In our model, countries were deemed the highest hierarchical level and the categories of formal education the second hierarchical level. Beta coefficients alongside the 95% confidence interval are given for each indicator for all countries, income group, and region of the world. Linearity was checked using fractional polynomials and no significant differences between the polynomials and the linear regression were observed, therefore, we adopted the latter for simplicity. Graph bars are presented to illustrate AAAC and line graphs to show trends over time by category of education.

### Inequalities in IYCF indicators by formal education categories

To visualize inequalities in the prevalence of the feeding indicators by levels of mother’s education we used equiplots, which includes a horizontal line to link dots that represent each education category. Among all surveys, we selected the most recent one for each country included in the trend analysis, grouping the countries by income groups and regions of the world.

### Country specific analysis

We conducted a data validation check by evaluating the relationship between the prevalence of the formula feeding indicators and per capita annual sales of commercial milk formulas sourced from Euromonitor International, for one country per region of the world [30]. Annual sales volume data of formula (kg) were obtained from Euromonitor from 2005 to 2019 for standard (0–6 months), follow-on (6–12 months), and growing-up (13–36 months) categories. These market categories are defined by Euromonitor, when in reality a much wider range of age-specific products are available in markets [31]. Our criteria for selecting these countries was as follows: they must have contributed at least three-point estimates in our nearly 20-year trend analysis, have non-modeled data on formula volume sales (data based on actual sales in each country, not estimated by statistical methods), and have a large population of children less than 2 years (over one million children). Per capita sales volumes were generated using age-specific ranges of population size in each country from the World Bank [27]. Furthermore, AAAC were estimated for each studied country through the same methods used in the pooled trend analysis. For each country, Euromonitor per capita volume sales in kg are depicted as line graphs over the period. We also graphed bar charts to visualize annual changes in p.p. in feeding indicators.

### Changes in women’s education

To document changes over the last decades for some indicators related to women’s education, we obtained data from the World Bank for the following indicators: Literacy rate – women 15+ years (%): the percentage of women ages 15 and above who can both read and write with understanding a short simple statement about their everyday life; and School enrollment female – primary (net %): the ratio of children of official school age who are enrolled in school to the population of the corresponding official school age [26]. We graphed trends over 18-year period for LMICs using weighted local polynomials [32].

All analyses were run using Stata 16.0 (Stata Corp.).

## Results

Categorization of feeding indicators by maternal formal educational level was available for 319 surveys with 5 years spacing between the earliest and the latest survey (159 surveys in low income countries (49.8%), 115 surveys in lower-middle income countries (36.0%), and 45 surveys in upper-middle income countries (14.2%)). The sample size of children under 2 years ranged from 197 (Dominican Republic - DHS 1999) to 97,935 (India - DHS 2015). The median number of children under 2 years in the samples slightly changed over the period from 2479 (IQR 1450–3677) in the 1990’s, to 2757 (IQR 1544–4149) in the 2000’s, and 2769 (IQR 1606–4155) in the 2010’s. Supplementary tables 2, 3 and 4 detail the prevalence of the breastfeeding and formula indicators by categories of maternal formal education for each survey included in the analysis, along with the national prevalence.

### Inequalities in feeding indicators by mother’s formal education level

**Fig. 3:**
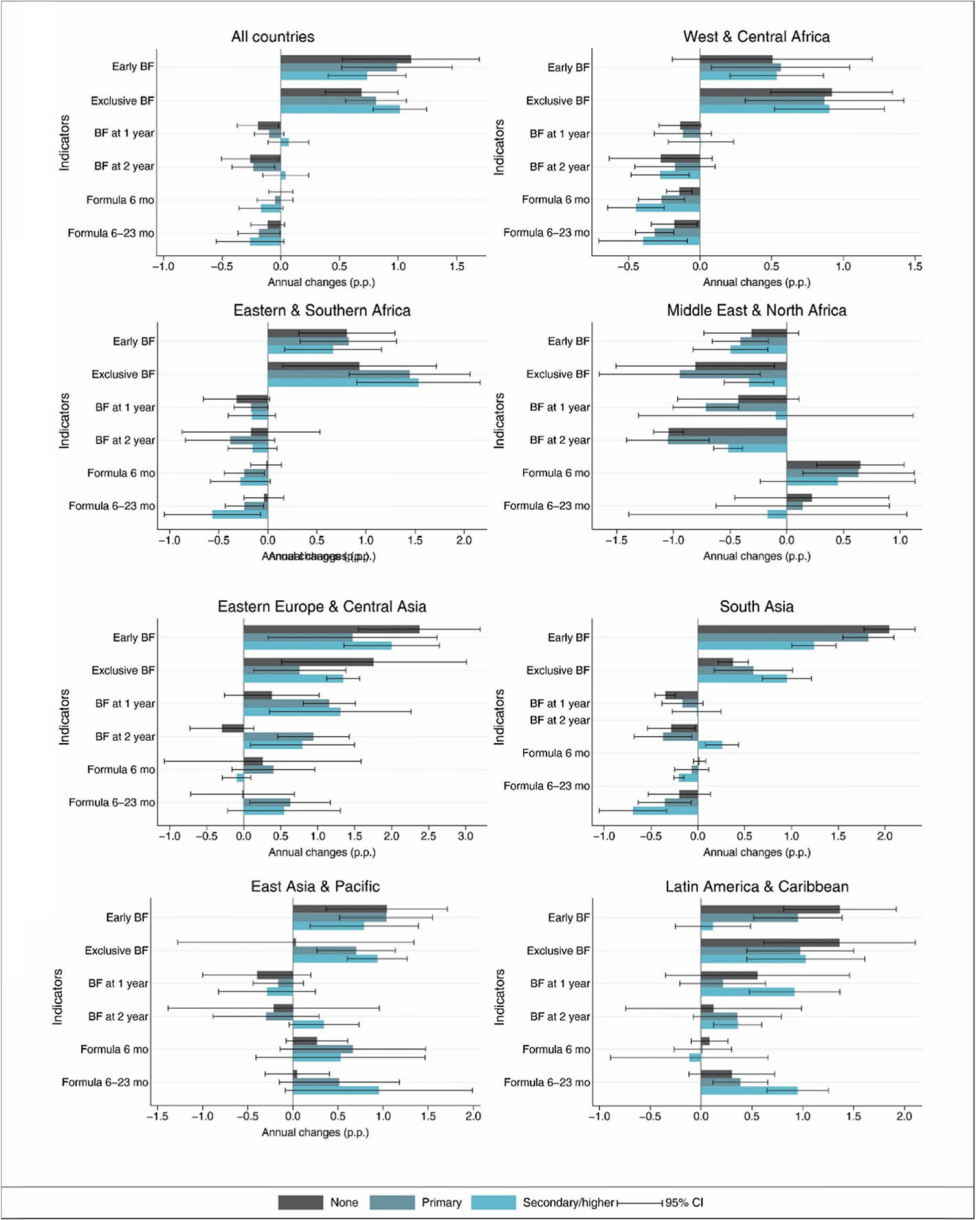
Average absolute annual changes in percentage points (p.p.) in breast milk and formula consumption indicators for all countries and by regions of the world. Early BF – Early initiation of breastfeeding; Exclusive BF – Exclusive breastfeeding under 6 months; BF at 1 year - Continued breastfeeding at 1 year; BF at 2 years – Continued breastfeeding at 2 years; Formula 6 months – Consumption of formula under 6 months; Formula 6–23 months – Consumption of formula between 6 and 23 months

National level AAAC of each feeding indicator by categories of maternal education are shown in Fig. 3 and Table 1. Significant increases over time in prevalence were observed for early initiation and exclusive breastfeeding across all education categories, but more prominently in women with no education for early breastfeeding (AAAC: 1.11 p.p., 95% CI: 0.53; 1.69), and in higher level educated women for exclusive breastfeeding (AAAC: 1.02 p.p., 95% CI: 0.79; 1.24). Small decreases in prevalence were seen mostly for women with no formal education for continued breastfeeding at 1 year (AAAC: − 0.29 p.p., 95% CI: − 0.37; − 0.02) and 2 years (AAAC: − 0.26 p.p, 95% CI: − 0.51; − 0.02). Among formula indicators, only formula consumption between 6 and 23 months decreased significantly over the period for women with primary education (AAAC: -0.19, 95% CI: − 0.37; − 0.01).

**Table 1:** Average absolute annual changes (AAAC) in percentage points in the prevalence of breast milk and formula consumption indicators by mother’s formal education level. Source: Demographic Health Survey and Multiple Indicator Cluster Survey

Indicator	Formal education level											
	None				Primary				Secondary or higher			
	AAAC	95% CI		P	AAAC	95% CI		P	AAAC	95% CI		P
Early initiation of breastfeeding	**1.11**	**0.53**	**1.69**	**< 0.001**	**0.99**	**0.52**	**1.47**	**< 0.001**	**0.74**	**0.41**	**1.07**	**< 0.001**
Exclusive breastfeeding under 6 months	**0.69**	**0.38**	**1.00**	**< 0.001**	**0.81**	**0.55**	**1.07**	**< 0.001**	**1.02**	**0.79**	**1.24**	**< 0.001**
Continued breastfeeding at 1 year	**-0.19**	**−0.37**	**− 0.02**	**0.030**	− 0.10	− 0.22	0.03	0.118	0.07	−0.11	0.24	0.456
Continued breastfeeding at 2 years	**−0.26**	**−0.51**	**− 0.02**	**0.037**	**− 0.23**	**−0.42**	**− 0.05**	**0.012**	0.04	−0.16	0.24	0.689
Formula consumption under 6 months	0.00	−0.10	0.10	0.963	−0.05	−0.20	0.10	0.522	−0.17	−0.35	0.02	0.074
Formula consumption between 6 and 23 months	−0.11	− 0.25	0.03	0.126	**−0.19**	**− 0.37**	**−0.01**	**0.041**	−0.26	− 0.55	0.03	0.076

Bold figures represent statistical significance at *P* < 0.05.

Analysis by regions of the world demonstrates that gains in early and exclusive breastfeeding were almost universally distributed among all education categories in all regions, except in the Middle East and North Africa where these indicators decreased throughout education categories (Supplementary table 5 and Fig. 3). Continued breastfeeding at 1 and 2 years increased in South Asia, Latin America and the Caribbean, and Eastern Europe and Central Asia for primary or higher education categories. In contrast, declines occurred for the group of no formal education in South Asia and nearly all education categories in the Middle East and North Africa with the decline steeper for continued breastfeeding at 2 years.

With respect to country income classifications, increases in early and exclusive breastfeeding occurred in almost all education categories in low- and upper-middle income countries; increases in continued breastfeeding at 1 and 2 years were mainly observed for the secondary or higher education category in upper- and lower-middle income countries, while women with no formal education in low-income countries had declines for both indicators of continued breastfeeding. The consumption of formula under 6 months only increased significantly for women with primary education in upper-middle income countries, and decreased significantly in all education categories in low income countries. Significant increases in formula consumption occurred solely in older infants and young children of mothers with primary or higher levels of education in upper-middle income countries. The inverse pattern occurred in low income countries and for the category no formal education in lower-middle income countries (Supplementary table 6 and supplementary figure 1).

**Fig. 4:**
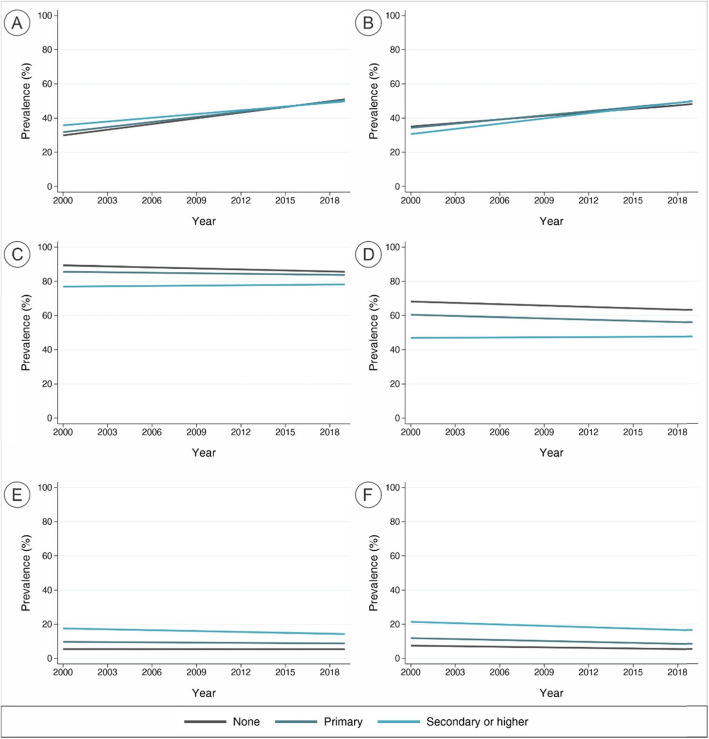
Trends over the 20-years period in breast milk and formula consumption indicators for all countries. **a** – Early initiation of breastfeeding; **b** – Exclusive breastfeeding under 6 months; **c** – Continued breastfeeding at 1 year; **d** – Continued breastfeeding at 2 years; **e** – Consumption of formula under 6 months; **f** – Consumption of formula between 6 and 23 months

Prevalence of early and exclusive breastfeeding for all countries increased from, on average, 30% to over 50% in all categories of education (Fig. 4a-b). On average, the prevalence of continued breastfeeding at 1 and 2 years (Fig. 4c-d), and formula consumption in all age ranges (Fig. 4e-f) for all education categories remained high (80% or higher), medium (~ 60%), and low (< 20%), respectively. Patterns of breastfeeding indicators by income groups and regions of the world are graphically shown in supplementary figures 2 and 3.

**Fig. 5:**
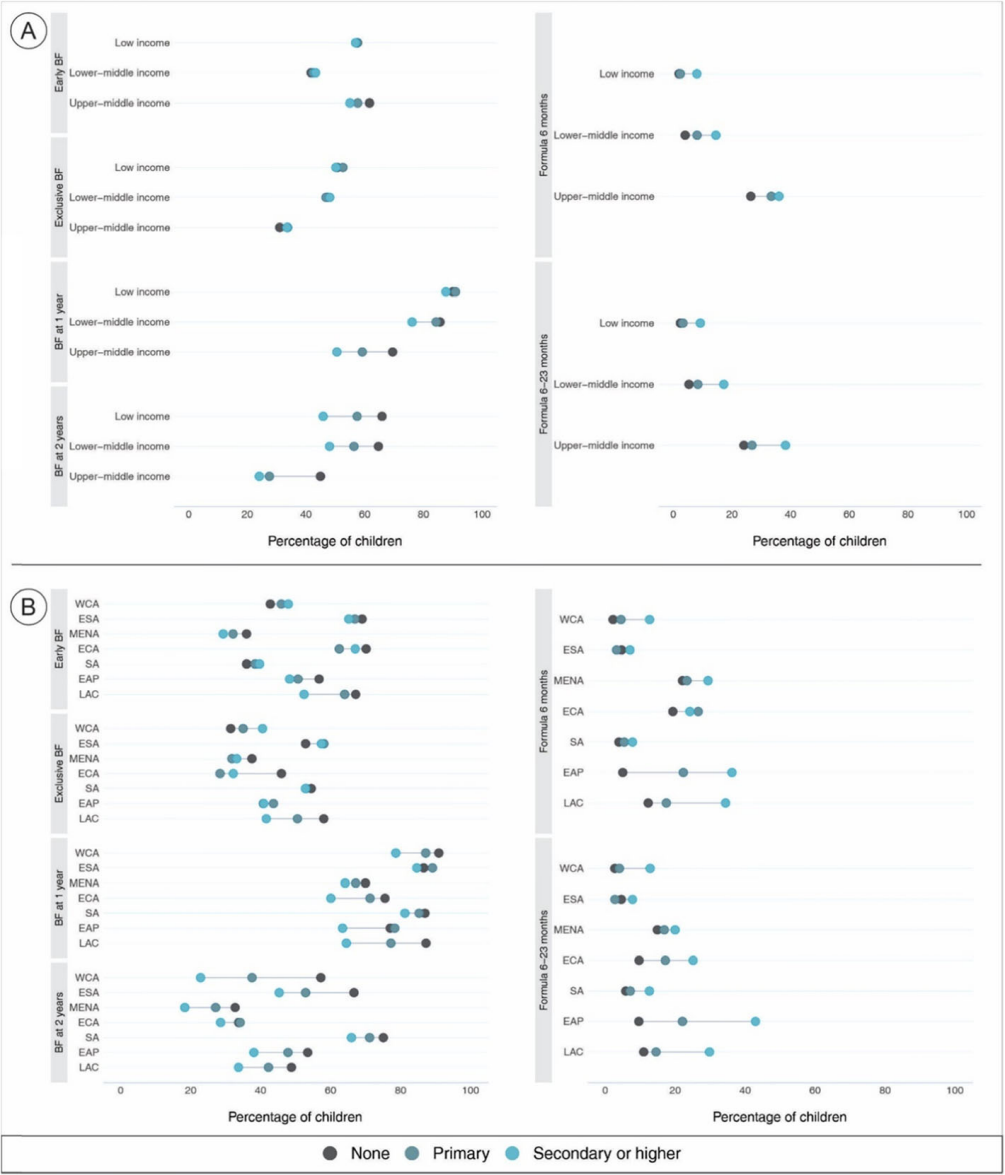
Average prevalence of breast milk and formula consumption indicators for the most recent survey included in the trend analysis by (**a**) country income level and (**b**) regions of the world. Early BF – Early initiation of breastfeeding; Exclusive BF – Exclusive breastfeeding under 6 months; BF at 1 year - Continued breastfeeding at 1 year; BF at 2 years – Continued breastfeeding at 2 years; Formula 6 months – Consumption of formula under 6 months; Formula 6–23 months – Consumption of formula between 6 and 23 months; WCA – West & Central Africa; ESA – Eastern & Southern Africa; MENA – Middle East & Central Africa; ECA – Eastern Europe & Central Asia; SA – South Asia; EAP – East Asia & Pacific; LAC – Latin America & Caribbean

Figure 5 and supplementary tables 7-8 show the average proportion of children within specific age-ranges of each feeding indicators by categories of formal education according to income groups and regions of the world. No salient inequalities in early and exclusive breastfeeding were observed across income groups, except for a slightly higher prevalence among women with no or primary education compared to women with secondary or higher education in early initiation of breastfeeding in upper-middle income countries. Wide disparities in continued breastfeeding were apparent in all but one income category, with higher prevalences in women with no formal education followed by women with primary education. The only exception was for breastfeeding at 1 year in low income countries where prevalences were virtually the same. The widest disparities in continued breastfeeding at 1 year were seen in upper-middle income countries with a difference of ~ 20% between the highest prevalence in the category of no formal education and the lowest in the category of secondary or higher education. Monotonic associations in continued breastfeeding at 2 years appeared in all income groups, with an evident higher prevalence among children of women with no formal education compared to secondary or higher educated women. Concerning formula use, its consumption is consistently higher among children of women with higher education levels. For all formula consumption indicators, the lowest prevalence in the upper-middle income countries (~ 22%) was persistently higher across the indicators than the top highest prevalence in all other income groups (~ 18%). On average, in upper-middle income countries, the prevalence of formula use remained above 20% in all education categories in the entire age distribution (Fig. 5a).

Regarding the regions of the world, among all breastfeeding indicators, Latin America and the Caribbean, East Asia and the Pacific, and Eastern Europe and Central Asia consistently exhibited wider inequalities between groups with no formal education and those with middle and higher formal education. In the Middle East and North Africa, the inequalities were narrowed, but persisted across all breastfeeding indicators. West and Central Africa presented the largest differences among education categories for continued breastfeeding at 1 and 2 years; at 1 year, the gap between the categories no formal education to those with higher level of formal education is ~ 12%, and increased to ~ 35% at 2 years. Despite a few exceptions, the use of formula in the entire child’s age distribution is systematically higher among children of women at the highest education level in all regions. Important differences are shown in Latin America and the Caribbean; however, the largest differences were observed in East Asia and the Pacific where consumption of formula among children of women with secondary or higher education is about 35% greater than for children of women with no formal education. More than 40% of children between 6 and 23 months of age of women with the highest levels of education were formula fed in this region (Fig. 5b).

### Country specific analysis

The following countries were included in this analysis as they met our criteria for selection: Bangladesh, Egypt, Kenya, Nigeria, Peru, Turkey, and Vietnam.

Supplementary figure 4 and supplementary table 9 illustrate the AAAC variation in breast milk and formula consumption indicators in these countries. Significant increases in early initiation of breastfeeding occurred in all education categories in Bangladesh, and for the no and primary education categories in Peru and Turkey. Exclusive breastfeeding significantly increased among children of women with no formal education in Turkey, primary, secondary or higher education in Kenya and Bangladesh, and among all education categories in Peru. Turkey and Peru showed substantial increases in continued breastfeeding at 1 year in the primary or higher levels of education, though decreases in continued breastfeeding at 2 years for categories no and primary of education were seen in Bangladesh, Egypt, and Turkey. Conversely, Turkey was the only country where breastfeeding practices at 2 years increased over the period in the primary and secondary or higher education categories.

Peru, Turkey, and Vietnam displayed significant increases in the consumption of formula, being more pronounced in Vietnam, where AAAC surpassed 2 p.p. for children of mothers with secondary or higher education in all formula indicators. In contrast, in Bangladesh and Egypt formula use declined over the period for children of mothers with the highest level of formal education (supplementary figure 4 and supplementary table 9). In Nigeria, no indicator had marked changes throughout the period of analysis.

Euromonitor data show that increases in per capita formula sales were more pronounced in Peru, Turkey, and Vietnam compared to the other countries. Vietnam particularly stands out compared to the other countries with respect to large increases in the growth in sales of all Euromonitor’s formula categories. Sales of follow-on and growing-up formula surpassed sales of standard infant formula category, especially in the last decade (Supplementary figure 5).

### Changes in women’s education

Over the last 18 years, there were improvements in women’s literacy and primary school enrollment in all LMICs, especially in the last decade. Primary school enrollment among girls increased from less than 60% in low-income countries to over 80%. In lower- middle-income countries, primary school enrollment among girls increased from slightly less than 80% to about 87% whereas it remained virtually unchanged among girls in upper-middle income countries at 95%. Yet, major gaps still remain, as a large number of women are illiterate and girls are not enrolled in primary school in low income and lower-middle countries compared to upper-middle income countries (Supplementary figure 6).

## Discussion

Clear patterns were identified with respect to early initiation of breastfeeding and exclusive breastfeeding with significant increases over the past 19 years in all categories of formal education. The largest increases for early initiation were observed among women with no formal education and the smallest among women with secondary or higher education. A reverse pattern was observed in exclusive breastfeeding with the largest increases among women with secondary or higher education and the smallest among women with no formal education.

For the other breastfeeding indicators, findings were mixed though troubling trends were observed in continued breastfeeding at 1 and 2 years with significant declines among women with no formal education. Trends were also negative among women with primary education though only significant for continued breastfeeding at 2 years. For women with secondary or higher education, the coefficient was positive though not significant. All coefficients for formula consumption were negative, except for one, though only formula 6–23 months among women with primary education was significant. The only education category for which the coefficient was not negative was for formula consumption under 6 months among women with no formal education that neither positive or negative.

The significant increases in early initiation and exclusive breastfeeding are consistent with the program and policy emphasis on these practices by the global nutrition and health communities [33]. Increasing the prevalence of exclusive breastfeeding is one of six global nutrition targets endorsed by the World Health Assembly. Over the period of our study, the Baby Friendly Hospital Initiative, launched in 1991, has been widely promoted by UNICEF and WHO and one of the 10 steps for certification as a Baby Friendly hospital is early initiation of breastfeeding [34]. Larger improvements in early initiation for women with no formal education compared to women in the higher education categories may reflect increased births in facilities where early initiation has been promoted rather than at home [35]. In 2002, WHO changed its recommendation for the duration of exclusive breastfeeding from 4 to 6 months to 6 months and the indicator started being tracked by national surveys [36]. In contrast to the global drive to increase early initiation of breastfeeding and exclusive breastfeeding, efforts to promote continued breastfeeding at 1 and 2 years have been far less visible. Our study showing significant declines among some education categories and lack of increase in the others likely reflects the lack of policy and program focus on these practices. The finding of significant declines of continued breastfeeding at 1 and 2 years among women with no or lower levels of formal education compared to those with higher levels is consistent with that reported by Lutter and colleagues who found that gains in the duration of breastfeeding favored women with higher levels of formal education in Bolivia, Brazil, Colombia, Haiti, and Peru [5].

The same pattern of increases in early initiation of breastfeeding and exclusive breastfeeding and declines in continued breastfeeding at 1 and 2 years were observed in the majority of world regions with several notable exceptions. In the Middle East and North Africa, all breastfeeding indicators in all education categories declined. In Latin America and the Caribbean increases in continued breastfeeding were observed in most education categories. Changes in formula consumption were varied. It declined in all education categories in West and Central Africa, Eastern and Southern Africa, and South Asia. Increases were generally observed in Eastern Europe and Central Asia, the Middle East and North Africa, and East Asia and the Pacific. In Latin America and the Caribbean, formula use increased only in the 6–23 months category. The equiplots, summarizing the most recent surveys, show that for both early initiation and exclusive breastfeeding there are few differences by education category. For continued breastfeeding at 1 year, the plots show no difference by education category for low-income countries but lower prevalence among women with the highest level of education compared to the other two education categories. For continued breastfeeding at 2 years, a traditional gradient remains with women with no formal education with higher rates, and women high secondary or higher education with the lowest rates. As with the trend data, the convergence of early initiation and exclusive breastfeeding among the education categories likely reflects the global focus on these two practices compared to continued breastfeeding at 1 and 2 years. The equiplots for formula use reflect the typical pattern of greater formula use among women with secondary or higher education, which reflects greater economic resources for purchasing breast-milk substitutes [15]. The plots by region illustrate very wide variation in all breastfeeding and formula feeding indicators, reflecting different normative behaviors by region. Nonetheless, they generally show higher levels for the breastfeeding indicators for women with no formal education compared to the other education categories and higher levels of formula use for women in the highest education category compared to the lower two education levels reflecting traditionally expected patterns.

Not unexpectedly, we show that literacy rates, which are likely associated with levels of education, have increased in all three income groups over the past 18 years with the largest increases occurring in low- and lower middle-income countries compared to women in upper middle-income countries. Increased female education in LMICs is associated with increased labor force participation [37]. Returning to work is also associated with poorer breastfeeding practices [38, 39]. Therefore, as levels of women’s formal education and labor force increase, protective policies are needed to support breastfeeding. Yet, such supportive policies are lacking in many countries and even when legislated are inadequately implemented resulting in low coverage. Mothers, usually those with the lowest levels of education, working in the informal sector usually have no protections whatsoever [40] although there are efforts underway to cost providing this benefit in some countries [41, 42].

Somewhat paradoxically, a concomitant increase in exclusive breastfeeding and formula consumption under 6 months and breastfeeding and formula consumption at 1 and 2 years with increased maternal education was observed in the regions of Eastern Europe and Central Asia and Latin America and the Caribbean. This may to some degree reflect an increase in mixed-feeding practices in these countries. It is also possible that in these regions, the apparent choice of either breastfeeding or formula feeding among educated women reflects an inverse equity hypothesis, whereby early adopters include families with greater access to information about the benefits of breastfeeding practices and to health services which provide breastfeeding promotion, while at the same time continuing the practice of formula feeding. In this case, the possibility of improvement in breastfeeding practices among more educated women could be considered a ‘trickle up’ phenomenon, since health messages had been primarily directed to the poorest and less educated population [43]. As noted above, the improvement in maternal education is strongly associated with increased participation of women in the labor force. The use of formula is likely seen as an alternative for mothers who return to work and face barriers to maintaining breastfeeding. While continuing to breastfeed when they are with their child, these mothers may resort to the use of infant formula while at work [18].

Our observation of a modest overall decline in formula consumption for all countries combined, contrasts with studies reporting a remarkable increase in world milk formula market sales since at least the mid-2000s, especially in the follow-up and toddler formula categories [19]. For example, annual world milk formula sales grew by 121.5% between 2005 and 2019, from 3.5 to 7.4 kg per child, led by growth in the highly-populated middle-income countries of East and South East Asia [31]. However, our results showing growth in formula consumption in the East Asia & Pacific, Middle East & North Africa, Eastern Europe & Central Asia, and Latin America & Caribbean regions, is consistent with the findings of these studies.

Although the MICS and DHS questionnaires have a separate question for infant formula and DHS tabulates the results in their open access Statcompiler as well as the DHS reports [44], an indicator to capture formula feeding was not adopted by the Technical Expert Advisory Group on Nutrition Monitoring (TEAM) [21]. Including the regular monitoring of such and indicator should be revisited in the future.

Our paper has some limitations. These include the lack of data for high-income countries and some upper-middle income countries with large populations of children under 2 years - in particular Brazil, Mexico, and China, that represent important and expanding markets for formula companies. China is now by far the world’s largest market for commercial milk formulas, accounting for more than one third of world sales [31]. Our data have captured formula consumption among infants and young children between the ages of 0–24 months, which misses young children consuming toddler milks up to the age of 36 months. This is a key limitation given toddler milks are now more important than infant and follow-up formulas in terms of worldwide sales volume [31]. Another limitation is the lack of more recent surveys for Bolivia, the Philippines, and Uzbekistan for which the most recent survey in our data set was carried out more than 10 years ago. Although two other DHS surveys are available for the Philippines (2013 and 2017) they do not have data to generate all the indicators in our analysis and so were not included in our dataset. Finally, most of the information available for this analysis came from low and lower middle-income countries, with only nearly 15% of the surveys from upper middle-income countries.

Despite these limitations, the strengths of our analyses include the use of 319 national surveys from 81 countries conducted periodically over a nearly 20-year period in LMICs, which are highly comparable in methods and field procedures. The feeding data, with the exception of early initition of breastfeeding, was collected through 24-h food recall, thus minimizing recall bias. Some countries, especially in Eastern Europe and Central Asia, presented none or a very small number of women with no formal education, which could affect our results; however, the multilevel statistical approach employed reduced any noise caused by such pattern, by considering each country separately as the highest and independent level before generating an overall average estimate of change throughout the period of analysis.

At national-level, significant improvements in early and exclusive breastfeeding for all maternal education categories likely resulted from years of global and national policies and programs promoting and supporting these behaviors. Declines in continued breastfeeding likely reflect the absence of a similar focus on these behaviors. Patterns for formula consumption did not change significantly in the overall analysis, but some regions presented large increases throughout the period primarily where most of the upper middle-income countries are located. This might reflect the differential marketing strategies by formula companies in leveraging and expanding the markets to these regions in the world, especially among women with higher levels of education.

Globally, over the course of our study women with no formal education appear to have worsening breastfeeding indicators compared to women with primary and secondary or higher education. Although women with no formal education had larger increases in early initiation of breastfeeding, the coefficients for improvements in exclusive breastfeeding were lower than for the other education categories. Furthermore, no formal education was the only education category with significant declines in continued breastfeeding at 1 year. Although declines in continued breastfeeding at 2 years were significant in both women with no formal education and primary education, the coefficient for women with no formal education was larger. These results suggest that while the equity gap for breastfeeding is decreasing, the equity gap for child health outcomes as a result of breastfeeding is possibly increasing as children of women with no formal education are at higher risk of morbidity and mortality in the absence of breastfeeding. Because the ultimate public health goal is to increase both breastfeeding and positive child health outcomes, the policy implications of our paper include the need for expanded maternity benefits to include women in both the formal and informal sectors that include longer periods of paid leave after childbirth to facilitate exclusive breastfeeding and increased support for breastfeeding in the workplace, including properly equipped breastfeeding rooms and paid breaks to express breast milk [45]. In addition, there are other strategies to make it easier for women to breastfeeding such as the Baby Friendly Hospital Initiative, health worker training in lactation management, and community-based promotion among others [46]. Full implementation and routine monitoring of the International Code of Marketing of Breast-milk Substitutes are also indicated [47]. Other important measures include a strong coordination mechanism at the country level and regular monitoring of breastfeding practices using available tools [48, 49].

## Conclusion

The main findings of our paper are twofold. First, Over the course of our study, women with no formal education have worsening breastfeeding indicators compared to women with primary and secondary or higher education. This is concerning in that children of women with no formal education are most likely to live in impoverished environments and therefore at higher risk of morbity and mortality from less breastfeeding compared to children of women with higher levels of education. Second, while early initiation and exclusive have generally increased, this is not the case for continued breastfeeding at 1 and 2 years. Therefore, increased promotion of these practices is warrented.

## Supplementary Information

**Additional file 1: Table S1.** Countries and surveys included in the trend and inequalities analyses by mother’s formal education level. Source: Demographic Health Survey and Multiple Indicator Cluster Survey, 1993–2019. **Table S2.** Percentage of children who were put to breast within 1 h after birth and of children exclusively breastfed under 6 months by mother’s formal education level. Source: Demographic Health Survey and Multiple Indicator Cluster Survey. **Table S3.** Percentage of children at 1 and 2 years of age who were fed breastmilk by mother’s formal education level. Source: DHS and MICS. **Table S4.** Percentage of children under 6 months and between 6 and 23 months of age who were fed formula by mother’s formal education level. Source: Demographic Health Survey and Multiple Indicator Cluster Survey. **Table S5.** Annual changes in the prevalence of breast milk and formula consumption indicators by mother’s formal education level according to the regions of the world. Source: Demographic Health Survey and Multiple Indicator Cluster Survey. **Table S6.** Annual changes in the prevalence of breast milk and formula consumption indicators by mother’s formal education level according to the World Bank income groups. Source: Demographic Health Survey and Multiple Indicator Cluster Survey. **Table S7.** Average weighted prevalence of breast milk and formula consumption indicators by mother’s formal education level according to income groups^*^. **Table S8.** Average weighted prevalence of breast milk and formula consumption indicators by mother’s formal education level according to regions of the world^*^. **Table S9.** Annual changes in the prevalence of breast milk and formula consumption indicators by mother’s formal education level for selected countries. Source: Demographic Health Survey and Multiple Indicator Cluster Survey.

**Additional file 2: Figure S1.** Average absolute annual changes in breast milk and formula consumption indicators by income groups. **Figure S2.** Trends over the 20-years period in breast milk and formula consumption indicators by income groups. **Figure S3.** Trends over the 20-years period in breast milk and formula consumption indicators by regions of the world. **Figure S4.** Average absolute annual changes in breast milk and formula consumption indicators for selected countries. **Figure S5.** Per capita sales of standard (0–5 months), follow-on (6–12 months), and growing-up (13–36 months) formula from Euromonitor International for selected countries. **Figure S6.** Changes over the 18-year period in (A) literacy rate for women 15 years or above and (B) school enrollment in primary education for girls.

## Data Availability

Not applicable.

## Abbreviations

AAAC: Absolute average annual changes
BMS: Breast milk substitutes
DHS: Demographic Health Surveys
IYCF: Infant and young child feeding
IQR: Interquartile interval
LMICs: Low- and middle-income countries
MICS: Multiple Indicator Cluster Surveys
p.p.: Percentage points
TEAM: Technical Expert Advisory Group on Nutrition Monitoring
UNICEF: United Nations Children’s Fund
WHO: World Health Organization
