# Supplementary material for: Maternal education and equity in breastfeeding: trends and patterns in 81 low- and middle-income countries between 2000 and 2019
Source: Int J Equity Health. 2021 Jan 7;20:20. doi: 10.1186/s12939-020-01357-3 (PMC7792102; doi:10.1186/s12939-020-01357-3)
Supplement: Supplementary file 1 — Additional file 1: Table S1. Countries and surveys included in the trend and inequalities analyses by mother’s formal education level. Source: Demographic Health Survey and Multiple Indicator Cluster Survey, 1993–2019. Table S2. Percentage of children who were put to breast within 1 h after birth and of children exclusively breastfed under 6 months by mother’s formal education level. Source: Demographic Health Survey and Multiple Indicator Cluster Survey. Table S3. Percentage of children at 1 and 2 years of age who were fed breastmilk by mother’s formal education level. Source: DHS and MICS. Table S4. Percentage of children under 6 months and between 6 and 23 months of age who were fed formula by mother’s formal education level. Source: Demographic Health Survey and Multiple Indicator Cluster Survey. Table S5. Annual changes in the prevalence of breast milk and formula consumption indicators by mother’s formal education level according to the regions of the world. Source: Demographic Health Survey and Multiple Indicator Cluster Survey. Table S6. Annual changes in the prevalence of breast milk and formula consumption indicators by mother’s formal education level according to the World Bank income groups. Source: Demographic Health Survey and Multiple Indicator Cluster Survey. Table S7. Average weighted prevalence of breast milk and formula consumption indicators by mother’s formal education level according to income groups*. Table S8. Average weighted prevalence of breast milk and formula consumption indicators by mother’s formal education level according to regions of the world*. Table S9. Annual changes in the prevalence of breast milk and formula consumption indicators by mother’s formal education level for selected countries. Source: Demographic Health Survey and Multiple Indicator Cluster Survey. [file 12939_2020_1357_MOESM1_ESM.docx]

**Supplementary table 1.** Countries and surveys included in the trend and inequalities analyses by mother’s formal education level. Source: Demographic Health Survey and Multiple Indicator Cluster Survey, 1993-2019.

| Country | ISO^1^ | Survey and year | Income group^2^ | Region of the world | Number of children under 6 months | Number of children between 12-15 months | Number of children between 20-23 months | Number of children between 6-23 months | Number of children under 2 years |
| --- | --- | --- | --- | --- | --- | --- | --- | --- | --- |
| Afghanistan | AFG | MICS 2010 | LI | South Asia | 1270 | 1033 | 556 | 3635 | 4962 |
| Afghanistan | AFG | DHS 2015 | LI | South Asia | 3203 | 2467 | 964 | 8078 | 11762 |
| Albania | ALB | MICS 2005 | LMI | Eastern Europe & Central Asia | 99 | 84 | 58 | 273 | 395 |
| Albania | ALB | DHS 2008 | LMI | Eastern Europe & Central Asia | 138 | 94 | 74 | 386 | 533 |
| Albania | ALB | DHS 2017 | UMI | Eastern Europe & Central Asia | 285 | 188 | 173 | 766 | 1056 |
| Armenia | ARM | DHS 2000 | LI | Eastern Europe & Central Asia | 155 | 101 | 92 | 435 | 603 |
| Armenia | ARM | DHS 2005 | LMI | Eastern Europe & Central Asia | 157 | 88 | 82 | 391 | 559 |
| Armenia | ARM | DHS 2010 | LMI | Eastern Europe & Central Asia | 156 | 111 | 80 | 464 | 628 |
| Armenia | ARM | DHS 2015 | LMI | Eastern Europe & Central Asia | 177 | 118 | 86 | 499 | 682 |
| Bangladesh | BGD | DHS 1993 | LI | South Asia | 616 | 498 | 304 | 1688 | 2466 |
| Bangladesh | BGD | DHS 1996 | LI | South Asia | 612 | 431 | 296 | 1593 | 2341 |
| Bangladesh | BGD | DHS 1999 | LI | South Asia | 746 | 518 | 367 | 1786 | 2671 |
| Bangladesh | BGD | DHS 2004 | LI | South Asia | 679 | 474 | 310 | 1791 | 2586 |
| Bangladesh | BGD | MICS 2006 | LI | South Asia | 2300 | 1803 | 2304 | 9453 | 11942 |
| Bangladesh | BGD | DHS 2007 | LI | South Asia | 510 | 337 | 356 | 1718 | 2308 |
| Bangladesh | BGD | DHS 2011 | LI | South Asia | 789 | 547 | 477 | 2356 | 3250 |
| Bangladesh | BGD | MICS 2012 | LI | South Asia | 1959 | 1553 | 1129 | 5968 | 7866 |
| Bangladesh | BGD | DHS 2014 | LMI | South Asia | 632 | 552 | 486 | 2336 | 3078 |
| Bangladesh | BGD | MICS 2019 | LMI | South Asia | 2370 | 1538 | 1306 | 6691 | 9285 |
| Belize | BLZ | MICS 2006 | UMI | Latin America & Caribbean | 88 | 38 | 54 | 239 | 312 |
| Belize | BLZ | MICS 2011 | LMI | Latin America & Caribbean | 145 | 114 | 156 | 610 | 702 |
| Belize | BLZ | MICS 2015 | UMI | Latin America & Caribbean | 165 | 209 | 147 | 730 | 916 |
| Benin | BEN | DHS 1996 | LI | West & Central Africa | 518 | 334 | 251 | 1370 | 1999 |
| Benin | BEN | DHS 2001 | LI | West & Central Africa | 527 | 312 | 288 | 1436 | 2092 |
| Benin | BEN | DHS 2006 | LI | West & Central Africa | 1525 | 1086 | 820 | 4593 | 6432 |
| Benin | BEN | DHS 2011 | LI | West & Central Africa | 1195 | 822 | 792 | 3739 | 5178 |
| Benin | BEN | MICS 2014 | LI | West & Central Africa | 1284 | 856 | 744 | 3639 | 5052 |
| Benin | BEN | DHS 2017 | LI | West & Central Africa | 1381 | 764 | 821 | 3884 | 5486 |
| Bolivia | BOL | DHS 1994 | LMI | Latin America & Caribbean | 556 | 343 | 320 | 1622 | 2328 |
| Bolivia | BOL | DHS 1998 | LMI | Latin America & Caribbean | 629 | 440 | 385 | 1984 | 2757 |
| Bolivia | BOL | DHS 2003 | LMI | Latin America & Caribbean | 867 | 664 | 541 | 2703 | 3738 |
| Bolivia | BOL | DHS 2008 | LMI | Latin America & Caribbean | 791 | 556 | 528 | 2461 | 3383 |
| Bosnia and Herzegovina | BIH | MICS 2006 | LMI | Eastern Europe & Central Asia | 186 | 201 | 248 | 1008 | 1174 |
| Bosnia and Herzegovina | BIH | MICS 2011 | UMI | Eastern Europe & Central Asia | 117 | 156 | 186 | 635 | 718 |
| Burkina Faso | BFA | DHS 1998 | LI | West & Central Africa | 618 | 373 | 274 | 1491 | 2297 |
| Burkina Faso | BFA | DHS 2003 | LI | West & Central Africa | 1071 | 659 | 493 | 2782 | 4121 |
| Burkina Faso | BFA | MICS 2006 | LI | West & Central Africa | 543 | 466 | 271 | 1708 | 2384 |
| Burkina Faso | BFA | DHS 2010 | LI | West & Central Africa | 1454 | 986 | 820 | 4148 | 5860 |
| Burundi | BDI | DHS 2010 | LI | Eastern & Southern Africa | 685 | 493 | 455 | 2196 | 3030 |
| Burundi | BDI | DHS 2016 | LI | Eastern & Southern Africa | 1236 | 894 | 749 | 3858 | 5261 |
| CAR^3^ | CAF | DHS 1994 | LI | West & Central Africa | 458 | 299 | 220 | 1215 | 1821 |
| CAR^3^ | CAF | MICS 2006 | LI | West & Central Africa | 1266 | 764 | 410 | 2759 | 4129 |
| CAR^3^ | CAF | MICS 2010 | LI | West & Central Africa | 1283 | 768 | 617 | 3266 | 4545 |
| Cambodia | KHM | DHS 2000 | LI | East Asia & the Pacific | 873 | 501 | 381 | 2099 | 3210 |
| Cambodia | KHM | DHS 2005 | LI | East Asia & the Pacific | 788 | 542 | 492 | 2329 | 3268 |
| Cambodia | KHM | DHS 2010 | LI | East Asia & the Pacific | 717 | 567 | 485 | 2368 | 3215 |
| Cambodia | KHM | DHS 2014 | LI | East Asia & the Pacific | 688 | 450 | 486 | 2127 | 2899 |
| Cameroon | CMR | DHS 1998 | LI | West & Central Africa | 389 | 234 | 199 | 1009 | 1514 |
| Cameroon | CMR | DHS 2004 | LI | West & Central Africa | 794 | 517 | 416 | 2150 | 3184 |
| Cameroon | CMR | MICS 2006 | LMI | West & Central Africa | 658 | 520 | 375 | 2053 | 2878 |
| Cameroon | CMR | DHS 2011 | LMI | West & Central Africa | 1123 | 796 | 577 | 3287 | 4702 |
| Cameroon | CMR | MICS 2014 | LMI | West & Central Africa | 703 | 493 | 436 | 2128 | 2899 |
| Cameroon | CMR | DHS 2018 | LMI | West & Central Africa | 986 | 640 | 452 | 2576 | 3788 |
| Chad | TCD | DHS 1996 | LI | West & Central Africa | 807 | 510 | 272 | 1813 | 2851 |
| Chad | TCD | DHS 2004 | LI | West & Central Africa | 583 | 394 | 197 | 1371 | 2116 |
| Chad | TCD | MICS 2010 | LI | West & Central Africa | 1867 | 1141 | 941 | 4579 | 6153 |
| Chad | TCD | DHS 2014 | LI | West & Central Africa | 1823 | 1094 | 713 | 4403 | 6590 |
| Colombia | COL | DHS 1995 | LMI | Latin America & Caribbean | 440 | 322 | 305 | 1454 | 1951 |
| Colombia | COL | DHS 2000 | LMI | Latin America & Caribbean | 430 | 276 | 259 | 1335 | 1812 |
| Colombia | COL | DHS 2005 | LMI | Latin America & Caribbean | 1397 | 926 | 845 | 4110 | 5688 |
| Colombia | COL | DHS 2010 | UMI | Latin America & Caribbean | 1515 | 1089 | 1061 | 5023 | 6700 |
| Comoros | COM | DHS 1996 | LI | Eastern & Southern Africa | 186 | 136 | 79 | 522 | 751 |
| Comoros | COM | DHS 2012 | LI | Eastern & Southern Africa | 327 | 210 | 141 | 869 | 1255 |
| CDR^4^ | COD | DHS 2007 | LI | West & Central Africa | 907 | 631 | 414 | 2355 | 3515 |
| CDR^4^ | COD | MICS 2010 | LI | West & Central Africa | 1271 | 846 | 763 | 3617 | 4809 |
| CDR^4^ | COD | DHS 2013 | LI | West & Central Africa | 1934 | 1294 | 863 | 4991 | 7322 |
| CDR^4^ | COD | MICS 2017 | LI | West & Central Africa | 2106 | 1607 | 1276 | 6499 | 8560 |
| Cote d'Ivoire | CIV | DHS 1994 | LI | West & Central Africa | 658 | 367 | 334 | 1710 | 2546 |
| Cote d'Ivoire | CIV | DHS 1998 | LI | West & Central Africa | 166 | 140 | 112 | 583 | 811 |
| Cote d'Ivoire | CIV | MICS 2006 | LI | West & Central Africa | 950 | 584 | 531 | 2765 | 3711 |
| Cote d'Ivoire | CIV | DHS 2011 | LMI | West & Central Africa | 775 | 471 | 443 | 2145 | 3113 |
| Cote d'Ivoire | CIV | MICS 2016 | LMI | West & Central Africa | 981 | 657 | 538 | 2668 | 3749 |
| Dominican Republic | DOM | DHS 1996 | LMI | Latin America & Caribbean | 415 | 310 | 238 | 1205 | 1705 |
| Dominican Republic | DOM | DHS 1999 | LMI | Latin America & Caribbean | 52 | 21 | 25 | 140 | 197 |
| Dominican Republic | DOM | DHS 2002 | LMI | Latin America & Caribbean | 1003 | 698 | 676 | 3098 | 4325 |
| Dominican Republic | DOM | DHS 2007 | LMI | Latin America & Caribbean | 986 | 530 | 634 | 2915 | 4125 |
| Dominican Republic | DOM | DHS 2013 | UMI | Latin America & Caribbean | 307 | 253 | 229 | 1048 | 1430 |
| Dominican Republic | DOM | MICS 2014 | UMI | Latin America & Caribbean | 1656 | 1082 | 1512 | 6235 | 7566 |
| Egypt | EGY | DHS 1995 | LMI | Middle East & North Africa | 1138 | 760 | 666 | 3182 | 4499 |
| Egypt | EGY | DHS 2000 | LMI | Middle East & North Africa | 1201 | 744 | 579 | 3127 | 4461 |
| Egypt | EGY | DHS 2005 | LMI | Middle East & North Africa | 1250 | 851 | 811 | 3891 | 5283 |
| Egypt | EGY | DHS 2008 | LMI | Middle East & North Africa | 1130 | 699 | 649 | 3324 | 4535 |
| Egypt | EGY | DHS 2014 | LMI | Middle East & North Africa | 1487 | 1018 | 1007 | 4834 | 6410 |
| Eswatini | SWZ | DHS 2006 | LMI | Eastern & Southern Africa | 258 | 193 | 148 | 767 | 1152 |
| Eswatini | SWZ | MICS 2010 | LMI | Eastern & Southern Africa | 269 | 169 | 192 | 773 | 1018 |
| Eswatini | SWZ | MICS 2014 | LMI | Eastern & Southern Africa | 235 | 191 | 171 | 789 | 987 |
| Ethiopia | ETH | DHS 2011 | LI | Eastern & Southern Africa | 1187 | 676 | 475 | 2850 | 4284 |
| Ethiopia | ETH | DHS 2016 | LI | Eastern & Southern Africa | 1092 | 755 | 462 | 2822 | 4081 |
| Gabon | GAB | DHS 2000 | UMI | West & Central Africa | 451 | 310 | 180 | 1183 | 1776 |
| Gabon | GAB | DHS 2012 | UMI | West & Central Africa | 631 | 381 | 356 | 1719 | 2511 |
| Gambia | GMB | MICS 2005 | LI | West & Central Africa | 855 | 605 | 401 | 2177 | 3093 |
| Gambia | GMB | MICS 2010 | LI | West & Central Africa | 1412 | 773 | 755 | 3790 | 5222 |
| Gambia | GMB | DHS 2013 | LI | West & Central Africa | 951 | 640 | 412 | 2424 | 3481 |
| Gambia | GMB | MICS 2018 | LI | West & Central Africa | 910 | 725 | 490 | 2724 | 3796 |
| Georgia | GEO | MICS 2005 | LMI | Eastern Europe & Central Asia | 171 | 137 | 112 | 577 | 760 |
| Georgia | GEO | MICS 2018 | UMI | Eastern Europe & Central Asia | 234 | 161 | 163 | 701 | 908 |
| Ghana | GHA | DHS 1993 | LI | West & Central Africa | 379 | 203 | 190 | 968 | 1430 |
| Ghana | GHA | DHS 1998 | LI | West & Central Africa | 302 | 224 | 189 | 950 | 1318 |
| Ghana | GHA | DHS 2003 | LI | West & Central Africa | 338 | 274 | 197 | 1102 | 1519 |
| Ghana | GHA | MICS 2006 | LI | West & Central Africa | 384 | 239 | 224 | 1043 | 1459 |
| Ghana | GHA | DHS 2008 | LI | West & Central Africa | 318 | 203 | 144 | 857 | 1225 |
| Ghana | GHA | MICS 2011 | LMI | West & Central Africa | 792 | 489 | 455 | 2171 | 2873 |
| Ghana | GHA | DHS 2014 | LMI | West & Central Africa | 606 | 362 | 337 | 1656 | 2329 |
| Ghana | GHA | MICS 2017 | LMI | West & Central Africa | 891 | 543 | 539 | 2585 | 3466 |
| Guatemala | GTM | DHS 1995 | LMI | Latin America & Caribbean | 969 | 682 | 510 | 2820 | 3962 |
| Guatemala | GTM | DHS 1998 | LMI | Latin America & Caribbean | 476 | 273 | 259 | 1324 | 1872 |
| Guatemala | GTM | DHS 2014 | LMI | Latin America & Caribbean | 1175 | 825 | 715 | 3509 | 4789 |
| Guinea | GIN | DHS 1999 | LI | West & Central Africa | 662 | 416 | 156 | 1321 | 2178 |
| Guinea | GIN | DHS 2005 | LI | West & Central Africa | 733 | 483 | 292 | 1650 | 2586 |
| Guinea | GIN | DHS 2012 | LI | West & Central Africa | 718 | 532 | 291 | 1953 | 2810 |
| Guinea | GIN | MICS 2016 | LI | West & Central Africa | 686 | 514 | 391 | 2122 | 2794 |
| Guinea | GIN | DHS 2018 | LI | West & Central Africa | 916 | 687 | 271 | 1909 | 3066 |
| Guinea Bissau | GNB | MICS 2006 | LI | West & Central Africa | 658 | 555 | 280 | 1837 | 2455 |
| Guinea Bissau | GNB | MICS 2014 | LI | West & Central Africa | 830 | 573 | 489 | 2268 | 3196 |
| Guyana | GUY | MICS 2006 | LMI | Latin America & Caribbean | 219 | 148 | 142 | 716 | 917 |
| Guyana | GUY | DHS 2009 | LMI | Latin America & Caribbean | 230 | 135 | 109 | 607 | 869 |
| Guyana | GUY | MICS 2014 | LMI | Latin America & Caribbean | 290 | 204 | 200 | 1034 | 1258 |
| Haiti | HTI | DHS 1994 | LI | Latin America & Caribbean | 317 | 210 | 179 | 931 | 1375 |
| Haiti | HTI | DHS 2000 | LI | Latin America & Caribbean | 563 | 402 | 343 | 1797 | 2551 |
| Haiti | HTI | DHS 2005 | LI | Latin America & Caribbean | 598 | 420 | 306 | 1691 | 2443 |
| Haiti | HTI | DHS 2012 | LI | Latin America & Caribbean | 726 | 447 | 341 | 1972 | 2907 |
| Haiti | HTI | DHS 2016 | LI | Latin America & Caribbean | 700 | 425 | 285 | 1652 | 2535 |
| Honduras | HND | DHS 2005 | LMI | Latin America & Caribbean | 973 | 741 | 565 | 3066 | 4153 |
| Honduras | HND | DHS 2011 | LMI | Latin America & Caribbean | 1084 | 769 | 649 | 3237 | 4449 |
| India | IND | DHS 2005 | LI | South Asia | 4616 | 3132 | 2627 | 13904 | 19275 |
| India | IND | DHS 2015 | LMI | South Asia | 22626 | 16241 | 14279 | 71762 | 97935 |
| Indonesia | IDN | DHS 2002 | LI | East Asia & the Pacific | 1641 | 1094 | 820 | 4413 | 6295 |
| Indonesia | IDN | DHS 2007 | LMI | East Asia & the Pacific | 1802 | 1225 | 1011 | 5209 | 7274 |
| Indonesia | IDN | DHS 2012 | LMI | East Asia & the Pacific | 1686 | 1174 | 1081 | 5193 | 7141 |
| Indonesia | IDN | DHS 2017 | LMI | East Asia & the Pacific | 1666 | 1202 | 1066 | 5033 | 6925 |
| Iraq | IRQ | MICS 2006 | LMI | Middle East & North Africa | 1624 | 1315 | 1134 | 5352 | 6535 |
| Iraq | IRQ | MICS 2011 | LMI | Middle East & North Africa | 3882 | 2765 | 2287 | 11168 | 13994 |
| Iraq | IRQ | MICS 2018 | UMI | Middle East & North Africa | 1681 | 1106 | 990 | 4786 | 6250 |
| Jamaica | JAM | MICS 2005 | LMI | Latin America & Caribbean | 129 | 94 | 99 | 412 | 538 |
| Jamaica | JAM | MICS 2011 | UMI | Latin America & Caribbean | 167 | 118 | 89 | 476 | 630 |
| Jordan | JOR | DHS 1997 | LMI | Middle East & North Africa | 499 | 399 | 330 | 1767 | 2321 |
| Jordan | JOR | DHS 2002 | LMI | Middle East & North Africa | 507 | 385 | 309 | 1728 | 2280 |
| Jordan | JOR | DHS 2007 | LMI | Middle East & North Africa | 1117 | 626 | 513 | 2739 | 3895 |
| Jordan | JOR | DHS 2012 | UMI | Middle East & North Africa | 850 | 636 | 579 | 2783 | 3678 |
| Jordan | JOR | DHS 2017 | UMI | Middle East & North Africa | 1218 | 581 | 536 | 2680 | 3958 |
| Kazakhstan | KAZ | DHS 1995 | LMI | Eastern Europe & Central Asia | 118 | 108 | 85 | 403 | 539 |
| Kazakhstan | KAZ | DHS 1999 | LMI | Eastern Europe & Central Asia | 99 | 88 | 67 | 349 | 470 |
| Kazakhstan | KAZ | MICS 2006 | UMI | Eastern Europe & Central Asia | 387 | 321 | 302 | 1437 | 1784 |
| Kazakhstan | KAZ | MICS 2015 | UMI | Eastern Europe & Central Asia | 508 | 381 | 361 | 1632 | 2106 |
| Kenya | KEN | DHS 1993 | LI | Eastern & Southern Africa | 518 | 380 | 318 | 1645 | 2305 |
| Kenya | KEN | DHS 1998 | LI | Eastern & Southern Africa | 518 | 367 | 329 | 1624 | 2300 |
| Kenya | KEN | DHS 2003 | LI | Eastern & Southern Africa | 599 | 383 | 298 | 1610 | 2357 |
| Kenya | KEN | DHS 2008 | LI | Eastern & Southern Africa | 587 | 346 | 315 | 1656 | 2373 |
| Kenya | KEN | DHS 2014 | LMI | Eastern & Southern Africa | 856 | 666 | 523 | 5792 | 3821 |
| Kyrgyzstan | KGZ | DHS 1997 | LI | Eastern Europe & Central Asia | 177 | 132 | 115 | 521 | 726 |
| Kyrgyzstan | KGZ | MICS 2005 | LI | Eastern Europe & Central Asia | 286 | 250 | 152 | 874 | 1152 |
| Kyrgyzstan | KGZ | DHS 2012 | LI | Eastern Europe & Central Asia | 447 | 318 | 221 | 1317 | 1805 |
| Kyrgyzstan | KGZ | MICS 2014 | LMI | Eastern Europe & Central Asia | 432 | 288 | 299 | 1402 | 1766 |
| Kyrgyzstan | KGZ | MICS 2018 | LMI | Eastern Europe & Central Asia | 390 | 221 | 217 | 992 | 1346 |
| Lao | LAO | MICS 2006 | LI | East Asia & the Pacific | 445 | 326 | 265 | 1201 | 1622 |
| Lao | LAO | MICS 2011 | LMI | East Asia & the Pacific | 1168 | 802 | 645 | 3265 | 4444 |
| Lao | LAO | MICS 2017 | LMI | East Asia & the Pacific | 1134 | 755 | 769 | 3428 | 4460 |
| Lesotho | LSO | DHS 2004 | LI | Eastern & Southern Africa | 399 | 257 | 173 | 982 | 1534 |
| Lesotho | LSO | DHS 2009 | LMI | Eastern & Southern Africa | 434 | 297 | 218 | 1118 | 1712 |
| Lesotho | LSO | DHS 2014 | LMI | Eastern & Southern Africa | 327 | 260 | 170 | 948 | 1387 |
| Lesotho | LSO | MICS 2018 | LMI | Eastern & Southern Africa | 215 | 217 | 238 | 1009 | 1175 |
| Liberia | LBR | DHS 2007 | LI | West & Central Africa | 504 | 309 | 294 | 1519 | 2197 |
| Liberia | LBR | DHS 2013 | LI | West & Central Africa | 718 | 472 | 418 | 2155 | 3064 |
| Madagascar | MDG | DHS 1997 | LI | Eastern & Southern Africa | 628 | 387 | 262 | 1638 | 2419 |
| Madagascar | MDG | DHS 2003 | LI | Eastern & Southern Africa | 499 | 356 | 269 | 1538 | 2149 |
| Madagascar | MDG | DHS 2008 | LI | Eastern & Southern Africa | 1214 | 754 | 643 | 3306 | 4704 |
| Madagascar | MDG | MICS 2018 | LI | Eastern & Southern Africa | 1386 | 934 | 764 | 3859 | 5239 |
| Malawi | MWI | DHS 2000 | LI | Eastern & Southern Africa | 1242 | 777 | 655 | 3312 | 4971 |
| Malawi | MWI | DHS 2004 | LI | Eastern & Southern Africa | 1099 | 797 | 611 | 3327 | 4662 |
| Malawi | MWI | MICS 2006 | LI | Eastern & Southern Africa | 2298 | 1803 | 1609 | 7668 | 10374 |
| Malawi | MWI | DHS 2010 | LI | Eastern & Southern Africa | 1663 | 1174 | 1266 | 5688 | 7755 |
| Malawi | MWI | MICS 2013 | LI | Eastern & Southern Africa | 1686 | 1394 | 1178 | 5661 | 7576 |
| Malawi | MWI | DHS 2015 | LI | Eastern & Southern Africa | 1636 | 1118 | 1023 | 4747 | 6683 |
| Maldives | MDV | DHS 2009 | LMI | South Asia | 414 | 267 | 279 | 1256 | 1686 |
| Maldives | MDV | DHS 2016 | UMI | South Asia | 288 | 199 | 202 | 848 | 1168 |
| Mali | MLI | DHS 1995 | LI | West & Central Africa | 1036 | 612 | 426 | 2475 | 3866 |
| Mali | MLI | DHS 2001 | LI | West & Central Africa | 1418 | 984 | 475 | 3372 | 5259 |
| Mali | MLI | DHS 2006 | LI | West & Central Africa | 1421 | 1001 | 608 | 3780 | 5581 |
| Mali | MLI | MICS 2009 | LI | West & Central Africa | 2913 | 1877 | 1582 | 7816 | 10598 |
| Mali | MLI | DHS 2012 | LI | West & Central Africa | 999 | 584 | 543 | 2795 | 3952 |
| Mali | MLI | MICS 2015 | LI | West & Central Africa | 1663 | 1290 | 787 | 4865 | 6584 |
| Mali | MLI | DHS 2018 | LI | West & Central Africa | 997 | 644 | 468 | 2713 | 3926 |
| Mauritania | MRT | MICS 2007 | LI | West & Central Africa | 915 | 720 | 446 | 2693 | 3539 |
| Mauritania | MRT | MICS 2011 | LI | West & Central Africa | 995 | 657 | 499 | 2749 | 3629 |
| Mauritania | MRT | MICS 2015 | LMI | West & Central Africa | 915 | 884 | 561 | 3184 | 4172 |
| Moldova | MDA | DHS 2005 | LMI | Eastern Europe & Central Asia | 158 | 133 | 94 | 486 | 651 |
| Moldova | MDA | MICS 2012 | LMI | Eastern Europe & Central Asia | 176 | 112 | 131 | 591 | 723 |
| Mongolia | MNG | MICS 2005 | LI | East Asia & the Pacific | 399 | 210 | 208 | 1098 | 1460 |
| Mongolia | MNG | MICS 2010 | LMI | East Asia & the Pacific | 410 | 314 | 245 | 1323 | 1690 |
| Mongolia | MNG | MICS 2013 | LMI | East Asia & the Pacific | 644 | 382 | 377 | 1802 | 2375 |
| Mongolia | MNG | MICS 2018 | LMI | East Asia & the Pacific | 616 | 379 | 324 | 1674 | 2199 |
| Montenegro | MNE | MICS 2005 | UMI | Eastern Europe & Central Asia | 71 | 62 | 61 | 284 | 350 |
| Montenegro | MNE | MICS 2013 | UMI | Eastern Europe & Central Asia | 108 | 82 | 85 | 388 | 494 |
| Montenegro | MNE | MICS 2018 | UMI | Eastern Europe & Central Asia | 101 | 65 | 73 | 332 | 432 |
| Mozambique | MOZ | DHS 2003 | LI | Eastern & Southern Africa | 1027 | 663 | 538 | 2805 | 4145 |
| Mozambique | MOZ | MICS 2008 | LI | Eastern & Southern Africa | 1229 | 879 | 670 | 3652 | 4904 |
| Mozambique | MOZ | DHS 2011 | LI | Eastern & Southern Africa | 1044 | 770 | 605 | 3280 | 4612 |
| Namibia | NAM | DHS 2006 | LMI | Eastern & Southern Africa | 495 | 345 | 251 | 1396 | 2135 |
| Namibia | NAM | DHS 2013 | UMI | Eastern & Southern Africa | 525 | 311 | 239 | 1303 | 2051 |
| Nepal | NPL | DHS 1996 | LI | South Asia | 677 | 505 | 408 | 2038 | 2878 |
| Nepal | NPL | DHS 2006 | LI | South Asia | 473 | 331 | 339 | 1542 | 2103 |
| Nepal | NPL | DHS 2011 | LI | South Asia | 497 | 316 | 273 | 1421 | 1980 |
| Nepal | NPL | MICS 2014 | LI | South Asia | 452 | 336 | 322 | 1556 | 2086 |
| Nepal | NPL | DHS 2016 | LI | South Asia | 467 | 340 | 347 | 1463 | 1970 |
| Niger | NER | DHS 1998 | LI | West & Central Africa | 816 | 540 | 351 | 2063 | 3113 |
| Niger | NER | DHS 2006 | LI | West & Central Africa | 945 | 674 | 404 | 2479 | 3613 |
| Niger | NER | DHS 2012 | LI | West & Central Africa | 1303 | 926 | 533 | 3260 | 4759 |
| Nigeria | NGA | DHS 1999 | LI | West & Central Africa | 555 | 578 | 242 | 1620 | 2359 |
| Nigeria | NGA | DHS 2003 | LI | West & Central Africa | 611 | 391 | 267 | 1586 | 2395 |
| Nigeria | NGA | MICS 2007 | LI | West & Central Africa | 1729 | 1750 | 658 | 4788 | 6307 |
| Nigeria | NGA | DHS 2008 | LMI | West & Central Africa | 2889 | 2085 | 1094 | 7557 | 11228 |
| Nigeria | NGA | MICS 2011 | LMI | West & Central Africa | 2714 | 1888 | 1529 | 7528 | 10036 |
| Nigeria | NGA | DHS 2013 | LMI | West & Central Africa | 2934 | 2304 | 1466 | 8721 | 12397 |
| Nigeria | NGA | MICS 2016 | LMI | West & Central Africa | 2748 | 2042 | 1499 | 8219 | 11204 |
| Nigeria | NGA | DHS 2018 | LMI | West & Central Africa | 3193 | 2266 | 1445 | 8883 | 12818 |
| North Macedonia | MKD | MICS 2005 | LMI | Eastern Europe & Central Asia | 233 | 317 | 271 | 1199 | 1436 |
| North Macedonia | MKD | MICS 2011 | UMI | Eastern Europe & Central Asia | 112 | 87 | 88 | 410 | 503 |
| Pakistan | PAK | DHS 2012 | LMI | South Asia | 1076 | 802 | 432 | 2870 | 4155 |
| Pakistan | PAK | DHS 2017 | LMI | South Asia | 1117 | 701 | 422 | 2566 | 3872 |
| Peru | PER | DHS 1996 | LMI | Latin America & Caribbean | 1519 | 1112 | 969 | 4729 | 6510 |
| Peru | PER | DHS 2000 | LMI | Latin America & Caribbean | 1162 | 844 | 778 | 3699 | 5010 |
| Peru | PER | DHS 2004 | LMI | Latin America & Caribbean | 233 | 173 | 159 | 722 | 985 |
| Peru | PER | DHS 2005 | LMI | Latin America & Caribbean | 273 | 184 | 186 | 801 | 1102 |
| Peru | PER | DHS 2006 | LMI | Latin America & Caribbean | 251 | 198 | 181 | 842 | 1120 |
| Peru | PER | DHS 2007 | LMI | Latin America & Caribbean | 223 | 162 | 178 | 752 | 993 |
| Peru | PER | DHS 2008 | UMI | Latin America & Caribbean | 545 | 413 | 379 | 1834 | 2425 |
| Peru | PER | DHS 2009 | UMI | Latin America & Caribbean | 901 | 699 | 614 | 3027 | 4013 |
| Peru | PER | DHS 2010 | UMI | Latin America & Caribbean | 766 | 603 | 574 | 2706 | 3538 |
| Peru | PER | DHS 2011 | UMI | Latin America & Caribbean | 780 | 589 | 556 | 2557 | 3389 |
| Peru | PER | DHS 2012 | UMI | Latin America & Caribbean | 827 | 600 | 619 | 2729 | 3620 |
| Peru | PER | DHS 2013 | UMI | Latin America & Caribbean | 750 | 581 | 551 | 2580 | 3389 |
| Peru | PER | DHS 2014 | UMI | Latin America & Caribbean | 878 | 670 | 577 | 2767 | 3718 |
| Peru | PER | DHS 2015 | UMI | Latin America & Caribbean | 1847 | 1648 | 1550 | 7040 | 8966 |
| Peru | PER | DHS 2016 | UMI | Latin America & Caribbean | 1446 | 1405 | 1352 | 6198 | 7723 |
| Peru | PER | DHS 2017 | UMI | Latin America & Caribbean | 1792 | 1482 | 1434 | 6580 | 8471 |
| Peru | PER | DHS 2018 | UMI | Latin America & Caribbean | 1737 | 1481 | 1407 | 6534 | 8340 |
| Philippines | PHL | DHS 1993 | LMI | East Asia & the Pacific | 821 | 572 | 436 | 2481 | 3404 |
| Philippines | PHL | DHS 1998 | LMI | East Asia & the Pacific | 757 | 496 | 368 | 2137 | 2992 |
| Philippines | PHL | DHS 2003 | LMI | East Asia & the Pacific | 624 | 445 | 399 | 1940 | 2665 |
| Philippines | PHL | DHS 2008 | LMI | East Asia & the Pacific | 593 | 434 | 384 | 1818 | 2492 |
| Rwanda | RWA | DHS 2000 | LI | Eastern & Southern Africa | 757 | 511 | 249 | 2115 | 3092 |
| Rwanda | RWA | DHS 2005 | LI | Eastern & Southern Africa | 876 | 590 | 442 | 2340 | 3412 |
| Rwanda | RWA | DHS 2010 | LI | Eastern & Southern Africa | 709 | 506 | 510 | 2333 | 3159 |
| Rwanda | RWA | DHS 2014 | LI | Eastern & Southern Africa | 703 | 502 | 453 | 2354 | 3169 |
| Sao Tome and Principe | STP | DHS 2008 | LMI | West & Central Africa | 192 | 109 | 118 | 563 | 778 |
| Sao Tome and Principe | STP | MICS 2014 | LMI | West & Central Africa | 169 | 137 | 129 | 571 | 758 |
| Senegal | SEN | DHS 2005 | LI | West & Central Africa | 1290 | 701 | 526 | 3060 | 4551 |
| Senegal | SEN | DHS 2010 | LMI | West & Central Africa | 1334 | 912 | 594 | 3375 | 4911 |
| Senegal | SEN | DHS 2012 | LMI | West & Central Africa | 672 | 460 | 356 | 1935 | 2710 |
| Senegal | SEN | DHS 2014 | LMI | West & Central Africa | 612 | 403 | 397 | 1885 | 2584 |
| Senegal | SEN | DHS 2015 | LI | West & Central Africa | 626 | 434 | 377 | 1984 | 2712 |
| Senegal | SEN | DHS 2016 | LI | West & Central Africa | 605 | 429 | 396 | 1940 | 2641 |
| Senegal | SEN | DHS 2017 | LI | West & Central Africa | 1142 | 734 | 752 | 3489 | 4818 |
| Serbia | SRB | MICS 2005 | UMI | Eastern Europe & Central Asia | 334 | 277 | 259 | 1129 | 1436 |
| Serbia | SRB | MICS 2010 | UMI | Eastern Europe & Central Asia | 246 | 241 | 223 | 1011 | 1187 |
| Serbia | SRB | MICS 2014 | UMI | Eastern Europe & Central Asia | 169 | 146 | 183 | 795 | 959 |
| Sierra Leone | SLE | MICS 2005 | LI | West & Central Africa | 508 | 375 | 267 | 1592 | 2356 |
| Sierra Leone | SLE | DHS 2008 | LI | West & Central Africa | 617 | 443 | 218 | 1537 | 2410 |
| Sierra Leone | SLE | MICS 2010 | LI | West & Central Africa | 831 | 463 | 468 | 2442 | 3415 |
| Sierra Leone | SLE | DHS 2013 | LI | West & Central Africa | 1115 | 803 | 435 | 3088 | 4668 |
| Sierra Leone | SLE | MICS 2017 | LI | West & Central Africa | 1170 | 808 | 716 | 3411 | 4744 |
| South Africa | ZAF | DHS 1998 | LMI | Eastern & Southern Africa | 505 | 295 | 270 | 1374 | 2041 |
| South Africa | ZAF | DHS 2016 | UMI | Eastern & Southern Africa | 346 | 203 | 172 | 877 | 1376 |
| Suriname | SUR | MICS 2006 | LMI | Latin America & Caribbean | 182 | 136 | 144 | 659 | 798 |
| Suriname | SUR | MICS 2010 | UMI | Latin America & Caribbean | 304 | 187 | 294 | 1062 | 1265 |
| Suriname | SUR | MICS 2018 | UMI | Latin America & Caribbean | 335 | 250 | 241 | 1182 | 1395 |
| Tajikistan | TJK | MICS 2005 | LI | Eastern Europe & Central Asia | 388 | 311 | 244 | 1275 | 1622 |
| Tajikistan | TJK | DHS 2012 | LI | Eastern Europe & Central Asia | 424 | 346 | 286 | 1467 | 1938 |
| Tajikistan | TJK | DHS 2017 | LI | Eastern Europe & Central Asia | 553 | 416 | 355 | 1722 | 2321 |
| Tanzania | TZA | DHS 1996 | LI | Eastern & Southern Africa | 654 | 458 | 360 | 1920 | 2770 |
| Tanzania | TZA | DHS 2004 | LI | Eastern & Southern Africa | 825 | 522 | 474 | 2408 | 3390 |
| Tanzania | TZA | DHS 2010 | LI | Eastern & Southern Africa | 803 | 508 | 433 | 2235 | 3173 |
| Tanzania | TZA | DHS 2015 | LI | Eastern & Southern Africa | 1015 | 724 | 587 | 3020 | 4219 |
| Thailand | THA | MICS 2005 | LMI | East Asia & the Pacific | 873 | 661 | 572 | 2913 | 3365 |
| Thailand | THA | MICS 2012 | UMI | East Asia & the Pacific | 591 | 720 | 631 | 2618 | 2762 |
| Thailand | THA | MICS 2015 | UMI | East Asia & the Pacific | 661 | 804 | 848 | 3222 | 3382 |
| Timor-Leste | TLS | DHS 2009 | LMI | East Asia & the Pacific | 960 | 631 | 444 | 2570 | 3674 |
| Timor-Leste | TLS | DHS 2016 | LMI | East Asia & the Pacific | 743 | 491 | 343 | 1950 | 2837 |
| Togo | TGO | DHS 1998 | LI | West & Central Africa | 692 | 383 | 382 | 1878 | 2713 |
| Togo | TGO | MICS 2006 | LI | West & Central Africa | 447 | 325 | 282 | 1341 | 1751 |
| Togo | TGO | MICS 2010 | LI | West & Central Africa | 563 | 323 | 299 | 1392 | 1961 |
| Togo | TGO | DHS 2013 | LI | West & Central Africa | 603 | 477 | 416 | 2070 | 2782 |
| Togo | TGO | MICS 2017 | LI | West & Central Africa | 504 | 345 | 301 | 1461 | 1988 |
| Tunisia | TUN | MICS 2011 | UMI | Middle East & North Africa | 306 | 176 | 183 | 854 | 1135 |
| Tunisia | TUN | MICS 2018 | LMI | Middle East & North Africa | 299 | 198 | 234 | 946 | 1212 |
| Turkey | TUR | DHS 1993 | LMI | Eastern Europe & Central Asia | 362 | 256 | 178 | 1034 | 1447 |
| Turkey | TUR | DHS 1998 | UMI | Eastern Europe & Central Asia | 370 | 273 | 170 | 1012 | 1425 |
| Turkey | TUR | DHS 2003 | LMI | Eastern Europe & Central Asia | 413 | 271 | 207 | 1156 | 1606 |
| Turkey | TUR | DHS 2013 | UMI | Eastern Europe & Central Asia | 332 | 241 | 195 | 1053 | 1401 |
| Turkmenistan | TKM | MICS 2006 | LMI | Eastern Europe & Central Asia | 237 | 136 | 147 | 625 | 846 |
| Turkmenistan | TKM | MICS 2015 | UMI | Eastern Europe & Central Asia | 342 | 270 | 240 | 1169 | 1467 |
| Uganda | UGA | DHS 1995 | LI | Eastern & Southern Africa | 629 | 497 | 405 | 2079 | 2922 |
| Uganda | UGA | DHS 2000 | LI | Eastern & Southern Africa | 656 | 441 | 389 | 1994 | 2831 |
| Uganda | UGA | DHS 2006 | LI | Eastern & Southern Africa | 800 | 525 | 460 | 2236 | 3239 |
| Uganda | UGA | DHS 2011 | LI | Eastern & Southern Africa | 778 | 457 | 410 | 2069 | 3019 |
| Uganda | UGA | DHS 2016 | LI | Eastern & Southern Africa | 1482 | 883 | 840 | 4160 | 5992 |
| Ukraine | UKR | MICS 2005 | LMI | Eastern Europe & Central Asia | 229 | 204 | 191 | 907 | 1128 |
| Ukraine | UKR | DHS 2007 | LMI | Eastern Europe & Central Asia | 98 | 85 | 50 | 338 | 438 |
| Ukraine | UKR | MICS 2012 | LMI | Eastern Europe & Central Asia | 307 | 310 | 276 | 1256 | 1564 |
| Uzbekistan | UZB | DHS 1996 | LMI | Eastern Europe & Central Asia | 164 | 147 | 128 | 650 | 841 |
| Uzbekistan | UZB | MICS 2006 | LI | Eastern Europe & Central Asia | 446 | 352 | 373 | 1663 | 2095 |
| Vietnam | VNM | DHS 1997 | LI | East Asia & the Pacific | 237 | 187 | 236 | 873 | 1132 |
| Vietnam | VNM | DHS 2002 | LI | East Asia & the Pacific | 191 | 168 | 125 | 642 | 839 |
| Vietnam | VNM | MICS 2006 | LI | East Asia & the Pacific | 229 | 200 | 185 | 808 | 1023 |
| Vietnam | VNM | MICS 2010 | LMI | East Asia & the Pacific | 319 | 276 | 241 | 1110 | 1363 |
| Vietnam | VNM | MICS 2013 | LMI | East Asia & the Pacific | 358 | 265 | 246 | 1118 | 1484 |
| Zambia | ZMB | DHS 1996 | LI | Eastern & Southern Africa | 676 | 495 | 393 | 1923 | 2872 |
| Zambia | ZMB | DHS 2001 | LI | Eastern & Southern Africa | 650 | 435 | 409 | 1923 | 2769 |
| Zambia | ZMB | DHS 2007 | LI | Eastern & Southern Africa | 618 | 411 | 397 | 1829 | 2615 |
| Zambia | ZMB | DHS 2013 | LMI | Eastern & Southern Africa | 1189 | 863 | 773 | 3722 | 5096 |
| Zambia | ZMB | DHS 2018 | LMI | Eastern & Southern Africa | 1019 | 636 | 603 | 2785 | 3958 |
| Zimbabwe | ZWE | DHS 1994 | LI | Eastern & Southern Africa | 400 | 227 | 210 | 1063 | 1554 |
| Zimbabwe | ZWE | DHS 1999 | LI | Eastern & Southern Africa | 347 | 247 | 215 | 1000 | 1462 |
| Zimbabwe | ZWE | DHS 2005 | LI | Eastern & Southern Africa | 534 | 379 | 267 | 1445 | 2132 |
| Zimbabwe | ZWE | MICS 2009 | LI | Eastern & Southern Africa | 681 | 469 | 477 | 2147 | 2850 |
| Zimbabwe | ZWE | DHS 2010 | LI | Eastern & Southern Africa | 641 | 412 | 255 | 1649 | 2437 |
| Zimbabwe | ZWE | MICS 2014 | LI | Eastern & Southern Africa | 867 | 641 | 697 | 2965 | 3913 |
| Zimbabwe | ZWE | DHS 2015 | LI | Eastern & Southern Africa | 603 | 379 | 328 | 1628 | 2367 |
| Zimbabwe | ZWE | MICS 2019 | LMI | Eastern & Southern Africa | 605 | 397 | 387 | 1737 | 2338 |

^1^ISO: International Organization for Standardization; ^2^LI: Low income countries; LMI: Lower middle-income countries; UMI: Upper middle-income countries. ^3^CAR: Central African Republic; ^4^CDR: Congo Democratic Republic.

**Supplementary table 2.** Percentage of children who were put to breast within 1 hour after birth and of children exclusively breastfed under six months by mother’s formal education level. Source: Demographic Health Survey and Multiple Indicator Cluster Survey.

| Country | Year | Early initiation of breastfeeding within 1 hour | | | | | | | | | | | | National prevalence (%) | Exclusive breastfeeding under 6 months | | | | | | | | | | | | National prevalence (%) |
| --- | --- | --- | --- | --- | --- | --- | --- | --- | --- | --- | --- | --- | --- | --- | --- | --- | --- | --- | --- | --- | --- | --- | --- | --- | --- | --- | --- |
|  |  | None | | | | Primary | | | | Secondary or higher | | | |  | None | | | | Primary | | | | Secondary or higher | | | |  |
|  |  | % | 95% CI | | N | % | 95% CI | | N | % | 95% CI | | N |  | % | 95% CI | | N | % | 95% CI | | N | % | 95% CI | | N |  |
| Afghanistan | 2010 | 53.0 | 49.6 | 56.3 | 4288 | 63.2 | 57.3 | 68.7 | 332 | 53.3 | 47.3 | 59.2 | 342 | 53.6 | 55.4 | 51.5 | 59.3 | 1075 | 58.8 | 46.1 | 70.5 | 89 | 36.4 | 27.0 | 47.0 | 106 | 54.3 |
| Afghanistan | 2015 | 40.2 | 37.4 | 43.1 | 9900 | 45.2 | 38.9 | 51.7 | 851 | 42.7 | 36.7 | 48.9 | 1011 | 40.9 | 44.3 | 41.2 | 47.3 | 2681 | 43.4 | 30.2 | 57.6 | 227 | 36.0 | 27.2 | 46.0 | 295 | 43.3 |
| Albania | 2005 | 22.3 | 2.4 | 77.1 | 3 | 34.0 | 7.9 | 75.7 | 6 | 29.9 | 24.5 | 36.0 | 386 | 29.9 | . | . | . | . | . | . | . | . | 3.4 | 1.1 | 10.2 | 98 | 3.4 |
| Albania | 2008 | 61.7 | 20.0 | 91.2 | 8 | 42.7 | 36.0 | 49.6 | 319 | 44.1 | 36.3 | 52.2 | 206 | 43.4 | 15.1 | 0.9 | 78.4 | 2 | 41.1 | 27.2 | 56.6 | 86 | 34.7 | 23.7 | 47.7 | 50 | 38.6 |
| Albania | 2017 | 76.1 | 34.9 | 95.0 | 7 | 52.8 | 46.6 | 58.9 | 545 | 59.1 | 53.2 | 64.7 | 504 | 56.5 | 32.7 | 2.9 | 88.7 | 2 | 35.3 | 26.3 | 45.5 | 139 | 37.8 | 28.0 | 48.7 | 144 | 36.7 |
| Armenia | 2000 | . | . | . | . | 0.0 | - | - | 1 | 24.5 | 20.8 | 28.7 | 602 | 24.5 | . | . | . | . | 0.0 | - | - | 1 | 30.0 | 22.7 | 38.6 | 154 | 29.9 |
| Armenia | 2005 | . | . | . | . | 0.0 | - | - | 2 | 32.6 | 27.9 | 37.7 | 557 | 32.2 | . | . | . | . | 100.0 | - | - | 1 | 32.0 | 22.6 | 43.1 | 156 | 32.5 |
| Armenia | 2010 | . | . | . | . | . | . | . | . | 35.7 | 31.6 | 40.0 | 628 | 35.7 | . | . | . | . | . | . | . | . | 34.6 | 26.2 | 44.0 | 156 | 34.6 |
| Armenia | 2015 | . | . | . | . | 57.3 | 7.6 | 95.6 | 2 | 40.8 | 36.5 | 45.3 | 680 | 40.9 | . | . | . | . | 100.0 | - | - | 1 | 44.3 | 36.4 | 52.5 | 176 | 44.5 |
| Bangladesh | 1993 | 7.5 | 6.0 | 9.4 | 1372 | 8.0 | 6.2 | 10.3 | 685 | 12.9 | 9.5 | 17.2 | 409 | 8.5 | 50.8 | 44.8 | 56.8 | 319 | 41.3 | 34.4 | 48.6 | 197 | 39.0 | 29.5 | 49.5 | 100 | 46.1 |
| Bangladesh | 1996 | 17.0 | 14.6 | 19.7 | 1289 | 20.8 | 17.3 | 24.8 | 652 | 23.3 | 19.1 | 28.2 | 400 | 19.1 | 49.8 | 43.9 | 55.6 | 321 | 37.2 | 28.5 | 46.7 | 170 | 42.8 | 33.5 | 52.7 | 121 | 45.0 |
| Bangladesh | 1999 | 12.2 | 10.0 | 14.8 | 1154 | 17.1 | 14.3 | 20.4 | 776 | 24.4 | 21.5 | 27.7 | 741 | 16.9 | 46.8 | 41.0 | 52.6 | 290 | 47.0 | 40.3 | 53.8 | 227 | 45.6 | 38.6 | 52.7 | 229 | 46.5 |
| Bangladesh | 2004 | 21.1 | 18.3 | 24.3 | 831 | 22.8 | 19.8 | 26.0 | 805 | 30.5 | 26.8 | 34.4 | 950 | 24.9 | 46.6 | 38.9 | 54.5 | 186 | 40.4 | 33.9 | 47.1 | 230 | 38.6 | 32.4 | 45.2 | 263 | 41.6 |
| Bangladesh | 2006 | 32.8 | 30.9 | 34.9 | 3780 | 36.1 | 34.1 | 38.2 | 3513 | 37.4 | 35.6 | 39.1 | 4614 | 35.6 | 37.2 | 33.1 | 41.5 | 710 | 37.4 | 33.2 | 41.8 | 659 | 37.7 | 34.0 | 41.6 | 924 | 37.4 |
| Bangladesh | 2007 | 42.4 | 36.9 | 48.1 | 528 | 44.4 | 40.2 | 48.7 | 677 | 42.5 | 38.8 | 46.3 | 1093 | 43.1 | 35.6 | 26.3 | 46.2 | 108 | 46.7 | 38.3 | 55.3 | 152 | 43.9 | 36.7 | 51.3 | 246 | 42.9 |
| Bangladesh | 2011 | 45.7 | 40.6 | 51.0 | 521 | 48.1 | 44.1 | 52.2 | 950 | 45.1 | 42.5 | 47.8 | 1779 | 46.1 | 56.5 | 46.4 | 66.1 | 126 | 62.4 | 54.7 | 69.5 | 217 | 67.3 | 61.9 | 72.3 | 446 | 64.1 |
| Bangladesh | 2012 | 67.2 | 64.2 | 70.1 | 1628 | 58.3 | 55.4 | 61.1 | 2317 | 53.5 | 51.3 | 55.7 | 3921 | 57.4 | 59.3 | 53.3 | 65.0 | 393 | 54.0 | 48.8 | 59.1 | 582 | 56.7 | 52.7 | 60.7 | 984 | 56.4 |
| Bangladesh | 2014 | 55.6 | 48.7 | 62.2 | 403 | 54.3 | 48.3 | 60.2 | 844 | 47.9 | 45.2 | 50.7 | 1831 | 50.8 | 56.2 | 44.8 | 67.0 | 84 | 53.7 | 42.7 | 64.3 | 165 | 56.0 | 48.5 | 63.1 | 383 | 55.3 |
| Bangladesh | 2019 | 56.0 | 52.2 | 59.7 | 846 | 54.0 | 51.6 | 56.3 | 2151 | 42.7 | 41.4 | 44.2 | 6288 | 46.6 | 55.0 | 46.7 | 63.0 | 179 | 63.3 | 58.8 | 67.7 | 585 | 64.1 | 61.4 | 66.7 | 1606 | 63.3 |
| Belize | 2006 | 66.1 | 40.2 | 84.9 | 16 | 55.7 | 48.0 | 63.2 | 179 | 40.5 | 30.6 | 51.3 | 117 | 50.4 | 36.4 | 3.2 | 90.9 | 2 | 10.9 | 4.4 | 24.7 | 54 | 7.9 | 2.0 | 26.8 | 29 | 10.1 |
| Belize | 2011 | 54.4 | 39.4 | 68.7 | 40 | 61.4 | 55.8 | 66.7 | 335 | 62.0 | 55.4 | 68.2 | 305 | 61.5 | 19.9 | 4.1 | 59.0 | 7 | 15.3 | 8.4 | 26.2 | 63 | 11.6 | 5.5 | 22.8 | 70 | 14.7 |
| Belize | 2015 | 76.6 | 61.4 | 87.0 | 23 | 67.5 | 61.8 | 72.7 | 413 | 68.4 | 62.3 | 73.9 | 472 | 68.3 | 49.0 | 5.5 | 94.1 | 2 | 34.7 | 23.0 | 48.6 | 75 | 32.1 | 20.4 | 46.6 | 86 | 33.2 |
| Benin | 1996 | 23.8 | 20.6 | 27.4 | 1608 | 21.6 | 16.9 | 27.3 | 308 | 34.1 | 23.1 | 47.1 | 83 | 24.0 | 10.1 | 7.0 | 14.3 | 423 | 9.3 | 4.8 | 17.0 | 74 | 13.4 | 4.2 | 35.5 | 21 | 10.1 |
| Benin | 2001 | 46.6 | 43.5 | 49.8 | 1521 | 50.5 | 45.3 | 55.7 | 404 | 51.6 | 43.5 | 59.6 | 167 | 47.8 | 38.1 | 32.8 | 43.6 | 384 | 39.7 | 30.7 | 49.5 | 99 | 33.1 | 20.2 | 49.1 | 44 | 37.9 |
| Benin | 2006 | 53.3 | 50.9 | 55.6 | 4742 | 55.2 | 52.0 | 58.4 | 1187 | 55.2 | 49.5 | 60.7 | 503 | 53.8 | 45.0 | 41.5 | 48.5 | 1127 | 38.7 | 32.9 | 44.7 | 274 | 36.3 | 28.5 | 45.0 | 124 | 43.1 |
| Benin | 2011 | 50.5 | 48.4 | 52.5 | 3719 | 51.2 | 47.5 | 54.9 | 827 | 49.4 | 44.9 | 53.9 | 632 | 50.4 | 33.0 | 29.6 | 36.5 | 854 | 31.7 | 25.4 | 38.7 | 198 | 35.7 | 28.6 | 43.5 | 143 | 33.1 |
| Benin | 2014 | 48.8 | 45.8 | 51.7 | 2923 | 43.5 | 39.2 | 47.9 | 1173 | 43.7 | 39.2 | 48.4 | 647 | 46.6 | 44.1 | 39.9 | 48.5 | 717 | 35.3 | 28.8 | 42.3 | 320 | 39.2 | 30.6 | 48.6 | 180 | 41.4 |
| Benin | 2017 | 57.3 | 55.1 | 59.5 | 3440 | 49.6 | 45.7 | 53.5 | 1024 | 47.7 | 44.0 | 51.3 | 1022 | 54.1 | 41.3 | 37.9 | 44.7 | 854 | 40.0 | 33.9 | 46.5 | 260 | 43.8 | 37.7 | 50.1 | 267 | 41.5 |
| Bolivia | 1994 | 22.4 | 18.5 | 26.7 | 336 | 21.0 | 18.5 | 23.8 | 1110 | 24.8 | 21.7 | 28.2 | 882 | 22.7 | 55.5 | 43.6 | 66.8 | 73 | 46.5 | 39.3 | 53.9 | 268 | 34.7 | 27.8 | 42.4 | 215 | 43.3 |
| Bolivia | 1998 | 29.3 | 24.1 | 35.0 | 363 | 36.4 | 33.3 | 39.6 | 1336 | 39.9 | 36.7 | 43.1 | 1058 | 37.0 | 59.2 | 46.5 | 70.8 | 79 | 59.0 | 52.0 | 65.6 | 289 | 40.7 | 34.3 | 47.5 | 261 | 50.6 |
| Bolivia | 2003 | 53.0 | 45.7 | 60.2 | 296 | 59.0 | 55.9 | 62.1 | 2149 | 57.2 | 53.8 | 60.5 | 1293 | 57.9 | 64.6 | 50.8 | 76.4 | 74 | 60.9 | 55.4 | 66.2 | 476 | 38.9 | 31.8 | 46.4 | 317 | 53.6 |
| Bolivia | 2008 | 72.1 | 63.6 | 79.3 | 168 | 65.5 | 62.6 | 68.3 | 1719 | 58.5 | 55.2 | 61.8 | 1496 | 62.8 | 69.7 | 49.1 | 84.6 | 42 | 68.2 | 62.3 | 73.6 | 380 | 50.9 | 44.9 | 57.0 | 369 | 60.4 |
| Bosnia and Herzegovina | 2006 | 60.0 | 19.9 | 90.1 | 5 | 52.2 | 43.5 | 60.8 | 339 | 58.6 | 52.5 | 64.4 | 829 | 56.7 | 0.0 | - | - | 1 | 18.2 | 8.1 | 36.0 | 62 | 17.4 | 8.8 | 31.5 | 123 | 17.6 |
| Bosnia and Herzegovina | 2011 | 0.0 | - | - | 2 | 53.3 | 40.9 | 65.3 | 155 | 39.2 | 32.9 | 45.9 | 561 | 42.3 | 0.0 | - | - | 1 | 30.7 | 10.1 | 63.7 | 23 | 15.7 | 9.1 | 25.8 | 93 | 18.5 |
| Burkina Faso | 1998 | 25.1 | 22.4 | 28.0 | 2026 | 24.0 | 18.7 | 30.2 | 171 | 32.6 | 24.3 | 42.2 | 100 | 25.2 | 5.1 | 3.2 | 7.9 | 548 | 7.1 | 2.5 | 18.6 | 41 | 15.4 | 6.4 | 32.5 | 29 | 5.5 |
| Burkina Faso | 2003 | 31.1 | 28.7 | 33.7 | 3572 | 36.0 | 29.5 | 42.9 | 380 | 55.6 | 44.4 | 66.3 | 169 | 32.4 | 18.3 | 15.2 | 21.9 | 911 | 20.6 | 12.5 | 32.0 | 110 | 26.4 | 14.2 | 43.8 | 50 | 18.8 |
| Burkina Faso | 2006 | 19.3 | 16.3 | 22.8 | 2090 | 21.1 | 15.1 | 28.7 | 214 | 20.5 | 10.2 | 36.9 | 80 | 19.6 | 6.8 | 4.3 | 10.6 | 483 | 7.9 | 2.4 | 22.9 | 42 | 4.1 | 0.5 | 25.6 | 18 | 6.8 |
| Burkina Faso | 2010 | 41.7 | 39.5 | 43.8 | 4827 | 40.6 | 36.3 | 45.1 | 670 | 52.4 | 46.0 | 58.7 | 362 | 42.1 | 23.3 | 20.4 | 26.6 | 1207 | 29.6 | 22.6 | 37.6 | 162 | 37.7 | 26.8 | 50.0 | 85 | 24.8 |
| Burundi | 2010 | 71.7 | 69.2 | 74.2 | 1447 | 75.8 | 73.0 | 78.4 | 1273 | 74.0 | 67.7 | 79.5 | 310 | 73.6 | 67.9 | 61.0 | 74.0 | 307 | 72.6 | 66.3 | 78.1 | 301 | 58.8 | 45.8 | 70.7 | 77 | 69.3 |
| Burundi | 2016 | 86.2 | 84.4 | 87.8 | 2198 | 85.8 | 84.0 | 87.4 | 2281 | 78.1 | 74.5 | 81.3 | 782 | 85.0 | 86.4 | 82.6 | 89.6 | 496 | 82.0 | 78.3 | 85.2 | 524 | 76.1 | 68.5 | 82.4 | 216 | 83.1 |
| CAR^1^ | 1994 | 32.1 | 28.8 | 35.5 | 902 | 35.8 | 32.1 | 39.6 | 682 | 39.7 | 32.6 | 47.4 | 237 | 34.5 | 3.1 | 1.3 | 6.9 | 221 | 3.2 | 1.4 | 7.2 | 189 | 2.1 | 0.3 | 13.6 | 48 | 3.0 |
| CAR^1^ | 2006 | 37.8 | 34.0 | 41.8 | 1770 | 39.1 | 34.4 | 44.1 | 1838 | 43.1 | 37.4 | 48.9 | 507 | 39.1 | 24.8 | 17.8 | 33.6 | 585 | 21.2 | 17.2 | 25.8 | 553 | 25.5 | 17.1 | 36.1 | 125 | 23.3 |
| CAR^1^ | 2010 | 45.8 | 42.3 | 49.3 | 1940 | 42.0 | 38.7 | 45.2 | 2098 | 41.8 | 35.6 | 48.2 | 507 | 43.5 | 32.0 | 26.9 | 37.6 | 556 | 34.6 | 29.8 | 39.8 | 587 | 35.6 | 25.6 | 46.9 | 140 | 33.7 |
| Cambodia | 2000 | 11.7 | 9.7 | 14.0 | 1186 | 9.9 | 8.4 | 11.6 | 1627 | 14.0 | 10.7 | 18.0 | 397 | 11.1 | 12.8 | 9.1 | 17.8 | 326 | 12.2 | 8.5 | 17.1 | 439 | 5.7 | 2.5 | 12.5 | 108 | 11.4 |
| Cambodia | 2005 | 32.6 | 28.3 | 37.1 | 901 | 34.7 | 31.9 | 37.5 | 1866 | 42.2 | 37.6 | 47.0 | 501 | 35.5 | 53.8 | 44.1 | 63.2 | 217 | 61.4 | 56.1 | 66.5 | 451 | 63.4 | 53.1 | 72.6 | 120 | 60.0 |
| Cambodia | 2010 | 63.8 | 58.1 | 69.1 | 650 | 66.8 | 63.5 | 70.0 | 1671 | 65.0 | 60.5 | 69.2 | 894 | 65.8 | 70.9 | 62.1 | 78.4 | 153 | 75.2 | 69.5 | 80.2 | 367 | 71.8 | 63.0 | 79.2 | 197 | 73.5 |
| Cambodia | 2014 | 58.4 | 51.5 | 65.0 | 369 | 66.6 | 63.0 | 70.0 | 1375 | 58.4 | 54.5 | 62.1 | 1155 | 62.6 | 63.8 | 49.0 | 76.3 | 81 | 68.7 | 61.6 | 75.0 | 313 | 61.3 | 54.4 | 67.7 | 294 | 65.2 |
| Cameroon | 1998 | 28.5 | 22.3 | 35.7 | 458 | 41.6 | 37.2 | 46.0 | 587 | 40.2 | 34.9 | 45.8 | 469 | 36.9 | 8.2 | 4.3 | 14.8 | 135 | 15.5 | 10.3 | 22.8 | 139 | 13.1 | 7.2 | 22.6 | 115 | 12.2 |
| Cameroon | 2004 | 26.0 | 22.0 | 30.5 | 791 | 30.4 | 28.1 | 32.7 | 1425 | 31.8 | 28.7 | 35.0 | 968 | 29.5 | 16.2 | 11.6 | 22.3 | 196 | 24.5 | 19.2 | 30.8 | 339 | 28.6 | 23.0 | 35.0 | 259 | 23.5 |
| Cameroon | 2006 | 16.4 | 13.2 | 20.1 | 782 | 18.6 | 16.0 | 21.5 | 1231 | 24.3 | 20.5 | 28.5 | 864 | 19.6 | 12.6 | 7.6 | 20.3 | 184 | 25.0 | 18.8 | 32.4 | 283 | 25.0 | 18.8 | 32.3 | 191 | 21.2 |
| Cameroon | 2011 | 33.4 | 29.0 | 38.1 | 1103 | 40.5 | 37.5 | 43.6 | 1915 | 44.1 | 41.2 | 47.0 | 1684 | 39.8 | 7.7 | 5.0 | 11.7 | 276 | 22.9 | 18.8 | 27.7 | 460 | 27.6 | 22.3 | 33.5 | 387 | 20.2 |
| Cameroon | 2014 | 23.8 | 19.9 | 28.2 | 682 | 33.1 | 29.6 | 36.8 | 1060 | 35.2 | 31.9 | 38.8 | 1157 | 31.2 | 13.1 | 8.8 | 19.1 | 190 | 33.8 | 26.7 | 41.7 | 225 | 36.8 | 30.5 | 43.6 | 288 | 28.2 |
| Cameroon | 2018 | 50.3 | 45.4 | 55.1 | 850 | 47.8 | 44.2 | 51.4 | 1201 | 47.6 | 44.2 | 51.1 | 1737 | 48.4 | 31.6 | 25.0 | 39.0 | 220 | 42.8 | 36.9 | 48.9 | 310 | 42.6 | 36.8 | 48.7 | 456 | 39.7 |
| Chad | 1996 | 24.3 | 21.6 | 27.2 | 2077 | 16.6 | 13.8 | 19.9 | 634 | 23.9 | 17.0 | 32.4 | 140 | 22.7 | 1.4 | 0.7 | 2.6 | 600 | 2.9 | 1.2 | 6.8 | 164 | 8.5 | 2.7 | 23.9 | 43 | 1.9 |
| Chad | 2004 | 34.9 | 31.4 | 38.6 | 1541 | 25.4 | 20.3 | 31.2 | 416 | 25.3 | 17.7 | 34.9 | 159 | 32.4 | 1.7 | 0.7 | 4.3 | 456 | 3.7 | 1.1 | 11.7 | 94 | 0.0 | - | - | 33 | 2.0 |
| Chad | 2010 | 31.2 | 28.5 | 34.1 | 4340 | 22.4 | 18.9 | 26.3 | 1229 | 23.9 | 18.5 | 30.2 | 584 | 28.7 | 3.0 | 2.0 | 4.5 | 1339 | 3.4 | 1.7 | 6.4 | 363 | 6.5 | 2.9 | 14.0 | 165 | 3.4 |
| Chad | 2014 | 25.8 | 23.1 | 28.8 | 4506 | 18.1 | 15.5 | 21.0 | 1403 | 19.2 | 16.0 | 22.9 | 681 | 23.0 | 0.1 | 0.0 | 0.6 | 1253 | 0.4 | 0.1 | 1.5 | 376 | 0.9 | 0.2 | 3.6 | 194 | 0.3 |
| Colombia | 1995 | 54.3 | 43.8 | 64.4 | 88 | 49.2 | 45.8 | 52.7 | 830 | 47.0 | 43.8 | 50.3 | 1033 | 48.3 | 4.9 | 0.7 | 28.5 | 25 | 9.0 | 5.4 | 14.6 | 168 | 13.8 | 9.8 | 19.1 | 247 | 11.5 |
| Colombia | 2000 | 52.9 | 39.5 | 65.9 | 58 | 65.8 | 61.9 | 69.5 | 725 | 58.9 | 55.7 | 62.0 | 1029 | 61.4 | 24.5 | 7.0 | 58.2 | 12 | 28.9 | 22.0 | 36.9 | 169 | 23.9 | 18.7 | 30.1 | 249 | 25.8 |
| Colombia | 2005 | 55.3 | 46.4 | 63.8 | 220 | 64.5 | 61.5 | 67.4 | 1824 | 56.8 | 54.5 | 59.1 | 3644 | 59.2 | 61.3 | 45.3 | 75.2 | 61 | 45.4 | 39.4 | 51.6 | 397 | 46.8 | 42.2 | 51.6 | 939 | 47.0 |
| Colombia | 2010 | 64.0 | 54.7 | 72.3 | 183 | 68.5 | 65.5 | 71.2 | 1861 | 61.9 | 60.1 | 63.6 | 4656 | 63.4 | 46.1 | 26.8 | 66.6 | 45 | 39.5 | 32.8 | 46.6 | 399 | 43.8 | 40.0 | 47.6 | 1071 | 42.9 |
| Comoros | 1996 | 21.8 | 17.9 | 26.4 | 472 | 21.3 | 14.9 | 29.4 | 174 | 32.4 | 24.8 | 41.0 | 105 | 23.2 | 3.4 | 1.2 | 8.8 | 119 | 2.2 | 0.3 | 13.7 | 46 | 4.8 | 0.7 | 26.8 | 21 | 3.2 |
| Comoros | 2012 | 34.9 | 29.3 | 40.9 | 512 | 30.4 | 23.7 | 38.0 | 311 | 34.8 | 29.3 | 40.7 | 427 | 33.7 | 11.2 | 6.4 | 18.8 | 144 | 15.5 | 7.3 | 30.2 | 72 | 11.3 | 5.7 | 21.3 | 109 | 12.1 |
| CDR^2^ | 2007 | 45.5 | 36.7 | 54.7 | 841 | 50.8 | 45.7 | 55.9 | 1527 | 46.1 | 41.2 | 51.1 | 1147 | 48.0 | 41.9 | 32.9 | 51.5 | 225 | 31.7 | 25.1 | 39.2 | 401 | 37.2 | 30.0 | 45.1 | 281 | 36.1 |
| CDR^2^ | 2010 | 45.0 | 38.8 | 51.3 | 1067 | 41.3 | 36.8 | 46.0 | 2009 | 42.2 | 38.8 | 45.6 | 1733 | 42.5 | 29.2 | 23.5 | 35.6 | 305 | 41.4 | 36.4 | 46.6 | 510 | 37.4 | 32.2 | 42.8 | 456 | 37.0 |
| CDR^2^ | 2013 | 55.5 | 50.7 | 60.2 | 1471 | 53.1 | 49.7 | 56.4 | 3227 | 49.1 | 45.7 | 52.5 | 2624 | 51.9 | 53.7 | 46.6 | 60.7 | 407 | 47.2 | 42.2 | 52.3 | 830 | 45.5 | 40.6 | 50.5 | 697 | 47.6 |
| CDR^2^ | 2017 | 50.7 | 44.0 | 57.4 | 1873 | 48.5 | 44.6 | 52.4 | 3408 | 44.5 | 40.4 | 48.6 | 3279 | 46.9 | 50.1 | 42.0 | 58.2 | 481 | 57.1 | 51.1 | 62.9 | 809 | 54.7 | 48.2 | 61.0 | 816 | 54.6 |
| Cote d'Ivoire | 1994 | 44.7 | 41.0 | 48.3 | 1686 | 38.7 | 34.2 | 43.3 | 636 | 39.1 | 32.3 | 46.3 | 224 | 42.7 | 3.1 | 1.9 | 5.3 | 446 | 3.7 | 1.7 | 7.9 | 158 | 0.0 | - | - | 54 | 3.0 |
| Cote d'Ivoire | 1998 | 31.8 | 27.4 | 36.6 | 498 | 22.5 | 16.9 | 29.3 | 231 | 22.1 | 13.9 | 33.2 | 82 | 28.6 | 3.7 | 1.1 | 12.0 | 102 | 3.7 | 0.5 | 23.3 | 50 | 0.0 | - | - | 14 | 3.5 |
| Cote d'Ivoire | 2006 | 25.0 | 21.6 | 28.7 | 2304 | 24.9 | 20.3 | 30.1 | 1013 | 24.7 | 17.9 | 32.9 | 365 | 24.9 | 6.2 | 3.6 | 10.5 | 582 | 1.7 | 0.5 | 5.7 | 261 | 0.9 | 0.1 | 6.3 | 97 | 4.3 |
| Cote d'Ivoire | 2011 | 31.3 | 27.6 | 35.2 | 2037 | 30.0 | 25.3 | 35.1 | 751 | 30.2 | 24.2 | 37.0 | 325 | 30.8 | 10.7 | 7.7 | 14.6 | 531 | 13.0 | 8.1 | 20.2 | 172 | 20.1 | 11.2 | 33.5 | 72 | 12.2 |
| Cote d'Ivoire | 2016 | 39.7 | 36.4 | 43.1 | 2464 | 33.4 | 28.9 | 38.2 | 819 | 30.3 | 25.0 | 36.1 | 466 | 36.6 | 23.0 | 19.3 | 27.2 | 677 | 25.4 | 18.7 | 33.6 | 193 | 22.8 | 14.6 | 33.8 | 111 | 23.5 |
| Dominican Republic | 1996 | 65.5 | 57.0 | 73.1 | 198 | 61.9 | 58.1 | 65.6 | 948 | 52.0 | 46.6 | 57.3 | 559 | 58.5 | 12.4 | 5.0 | 27.6 | 43 | 21.2 | 15.3 | 28.4 | 238 | 14.1 | 8.2 | 23.0 | 134 | 18.0 |
| Dominican Republic | 1999 | 46.0 | . | . | 5 | 64.7 | . | . | 110 | 45.3 | . | . | 82 | 55.6 | 0.0 | - | - | 2 | 17.8 | . | . | 27 | 12.4 | . | . | 23 | 15.1 |
| Dominican Republic | 2002 | 76.7 | 68.7 | 83.2 | 248 | 66.2 | 63.1 | 69.1 | 2269 | 56.7 | 53.6 | 59.7 | 1808 | 62.0 | 9.7 | 3.5 | 24.1 | 58 | 10.8 | 7.2 | 15.8 | 525 | 10.1 | 6.7 | 14.9 | 420 | 10.4 |
| Dominican Republic | 2007 | 72.2 | 63.0 | 79.8 | 242 | 66.4 | 63.0 | 69.7 | 1738 | 55.9 | 52.5 | 59.1 | 2145 | 60.4 | 13.6 | 5.7 | 28.9 | 58 | 7.3 | 4.8 | 11.0 | 407 | 7.6 | 4.9 | 11.7 | 521 | 7.8 |
| Dominican Republic | 2013 | 66.8 | 49.2 | 80.7 | 50 | 46.9 | 39.4 | 54.5 | 455 | 40.6 | 35.9 | 45.5 | 925 | 43.2 | 33.2 | 7.1 | 76.3 | 12 | 6.4 | 2.5 | 15.5 | 84 | 5.8 | 2.7 | 11.9 | 211 | 6.7 |
| Dominican Republic | 2014 | 58.2 | 48.9 | 66.9 | 252 | 47.1 | 43.7 | 50.6 | 2162 | 33.8 | 31.6 | 36.0 | 5152 | 38.1 | 14.3 | 6.5 | 28.6 | 71 | 6.9 | 3.6 | 12.9 | 460 | 3.5 | 2.5 | 5.0 | 1123 | 4.7 |
| Egypt | 1995 | 39.2 | 36.4 | 42.0 | 2087 | 38.6 | 34.7 | 42.7 | 872 | 39.1 | 36.1 | 42.1 | 1540 | 39.0 | 63.1 | 58.2 | 67.7 | 525 | 59.6 | 50.9 | 67.8 | 208 | 46.8 | 40.9 | 52.9 | 405 | 56.3 |
| Egypt | 2000 | 60.2 | 57.3 | 62.9 | 1667 | 53.6 | 49.5 | 57.6 | 643 | 49.0 | 46.5 | 51.5 | 2151 | 53.9 | 59.9 | 55.0 | 64.6 | 465 | 57.1 | 49.9 | 64.0 | 159 | 52.9 | 48.4 | 57.4 | 577 | 56.2 |
| Egypt | 2005 | 46.9 | 41.9 | 52.0 | 1671 | 41.4 | 35.5 | 47.6 | 603 | 36.3 | 33.0 | 39.8 | 3009 | 40.1 | 38.5 | 28.5 | 49.5 | 403 | 45.7 | 31.0 | 61.1 | 144 | 37.8 | 31.0 | 45.1 | 703 | 39.0 |
| Egypt | 2008 | 58.6 | 55.2 | 61.9 | 1140 | 58.7 | 53.2 | 63.9 | 448 | 51.4 | 49.4 | 53.5 | 2947 | 53.8 | 55.6 | 49.6 | 61.4 | 300 | 54.1 | 42.7 | 65.1 | 97 | 52.2 | 48.1 | 56.2 | 733 | 53.2 |
| Egypt | 2014 | 30.7 | 27.1 | 34.6 | 985 | 29.3 | 25.0 | 34.1 | 519 | 26.1 | 24.4 | 27.9 | 4906 | 27.1 | 42.7 | 34.9 | 50.9 | 254 | 34.0 | 24.7 | 44.7 | 122 | 39.6 | 36.2 | 43.1 | 1111 | 39.7 |
| Eswatini | 2006 | 64.4 | 53.7 | 73.8 | 98 | 60.9 | 55.4 | 66.1 | 415 | 55.3 | 51.3 | 59.3 | 639 | 58.1 | 43.3 | 25.9 | 62.5 | 31 | 34.6 | 23.7 | 47.4 | 82 | 28.7 | 21.9 | 36.7 | 145 | 32.3 |
| Eswatini | 2010 | 54.4 | 40.1 | 68.0 | 55 | 62.4 | 56.4 | 68.1 | 284 | 51.1 | 47.0 | 55.3 | 679 | 54.5 | 40.9 | 16.1 | 71.4 | 11 | 57.0 | 43.9 | 69.1 | 73 | 39.2 | 32.6 | 46.3 | 185 | 44.1 |
| Eswatini | 2014 | 44.3 | 28.2 | 61.7 | 43 | 49.2 | 42.4 | 56.1 | 256 | 48.2 | 43.4 | 53.0 | 688 | 48.3 | 42.8 | 15.2 | 75.8 | 10 | 68.3 | 55.0 | 79.1 | 54 | 63.7 | 53.2 | 73.1 | 170 | 63.8 |
| Ethiopia | 2011 | 50.7 | 46.9 | 54.4 | 2845 | 51.5 | 46.8 | 56.2 | 1184 | 64.1 | 54.9 | 72.3 | 255 | 51.5 | 53.7 | 47.4 | 59.9 | 788 | 49.1 | 41.5 | 56.7 | 330 | 47.9 | 28.9 | 67.6 | 69 | 52.0 |
| Ethiopia | 2016 | 73.4 | 70.6 | 76.1 | 2417 | 74.1 | 70.0 | 77.9 | 1138 | 69.8 | 62.9 | 75.9 | 526 | 73.3 | 57.3 | 50.9 | 63.6 | 638 | 58.6 | 50.7 | 66.1 | 306 | 54.6 | 42.5 | 66.2 | 148 | 57.5 |
| Gabon | 2000 | 60.2 | 49.0 | 70.4 | 97 | 62.6 | 58.1 | 66.9 | 860 | 58.9 | 55.0 | 62.8 | 819 | 60.5 | 2.8 | 0.4 | 18.2 | 23 | 7.8 | 4.4 | 13.4 | 237 | 3.5 | 1.3 | 8.9 | 191 | 5.4 |
| Gabon | 2012 | 34.4 | 22.1 | 49.1 | 142 | 32.5 | 27.5 | 37.9 | 978 | 32.1 | 27.8 | 36.7 | 1391 | 32.3 | 6.3 | 1.9 | 18.8 | 36 | 8.0 | 3.4 | 18.0 | 244 | 5.2 | 2.7 | 10.0 | 351 | 6.0 |
| Gambia | 2005 | 50.5 | 47.4 | 53.7 | 1973 | 43.3 | 37.8 | 48.9 | 344 | 45.4 | 40.6 | 50.3 | 475 | 47.7 | 37.6 | 33.2 | 42.2 | 550 | 43.6 | 33.3 | 54.4 | 87 | 43.4 | 35.2 | 52.0 | 140 | 40.8 |
| Gambia | 2010 | 51.3 | 48.5 | 54.1 | 4018 | 50.2 | 44.2 | 56.3 | 435 | 52.7 | 47.4 | 57.9 | 769 | 51.5 | 34.6 | 30.7 | 38.6 | 1073 | 23.8 | 15.9 | 34.1 | 126 | 34.9 | 28.8 | 41.5 | 213 | 33.5 |
| Gambia | 2013 | 55.1 | 50.1 | 60.0 | 2135 | 46.8 | 39.9 | 53.9 | 514 | 46.6 | 40.3 | 53.0 | 832 | 51.5 | 45.2 | 39.3 | 51.3 | 535 | 49.0 | 39.7 | 58.4 | 161 | 48.2 | 39.5 | 57.1 | 255 | 46.8 |
| Gambia | 2018 | 47.6 | 44.7 | 50.4 | 2087 | 44.7 | 39.6 | 49.8 | 706 | 45.9 | 41.8 | 50.0 | 1003 | 46.5 | 51.1 | 45.1 | 57.0 | 487 | 60.4 | 51.3 | 68.9 | 166 | 58.1 | 50.0 | 65.8 | 256 | 55.2 |
| Georgia | 2005 | . | . | . | . | 0.0 | - | - | 3 | 36.8 | 32.7 | 41.1 | 757 | 36.6 | . | . | . | . | . | . | . | . | 11.8 | 7.0 | 19.3 | 171 | 11.8 |
| Georgia | 2018 | . | . | . | . | 32.7 | 21.5 | 46.3 | 107 | 32.9 | 28.4 | 37.7 | 801 | 32.8 | 0.0 | - | - | 1 | 17.9 | 6.9 | 39.3 | 30 | 21.6 | 15.7 | 29.0 | 203 | 21.2 |
| Ghana | 1993 | 14.9 | 12.2 | 18.0 | 578 | 16.1 | 13.7 | 18.7 | 766 | 15.1 | 9.0 | 24.2 | 86 | 15.5 | 4.4 | 2.0 | 9.2 | 159 | 5.6 | 2.9 | 10.5 | 195 | 16.0 | 6.3 | 35.1 | 25 | 5.8 |
| Ghana | 1998 | 21.6 | 17.7 | 26.1 | 596 | 25.5 | 19.9 | 32.0 | 245 | 28.5 | 24.2 | 33.2 | 477 | 25.3 | 24.2 | 17.1 | 33.0 | 142 | 25.4 | 15.5 | 38.8 | 56 | 41.8 | 32.9 | 51.2 | 104 | 31.5 |
| Ghana | 2003 | 47.6 | 43.1 | 52.1 | 719 | 39.2 | 33.2 | 45.6 | 316 | 46.9 | 42.3 | 51.6 | 484 | 45.4 | 52.8 | 43.7 | 61.8 | 177 | 49.5 | 37.0 | 62.1 | 64 | 56.6 | 47.3 | 65.4 | 97 | 53.4 |
| Ghana | 2006 | 35.9 | 30.9 | 41.2 | 674 | 32.5 | 26.7 | 38.9 | 287 | 35.9 | 30.9 | 41.3 | 498 | 35.2 | 61.3 | 51.3 | 70.4 | 165 | 53.3 | 41.7 | 64.5 | 72 | 51.6 | 42.3 | 60.8 | 147 | 55.3 |
| Ghana | 2008 | 52.0 | 45.3 | 58.6 | 433 | 52.0 | 45.6 | 58.4 | 298 | 51.9 | 47.2 | 56.5 | 494 | 51.9 | 68.0 | 57.0 | 77.3 | 104 | 60.2 | 48.0 | 71.2 | 82 | 60.8 | 51.9 | 69.1 | 132 | 62.8 |
| Ghana | 2011 | 44.9 | 39.7 | 50.2 | 1442 | 45.8 | 39.6 | 52.3 | 562 | 46.5 | 41.6 | 51.4 | 869 | 45.9 | 55.0 | 45.7 | 63.9 | 382 | 43.1 | 32.7 | 54.2 | 150 | 41.4 | 34.0 | 49.3 | 260 | 45.7 |
| Ghana | 2014 | 58.8 | 53.1 | 64.4 | 783 | 52.8 | 46.3 | 59.2 | 469 | 55.0 | 51.5 | 58.5 | 1077 | 55.6 | 52.6 | 43.2 | 61.8 | 205 | 45.6 | 35.7 | 55.9 | 128 | 54.6 | 46.8 | 62.3 | 273 | 52.3 |
| Ghana | 2017 | 51.9 | 47.5 | 56.2 | 935 | 52.0 | 48.8 | 55.2 | 1910 | 51.9 | 45.6 | 58.2 | 621 | 52.0 | 46.0 | 37.1 | 55.2 | 233 | 39.5 | 33.5 | 45.9 | 482 | 49.1 | 38.9 | 59.3 | 176 | 42.9 |
| Guatemala | 1995 | 49.6 | 45.9 | 53.3 | 1682 | 53.8 | 50.6 | 57.0 | 1941 | 48.1 | 41.4 | 54.9 | 339 | 51.5 | 59.3 | 53.5 | 64.9 | 401 | 45.1 | 38.6 | 51.7 | 475 | 13.6 | 6.9 | 25.1 | 93 | 46.4 |
| Guatemala | 1998 | 48.9 | 42.2 | 55.7 | 704 | 46.5 | 41.7 | 51.3 | 990 | 48.2 | 39.3 | 57.3 | 178 | 47.5 | 58.2 | 46.7 | 68.8 | 176 | 35.7 | 28.7 | 43.4 | 249 | 16.2 | 3.8 | 48.4 | 51 | 39.2 |
| Guatemala | 2014 | 73.2 | 69.1 | 76.9 | 774 | 68.0 | 65.6 | 70.3 | 2487 | 50.3 | 46.9 | 53.7 | 1528 | 63.1 | 71.3 | 63.0 | 78.4 | 179 | 61.6 | 56.9 | 66.2 | 588 | 33.5 | 28.4 | 38.9 | 408 | 53.2 |
| Guinea | 1999 | 24.9 | 22.8 | 27.2 | 1841 | 24.6 | 19.3 | 30.9 | 197 | 26.1 | 19.3 | 34.4 | 140 | 25.0 | 11.3 | 8.4 | 15.1 | 573 | 14.0 | 7.3 | 25.3 | 58 | 6.3 | 1.6 | 22.0 | 31 | 11.3 |
| Guinea | 2005 | 37.9 | 35.0 | 41.0 | 2236 | 37.1 | 30.7 | 43.9 | 218 | 39.6 | 31.0 | 48.9 | 132 | 37.9 | 26.7 | 22.4 | 31.5 | 638 | 19.4 | 11.2 | 31.7 | 68 | 52.0 | 32.2 | 71.1 | 27 | 27.0 |
| Guinea | 2012 | 16.6 | 13.8 | 19.9 | 2133 | 15.7 | 11.6 | 20.9 | 373 | 17.2 | 12.4 | 23.4 | 304 | 16.6 | 20.5 | 15.6 | 26.6 | 548 | 17.9 | 11.3 | 27.3 | 92 | 23.3 | 15.2 | 34.1 | 78 | 20.5 |
| Guinea | 2016 | 34.1 | 31.3 | 36.9 | 1999 | 27.8 | 23.1 | 33.1 | 392 | 37.4 | 32.1 | 43.1 | 403 | 33.7 | 33.0 | 28.2 | 38.3 | 509 | 45.5 | 33.5 | 58.1 | 92 | 36.2 | 24.2 | 50.3 | 85 | 35.2 |
| Guinea | 2018 | 42.1 | 38.7 | 45.6 | 2275 | 39.5 | 34.1 | 45.2 | 379 | 49.5 | 43.8 | 55.1 | 412 | 42.8 | 33.5 | 29.4 | 38.0 | 706 | 30.5 | 21.6 | 41.2 | 103 | 35.5 | 27.2 | 44.8 | 107 | 33.4 |
| Guinea Bissau | 2006 | 22.1 | 19.6 | 25.0 | 1645 | 22.7 | 18.4 | 27.8 | 535 | 25.0 | 19.4 | 31.7 | 240 | 22.6 | 14.2 | 10.7 | 18.6 | 442 | 21.2 | 14.9 | 29.4 | 143 | 21.5 | 11.8 | 35.9 | 62 | 16.3 |
| Guinea Bissau | 2014 | 33.1 | 30.2 | 36.0 | 1775 | 34.0 | 30.7 | 37.4 | 1038 | 35.0 | 29.2 | 41.2 | 383 | 33.7 | 47.0 | 42.3 | 51.9 | 488 | 61.1 | 53.3 | 68.3 | 265 | 57.5 | 41.9 | 71.7 | 77 | 52.5 |
| Guyana | 2006 | 88.4 | 31.9 | 99.2 | 2 | 47.7 | 39.1 | 56.4 | 203 | 42.3 | 37.4 | 47.3 | 664 | 43.1 | . | . | . | . | 29.0 | 15.5 | 47.7 | 50 | 20.4 | 14.6 | 27.8 | 161 | 21.4 |
| Guyana | 2009 | 54.2 | 27.3 | 78.9 | 24 | 65.4 | 53.0 | 76.1 | 208 | 58.3 | 53.1 | 63.3 | 637 | 59.7 | 0.0 | - | - | 2 | 40.3 | 26.3 | 56.0 | 59 | 31.4 | 23.2 | 41.0 | 169 | 33.2 |
| Guyana | 2014 | 25.4 | 12.5 | 44.9 | 26 | 48.5 | 39.4 | 57.7 | 180 | 49.8 | 45.7 | 53.9 | 1052 | 49.2 | 57.3 | 10.7 | 93.8 | 3 | 29.2 | 13.3 | 52.6 | 43 | 22.0 | 16.6 | 28.6 | 244 | 23.3 |
| Haiti | 1994 | 33.1 | 26.0 | 41.2 | 632 | 35.0 | 29.8 | 40.6 | 565 | 36.3 | 29.3 | 43.8 | 178 | 34.3 | 2.2 | 0.7 | 6.6 | 139 | 2.4 | 0.8 | 7.3 | 134 | 4.2 | 1.0 | 15.5 | 44 | 2.5 |
| Haiti | 2000 | 45.7 | 38.8 | 52.8 | 981 | 48.7 | 44.3 | 53.1 | 1186 | 55.2 | 47.6 | 62.6 | 384 | 48.7 | 24.5 | 15.4 | 36.6 | 208 | 27.6 | 19.6 | 37.4 | 267 | 12.8 | 6.3 | 24.3 | 88 | 23.7 |
| Haiti | 2005 | 46.1 | 41.5 | 50.7 | 787 | 44.2 | 39.1 | 49.5 | 1082 | 37.1 | 31.6 | 43.0 | 574 | 42.9 | 40.2 | 31.3 | 49.8 | 171 | 41.9 | 33.2 | 51.1 | 274 | 38.9 | 30.2 | 48.4 | 153 | 40.7 |
| Haiti | 2012 | 45.3 | 39.2 | 51.5 | 571 | 48.1 | 44.1 | 52.1 | 1342 | 45.7 | 41.6 | 49.9 | 994 | 46.7 | 40.7 | 30.1 | 52.2 | 114 | 38.9 | 32.7 | 45.5 | 353 | 40.3 | 32.6 | 48.6 | 259 | 39.7 |
| Haiti | 2016 | 52.0 | 46.1 | 57.8 | 444 | 48.6 | 44.7 | 52.6 | 1057 | 44.3 | 40.9 | 47.8 | 1034 | 47.4 | 46.2 | 33.7 | 59.2 | 103 | 34.2 | 27.7 | 41.3 | 292 | 42.7 | 36.5 | 49.2 | 305 | 39.9 |
| Honduras | 2005 | 80.8 | 75.9 | 84.9 | 416 | 77.2 | 75.1 | 79.2 | 2944 | 68.8 | 65.1 | 72.3 | 793 | 75.6 | 44.2 | 33.0 | 56.1 | 82 | 32.0 | 28.0 | 36.2 | 706 | 16.7 | 11.7 | 23.4 | 185 | 29.7 |
| Honduras | 2011 | 75.7 | 67.6 | 82.2 | 228 | 68.6 | 66.3 | 70.7 | 2718 | 55.6 | 51.9 | 59.1 | 1503 | 63.8 | 47.7 | 31.5 | 64.4 | 45 | 35.9 | 32.1 | 40.0 | 685 | 22.3 | 17.8 | 27.5 | 354 | 31.2 |
| India | 2005 | 15.8 | 14.7 | 17.0 | 7411 | 23.4 | 21.5 | 25.5 | 2729 | 32.1 | 30.6 | 33.6 | 9134 | 23.1 | 48.1 | 45.1 | 51.2 | 1806 | 49.1 | 44.3 | 53.9 | 693 | 43.3 | 40.3 | 46.2 | 2117 | 46.4 |
| India | 2015 | 36.4 | 35.5 | 37.3 | 27630 | 40.4 | 39.1 | 41.8 | 13678 | 44.1 | 43.4 | 44.8 | 56627 | 41.5 | 53.9 | 52.2 | 55.5 | 6077 | 53.9 | 51.5 | 56.3 | 3137 | 55.6 | 54.3 | 56.8 | 13412 | 54.9 |
| Indonesia | 2002 | 43.1 | 32.4 | 54.5 | 247 | 36.8 | 33.7 | 40.0 | 2754 | 35.7 | 32.5 | 39.0 | 3294 | 36.5 | 51.9 | 27.0 | 75.8 | 64 | 40.1 | 33.9 | 46.6 | 711 | 37.9 | 32.5 | 43.7 | 866 | 39.5 |
| Indonesia | 2007 | 53.0 | 42.6 | 63.2 | 234 | 43.0 | 40.2 | 45.9 | 2723 | 37.5 | 35.2 | 39.8 | 4316 | 40.0 | 28.6 | 13.1 | 51.6 | 66 | 35.5 | 29.6 | 41.8 | 648 | 30.8 | 26.4 | 35.6 | 1088 | 32.4 |
| Indonesia | 2012 | 65.3 | 55.5 | 74.0 | 214 | 54.0 | 50.6 | 57.3 | 2025 | 46.8 | 44.6 | 49.1 | 4902 | 49.3 | 26.8 | 16.1 | 41.1 | 63 | 41.9 | 35.0 | 49.1 | 449 | 41.8 | 37.7 | 46.0 | 1174 | 41.5 |
| Indonesia | 2017 | 62.2 | 48.8 | 74.0 | 90 | 56.8 | 53.5 | 60.1 | 1551 | 56.4 | 54.5 | 58.2 | 5284 | 56.5 | 35.5 | 14.2 | 64.7 | 19 | 52.8 | 46.5 | 59.0 | 384 | 51.2 | 47.8 | 54.7 | 1263 | 51.5 |
| Iraq | 2006 | 34.5 | 30.9 | 38.3 | 1296 | 30.5 | 28.2 | 33.0 | 3155 | 28.3 | 25.6 | 31.2 | 2051 | 30.6 | 25.4 | 19.9 | 31.7 | 322 | 25.7 | 22.1 | 29.8 | 785 | 24.9 | 20.5 | 29.8 | 509 | 25.5 |
| Iraq | 2011 | 48.0 | 45.2 | 50.8 | 3331 | 42.4 | 40.4 | 44.4 | 6975 | 40.7 | 38.1 | 43.3 | 3688 | 42.8 | 22.9 | 19.0 | 27.4 | 977 | 20.6 | 18.0 | 23.5 | 1945 | 16.3 | 13.0 | 20.3 | 960 | 19.6 |
| Iraq | 2018 | 38.8 | 33.3 | 44.5 | 1285 | 33.0 | 29.7 | 36.5 | 2703 | 28.8 | 25.6 | 32.2 | 2262 | 32.4 | 32.7 | 25.9 | 40.3 | 333 | 28.0 | 23.9 | 32.6 | 715 | 22.8 | 19.0 | 27.1 | 633 | 26.7 |
| Jamaica | 2005 | . | . | . | . | 73.3 | 45.4 | 90.0 | 15 | 62.3 | 57.4 | 67.0 | 514 | 62.3 | . | . | . | . | 24.1 | 2.9 | 77.2 | 4 | 15.1 | 9.5 | 23.2 | 123 | 15.2 |
| Jamaica | 2011 | . | . | . | . | 77.3 | 45.5 | 93.3 | 11 | 64.5 | 59.2 | 69.5 | 619 | 64.7 | . | . | . | . | 29.5 | 4.1 | 80.5 | 4 | 23.7 | 17.4 | 31.3 | 163 | 23.8 |
| Jordan | 1997 | 31.6 | 23.8 | 40.5 | 176 | 34.4 | 29.0 | 40.3 | 265 | 28.8 | 26.4 | 31.2 | 1880 | 29.6 | 3.8 | 0.9 | 14.6 | 32 | 14.3 | 6.5 | 28.6 | 50 | 11.1 | 8.3 | 14.8 | 417 | 11.0 |
| Jordan | 2002 | 37.0 | 27.6 | 47.6 | 124 | 32.3 | 25.8 | 39.6 | 219 | 34.6 | 32.0 | 37.3 | 1937 | 34.5 | 30.7 | 11.5 | 60.2 | 30 | 16.5 | 6.9 | 34.3 | 44 | 27.4 | 22.8 | 32.5 | 433 | 26.7 |
| Jordan | 2007 | 38.9 | 29.5 | 49.3 | 174 | 51.4 | 42.6 | 60.2 | 265 | 36.3 | 33.8 | 38.8 | 3456 | 37.2 | 8.8 | 2.6 | 25.7 | 53 | 17.4 | 6.6 | 38.4 | 70 | 22.4 | 18.6 | 26.6 | 994 | 21.8 |
| Jordan | 2012 | 24.9 | 15.8 | 36.8 | 97 | 25.0 | 16.9 | 35.3 | 232 | 18.0 | 16.1 | 20.2 | 3349 | 18.6 | 46.5 | 21.2 | 73.7 | 17 | 15.0 | 6.6 | 30.5 | 45 | 22.8 | 18.0 | 28.5 | 788 | 22.7 |
| Jordan | 2017 | 80.4 | 67.1 | 89.1 | 89 | 61.7 | 52.0 | 70.6 | 287 | 67.2 | 64.4 | 69.8 | 3582 | 67.0 | 22.2 | 8.0 | 48.3 | 35 | 34.9 | 21.4 | 51.5 | 95 | 25.1 | 21.4 | 29.1 | 1088 | 25.5 |
| Kazakhstan | 1995 | . | . | . | . | 0.0 | - | - | 2 | 11.2 | 7.9 | 15.5 | 537 | 11.1 | . | . | . | . | . | . | . | . | 9.6 | 5.6 | 15.9 | 118 | 9.6 |
| Kazakhstan | 1999 | 39.2 | 3.7 | 91.5 | 2 | . | . | . | . | 28.5 | 23.6 | 34.1 | 468 | 28.6 | 39.2 | 3.1 | 92.9 | 2 | . | . | . | . | 35.7 | 26.0 | 46.6 | 97 | 35.8 |
| Kazakhstan | 2006 | 63.0 | 13.0 | 95.1 | 3 | 100.0 | - | - | 3 | 64.1 | 60.9 | 67.2 | 1778 | 64.2 | 0.0 | - | - | 1 | . | . | . | . | 16.8 | 13.2 | 21.3 | 386 | 16.8 |
| Kazakhstan | 2015 | 100.0 | - | - | 2 | . | . | . | . | 83.3 | 81.1 | 85.2 | 2104 | 83.3 | 100.0 | - | - | 1 | . | . | . | . | 37.8 | 32.0 | 43.9 | 506 | 37.8 |
| Kenya | 1993 | 56.0 | 50.2 | 61.6 | 374 | 52.2 | 49.2 | 55.2 | 1440 | 54.6 | 49.1 | 60.0 | 491 | 53.4 | 13.8 | 8.1 | 22.7 | 86 | 11.4 | 7.6 | 16.9 | 319 | 8.8 | 4.6 | 16.1 | 113 | 11.3 |
| Kenya | 1998 | 56.2 | 48.8 | 63.3 | 255 | 55.6 | 52.3 | 59.0 | 1496 | 58.5 | 53.5 | 63.4 | 549 | 56.4 | 8.9 | 3.0 | 23.8 | 44 | 14.0 | 10.3 | 18.8 | 330 | 10.6 | 5.6 | 19.1 | 144 | 12.6 |
| Kenya | 2003 | 54.0 | 44.9 | 62.8 | 450 | 48.0 | 45.1 | 50.9 | 1403 | 51.5 | 46.7 | 56.2 | 504 | 49.6 | 14.4 | 8.4 | 23.5 | 126 | 12.0 | 8.5 | 16.6 | 358 | 13.5 | 8.6 | 20.7 | 115 | 12.7 |
| Kenya | 2008 | 58.0 | 48.2 | 67.2 | 461 | 54.4 | 49.8 | 59.0 | 1360 | 58.1 | 53.0 | 63.0 | 552 | 55.8 | 24.6 | 15.0 | 37.4 | 125 | 36.4 | 29.6 | 43.6 | 311 | 25.1 | 17.7 | 34.3 | 151 | 31.9 |
| Kenya | 2014 | 76.0 | 71.3 | 80.2 | 824 | 61.4 | 58.6 | 64.2 | 1980 | 58.8 | 54.4 | 63.0 | 1017 | 62.2 | 54.8 | 46.9 | 62.5 | 193 | 59.8 | 53.6 | 65.8 | 455 | 66.6 | 57.3 | 74.7 | 208 | 61.4 |
| Kyrgyzstan | 1997 | 0.0 | - | - | 1 | 100.0 | - | - | 1 | 43.4 | 38.2 | 48.7 | 724 | 43.5 | . | . | . | . | 100.0 | - | - | 1 | 23.6 | 16.5 | 32.6 | 176 | 24.0 |
| Kyrgyzstan | 2005 | . | . | . | . | 100.0 | - | - | 1 | 64.9 | 59.4 | 70.1 | 1149 | 64.9 | . | . | . | . | . | . | . | . | 35.6 | 27.3 | 44.9 | 286 | 35.6 |
| Kyrgyzstan | 2012 | 100.0 | - | - | 1 | 76.8 | 25.7 | 96.9 | 5 | 83.8 | 81.6 | 85.9 | 1799 | 83.8 | . | . | . | . | 35.1 | 3.0 | 90.5 | 2 | 56.3 | 50.8 | 61.7 | 445 | 56.1 |
| Kyrgyzstan | 2014 | 90.9 | 38.1 | 99.4 | 6 | 93.3 | 57.8 | 99.3 | 4 | 82.4 | 80.2 | 84.4 | 1756 | 82.5 | 0.0 | - | - | 2 | . | . | . | . | 41.4 | 35.7 | 47.4 | 429 | 41.1 |
| Kyrgyzstan | 2018 | . | . | . | . | 62.1 | 9.2 | 96.4 | 2 | 81.0 | 77.8 | 83.9 | 1344 | 81.0 | . | . | . | . | . | . | . | . | 46.3 | 39.9 | 52.7 | 390 | 46.3 |
| Lao | 2006 | 24.4 | 19.6 | 29.9 | 618 | 25.4 | 21.5 | 29.8 | 727 | 51.3 | 44.4 | 58.1 | 260 | 29.8 | 29.7 | 21.3 | 39.7 | 171 | 23.6 | 17.3 | 31.2 | 186 | 26.7 | 16.7 | 39.9 | 86 | 26.7 |
| Lao | 2011 | 31.1 | 27.8 | 34.7 | 1363 | 37.8 | 35.0 | 40.7 | 1855 | 48.4 | 44.9 | 51.8 | 1226 | 39.1 | 45.1 | 38.8 | 51.6 | 369 | 35.0 | 30.1 | 40.2 | 454 | 42.8 | 37.1 | 48.8 | 345 | 40.4 |
| Lao | 2017 | 52.8 | 48.0 | 57.5 | 870 | 49.2 | 45.8 | 52.6 | 1682 | 49.7 | 46.8 | 52.6 | 1908 | 50.1 | 44.6 | 36.7 | 52.8 | 229 | 46.8 | 41.3 | 52.3 | 435 | 43.4 | 38.2 | 48.7 | 470 | 44.9 |
| Lesotho | 2004 | 64.1 | 45.1 | 79.5 | 38 | 56.7 | 53.2 | 60.2 | 1005 | 56.6 | 51.1 | 62.0 | 491 | 56.8 | 9.2 | 1.5 | 40.5 | 10 | 34.1 | 28.1 | 40.7 | 274 | 43.2 | 33.2 | 53.7 | 115 | 36.4 |
| Lesotho | 2009 | 59.6 | 39.5 | 77.0 | 34 | 48.1 | 44.4 | 51.8 | 994 | 51.5 | 46.9 | 56.1 | 684 | 49.8 | 65.6 | 30.1 | 89.4 | 8 | 54.9 | 47.5 | 62.1 | 254 | 51.3 | 42.7 | 59.8 | 172 | 53.5 |
| Lesotho | 2014 | 74.5 | 36.3 | 93.8 | 9 | 65.1 | 60.8 | 69.2 | 622 | 65.4 | 60.9 | 69.6 | 756 | 65.3 | 47.4 | 5.2 | 93.6 | 2 | 72.5 | 64.5 | 79.2 | 145 | 62.7 | 52.8 | 71.5 | 180 | 66.9 |
| Lesotho | 2018 | 48.0 | 13.9 | 84.1 | 7 | 58.4 | 52.5 | 64.0 | 413 | 55.9 | 51.3 | 60.3 | 754 | 56.4 | . | . | . | . | 66.9 | 55.0 | 76.9 | 79 | 54.5 | 43.5 | 65.0 | 136 | 59.0 |
| Liberia | 2007 | 68.6 | 63.6 | 73.1 | 989 | 66.5 | 62.2 | 70.5 | 851 | 58.5 | 52.0 | 64.7 | 355 | 66.0 | 30.2 | 22.9 | 38.7 | 238 | 31.9 | 25.1 | 39.6 | 196 | 19.0 | 10.3 | 32.4 | 69 | 29.1 |
| Liberia | 2013 | 64.1 | 59.3 | 68.7 | 1262 | 62.4 | 57.4 | 67.1 | 1127 | 56.4 | 49.9 | 62.7 | 675 | 61.2 | 59.5 | 51.3 | 67.2 | 298 | 56.2 | 46.0 | 65.8 | 272 | 47.5 | 38.0 | 57.2 | 148 | 55.2 |
| Madagascar | 1997 | 31.9 | 27.2 | 37.0 | 574 | 31.5 | 28.4 | 34.7 | 1321 | 39.3 | 34.8 | 44.0 | 524 | 33.2 | 39.2 | 32.0 | 47.0 | 153 | 49.9 | 44.3 | 55.6 | 346 | 53.3 | 43.7 | 62.6 | 129 | 48.0 |
| Madagascar | 2003 | 55.1 | 47.8 | 62.2 | 465 | 59.0 | 53.2 | 64.5 | 1025 | 71.8 | 62.6 | 79.4 | 659 | 60.6 | 58.8 | 47.5 | 69.3 | 114 | 68.0 | 61.2 | 74.1 | 247 | 79.7 | 69.3 | 87.2 | 138 | 67.2 |
| Madagascar | 2008 | 66.5 | 63.2 | 69.6 | 1348 | 71.9 | 69.6 | 74.1 | 2398 | 72.7 | 67.6 | 77.2 | 958 | 70.7 | 42.7 | 36.6 | 48.9 | 370 | 52.3 | 46.4 | 58.1 | 598 | 58.0 | 50.2 | 65.4 | 246 | 50.7 |
| Madagascar | 2018 | 41.9 | 38.3 | 45.7 | 1319 | 46.6 | 43.8 | 49.4 | 2570 | 45.3 | 41.6 | 49.0 | 1350 | 45.2 | 42.4 | 36.0 | 49.0 | 362 | 52.9 | 48.1 | 57.7 | 639 | 53.3 | 47.2 | 59.4 | 382 | 50.6 |
| Malawi | 2000 | 69.5 | 66.8 | 72.2 | 1384 | 69.9 | 68.1 | 71.7 | 3171 | 75.5 | 70.0 | 80.3 | 416 | 70.2 | 43.3 | 37.5 | 49.2 | 329 | 41.7 | 38.0 | 45.6 | 800 | 62.5 | 50.2 | 73.4 | 113 | 44.0 |
| Malawi | 2004 | 63.0 | 58.9 | 66.8 | 1149 | 68.7 | 66.5 | 70.8 | 3007 | 72.3 | 67.7 | 76.5 | 506 | 67.7 | 44.4 | 35.8 | 53.3 | 253 | 54.6 | 50.2 | 59.0 | 726 | 58.9 | 48.9 | 68.2 | 120 | 52.8 |
| Malawi | 2006 | 55.1 | 51.9 | 58.3 | 2146 | 59.1 | 56.9 | 61.3 | 7002 | 59.7 | 55.0 | 64.3 | 1203 | 58.3 | 57.3 | 49.5 | 64.6 | 447 | 55.6 | 51.8 | 59.3 | 1521 | 62.2 | 54.8 | 69.1 | 326 | 56.8 |
| Malawi | 2010 | 93.5 | 91.6 | 95.1 | 1204 | 94.2 | 93.4 | 95.0 | 5416 | 94.5 | 92.6 | 95.9 | 1135 | 94.2 | 67.7 | 59.7 | 74.7 | 242 | 71.8 | 68.0 | 75.4 | 1166 | 72.9 | 64.3 | 80.1 | 255 | 71.4 |
| Malawi | 2013 | 74.9 | 70.7 | 78.7 | 848 | 74.0 | 72.0 | 75.9 | 5375 | 76.1 | 72.5 | 79.3 | 1352 | 74.5 | 71.3 | 60.4 | 80.2 | 194 | 69.0 | 64.7 | 73.0 | 1176 | 74.1 | 66.9 | 80.2 | 314 | 70.2 |
| Malawi | 2015 | 78.7 | 74.6 | 82.3 | 746 | 77.3 | 75.5 | 79.0 | 4463 | 71.6 | 68.0 | 74.9 | 1474 | 76.3 | 60.8 | 53.0 | 68.0 | 203 | 58.2 | 54.4 | 61.9 | 1065 | 69.8 | 63.0 | 75.9 | 368 | 61.0 |
| Maldives | 2009 | 66.4 | 58.1 | 73.8 | 173 | 66.2 | 60.8 | 71.2 | 564 | 56.4 | 52.1 | 60.5 | 927 | 60.1 | 34.6 | 17.9 | 56.2 | 30 | 52.4 | 42.2 | 62.4 | 151 | 46.9 | 39.7 | 54.3 | 231 | 47.8 |
| Maldives | 2016 | 71.0 | 42.9 | 88.9 | 15 | 67.4 | 57.2 | 76.1 | 179 | 66.2 | 62.0 | 70.2 | 974 | 66.5 | 28.5 | 3.4 | 81.8 | 3 | 61.6 | 44.8 | 76.0 | 47 | 64.2 | 54.6 | 72.8 | 238 | 63.5 |
| Mali | 1995 | 9.0 | 7.8 | 10.5 | 3234 | 11.6 | 8.3 | 16.0 | 456 | 19.2 | 13.4 | 26.8 | 176 | 9.8 | 8.6 | 6.6 | 11.1 | 857 | 7.1 | 3.5 | 13.8 | 131 | 7.8 | 2.4 | 22.2 | 48 | 8.4 |
| Mali | 2001 | 30.3 | 27.9 | 32.8 | 4436 | 31.7 | 26.1 | 37.8 | 585 | 34.6 | 26.2 | 44.0 | 238 | 30.6 | 25.6 | 22.3 | 29.3 | 1215 | 22.8 | 14.9 | 33.2 | 150 | 18.5 | 9.0 | 34.3 | 53 | 25.1 |
| Mali | 2006 | 43.5 | 40.8 | 46.2 | 4661 | 46.1 | 41.3 | 50.9 | 576 | 53.9 | 47.8 | 59.9 | 344 | 44.3 | 38.4 | 34.8 | 42.2 | 1184 | 37.2 | 28.4 | 46.9 | 156 | 28.9 | 18.4 | 42.3 | 81 | 37.8 |
| Mali | 2009 | 57.2 | 55.1 | 59.3 | 8322 | 55.2 | 52.0 | 58.3 | 1395 | 59.6 | 55.5 | 63.6 | 881 | 57.1 | 20.1 | 17.9 | 22.6 | 2299 | 22.1 | 17.6 | 27.4 | 357 | 21.6 | 16.3 | 28.0 | 257 | 20.5 |
| Mali | 2012 | 57.9 | 55.1 | 60.6 | 3179 | 52.9 | 47.0 | 58.7 | 369 | 61.4 | 56.5 | 66.1 | 404 | 57.8 | 30.9 | 26.8 | 35.2 | 798 | 42.3 | 31.3 | 54.1 | 81 | 40.3 | 31.0 | 50.3 | 120 | 32.8 |
| Mali | 2015 | 53.6 | 51.3 | 55.8 | 4967 | 52.6 | 48.1 | 57.2 | 767 | 47.6 | 42.4 | 52.9 | 494 | 53.2 | 32.3 | 29.0 | 35.8 | 1268 | 32.8 | 25.0 | 41.8 | 184 | 34.9 | 25.8 | 45.3 | 116 | 32.6 |
| Mali | 2018 | 63.9 | 61.2 | 66.5 | 2752 | 64.6 | 59.4 | 69.6 | 504 | 62.1 | 57.3 | 66.7 | 670 | 63.7 | 44.6 | 40.2 | 49.1 | 695 | 38.9 | 29.9 | 48.7 | 129 | 25.1 | 18.4 | 33.3 | 173 | 40.4 |
| Mauritania | 2007 | 45.1 | 41.3 | 48.8 | 1042 | 45.2 | 41.8 | 48.7 | 1162 | 46.7 | 41.7 | 51.9 | 494 | 44.3 | 8.4 | 5.1 | 13.4 | 248 | 12.1 | 8.7 | 16.7 | 313 | 14.3 | 8.4 | 23.2 | 127 | 11.5 |
| Mauritania | 2011 | 58.8 | 54.7 | 62.8 | 872 | 53.5 | 50.1 | 56.9 | 1307 | 53.5 | 48.5 | 58.5 | 616 | 55.7 | 26.9 | 20.2 | 34.8 | 258 | 26.4 | 21.4 | 32.0 | 353 | 23.8 | 17.0 | 32.2 | 154 | 26.9 |
| Mauritania | 2015 | 66.7 | 62.6 | 70.5 | 1055 | 61.5 | 58.2 | 64.6 | 2371 | 54.9 | 48.6 | 61.0 | 737 | 61.8 | 48.4 | 41.2 | 55.6 | 242 | 41.8 | 37.0 | 46.8 | 519 | 29.0 | 20.4 | 39.3 | 153 | 41.4 |
| Moldova | 2005 | 32.9 | 10.5 | 67.1 | 3 | 70.5 | 30.9 | 92.8 | 7 | 66.5 | 62.9 | 69.9 | 641 | 66.4 | . | . | . | . | 0.0 | - | - | 2 | 46.2 | 37.2 | 55.5 | 156 | 45.5 |
| Moldova | 2012 | . | . | . | . | 68.5 | 19.8 | 95.0 | 8 | 60.8 | 56.6 | 64.9 | 710 | 60.9 | . | . | . | . | 0.0 | - | - | 1 | 36.7 | 29.2 | 44.9 | 174 | 36.4 |
| Mongolia | 2005 | 77.3 | 65.0 | 86.2 | 71 | 77.3 | 67.5 | 84.8 | 119 | 77.5 | 74.8 | 80.1 | 1270 | 77.5 | 63.5 | 41.1 | 81.3 | 22 | 72.7 | 56.8 | 84.4 | 33 | 55.3 | 49.5 | 60.9 | 344 | 57.2 |
| Mongolia | 2010 | 73.3 | 60.9 | 82.9 | 84 | 72.4 | 63.8 | 79.6 | 142 | 71.3 | 68.1 | 74.2 | 1464 | 71.4 | 76.3 | 51.5 | 90.7 | 22 | 68.7 | 49.2 | 83.3 | 29 | 65.0 | 58.7 | 70.8 | 359 | 65.7 |
| Mongolia | 2013 | 72.5 | 64.0 | 79.6 | 142 | 72.4 | 64.8 | 78.9 | 167 | 70.9 | 68.5 | 73.2 | 2066 | 71.1 | 57.4 | 40.2 | 73.0 | 34 | 52.7 | 38.1 | 66.8 | 37 | 46.2 | 41.8 | 50.8 | 573 | 47.1 |
| Mongolia | 2018 | 83.2 | 72.5 | 90.3 | 120 | 61.5 | 48.7 | 72.9 | 132 | 70.0 | 66.9 | 72.9 | 1947 | 70.2 | 42.3 | 22.9 | 64.4 | 36 | 46.3 | 26.5 | 67.4 | 35 | 52.2 | 45.6 | 58.8 | 545 | 51.5 |
| Montenegro | 2005 | . | . | . | . | 41.7 | 28.1 | 56.7 | 81 | 20.0 | 14.4 | 27.1 | 269 | 25.0 | . | . | . | . | 29.0 | 12.8 | 53.3 | 18 | 17.1 | 9.0 | 30.0 | 53 | 20.1 |
| Montenegro | 2013 | 62.8 | 12.3 | 95.3 | 3 | 29.7 | 18.2 | 44.5 | 74 | 10.9 | 7.5 | 15.7 | 417 | 14.4 | . | . | . | . | 5.1 | 1.0 | 21.9 | 18 | 19.5 | 11.7 | 30.8 | 90 | 16.8 |
| Montenegro | 2018 | 34.9 | 5.5 | 83.0 | 14 | 19.7 | 9.5 | 36.3 | 44 | 23.8 | 17.6 | 31.2 | 374 | 24.1 | 42.6 | 26.4 | 60.5 | 3 | 17.7 | 4.5 | 49.8 | 14 | 20.1 | 12.3 | 31.1 | 84 | 20.5 |
| Mozambique | 2003 | 68.2 | 65.4 | 71.0 | 1691 | 61.7 | 59.0 | 64.4 | 2223 | 41.0 | 34.8 | 47.5 | 231 | 63.8 | 32.0 | 26.9 | 37.5 | 400 | 29.0 | 24.8 | 33.5 | 575 | 21.5 | 9.8 | 41.0 | 52 | 30.0 |
| Mozambique | 2008 | 69.7 | 65.9 | 73.3 | 1449 | 60.1 | 57.1 | 63.0 | 2858 | 60.0 | 54.0 | 65.7 | 570 | 63.2 | 35.1 | 29.3 | 41.3 | 355 | 39.3 | 34.7 | 44.2 | 703 | 31.7 | 22.4 | 42.7 | 171 | 37.2 |
| Mozambique | 2011 | 79.9 | 77.1 | 82.5 | 1507 | 75.0 | 72.7 | 77.2 | 2410 | 74.6 | 70.0 | 78.6 | 695 | 76.7 | 39.3 | 32.8 | 46.2 | 319 | 41.4 | 36.4 | 46.5 | 553 | 44.8 | 37.0 | 52.9 | 172 | 41.1 |
| Namibia | 2006 | 69.7 | 63.5 | 75.3 | 256 | 69.5 | 65.0 | 73.6 | 636 | 65.2 | 61.5 | 68.8 | 1243 | 66.9 | 19.2 | 9.7 | 34.5 | 57 | 21.8 | 15.3 | 30.2 | 149 | 25.7 | 20.1 | 32.2 | 289 | 23.9 |
| Namibia | 2013 | 73.7 | 64.8 | 81.0 | 158 | 74.5 | 69.4 | 78.9 | 480 | 70.0 | 67.5 | 72.4 | 1413 | 71.2 | 33.2 | 19.8 | 50.1 | 43 | 50.2 | 39.5 | 60.9 | 112 | 49.1 | 43.5 | 54.8 | 370 | 48.5 |
| Nepal | 1996 | 17.4 | 15.3 | 19.7 | 2275 | 18.2 | 14.1 | 23.1 | 317 | 20.4 | 15.8 | 25.9 | 286 | 17.8 | 76.6 | 72.2 | 80.5 | 516 | 74.4 | 65.4 | 81.8 | 91 | 62.6 | 50.1 | 73.6 | 70 | 74.9 |
| Nepal | 2006 | 31.8 | 27.7 | 36.1 | 1189 | 37.1 | 31.8 | 42.8 | 397 | 42.5 | 36.8 | 48.4 | 517 | 35.5 | 53.4 | 46.5 | 60.3 | 255 | 66.3 | 53.0 | 77.4 | 82 | 42.4 | 30.8 | 54.9 | 136 | 53.0 |
| Nepal | 2011 | 34.7 | 30.1 | 39.5 | 810 | 44.5 | 38.3 | 50.9 | 392 | 55.5 | 51.0 | 59.9 | 778 | 44.5 | 75.6 | 65.4 | 83.6 | 208 | 73.7 | 63.7 | 81.7 | 103 | 60.0 | 51.3 | 68.2 | 186 | 69.6 |
| Nepal | 2014 | 54.0 | 48.9 | 59.1 | 822 | 49.9 | 43.4 | 56.5 | 349 | 44.0 | 39.8 | 48.3 | 915 | 48.7 | 61.1 | 49.9 | 71.2 | 171 | 52.2 | 38.1 | 65.9 | 75 | 55.5 | 46.1 | 64.5 | 206 | 56.9 |
| Nepal | 2016 | 49.1 | 42.6 | 55.6 | 543 | 56.8 | 51.0 | 62.4 | 380 | 57.5 | 53.7 | 61.2 | 1047 | 54.9 | 71.5 | 59.8 | 80.9 | 111 | 67.7 | 56.7 | 76.9 | 89 | 63.3 | 56.4 | 69.7 | 267 | 66.1 |
| Niger | 1998 | 25.9 | 23.6 | 28.3 | 2650 | 37.6 | 31.7 | 43.8 | 327 | 48.8 | 37.9 | 59.9 | 136 | 27.6 | 0.6 | 0.2 | 1.5 | 692 | 1.0 | 0.1 | 6.8 | 88 | 5.8 | 1.5 | 20.3 | 36 | 0.8 |
| Niger | 2006 | 45.1 | 42.2 | 48.0 | 2988 | 50.5 | 44.3 | 56.8 | 414 | 67.1 | 58.5 | 74.8 | 211 | 46.4 | 13.5 | 11.1 | 16.4 | 798 | 14.7 | 8.6 | 24.0 | 110 | 6.2 | 1.7 | 20.2 | 37 | 13.5 |
| Niger | 2012 | 51.5 | 48.7 | 54.4 | 3877 | 58.5 | 53.2 | 63.6 | 528 | 66.3 | 59.6 | 72.5 | 346 | 52.9 | 22.5 | 18.9 | 26.5 | 1091 | 28.7 | 20.2 | 39.0 | 138 | 29.2 | 17.5 | 44.6 | 72 | 23.3 |
| Nigeria | 1999 | 36.9 | 33.0 | 40.9 | 1147 | 36.4 | 32.1 | 41.0 | 589 | 37.5 | 33.1 | 42.2 | 623 | 36.9 | 8.3 | 5.3 | 13.0 | 271 | 19.6 | 13.3 | 27.9 | 134 | 24.5 | 17.8 | 32.7 | 150 | 15.5 |
| Nigeria | 2003 | 27.0 | 22.3 | 32.4 | 1168 | 35.0 | 29.7 | 40.6 | 599 | 33.5 | 27.8 | 39.7 | 628 | 30.6 | 10.8 | 7.2 | 16.0 | 314 | 14.2 | 8.4 | 22.9 | 139 | 32.0 | 21.9 | 44.1 | 158 | 17.2 |
| Nigeria | 2007 | 27.7 | 25.0 | 30.5 | 2837 | 32.5 | 29.4 | 35.7 | 1498 | 30.8 | 27.7 | 34.2 | 1806 | 29.9 | 8.0 | 5.9 | 10.7 | 840 | 12.2 | 9.0 | 16.3 | 388 | 17.9 | 13.6 | 23.2 | 445 | 11.7 |
| Nigeria | 2008 | 30.0 | 27.8 | 32.3 | 5540 | 42.5 | 39.7 | 45.4 | 2492 | 42.5 | 40.2 | 44.8 | 3196 | 36.8 | 7.2 | 5.4 | 9.5 | 1395 | 13.5 | 10.8 | 16.8 | 634 | 20.5 | 17.6 | 23.6 | 860 | 13.1 |
| Nigeria | 2011 | 19.9 | 17.7 | 22.2 | 4532 | 23.0 | 20.8 | 25.4 | 2028 | 25.8 | 23.5 | 28.3 | 3473 | 22.9 | 8.4 | 6.5 | 10.7 | 1291 | 18.5 | 13.7 | 24.5 | 524 | 21.2 | 17.9 | 24.9 | 899 | 15.1 |
| Nigeria | 2013 | 28.7 | 26.6 | 30.8 | 5617 | 35.0 | 32.6 | 37.6 | 2402 | 38.5 | 36.5 | 40.5 | 4378 | 33.2 | 7.1 | 5.5 | 9.0 | 1359 | 20.0 | 16.1 | 24.6 | 549 | 31.0 | 27.4 | 34.9 | 1026 | 17.4 |
| Nigeria | 2016 | 31.0 | 28.5 | 33.6 | 2913 | 36.3 | 33.2 | 39.6 | 1753 | 32.9 | 30.8 | 35.0 | 3392 | 32.8 | 19.6 | 15.9 | 24.0 | 700 | 20.8 | 16.7 | 25.5 | 439 | 30.6 | 26.6 | 34.9 | 867 | 23.7 |
| Nigeria | 2018 | 34.2 | 32.3 | 36.2 | 5648 | 44.4 | 41.1 | 47.8 | 1919 | 50.0 | 48.2 | 51.8 | 5251 | 42.1 | 20.6 | 17.8 | 23.6 | 1404 | 24.3 | 20.3 | 28.8 | 494 | 39.7 | 36.6 | 42.9 | 1295 | 28.7 |
| North Macedonia | 2005 | 32.9 | 18.7 | 51.1 | 195 | 27.9 | 20.7 | 36.5 | 828 | 23.1 | 14.4 | 35.1 | 413 | 26.6 | 8.4 | 1.8 | 31.5 | 39 | 21.0 | 6.2 | 51.6 | 130 | 8.7 | 3.5 | 20.2 | 64 | 16.2 |
| North Macedonia | 2011 | 3.4 | 0.4 | 23.4 | 9 | 19.0 | 12.1 | 28.5 | 167 | 22.8 | 17.7 | 28.8 | 327 | 21.0 | 0.0 | - | - | 3 | 28.1 | 14.5 | 47.4 | 41 | 20.5 | 11.7 | 33.4 | 68 | 23.0 |
| Pakistan | 2012 | 18.8 | 16.2 | 21.8 | 2231 | 12.3 | 9.4 | 15.9 | 617 | 20.0 | 17.1 | 23.2 | 1307 | 18.0 | 39.7 | 34.2 | 45.6 | 566 | 38.4 | 28.7 | 49.1 | 168 | 33.8 | 27.9 | 40.2 | 342 | 37.7 |
| Pakistan | 2017 | 21.7 | 18.3 | 25.6 | 2046 | 18.6 | 14.3 | 23.8 | 498 | 17.2 | 14.5 | 20.3 | 1328 | 19.6 | 56.8 | 51.1 | 62.3 | 585 | 44.0 | 32.5 | 56.2 | 163 | 37.5 | 31.3 | 44.1 | 369 | 47.5 |
| Peru | 1996 | 39.9 | 35.6 | 44.4 | 678 | 42.5 | 40.0 | 45.0 | 2850 | 48.4 | 46.2 | 50.7 | 2982 | 45.3 | 68.5 | 59.5 | 76.4 | 162 | 65.6 | 60.8 | 70.1 | 643 | 40.9 | 36.3 | 45.7 | 714 | 53.0 |
| Peru | 2000 | 59.6 | 53.7 | 65.3 | 425 | 55.1 | 52.5 | 57.7 | 2164 | 53.6 | 51.0 | 56.1 | 2421 | 54.6 | 83.6 | 71.6 | 91.2 | 88 | 70.2 | 64.6 | 75.3 | 497 | 63.2 | 58.1 | 68.1 | 577 | 67.2 |
| Peru | 2004 | 45.9 | 32.4 | 60.1 | 52 | 52.8 | 47.0 | 58.6 | 377 | 36.2 | 30.7 | 42.1 | 556 | 42.0 | 78.8 | 48.5 | 93.6 | 15 | 66.4 | 55.2 | 76.0 | 85 | 56.2 | 44.1 | 67.6 | 133 | 60.5 |
| Peru | 2005 | 58.7 | 42.0 | 73.6 | 48 | 48.6 | 41.7 | 55.6 | 442 | 50.2 | 44.4 | 56.1 | 612 | 50.0 | 41.6 | 18.6 | 69.0 | 13 | 77.4 | 69.3 | 83.9 | 106 | 57.5 | 46.5 | 67.8 | 154 | 64.5 |
| Peru | 2006 | 51.5 | 36.4 | 66.2 | 64 | 52.7 | 46.4 | 58.9 | 427 | 48.3 | 42.3 | 54.4 | 629 | 50.0 | 82.6 | 35.4 | 97.6 | 13 | 70.0 | 56.7 | 80.6 | 90 | 57.2 | 45.3 | 68.4 | 148 | 62.7 |
| Peru | 2007 | 61.8 | 39.7 | 79.9 | 42 | 53.3 | 44.8 | 61.7 | 383 | 46.6 | 41.1 | 52.2 | 568 | 49.6 | 76.3 | 45.6 | 92.5 | 10 | 74.9 | 52.8 | 88.8 | 84 | 64.4 | 52.9 | 74.5 | 129 | 68.7 |
| Peru | 2008 | 67.0 | 53.0 | 78.5 | 85 | 68.7 | 63.8 | 73.2 | 800 | 54.7 | 50.4 | 59.0 | 1540 | 59.5 | 78.8 | 50.1 | 93.2 | 20 | 77.2 | 66.2 | 85.5 | 152 | 60.3 | 51.0 | 69.0 | 373 | 65.7 |
| Peru | 2009 | 74.4 | 64.5 | 82.3 | 135 | 64.3 | 60.8 | 67.7 | 1304 | 45.5 | 42.9 | 48.2 | 2574 | 51.6 | 85.1 | 66.3 | 94.3 | 34 | 81.6 | 75.1 | 86.7 | 282 | 62.9 | 57.7 | 67.9 | 585 | 68.5 |
| Peru | 2010 | 67.5 | 55.9 | 77.3 | 87 | 60.2 | 56.5 | 63.7 | 1214 | 46.5 | 43.7 | 49.3 | 2237 | 51.1 | 78.0 | 47.4 | 93.3 | 18 | 77.6 | 70.7 | 83.3 | 255 | 64.1 | 58.6 | 69.3 | 493 | 68.3 |
| Peru | 2011 | 71.7 | 61.2 | 80.2 | 113 | 64.7 | 61.1 | 68.2 | 1131 | 45.5 | 42.5 | 48.7 | 2145 | 51.6 | 88.3 | 51.1 | 98.2 | 18 | 83.7 | 76.3 | 89.2 | 234 | 65.8 | 59.6 | 71.4 | 528 | 70.6 |
| Peru | 2012 | 73.1 | 64.1 | 80.6 | 120 | 67.9 | 64.3 | 71.3 | 1107 | 47.8 | 44.9 | 50.7 | 2393 | 53.7 | 87.5 | 67.6 | 95.9 | 28 | 80.0 | 73.4 | 85.3 | 254 | 62.0 | 55.7 | 67.8 | 545 | 67.6 |
| Peru | 2013 | 72.3 | 61.0 | 81.4 | 97 | 69.4 | 65.1 | 73.5 | 948 | 50.6 | 47.7 | 53.5 | 2344 | 55.5 | 90.1 | 71.2 | 97.1 | 20 | 85.5 | 78.3 | 90.6 | 199 | 68.1 | 61.8 | 73.8 | 531 | 72.3 |
| Peru | 2014 | 85.1 | 73.9 | 92.0 | 71 | 66.4 | 62.6 | 70.0 | 944 | 50.1 | 47.7 | 52.6 | 2703 | 54.1 | 93.2 | 64.4 | 99.0 | 19 | 78.9 | 71.8 | 84.5 | 213 | 65.2 | 60.2 | 69.9 | 646 | 68.4 |
| Peru | 2015 | 78.8 | 70.3 | 85.4 | 155 | 67.8 | 64.9 | 70.6 | 2001 | 48.1 | 46.4 | 49.8 | 6809 | 52.8 | 73.5 | 53.3 | 87.1 | 29 | 80.8 | 75.3 | 85.3 | 400 | 60.5 | 56.9 | 64.0 | 1418 | 64.9 |
| Peru | 2016 | 73.2 | 62.8 | 81.6 | 128 | 67.1 | 63.6 | 70.4 | 1635 | 46.5 | 44.6 | 48.4 | 5960 | 51.0 | 90.8 | 55.3 | 98.7 | 16 | 79.7 | 74.2 | 84.3 | 325 | 66.8 | 63.0 | 70.4 | 1105 | 69.8 |
| Peru | 2017 | 79.9 | 70.8 | 86.7 | 121 | 69.4 | 66.2 | 72.4 | 1566 | 43.7 | 41.9 | 45.5 | 6784 | 48.7 | 84.0 | 62.5 | 94.3 | 32 | 77.1 | 71.7 | 81.7 | 347 | 61.0 | 57.4 | 64.6 | 1413 | 64.3 |
| Peru | 2018 | 72.7 | 60.9 | 81.9 | 113 | 65.3 | 62.0 | 68.4 | 1537 | 47.1 | 45.5 | 48.7 | 6690 | 50.5 | 84.4 | 54.9 | 96.0 | 19 | 80.4 | 74.2 | 85.4 | 340 | 63.1 | 59.7 | 66.3 | 1378 | 66.5 |
| Philippines | 1993 | 56.3 | 46.0 | 66.1 | 113 | 40.5 | 38.0 | 43.1 | 1398 | 33.6 | 31.1 | 36.2 | 1893 | 37.0 | 47.3 | 28.1 | 67.3 | 27 | 36.5 | 31.4 | 41.9 | 336 | 18.1 | 14.7 | 22.2 | 458 | 26.1 |
| Philippines | 1998 | 44.9 | 33.7 | 56.6 | 87 | 35.9 | 32.7 | 39.1 | 1035 | 33.5 | 31.0 | 36.1 | 1870 | 34.5 | 44.7 | 25.5 | 65.5 | 26 | 48.4 | 42.1 | 54.8 | 265 | 32.1 | 27.7 | 36.9 | 466 | 37.5 |
| Philippines | 2003 | 56.3 | 43.5 | 68.3 | 60 | 48.3 | 44.3 | 52.3 | 779 | 44.8 | 42.2 | 47.5 | 1826 | 46.0 | 48.6 | 23.6 | 74.3 | 16 | 45.9 | 38.8 | 53.2 | 182 | 28.2 | 24.0 | 32.8 | 426 | 33.5 |
| Philippines | 2008 | 61.2 | 46.2 | 74.3 | 47 | 51.9 | 47.4 | 56.5 | 608 | 45.9 | 43.3 | 48.5 | 1837 | 47.5 | 60.9 | 26.2 | 87.3 | 8 | 41.3 | 33.5 | 49.5 | 169 | 30.9 | 26.3 | 35.8 | 416 | 34.0 |
| Rwanda | 2000 | 44.2 | 40.4 | 48.0 | 951 | 46.1 | 43.2 | 49.0 | 1762 | 44.8 | 38.2 | 51.6 | 379 | 45.3 | 85.1 | 79.0 | 89.7 | 227 | 86.0 | 82.5 | 88.9 | 445 | 59.0 | 47.1 | 69.9 | 85 | 83.3 |
| Rwanda | 2005 | 56.5 | 52.7 | 60.1 | 904 | 62.8 | 60.5 | 65.0 | 2214 | 65.3 | 58.2 | 71.8 | 294 | 61.3 | 89.7 | 84.5 | 93.3 | 223 | 89.7 | 86.6 | 92.1 | 585 | 71.9 | 59.0 | 82.0 | 68 | 88.4 |
| Rwanda | 2010 | 68.8 | 64.6 | 72.8 | 534 | 72.4 | 70.4 | 74.3 | 2306 | 67.4 | 62.2 | 72.2 | 319 | 71.3 | 82.8 | 74.3 | 89.0 | 118 | 86.3 | 82.7 | 89.3 | 509 | 78.5 | 69.0 | 85.7 | 82 | 84.9 |
| Rwanda | 2014 | 84.2 | 80.3 | 87.4 | 418 | 80.4 | 78.6 | 82.1 | 2238 | 77.3 | 73.5 | 80.7 | 513 | 80.5 | 83.3 | 72.4 | 90.5 | 74 | 88.0 | 84.7 | 90.7 | 490 | 86.5 | 80.0 | 91.1 | 139 | 87.3 |
| Sao Tome and Principe | 2008 | 41.2 | 26.2 | 58.0 | 49 | 42.3 | 37.2 | 47.7 | 563 | 44.8 | 34.5 | 55.6 | 166 | 42.9 | 14.9 | 4.8 | 37.9 | 17 | 54.7 | 43.0 | 65.9 | 122 | 53.2 | 36.9 | 68.8 | 53 | 51.4 |
| Sao Tome and Principe | 2014 | 39.2 | 23.2 | 57.9 | 32 | 39.8 | 33.8 | 46.0 | 461 | 35.8 | 30.0 | 42.2 | 265 | 38.3 | 84.2 | 37.2 | 98.0 | 6 | 72.0 | 58.2 | 82.6 | 93 | 75.3 | 62.1 | 85.0 | 70 | 73.8 |
| Senegal | 2005 | 22.8 | 19.9 | 26.0 | 3371 | 20.9 | 17.0 | 25.4 | 921 | 26.7 | 17.6 | 38.4 | 259 | 22.6 | 30.3 | 26.2 | 34.8 | 950 | 42.5 | 36.0 | 49.2 | 260 | 42.6 | 29.8 | 56.4 | 80 | 34.1 |
| Senegal | 2010 | 48.3 | 45.3 | 51.2 | 3586 | 48.0 | 43.5 | 52.4 | 940 | 45.5 | 38.0 | 53.1 | 385 | 48.0 | 38.7 | 35.0 | 42.6 | 972 | 39.5 | 31.9 | 47.6 | 249 | 39.7 | 29.3 | 51.1 | 113 | 39.0 |
| Senegal | 2012 | 30.4 | 26.9 | 34.3 | 1885 | 31.7 | 26.5 | 37.4 | 560 | 42.4 | 33.6 | 51.7 | 265 | 32.1 | 34.6 | 28.9 | 40.9 | 433 | 36.6 | 27.5 | 46.8 | 152 | 46.8 | 35.4 | 58.5 | 87 | 37.0 |
| Senegal | 2014 | 28.1 | 24.6 | 31.9 | 1810 | 32.7 | 27.5 | 38.4 | 493 | 32.8 | 24.8 | 41.9 | 281 | 29.7 | 26.9 | 21.1 | 33.5 | 424 | 48.1 | 38.1 | 58.4 | 107 | 32.3 | 20.4 | 47.0 | 81 | 32.4 |
| Senegal | 2015 | 31.2 | 28.4 | 34.1 | 1862 | 27.2 | 22.6 | 32.2 | 529 | 32.5 | 26.7 | 38.9 | 321 | 30.5 | 34.4 | 27.3 | 42.3 | 408 | 33.1 | 23.1 | 44.9 | 130 | 29.7 | 18.7 | 43.6 | 88 | 33.3 |
| Senegal | 2016 | 30.8 | 27.7 | 34.2 | 1809 | 27.5 | 22.3 | 33.4 | 501 | 26.1 | 20.6 | 32.4 | 331 | 29.4 | 35.1 | 29.6 | 41.0 | 398 | 35.8 | 25.2 | 48.1 | 118 | 40.6 | 25.7 | 57.4 | 89 | 36.4 |
| Senegal | 2017 | 31.7 | 29.3 | 34.2 | 3016 | 36.6 | 32.7 | 40.7 | 968 | 36.0 | 31.6 | 40.8 | 833 | 33.6 | 37.4 | 33.1 | 41.8 | 712 | 53.0 | 45.0 | 60.8 | 227 | 46.0 | 37.4 | 54.9 | 203 | 42.1 |
| Serbia | 2005 | . | . | . | . | 19.9 | 14.3 | 27.1 | 657 | 16.8 | 13.6 | 20.7 | 779 | 17.5 | . | . | . | . | 13.7 | 7.2 | 24.4 | 169 | 15.9 | 10.9 | 22.7 | 165 | 15.3 |
| Serbia | 2010 | 15.1 | 4.2 | 41.6 | 10 | 7.2 | 3.9 | 13.0 | 151 | 7.5 | 5.5 | 10.2 | 1026 | 7.6 | 22.7 | 2.3 | 78.8 | 5 | 4.9 | 1.1 | 19.1 | 37 | 15.2 | 9.6 | 23.2 | 204 | 13.7 |
| Serbia | 2014 | 82.6 | 51.5 | 95.5 | 11 | 48.4 | 34.0 | 63.0 | 98 | 50.6 | 44.7 | 56.5 | 849 | 50.8 | 0.0 | - | - | 2 | 0.0 | - | - | 15 | 14.0 | 6.3 | 28.4 | 152 | 12.8 |
| Sierra Leone | 2005 | 34.2 | 30.7 | 37.8 | 1886 | 24.8 | 19.0 | 31.6 | 234 | 33.4 | 26.7 | 40.8 | 230 | 33.1 | 9.4 | 6.9 | 12.9 | 416 | 4.3 | 1.1 | 15.9 | 50 | 4.7 | 1.2 | 16.7 | 42 | 8.6 |
| Sierra Leone | 2008 | 49.8 | 46.4 | 53.2 | 1713 | 47.0 | 40.8 | 53.3 | 340 | 39.8 | 33.9 | 45.9 | 357 | 48.1 | 12.1 | 8.9 | 16.3 | 440 | 10.0 | 4.8 | 19.8 | 82 | 7.1 | 2.8 | 17.0 | 95 | 11.2 |
| Sierra Leone | 2010 | 46.6 | 43.5 | 49.7 | 2298 | 41.2 | 36.2 | 46.3 | 490 | 39.8 | 35.4 | 44.4 | 627 | 44.6 | 31.5 | 27.0 | 36.3 | 574 | 32.9 | 23.9 | 43.3 | 117 | 30.5 | 22.7 | 39.6 | 140 | 31.5 |
| Sierra Leone | 2013 | 55.6 | 52.0 | 59.1 | 3026 | 54.1 | 49.4 | 58.8 | 672 | 47.9 | 43.3 | 52.4 | 970 | 53.8 | 32.6 | 27.8 | 37.8 | 707 | 24.9 | 18.0 | 33.4 | 180 | 36.4 | 28.8 | 44.7 | 228 | 32.0 |
| Sierra Leone | 2017 | 56.9 | 54.2 | 59.6 | 2725 | 59.3 | 54.8 | 63.6 | 700 | 51.9 | 48.2 | 55.7 | 1319 | 55.7 | 55.0 | 50.7 | 59.2 | 671 | 52.4 | 43.3 | 61.2 | 166 | 47.6 | 41.6 | 53.7 | 333 | 52.2 |
| South Africa | 1998 | 38.4 | 30.1 | 47.4 | 157 | 40.2 | 35.2 | 45.4 | 583 | 38.4 | 35.2 | 41.8 | 1301 | 38.9 | 16.1 | 9.4 | 26.2 | 47 | 3.9 | 1.8 | 8.5 | 124 | 6.6 | 4.1 | 10.4 | 334 | 7.0 |
| South Africa | 2016 | 74.1 | 47.5 | 90.1 | 17 | 71.5 | 61.3 | 79.8 | 122 | 65.9 | 62.6 | 69.1 | 1237 | 66.5 | 0.0 | - | - | 6 | 41.8 | 19.7 | 67.8 | 25 | 31.7 | 25.7 | 38.4 | 315 | 31.6 |
| Suriname | 2006 | 30.1 | 19.5 | 43.4 | 115 | 31.7 | 25.4 | 38.7 | 227 | 36.5 | 32.2 | 41.1 | 450 | 34.4 | 0.0 | - | - | 20 | 1.6 | 0.2 | 10.7 | 54 | 2.9 | 0.9 | 8.6 | 106 | 2.2 |
| Suriname | 2010 | 61.0 | 53.0 | 68.5 | 242 | 49.1 | 43.8 | 54.3 | 459 | 38.9 | 34.3 | 43.6 | 559 | 44.7 | 1.3 | 0.2 | 9.1 | 64 | 5.3 | 1.8 | 14.5 | 106 | 1.9 | 0.8 | 4.5 | 133 | 2.8 |
| Suriname | 2018 | 70.6 | 57.6 | 81.0 | 60 | 57.5 | 49.8 | 64.9 | 225 | 49.6 | 45.4 | 53.9 | 1107 | 51.9 | 11.7 | 3.0 | 36.2 | 17 | 10.2 | 2.4 | 34.5 | 55 | 8.6 | 5.3 | 13.5 | 259 | 8.9 |
| Tajikistan | 2005 | 42.9 | 19.0 | 70.6 | 13 | 60.0 | 42.7 | 75.2 | 32 | 61.2 | 56.7 | 65.5 | 1576 | 60.9 | 0.0 | - | - | 5 | 1.9 | 0.2 | 13.4 | 10 | 26.6 | 20.7 | 33.4 | 373 | 25.4 |
| Tajikistan | 2012 | 30.4 | 20.2 | 43.1 | 48 | 38.8 | 28.2 | 50.6 | 106 | 50.8 | 47.0 | 54.6 | 1784 | 49.6 | 40.7 | 18.7 | 67.3 | 13 | 38.9 | 21.0 | 60.3 | 27 | 33.7 | 28.3 | 39.5 | 384 | 34.3 |
| Tajikistan | 2017 | 72.3 | 58.1 | 83.1 | 53 | 62.0 | 50.4 | 72.3 | 120 | 61.3 | 57.8 | 64.7 | 2148 | 61.6 | 84.0 | 34.4 | 98.1 | 4 | 47.9 | 28.7 | 67.8 | 28 | 34.8 | 29.7 | 40.3 | 521 | 35.8 |
| Tanzania | 1996 | 55.0 | 50.6 | 59.4 | 762 | 57.9 | 55.0 | 60.8 | 1863 | 64.0 | 55.6 | 71.7 | 145 | 57.3 | 34.0 | 26.5 | 42.5 | 188 | 28.3 | 24.0 | 32.9 | 428 | 15.5 | 6.0 | 34.5 | 38 | 29.4 |
| Tanzania | 2004 | 53.7 | 49.0 | 58.4 | 921 | 58.4 | 55.8 | 61.1 | 2177 | 65.9 | 56.8 | 74.1 | 292 | 57.6 | 41.6 | 33.2 | 50.4 | 201 | 40.7 | 35.8 | 45.9 | 551 | 47.6 | 28.8 | 67.0 | 73 | 41.3 |
| Tanzania | 2010 | 41.3 | 36.2 | 46.6 | 790 | 44.8 | 41.8 | 47.8 | 1975 | 60.6 | 53.0 | 67.7 | 408 | 45.1 | 50.4 | 41.7 | 59.0 | 207 | 49.8 | 44.6 | 55.0 | 501 | 47.8 | 33.9 | 62.0 | 95 | 49.8 |
| Tanzania | 2015 | 46.5 | 41.4 | 51.6 | 835 | 51.7 | 48.9 | 54.4 | 2501 | 54.7 | 50.3 | 59.0 | 883 | 51.2 | 55.6 | 47.9 | 63.1 | 201 | 59.6 | 55.0 | 64.1 | 618 | 61.8 | 52.5 | 70.3 | 196 | 59.2 |
| Thailand | 2005 | 52.8 | 41.2 | 64.1 | 105 | 48.8 | 43.7 | 54.0 | 1162 | 49.8 | 45.8 | 53.7 | 2094 | 49.6 | 1.6 | 0.2 | 12.3 | 30 | 4.0 | 2.2 | 7.3 | 317 | 6.5 | 3.8 | 11.0 | 524 | 5.4 |
| Thailand | 2012 | 66.9 | 43.7 | 84.0 | 59 | 48.2 | 39.4 | 57.1 | 558 | 45.0 | 41.0 | 49.0 | 2145 | 46.3 | 26.3 | 10.2 | 52.9 | 17 | 9.4 | 4.6 | 18.1 | 149 | 12.9 | 9.1 | 18.1 | 425 | 12.3 |
| Thailand | 2015 | 37.1 | 19.6 | 58.8 | 108 | 43.6 | 34.5 | 53.2 | 552 | 39.4 | 34.7 | 44.5 | 2714 | 39.9 | 0.2 | 0.0 | 1.3 | 26 | 16.9 | 7.1 | 35.1 | 126 | 26.2 | 18.8 | 35.2 | 505 | 23.1 |
| Timor-Leste | 2009 | 78.1 | 75.4 | 80.6 | 1202 | 78.5 | 75.8 | 81.0 | 1047 | 81.6 | 79.1 | 83.9 | 1425 | 79.6 | 53.7 | 47.8 | 59.4 | 332 | 53.3 | 46.9 | 59.7 | 264 | 48.4 | 41.9 | 54.9 | 364 | 51.5 |
| Timor-Leste | 2016 | 77.6 | 73.4 | 81.2 | 639 | 75.1 | 70.5 | 79.2 | 492 | 74.4 | 71.1 | 77.5 | 1706 | 75.2 | 52.2 | 44.1 | 60.1 | 171 | 50.3 | 39.5 | 61.0 | 96 | 50.2 | 43.6 | 56.7 | 476 | 50.7 |
| Togo | 1998 | 16.4 | 14.2 | 18.8 | 1677 | 19.6 | 16.3 | 23.4 | 829 | 23.1 | 16.6 | 31.1 | 207 | 18.0 | 5.6 | 3.5 | 8.7 | 407 | 15.0 | 10.3 | 21.5 | 232 | 21.6 | 11.9 | 36.0 | 53 | 10.5 |
| Togo | 2006 | 33.6 | 29.4 | 38.1 | 919 | 36.9 | 32.1 | 42.0 | 578 | 38.9 | 33.0 | 45.0 | 250 | 35.8 | 23.6 | 17.2 | 31.5 | 253 | 31.7 | 23.7 | 40.9 | 134 | 38.8 | 25.3 | 54.3 | 59 | 28.8 |
| Togo | 2010 | 46.0 | 42.1 | 50.0 | 992 | 46.8 | 42.4 | 51.3 | 668 | 44.5 | 38.2 | 51.0 | 301 | 46.0 | 54.8 | 47.6 | 61.8 | 277 | 61.5 | 52.9 | 69.5 | 201 | 80.0 | 68.9 | 87.9 | 85 | 62.4 |
| Togo | 2013 | 57.4 | 53.5 | 61.3 | 1224 | 61.5 | 57.3 | 65.6 | 955 | 64.5 | 60.2 | 68.6 | 603 | 60.6 | 50.3 | 43.6 | 57.0 | 258 | 58.1 | 49.9 | 65.8 | 207 | 67.2 | 57.4 | 75.7 | 138 | 57.5 |
| Togo | 2017 | 47.3 | 42.2 | 52.4 | 710 | 49.8 | 45.0 | 54.7 | 682 | 47.8 | 42.5 | 53.1 | 596 | 48.3 | 66.3 | 58.1 | 73.6 | 180 | 60.2 | 50.6 | 69.1 | 163 | 69.3 | 59.5 | 77.6 | 160 | 65.4 |
| Tunisia | 2011 | 48.3 | 38.9 | 57.9 | 168 | 43.4 | 37.1 | 49.9 | 360 | 36.6 | 31.5 | 41.9 | 607 | 39.9 | 15.5 | 6.3 | 33.5 | 44 | 14.0 | 6.9 | 26.2 | 85 | 5.2 | 3.0 | 9.0 | 177 | 8.5 |
| Tunisia | 2018 | 40.9 | 30.2 | 52.6 | 84 | 30.4 | 24.8 | 36.5 | 259 | 31.2 | 27.6 | 34.9 | 869 | 31.6 | 12.2 | 3.7 | 33.6 | 19 | 18.3 | 10.4 | 30.1 | 60 | 12.7 | 8.7 | 18.2 | 220 | 13.8 |
| Turkey | 1993 | 17.6 | 13.8 | 22.1 | 398 | 20.3 | 17.6 | 23.2 | 804 | 22.8 | 18.0 | 28.6 | 245 | 19.9 | 7.0 | 3.2 | 15.0 | 94 | 12.7 | 8.4 | 18.7 | 196 | 9.6 | 4.5 | 19.3 | 72 | 10.5 |
| Turkey | 1998 | 42.4 | 35.9 | 49.1 | 298 | 46.6 | 42.8 | 50.5 | 827 | 59.5 | 52.6 | 66.0 | 300 | 48.6 | 4.7 | 1.1 | 18.3 | 74 | 8.7 | 4.9 | 14.8 | 214 | 5.1 | 1.8 | 13.6 | 82 | 7.1 |
| Turkey | 2003 | 33.1 | 27.1 | 39.7 | 402 | 56.2 | 52.2 | 60.1 | 797 | 58.4 | 52.8 | 63.9 | 407 | 52.3 | 19.8 | 11.5 | 31.9 | 102 | 22.2 | 16.3 | 29.6 | 212 | 18.5 | 10.1 | 31.3 | 99 | 20.8 |
| Turkey | 2013 | 60.0 | 52.4 | 67.2 | 215 | 61.8 | 56.6 | 66.8 | 477 | 67.0 | 62.5 | 71.1 | 709 | 64.4 | 23.6 | 13.9 | 37.1 | 59 | 28.3 | 19.0 | 39.8 | 104 | 33.9 | 25.2 | 43.9 | 169 | 30.7 |
| Turkmenistan | 2006 | 100.0 | - | - | 4 | 57.9 | 11.9 | 93.3 | 4 | 59.6 | 53.3 | 65.7 | 838 | 59.8 | 0.0 | - | - | 1 | 0.0 | - | - | 1 | 11.0 | 7.1 | 16.5 | 235 | 10.9 |
| Turkmenistan | 2015 | 100.0 | - | - | 1 | 100.0 | - | - | 1 | 73.3 | 70.2 | 76.3 | 1465 | 73.4 | 100.0 | - | - | 1 | 0.0 | - | - | 1 | 58.9 | 53.2 | 64.4 | 340 | 58.9 |
| Uganda | 1995 | 52.6 | 46.1 | 59.1 | 784 | 45.5 | 42.7 | 48.5 | 1729 | 48.7 | 42.8 | 54.6 | 409 | 48.1 | 70.3 | 63.0 | 76.6 | 165 | 53.9 | 47.6 | 60.0 | 375 | 39.3 | 26.8 | 53.5 | 89 | 57.4 |
| Uganda | 2000 | 31.5 | 27.4 | 36.0 | 659 | 29.5 | 26.7 | 32.4 | 1730 | 33.3 | 27.8 | 39.4 | 442 | 30.4 | 68.3 | 58.3 | 76.9 | 134 | 62.5 | 57.1 | 67.7 | 421 | 58.4 | 46.0 | 69.8 | 101 | 63.2 |
| Uganda | 2006 | 40.4 | 35.7 | 45.1 | 731 | 41.8 | 38.6 | 45.0 | 2037 | 43.5 | 38.1 | 49.0 | 471 | 41.7 | 60.8 | 52.3 | 68.7 | 176 | 60.1 | 55.2 | 64.9 | 483 | 58.8 | 50.2 | 66.9 | 141 | 60.1 |
| Uganda | 2011 | 52.5 | 46.5 | 58.4 | 495 | 51.4 | 48.0 | 54.8 | 1803 | 55.5 | 50.4 | 60.5 | 721 | 52.5 | 60.2 | 48.7 | 70.8 | 122 | 63.6 | 57.9 | 68.9 | 453 | 59.3 | 52.1 | 66.2 | 203 | 62.1 |
| Uganda | 2016 | 73.0 | 68.8 | 76.9 | 720 | 63.7 | 61.7 | 65.6 | 3719 | 68.7 | 65.6 | 71.6 | 1553 | 66.1 | 67.6 | 59.2 | 75.1 | 180 | 66.5 | 62.3 | 70.4 | 938 | 62.6 | 56.9 | 68.0 | 364 | 65.5 |
| Ukraine | 2005 | . | . | . | . | . | . | . | . | 35.9 | 29.4 | 42.9 | 1128 | 35.9 | . | . | . | . | . | . | . | . | 6.0 | 3.3 | 10.6 | 229 | 6.0 |
| Ukraine | 2007 | 100.0 | - | - | 1 | 0.0 | - | - | 3 | 41.6 | 36.3 | 47.2 | 434 | 41.5 | . | . | . | . | 0.0 | - | - | 1 | 18.4 | 11.5 | 28.1 | 97 | 18.2 |
| Ukraine | 2012 | . | . | . | . | . | . | . | . | 65.6 | 61.5 | 69.4 | 1562 | 65.7 | . | . | . | . | . | . | . | . | 19.7 | 13.8 | 27.3 | 307 | 19.7 |
| Uzbekistan | 1996 | . | . | . | . | 100.0 | - | - | 1 | 19.6 | 15.5 | 24.4 | 840 | 19.6 | . | . | . | . | . | . | . | . | 2.4 | 0.9 | 6.4 | 164 | 2.4 |
| Uzbekistan | 2006 | . | . | . | . | . | . | . | . | 67.1 | 63.7 | 70.3 | 2093 | 67.1 | . | . | . | . | . | . | . | . | 26.7 | 21.9 | 32.1 | 445 | 26.7 |
| Vietnam | 1997 | 16.4 | 10.8 | 24.0 | 96 | 20.7 | 14.0 | 29.5 | 302 | 32.6 | 28.6 | 36.9 | 734 | 27.9 | 13.1 | 3.3 | 40.2 | 27 | 16.4 | 7.1 | 33.7 | 65 | 15.8 | 9.9 | 24.4 | 145 | 15.7 |
| Vietnam | 2002 | 52.1 | 32.7 | 70.9 | 55 | 42.9 | 35.1 | 51.1 | 229 | 58.1 | 53.0 | 63.1 | 555 | 53.7 | 0.0 | - | - | 10 | 14.8 | 7.0 | 28.5 | 60 | 16.6 | 9.9 | 26.4 | 121 | 15.5 |
| Vietnam | 2006 | 39.5 | 29.8 | 50.0 | 185 | 36.0 | 30.9 | 41.4 | 446 | 41.8 | 35.8 | 48.0 | 392 | 38.9 | 30.9 | 15.0 | 53.0 | 34 | 12.0 | 6.2 | 21.9 | 88 | 17.9 | 10.5 | 28.7 | 107 | 16.9 |
| Vietnam | 2010 | 55.9 | 39.0 | 71.4 | 90 | 38.5 | 29.8 | 48.1 | 204 | 38.9 | 35.1 | 42.9 | 1069 | 39.7 | 37.4 | 15.0 | 67.0 | 21 | 15.3 | 4.7 | 40.2 | 54 | 16.3 | 11.8 | 22.0 | 244 | 17.0 |
| Vietnam | 2013 | 39.8 | 28.3 | 52.5 | 89 | 27.5 | 20.9 | 35.3 | 199 | 25.6 | 22.9 | 28.5 | 1196 | 26.5 | 41.6 | 22.1 | 64.1 | 21 | 24.2 | 11.6 | 43.7 | 47 | 23.5 | 18.3 | 29.8 | 290 | 24.3 |
| Zambia | 1996 | 65.6 | 60.8 | 70.1 | 413 | 55.7 | 53.1 | 58.2 | 1887 | 54.3 | 49.8 | 58.8 | 571 | 56.7 | 9.7 | 4.5 | 19.7 | 93 | 18.0 | 14.8 | 21.8 | 450 | 26.3 | 18.7 | 35.6 | 132 | 18.8 |
| Zambia | 2001 | 50.7 | 46.2 | 55.2 | 429 | 46.1 | 43.5 | 48.8 | 1771 | 57.7 | 53.1 | 62.2 | 569 | 49.4 | 32.7 | 23.8 | 43.0 | 113 | 37.5 | 32.3 | 43.0 | 413 | 53.8 | 44.7 | 62.6 | 124 | 40.1 |
| Zambia | 2007 | 58.3 | 52.8 | 63.5 | 347 | 55.1 | 52.5 | 57.7 | 1614 | 55.1 | 50.5 | 59.6 | 654 | 55.6 | 51.7 | 41.9 | 61.3 | 89 | 59.2 | 53.7 | 64.4 | 373 | 70.2 | 62.2 | 77.2 | 156 | 60.9 |
| Zambia | 2013 | 58.5 | 53.7 | 63.2 | 544 | 64.6 | 62.1 | 67.0 | 2720 | 65.6 | 62.6 | 68.6 | 1826 | 64.3 | 68.0 | 58.5 | 76.2 | 134 | 72.2 | 68.0 | 76.0 | 619 | 72.9 | 67.9 | 77.4 | 433 | 71.9 |
| Zambia | 2018 | 73.8 | 67.7 | 79.1 | 390 | 77.0 | 74.4 | 79.5 | 2040 | 74.0 | 70.1 | 77.5 | 1528 | 75.5 | 72.2 | 61.5 | 80.9 | 95 | 70.5 | 65.7 | 74.8 | 515 | 68.5 | 63.2 | 73.4 | 409 | 69.9 |
| Zimbabwe | 1994 | 43.9 | 37.1 | 50.9 | 203 | 38.9 | 34.9 | 43.0 | 782 | 40.0 | 35.2 | 45.0 | 569 | 40.0 | 15.5 | 7.1 | 30.6 | 39 | 9.8 | 6.1 | 15.3 | 194 | 12.0 | 7.0 | 19.9 | 167 | 11.3 |
| Zimbabwe | 1999 | 56.3 | 45.7 | 66.4 | 98 | 59.5 | 54.8 | 64.0 | 665 | 61.7 | 57.6 | 65.6 | 699 | 60.4 | 44.5 | 21.8 | 69.7 | 22 | 28.5 | 21.6 | 36.7 | 151 | 33.3 | 25.2 | 42.4 | 174 | 31.9 |
| Zimbabwe | 2005 | 67.5 | 41.6 | 85.8 | 60 | 66.3 | 61.5 | 70.9 | 805 | 69.1 | 66.0 | 72.1 | 1267 | 68.1 | 4.5 | 0.6 | 27.4 | 15 | 22.5 | 16.7 | 29.5 | 207 | 22.7 | 18.2 | 28.0 | 312 | 22.2 |
| Zimbabwe | 2009 | 44.4 | 31.6 | 58.1 | 61 | 50.0 | 46.4 | 53.6 | 984 | 52.0 | 49.1 | 54.8 | 1805 | 51.2 | 28.2 | 9.2 | 60.5 | 20 | 21.2 | 15.9 | 27.6 | 212 | 28.1 | 23.3 | 33.4 | 449 | 25.9 |
| Zimbabwe | 2010 | 61.9 | 43.5 | 77.4 | 33 | 68.7 | 64.3 | 72.9 | 777 | 63.6 | 60.6 | 66.5 | 1627 | 65.2 | 47.6 | 18.7 | 78.2 | 9 | 29.1 | 22.5 | 36.7 | 208 | 32.3 | 27.3 | 37.8 | 424 | 31.4 |
| Zimbabwe | 2014 | 53.7 | 39.2 | 67.7 | 44 | 57.5 | 54.0 | 61.0 | 1188 | 59.5 | 57.4 | 61.7 | 2681 | 58.9 | 39.9 | 8.2 | 83.1 | 5 | 34.8 | 29.0 | 41.2 | 277 | 44.1 | 39.3 | 48.9 | 585 | 41.0 |
| Zimbabwe | 2015 | 55.5 | 38.0 | 71.7 | 24 | 59.8 | 54.5 | 64.9 | 698 | 56.6 | 53.5 | 59.7 | 1645 | 57.6 | 56.5 | 17.4 | 88.9 | 5 | 53.1 | 45.0 | 61.0 | 158 | 45.4 | 40.1 | 50.9 | 440 | 47.7 |
| Zimbabwe | 2019 | 65.7 | 39.1 | 85.1 | 18 | 60.4 | 56.1 | 64.6 | 617 | 58.8 | 56.1 | 61.5 | 1703 | 59.3 | 51.0 | 8.2 | 92.4 | 3 | 36.3 | 28.9 | 44.5 | 165 | 43.9 | 38.7 | 49.3 | 437 | 41.9 |

^1^CAR: Central African Republic; ^2^CDR: Congo Democratic Republic.

**Supplementary table 3.** Percentage of children at 1 and 2 years of age who were fed breastmilk by mother’s formal education level. Source: DHS and MICS.

| Country | Year | Continued breastfeeding at 1 year | | | | | | | | | | | | National prevalence (%) | Continued breastfeeding at 2 years | | | | | | | | | | | | National prevalence (%) |
| --- | --- | --- | --- | --- | --- | --- | --- | --- | --- | --- | --- | --- | --- | --- | --- | --- | --- | --- | --- | --- | --- | --- | --- | --- | --- | --- | --- |
|  |  | None | | | | Primary | | | | Secondary or higher | | | |  | None | | | | Primary | | | | Secondary or higher | | | |  |
|  |  | % | 95% CI | | N | % | 95% CI | | N | % | 95% CI | | N |  | % | 95% CI | | N | % | 95% CI | | N | % | 95% CI | | N |  |
| Afghanistan | 2010 | 88.8 | 86.0 | 91.0 | 901 | 70.5 | 55.4 | 82.1 | 64 | 87.0 | 77.0 | 93.1 | 68 | 87.8 | 70.2 | 64.7 | 75.1 | 492 | 61.3 | 41.2 | 78.2 | 30 | 62.8 | 44.0 | 78.3 | 34 | 69.4 |
| Afghanistan | 2015 | 77.1 | 73.2 | 80.6 | 2109 | 85.2 | 72.9 | 92.5 | 158 | 82.1 | 71.7 | 89.3 | 200 | 78.4 | 58.5 | 53.1 | 63.7 | 808 | 64.7 | 46.5 | 79.5 | 72 | 54.4 | 35.2 | 72.4 | 84 | 58.6 |
| Albania | 2005 | 100.0 | - | - | 2 | 100.0 | - | - | 1 | 56.3 | 44.1 | 67.7 | 77 | 57.8 | . | . | . | . | 0.0 | - | - | 2 | 20.8 | 11.2 | 35.5 | 56 | 20.0 |
| Albania | 2008 | 82.0 | 24.8 | 98.4 | 3 | 67.1 | 49.3 | 81.0 | 46 | 50.5 | 32.2 | 68.6 | 45 | 60.6 | 0.0 | - | - | 1 | 26.2 | 10.3 | 52.4 | 43 | 40.5 | 17.2 | 69.0 | 30 | 31.0 |
| Albania | 2017 | 0.0 | - | - | 1 | 73.4 | 59.4 | 83.9 | 99 | 48.8 | 35.7 | 62.1 | 88 | 58.4 | . | . | . | . | 34.8 | 24.6 | 46.7 | 90 | 24.2 | 15.3 | 35.9 | 83 | 29.7 |
| Armenia | 2000 | . | . | . | . | . | . | . | . | 31.2 | 21.3 | 43.2 | 101 | 31.2 | . | . | . | . | . | . | . | . | 14.3 | 8.4 | 23.4 | 92 | 14.3 |
| Armenia | 2005 | . | . | . | . | . | . | . | . | 40.4 | 25.1 | 57.9 | 88 | 40.4 | . | . | . | . | 0.0 | - | - | 1 | 15.9 | 7.8 | 29.7 | 81 | 14.9 |
| Armenia | 2010 | . | . | . | . | . | . | . | . | 44.2 | 33.8 | 55.1 | 111 | 44.2 | . | . | . | . | . | . | . | . | 22.8 | 14.3 | 34.3 | 80 | 22.8 |
| Armenia | 2015 | . | . | . | . | 100.0 | - | - | 1 | 35.5 | 26.9 | 45.1 | 117 | 36.0 | . | . | . | . | . | . | . | . | 21.6 | 13.2 | 33.3 | 86 | 21.6 |
| Bangladesh | 1993 | 98.5 | 96.0 | 99.5 | 286 | 94.0 | 87.3 | 97.3 | 119 | 89.6 | 80.0 | 94.9 | 93 | 95.9 | 92.6 | 87.3 | 95.8 | 167 | 93.5 | 85.9 | 97.2 | 92 | 77.7 | 62.2 | 88.1 | 45 | 90.8 |
| Bangladesh | 1996 | 98.6 | 95.5 | 99.6 | 252 | 97.7 | 94.2 | 99.1 | 119 | 92.8 | 83.8 | 97.0 | 60 | 97.6 | 95.6 | 90.4 | 98.0 | 166 | 95.9 | 87.2 | 98.8 | 78 | 83.5 | 71.0 | 91.3 | 52 | 93.6 |
| Bangladesh | 1999 | 98.2 | 94.6 | 99.4 | 213 | 97.0 | 92.8 | 98.8 | 155 | 90.9 | 84.5 | 94.7 | 150 | 95.9 | 92.5 | 87.2 | 95.7 | 174 | 88.6 | 80.6 | 93.6 | 101 | 86.2 | 77.7 | 91.8 | 92 | 89.9 |
| Bangladesh | 2004 | 99.1 | 95.7 | 99.8 | 158 | 96.7 | 91.0 | 98.8 | 146 | 93.5 | 86.9 | 96.9 | 170 | 96.4 | 95.5 | 88.3 | 98.4 | 100 | 94.9 | 87.7 | 98.0 | 88 | 91.8 | 85.1 | 95.7 | 122 | 94.0 |
| Bangladesh | 2006 | 96.8 | 94.3 | 98.3 | 560 | 97.5 | 95.6 | 98.6 | 542 | 92.8 | 89.6 | 95.0 | 690 | 95.4 | 89.4 | 86.3 | 91.9 | 736 | 91.2 | 88.5 | 93.3 | 664 | 87.5 | 84.6 | 89.9 | 901 | 89.2 |
| Bangladesh | 2007 | 97.5 | 83.8 | 99.7 | 76 | 95.4 | 85.2 | 98.7 | 102 | 92.6 | 85.5 | 96.4 | 158 | 94.5 | 91.7 | 79.2 | 97.0 | 77 | 96.9 | 92.2 | 98.8 | 103 | 87.2 | 79.8 | 92.1 | 174 | 91.0 |
| Bangladesh | 2011 | 96.1 | 86.7 | 99.0 | 81 | 95.4 | 89.4 | 98.1 | 160 | 94.5 | 90.1 | 97.0 | 306 | 95.0 | 92.7 | 81.7 | 97.3 | 71 | 93.6 | 88.6 | 96.6 | 146 | 86.5 | 81.3 | 90.5 | 260 | 89.6 |
| Bangladesh | 2012 | 96.9 | 94.4 | 98.3 | 325 | 97.5 | 95.4 | 98.6 | 437 | 93.7 | 91.2 | 95.5 | 791 | 95.3 | 87.8 | 82.2 | 91.9 | 272 | 88.3 | 83.9 | 91.7 | 331 | 87.0 | 82.3 | 90.5 | 526 | 87.5 |
| Bangladesh | 2014 | 100.0 | - | - | 59 | 94.3 | 84.2 | 98.1 | 157 | 95.9 | 92.4 | 97.8 | 336 | 96.0 | 90.2 | 80.1 | 95.5 | 71 | 90.8 | 83.7 | 95.0 | 136 | 85.1 | 79.0 | 89.6 | 279 | 87.3 |
| Bangladesh | 2019 | 87.2 | 79.3 | 92.4 | 137 | 93.4 | 89.3 | 95.9 | 373 | 93.7 | 91.7 | 95.2 | 1028 | 93.0 | 80.0 | 71.4 | 86.5 | 148 | 85.4 | 80.1 | 89.5 | 294 | 84.5 | 81.4 | 87.1 | 864 | 84.2 |
| Belize | 2006 | 62.5 | 11.4 | 95.6 | 3 | 45.0 | 28.9 | 62.3 | 28 | 18.8 | 2.6 | 66.8 | 7 | 41.6 | 0.0 | - | - | 1 | 27.7 | 12.8 | 49.9 | 26 | 27.6 | 11.9 | 52.0 | 22 | 26.8 |
| Belize | 2011 | 67.8 | 17.7 | 95.4 | 6 | 65.7 | 50.5 | 78.2 | 57 | 60.7 | 43.0 | 76.0 | 45 | 62.1 | 54.2 | 20.9 | 84.1 | 9 | 38.2 | 26.8 | 51.0 | 72 | 30.3 | 22.0 | 40.3 | 72 | 34.9 |
| Belize | 2015 | 54.7 | 15.2 | 89.1 | 7 | 64.1 | 51.4 | 75.0 | 96 | 39.9 | 29.6 | 51.2 | 103 | 51.5 | 79.3 | 27.0 | 97.5 | 5 | 48.7 | 32.8 | 65.0 | 48 | 27.0 | 18.4 | 37.9 | 93 | 35.1 |
| Benin | 1996 | 98.4 | 95.6 | 99.4 | 273 | 95.4 | 82.4 | 98.9 | 49 | 91.0 | 51.0 | 99.0 | 12 | 97.6 | 71.4 | 64.6 | 77.3 | 202 | 53.0 | 38.0 | 67.4 | 38 | 24.4 | 7.1 | 57.6 | 11 | 66.1 |
| Benin | 2001 | 99.0 | 96.2 | 99.8 | 227 | 91.3 | 80.4 | 96.4 | 61 | 83.2 | 63.4 | 93.4 | 24 | 96.2 | 70.6 | 63.8 | 76.6 | 215 | 36.4 | 23.3 | 51.8 | 49 | 43.6 | 24.8 | 64.4 | 24 | 62.4 |
| Benin | 2006 | 96.4 | 94.8 | 97.4 | 846 | 96.2 | 92.2 | 98.2 | 184 | 91.4 | 79.1 | 96.7 | 56 | 96.0 | 63.1 | 58.7 | 67.2 | 600 | 49.7 | 41.8 | 57.5 | 161 | 23.7 | 13.9 | 37.4 | 59 | 57.3 |
| Benin | 2011 | 81.5 | 77.8 | 84.7 | 602 | 84.3 | 73.8 | 91.1 | 122 | 82.3 | 72.6 | 89.1 | 98 | 82.1 | 58.4 | 54.3 | 62.4 | 585 | 44.8 | 34.5 | 55.6 | 117 | 30.2 | 21.8 | 40.2 | 90 | 52.6 |
| Benin | 2014 | 95.9 | 93.5 | 97.4 | 493 | 96.7 | 92.5 | 98.6 | 195 | 93.6 | 87.5 | 96.8 | 105 | 95.8 | 54.2 | 49.0 | 59.3 | 443 | 33.0 | 24.7 | 42.4 | 162 | 30.6 | 21.2 | 41.9 | 97 | 45.5 |
| Benin | 2017 | 91.7 | 88.8 | 93.9 | 479 | 92.7 | 86.9 | 96.1 | 156 | 88.0 | 79.9 | 93.1 | 129 | 91.2 | 49.6 | 44.8 | 54.5 | 546 | 34.3 | 26.4 | 43.2 | 136 | 21.3 | 15.0 | 29.3 | 139 | 42.4 |
| Bolivia | 1994 | 93.4 | 81.4 | 97.9 | 57 | 78.1 | 70.6 | 84.1 | 160 | 63.9 | 53.9 | 72.8 | 126 | 75.1 | 52.8 | 36.6 | 68.5 | 43 | 45.3 | 36.6 | 54.3 | 137 | 32.8 | 24.8 | 42.1 | 140 | 40.6 |
| Bolivia | 1998 | 87.2 | 72.4 | 94.6 | 51 | 82.8 | 75.3 | 88.5 | 215 | 69.1 | 61.1 | 76.2 | 174 | 77.5 | 61.8 | 46.9 | 74.9 | 52 | 40.7 | 32.5 | 49.4 | 177 | 25.3 | 19.1 | 32.7 | 156 | 36.0 |
| Bolivia | 2003 | 90.3 | 73.6 | 96.9 | 48 | 86.8 | 82.1 | 90.4 | 405 | 68.0 | 60.6 | 74.6 | 211 | 81.5 | 50.2 | 34.5 | 65.8 | 48 | 47.2 | 40.1 | 54.4 | 313 | 42.4 | 33.0 | 52.5 | 180 | 45.8 |
| Bolivia | 2008 | 97.1 | 81.2 | 99.6 | 21 | 91.3 | 86.8 | 94.4 | 293 | 75.4 | 68.0 | 81.6 | 242 | 84.6 | 53.9 | 34.2 | 72.5 | 38 | 39.3 | 31.8 | 47.5 | 275 | 37.6 | 29.7 | 46.3 | 215 | 39.8 |
| Bosnia and Herzegovina | 2006 | 0.0 | - | - | 1 | 38.1 | 24.9 | 53.3 | 57 | 20.6 | 15.1 | 27.5 | 143 | 25.6 | 50.0 | 5.7 | 94.3 | 2 | 18.4 | 10.1 | 31.3 | 53 | 6.7 | 4.1 | 10.9 | 193 | 9.6 |
| Bosnia and Herzegovina | 2011 | 0.0 | - | - | 1 | 18.8 | 7.8 | 38.9 | 25 | 11.4 | 7.2 | 17.4 | 130 | 12.4 | 0.0 | - | - | 1 | 19.1 | 9.3 | 35.4 | 40 | 10.4 | 6.4 | 16.5 | 145 | 12.2 |
| Burkina Faso | 1998 | 98.7 | 96.9 | 99.5 | 326 | 96.7 | 79.1 | 99.6 | 26 | 91.9 | 72.0 | 98.1 | 21 | 98.4 | 90.1 | 83.7 | 94.1 | 246 | 73.8 | 48.9 | 89.2 | 17 | 41.8 | 16.5 | 72.3 | 11 | 87.9 |
| Burkina Faso | 2003 | 98.5 | 96.0 | 99.4 | 568 | 93.5 | 70.8 | 98.9 | 68 | 100.0 | - | - | 23 | 98.1 | 83.9 | 79.6 | 87.4 | 426 | 67.8 | 52.1 | 80.4 | 47 | 47.4 | 20.4 | 76.1 | 20 | 81.0 |
| Burkina Faso | 2006 | 95.7 | 93.1 | 97.4 | 415 | 98.5 | 86.6 | 99.8 | 36 | 93.3 | 63.6 | 99.1 | 15 | 96.1 | 87.1 | 80.2 | 91.8 | 233 | 78.2 | 55.7 | 91.1 | 31 | 69.6 | 25.4 | 93.9 | 7 | 85.4 |
| Burkina Faso | 2010 | 97.5 | 96.1 | 98.4 | 814 | 93.7 | 85.8 | 97.3 | 120 | 93.0 | 75.9 | 98.3 | 52 | 96.8 | 84.1 | 81.0 | 86.7 | 668 | 65.1 | 53.7 | 75.0 | 95 | 52.3 | 36.0 | 68.2 | 57 | 80.1 |
| Burundi | 2010 | 94.0 | 89.6 | 96.7 | 252 | 95.4 | 91.1 | 97.7 | 190 | 89.9 | 78.8 | 95.5 | 51 | 94.3 | 78.9 | 73.1 | 83.8 | 228 | 84.2 | 78.3 | 88.8 | 186 | 50.3 | 32.5 | 68.0 | 41 | 79.1 |
| Burundi | 2016 | 97.2 | 94.5 | 98.6 | 346 | 94.7 | 91.8 | 96.6 | 418 | 89.2 | 80.3 | 94.3 | 130 | 95.0 | 83.0 | 77.7 | 87.2 | 322 | 80.9 | 76.1 | 84.9 | 333 | 76.3 | 65.6 | 84.5 | 94 | 81.4 |
| CAR^1^ | 1994 | 97.9 | 93.4 | 99.3 | 129 | 97.8 | 93.0 | 99.3 | 120 | 92.2 | 83.3 | 96.5 | 50 | 96.8 | 70.1 | 62.2 | 77.0 | 121 | 47.9 | 36.6 | 59.4 | 72 | 32.8 | 19.0 | 50.4 | 27 | 57.6 |
| CAR^1^ | 2006 | 88.1 | 81.6 | 92.5 | 320 | 87.2 | 79.9 | 92.1 | 342 | 76.3 | 61.5 | 86.6 | 101 | 85.9 | 63.7 | 55.4 | 71.2 | 167 | 46.0 | 35.9 | 56.6 | 179 | 22.3 | 13.6 | 34.3 | 62 | 47.1 |
| CAR^1^ | 2010 | 89.4 | 84.3 | 93.0 | 356 | 89.2 | 84.5 | 92.6 | 336 | 90.6 | 80.5 | 95.8 | 76 | 89.5 | 42.9 | 35.8 | 50.4 | 255 | 25.1 | 18.5 | 33.2 | 285 | 24.7 | 15.3 | 37.3 | 77 | 32.1 |
| Cambodia | 2000 | 91.8 | 84.3 | 95.9 | 180 | 88.3 | 82.7 | 92.3 | 267 | 79.5 | 64.9 | 89.0 | 54 | 88.1 | 65.9 | 56.3 | 74.3 | 141 | 54.7 | 46.3 | 62.8 | 194 | 54.9 | 39.0 | 69.9 | 46 | 58.5 |
| Cambodia | 2005 | 93.1 | 85.5 | 96.9 | 146 | 90.2 | 84.7 | 93.8 | 315 | 84.6 | 74.4 | 91.2 | 81 | 89.9 | 60.3 | 48.3 | 71.1 | 127 | 56.1 | 48.4 | 63.5 | 292 | 39.0 | 27.6 | 51.7 | 73 | 54.2 |
| Cambodia | 2010 | 89.6 | 81.0 | 94.6 | 106 | 86.2 | 80.7 | 90.3 | 296 | 73.1 | 63.5 | 80.9 | 165 | 83.3 | 65.0 | 51.5 | 76.5 | 80 | 43.5 | 36.4 | 50.8 | 266 | 31.7 | 22.5 | 42.5 | 139 | 43.4 |
| Cambodia | 2014 | 80.8 | 65.5 | 90.3 | 65 | 87.9 | 81.1 | 92.4 | 224 | 67.0 | 57.6 | 75.2 | 161 | 80.0 | 46.8 | 30.1 | 64.3 | 68 | 38.8 | 30.8 | 47.4 | 222 | 31.0 | 23.1 | 40.1 | 196 | 37.1 |
| Cameroon | 1998 | 96.2 | 89.2 | 98.7 | 78 | 87.5 | 78.3 | 93.2 | 85 | 76.2 | 62.3 | 86.1 | 71 | 87.8 | 64.0 | 48.0 | 77.4 | 54 | 26.9 | 18.3 | 37.8 | 83 | 6.1 | 2.0 | 17.7 | 62 | 32.4 |
| Cameroon | 2004 | 89.6 | 82.9 | 93.9 | 147 | 86.6 | 81.1 | 90.6 | 228 | 68.5 | 60.4 | 75.7 | 142 | 83.1 | 54.1 | 44.0 | 63.8 | 99 | 27.8 | 21.5 | 35.2 | 198 | 4.8 | 2.1 | 10.5 | 119 | 28.5 |
| Cameroon | 2006 | 90.6 | 83.0 | 95.1 | 169 | 84.5 | 77.8 | 89.5 | 203 | 54.8 | 45.1 | 64.1 | 147 | 78.7 | 47.0 | 35.3 | 59.1 | 77 | 20.2 | 13.4 | 29.3 | 174 | 6.5 | 2.5 | 15.7 | 124 | 21.2 |
| Cameroon | 2011 | 93.7 | 88.9 | 96.6 | 182 | 87.5 | 83.1 | 90.9 | 340 | 53.9 | 47.0 | 60.5 | 274 | 77.9 | 52.9 | 42.9 | 62.7 | 134 | 22.3 | 16.5 | 29.4 | 235 | 6.5 | 3.6 | 11.4 | 208 | 24.3 |
| Cameroon | 2014 | 86.5 | 76.3 | 92.7 | 114 | 76.5 | 68.5 | 82.9 | 173 | 52.6 | 44.2 | 60.9 | 206 | 70.3 | 36.8 | 26.8 | 48.0 | 85 | 16.7 | 10.9 | 24.6 | 176 | 7.0 | 3.7 | 12.7 | 175 | 18.5 |
| Cameroon | 2018 | 80.8 | 72.0 | 87.3 | 167 | 72.7 | 65.9 | 78.7 | 218 | 43.2 | 35.9 | 50.9 | 255 | 64.5 | 45.3 | 35.7 | 55.3 | 104 | 21.9 | 15.2 | 30.5 | 155 | 8.5 | 4.9 | 14.5 | 193 | 23.2 |
| Chad | 1996 | 93.4 | 89.8 | 95.8 | 375 | 91.7 | 86.5 | 95.0 | 114 | 88.1 | 66.8 | 96.5 | 21 | 93.0 | 69.6 | 61.4 | 76.8 | 196 | 51.1 | 37.6 | 64.3 | 62 | 37.7 | 15.0 | 67.5 | 14 | 65.0 |
| Chad | 2004 | 92.8 | 88.6 | 95.6 | 298 | 85.9 | 68.6 | 94.4 | 65 | 89.0 | 72.7 | 96.1 | 31 | 91.5 | 71.3 | 57.0 | 82.3 | 125 | 61.3 | 45.3 | 75.1 | 49 | 29.7 | 13.0 | 54.4 | 23 | 65.8 |
| Chad | 2010 | 85.8 | 82.1 | 88.8 | 837 | 91.9 | 86.1 | 95.4 | 204 | 89.9 | 81.3 | 94.8 | 100 | 87.2 | 68.5 | 63.9 | 72.8 | 698 | 53.0 | 43.3 | 62.4 | 159 | 32.5 | 20.4 | 47.3 | 83 | 63.0 |
| Chad | 2014 | 86.8 | 83.8 | 89.4 | 786 | 90.6 | 84.6 | 94.5 | 189 | 89.0 | 81.3 | 93.8 | 119 | 87.9 | 70.6 | 65.4 | 75.4 | 476 | 65.6 | 56.3 | 73.8 | 168 | 33.8 | 22.9 | 46.8 | 69 | 65.2 |
| Colombia | 1995 | 46.7 | 23.6 | 71.2 | 16 | 50.2 | 41.7 | 58.7 | 129 | 37.9 | 29.3 | 47.3 | 177 | 43.2 | 10.2 | 1.3 | 49.2 | 9 | 22.4 | 16.1 | 30.3 | 148 | 16.6 | 11.4 | 23.4 | 148 | 19.2 |
| Colombia | 2000 | 34.7 | 13.8 | 63.9 | 13 | 67.7 | 55.7 | 77.7 | 101 | 40.2 | 31.9 | 49.1 | 162 | 50.0 | 27.3 | 8.2 | 61.4 | 10 | 26.7 | 18.4 | 37.2 | 101 | 25.3 | 18.4 | 33.8 | 148 | 25.9 |
| Colombia | 2005 | 69.2 | 47.5 | 84.7 | 32 | 68.4 | 60.9 | 75.1 | 307 | 51.3 | 45.7 | 56.7 | 587 | 57.3 | 38.6 | 21.9 | 58.6 | 35 | 34.4 | 27.6 | 42.0 | 274 | 30.7 | 25.6 | 36.3 | 536 | 32.2 |
| Colombia | 2010 | 82.3 | 60.1 | 93.5 | 29 | 69.4 | 62.2 | 75.8 | 315 | 54.1 | 49.4 | 58.7 | 745 | 58.5 | 40.3 | 22.5 | 61.1 | 24 | 43.0 | 35.9 | 50.5 | 308 | 28.9 | 25.0 | 33.2 | 729 | 32.5 |
| Comoros | 1996 | 83.9 | 69.8 | 92.2 | 87 | 81.3 | 61.5 | 92.2 | 32 | 76.5 | 60.2 | 87.5 | 17 | 82.4 | 53.8 | 38.1 | 68.8 | 52 | 61.5 | 35.5 | 82.3 | 13 | 57.1 | 32.7 | 78.5 | 14 | 55.7 |
| Comoros | 2012 | 66.3 | 52.5 | 77.7 | 86 | 63.6 | 46.4 | 77.8 | 52 | 77.7 | 65.3 | 86.5 | 72 | 69.7 | 70.5 | 54.6 | 82.6 | 49 | 32.9 | 18.4 | 51.6 | 42 | 60.3 | 43.2 | 75.2 | 50 | 56.7 |
| CDR^2^ | 2007 | 88.9 | 78.8 | 94.6 | 152 | 95.5 | 91.5 | 97.6 | 293 | 85.9 | 76.9 | 91.7 | 186 | 90.8 | 73.9 | 57.7 | 85.4 | 81 | 66.8 | 57.8 | 74.7 | 179 | 55.2 | 45.1 | 64.9 | 154 | 63.5 |
| CDR^2^ | 2010 | 90.5 | 84.4 | 94.4 | 213 | 85.0 | 80.0 | 89.0 | 352 | 85.3 | 78.9 | 90.0 | 281 | 86.6 | 68.4 | 59.7 | 75.9 | 158 | 53.7 | 47.2 | 60.1 | 302 | 42.6 | 35.5 | 50.0 | 303 | 52.5 |
| CDR^2^ | 2013 | 94.5 | 90.2 | 97.0 | 252 | 92.2 | 89.2 | 94.4 | 603 | 90.6 | 86.5 | 93.6 | 439 | 92.0 | 82.2 | 73.8 | 88.4 | 185 | 67.7 | 60.1 | 74.5 | 358 | 56.5 | 48.4 | 64.3 | 320 | 66.3 |
| CDR^2^ | 2017 | 87.6 | 81.4 | 91.9 | 398 | 88.8 | 83.6 | 92.5 | 618 | 88.0 | 83.5 | 91.4 | 590 | 88.2 | 72.4 | 63.1 | 80.0 | 250 | 54.9 | 48.1 | 61.5 | 495 | 40.3 | 33.5 | 47.6 | 531 | 50.1 |
| Cote d'Ivoire | 1994 | 98.1 | 95.0 | 99.3 | 244 | 92.9 | 85.0 | 96.8 | 97 | 75.7 | 57.4 | 87.9 | 26 | 95.1 | 58.4 | 50.9 | 65.7 | 238 | 35.5 | 24.9 | 47.8 | 65 | 7.4 | 2.2 | 22.5 | 31 | 49.0 |
| Cote d'Ivoire | 1998 | 97.0 | 89.5 | 99.2 | 89 | 98.4 | 88.1 | 99.8 | 35 | 53.2 | 30.0 | 75.1 | 16 | 94.1 | 74.1 | 59.9 | 84.5 | 74 | 27.8 | 11.6 | 53.0 | 25 | 6.5 | 0.8 | 37.6 | 13 | 58.0 |
| Cote d'Ivoire | 2006 | 90.3 | 85.7 | 93.6 | 364 | 86.5 | 78.7 | 91.7 | 160 | 70.7 | 53.6 | 83.4 | 55 | 87.3 | 42.9 | 35.4 | 50.7 | 331 | 30.4 | 22.6 | 39.6 | 149 | 25.3 | 14.0 | 41.5 | 48 | 37.4 |
| Cote d'Ivoire | 2011 | 90.6 | 85.2 | 94.1 | 300 | 80.9 | 71.6 | 87.8 | 127 | 70.7 | 52.4 | 84.0 | 44 | 86.0 | 44.1 | 37.6 | 50.9 | 297 | 30.3 | 19.5 | 43.9 | 97 | 18.3 | 8.5 | 35.0 | 49 | 38.1 |
| Cote d'Ivoire | 2016 | 92.6 | 89.1 | 95.0 | 422 | 87.4 | 75.8 | 93.8 | 146 | 76.0 | 63.4 | 85.4 | 89 | 88.1 | 36.8 | 31.0 | 43.1 | 352 | 18.5 | 12.1 | 27.2 | 120 | 15.3 | 8.5 | 26.0 | 66 | 29.0 |
| Dominican Republic | 1996 | 60.8 | 42.5 | 76.5 | 39 | 37.0 | 29.5 | 45.1 | 171 | 18.3 | 10.8 | 29.2 | 100 | 31.9 | 26.1 | 10.2 | 52.2 | 33 | 9.1 | 5.0 | 16.2 | 126 | 5.7 | 2.0 | 15.1 | 79 | 9.6 |
| Dominican Republic | 1999 | 0.0 | - | - | 1 | 48.9 | . | . | 11 | 14.8 | . | . | 9 | 27.6 | . | . | . | . | 21.0 | . | . | 16 | 20.3 | . | . | 9 | 20.7 |
| Dominican Republic | 2002 | 68.8 | 48.0 | 84.1 | 38 | 33.4 | 26.7 | 41.0 | 340 | 23.9 | 17.6 | 31.6 | 320 | 29.9 | 39.4 | 21.0 | 61.4 | 37 | 18.0 | 13.3 | 23.9 | 361 | 11.4 | 7.3 | 17.3 | 278 | 15.6 |
| Dominican Republic | 2007 | 49.6 | 26.6 | 72.8 | 31 | 35.4 | 26.1 | 46.0 | 206 | 32.0 | 24.2 | 40.9 | 293 | 33.6 | 18.9 | 6.9 | 42.3 | 30 | 16.6 | 11.4 | 23.6 | 269 | 8.8 | 5.7 | 13.4 | 335 | 12.0 |
| Dominican Republic | 2013 | 60.9 | 23.2 | 88.9 | 10 | 48.3 | 33.4 | 63.4 | 75 | 28.9 | 20.8 | 38.5 | 168 | 34.9 | 62.3 | 20.9 | 91.2 | 7 | 19.3 | 10.3 | 33.3 | 87 | 9.9 | 4.5 | 20.5 | 135 | 14.4 |
| Dominican Republic | 2014 | 58.1 | 39.4 | 74.7 | 46 | 38.7 | 30.0 | 48.2 | 323 | 26.3 | 21.8 | 31.5 | 712 | 31.2 | 14.7 | 6.3 | 30.6 | 54 | 14.9 | 9.1 | 23.3 | 482 | 11.2 | 8.4 | 14.8 | 976 | 12.4 |
| Egypt | 1995 | 85.8 | 80.5 | 89.9 | 325 | 89.8 | 82.0 | 94.5 | 156 | 70.9 | 63.8 | 77.1 | 279 | 80.8 | 56.2 | 48.9 | 63.3 | 314 | 54.9 | 46.1 | 63.4 | 150 | 25.7 | 19.1 | 33.6 | 202 | 46.0 |
| Egypt | 2000 | 87.8 | 83.6 | 91.1 | 307 | 84.0 | 75.4 | 90.0 | 114 | 77.7 | 72.2 | 82.4 | 323 | 83.0 | 43.5 | 36.4 | 50.8 | 231 | 31.6 | 21.7 | 43.6 | 86 | 28.4 | 22.2 | 35.6 | 262 | 35.0 |
| Egypt | 2005 | 91.7 | 35.6 | 99.5 | 257 | 85.1 | 7.4 | 99.8 | 96 | 83.6 | 44.5 | 97.0 | 498 | 85.9 | 47.3 | 10.5 | 87.2 | 244 | 41.9 | 3.3 | 93.8 | 101 | 25.5 | 6.7 | 62.2 | 466 | 33.7 |
| Egypt | 2008 | 89.1 | 83.0 | 93.2 | 186 | 84.6 | 71.9 | 92.1 | 77 | 80.9 | 76.2 | 84.8 | 436 | 83.3 | 41.4 | 33.7 | 49.5 | 164 | 31.7 | 20.9 | 45.0 | 62 | 18.8 | 15.2 | 23.0 | 423 | 25.2 |
| Egypt | 2014 | 80.8 | 72.5 | 87.1 | 148 | 75.6 | 63.3 | 84.8 | 74 | 80.2 | 76.7 | 83.4 | 796 | 80.0 | 34.1 | 26.5 | 42.8 | 150 | 27.4 | 16.7 | 41.5 | 80 | 17.0 | 13.8 | 20.9 | 777 | 20.4 |
| Eswatini | 2006 | 84.1 | 59.6 | 95.0 | 21 | 83.4 | 71.7 | 90.9 | 73 | 73.4 | 61.8 | 82.5 | 99 | 78.4 | 60.4 | 29.8 | 84.6 | 16 | 32.6 | 20.8 | 47.3 | 57 | 22.3 | 13.6 | 34.3 | 75 | 30.7 |
| Eswatini | 2010 | 50.0 | 23.1 | 76.9 | 13 | 58.2 | 43.3 | 71.7 | 50 | 62.0 | 52.4 | 70.7 | 106 | 60.0 | 8.9 | 2.2 | 30.0 | 22 | 10.9 | 5.2 | 21.4 | 67 | 11.0 | 6.1 | 18.9 | 103 | 10.7 |
| Eswatini | 2014 | 48.3 | 12.9 | 85.5 | 6 | 43.8 | 29.3 | 59.5 | 62 | 49.5 | 39.5 | 59.6 | 123 | 47.8 | 0.0 | - | - | 18 | 9.0 | 3.7 | 20.6 | 52 | 7.8 | 3.8 | 15.4 | 101 | 7.6 |
| Ethiopia | 2011 | 96.4 | 93.5 | 98.0 | 491 | 96.6 | 91.8 | 98.6 | 149 | 95.7 | 86.6 | 98.7 | 36 | 96.4 | 89.3 | 84.0 | 93.0 | 304 | 65.6 | 51.9 | 77.2 | 139 | 83.4 | 71.1 | 91.1 | 32 | 82.2 |
| Ethiopia | 2016 | 90.1 | 85.8 | 93.3 | 472 | 95.8 | 91.0 | 98.1 | 200 | 90.2 | 79.5 | 95.6 | 83 | 91.8 | 76.7 | 69.0 | 83.0 | 264 | 74.8 | 65.1 | 82.6 | 132 | 69.5 | 49.2 | 84.2 | 66 | 75.5 |
| Gabon | 2000 | 36.2 | 15.0 | 64.7 | 16 | 52.0 | 43.6 | 60.4 | 147 | 41.0 | 32.1 | 50.5 | 147 | 45.1 | 17.6 | 5.6 | 43.5 | 13 | 16.6 | 8.6 | 29.6 | 90 | 2.3 | 0.7 | 7.5 | 77 | 9.4 |
| Gabon | 2012 | 89.4 | 58.4 | 98.1 | 22 | 64.0 | 46.9 | 78.2 | 147 | 34.9 | 25.6 | 45.6 | 212 | 45.4 | 2.3 | 0.5 | 9.9 | 24 | 10.8 | 5.5 | 20.0 | 145 | 1.7 | 0.8 | 3.8 | 187 | 3.9 |
| Gambia | 2005 | 94.2 | 91.0 | 96.3 | 375 | 84.0 | 72.8 | 91.2 | 69 | 88.1 | 80.4 | 93.1 | 102 | 92.3 | 52.8 | 46.4 | 59.1 | 265 | 57.4 | 43.9 | 69.9 | 51 | 36.4 | 23.1 | 52.3 | 44 | 53.2 |
| Gambia | 2010 | 93.1 | 89.6 | 95.4 | 609 | 99.3 | 95.1 | 99.9 | 70 | 87.7 | 67.4 | 96.1 | 94 | 92.9 | 34.0 | 28.9 | 39.6 | 594 | 33.6 | 13.4 | 62.4 | 45 | 18.3 | 11.5 | 27.8 | 116 | 30.5 |
| Gambia | 2013 | 98.1 | 95.3 | 99.3 | 388 | 100.0 | - | - | 98 | 96.1 | 89.8 | 98.6 | 154 | 97.8 | 49.1 | 41.2 | 56.9 | 271 | 28.5 | 17.4 | 42.9 | 67 | 33.4 | 21.8 | 47.4 | 74 | 42.2 |
| Gambia | 2018 | 98.3 | 94.7 | 99.5 | 417 | 98.5 | 95.2 | 99.6 | 114 | 92.6 | 85.6 | 96.3 | 192 | 96.4 | 41.0 | 33.8 | 48.7 | 284 | 43.5 | 30.9 | 57.0 | 100 | 32.7 | 22.3 | 45.0 | 104 | 39.0 |
| Georgia | 2005 | . | . | . | . | 0.0 | - | - | 2 | 41.2 | 32.4 | 50.6 | 135 | 40.5 | . | . | . | . | . | . | . | . | 19.6 | 12.3 | 29.6 | 112 | 19.6 |
| Georgia | 2018 | . | . | . | . | 64.3 | 27.4 | 89.6 | 12 | 29.0 | 19.9 | 40.2 | 149 | 31.5 | . | . | . | . | 48.9 | 21.4 | 77.1 | 18 | 19.7 | 13.3 | 28.1 | 145 | 22.8 |
| Ghana | 1993 | 97.6 | 90.9 | 99.4 | 84 | 96.3 | 90.3 | 98.7 | 109 | 60.0 | 25.2 | 87.0 | 10 | 95.1 | 73.0 | 61.9 | 81.8 | 74 | 47.1 | 36.9 | 57.5 | 104 | 33.3 | 12.6 | 63.5 | 12 | 56.3 |
| Ghana | 1998 | 98.5 | 89.8 | 99.8 | 102 | 97.3 | 83.2 | 99.6 | 42 | 96.0 | 88.4 | 98.7 | 80 | 97.2 | 79.5 | 66.9 | 88.2 | 81 | 56.4 | 39.7 | 71.8 | 36 | 42.9 | 31.6 | 55.0 | 72 | 59.2 |
| Ghana | 2003 | 97.1 | 81.3 | 99.6 | 110 | 95.1 | 84.2 | 98.6 | 57 | 95.2 | 88.5 | 98.1 | 107 | 95.8 | 87.3 | 78.0 | 93.0 | 98 | 61.9 | 43.5 | 77.4 | 40 | 43.7 | 30.4 | 58.0 | 59 | 66.7 |
| Ghana | 2006 | 94.9 | 87.1 | 98.1 | 114 | 95.0 | 84.0 | 98.6 | 53 | 93.9 | 84.1 | 97.8 | 72 | 94.6 | 73.3 | 61.2 | 82.7 | 106 | 58.0 | 41.8 | 72.6 | 34 | 40.9 | 29.6 | 53.3 | 84 | 56.1 |
| Ghana | 2008 | 98.0 | 92.1 | 99.5 | 82 | 90.2 | 67.9 | 97.5 | 38 | 93.4 | 85.2 | 97.2 | 83 | 94.5 | 59.6 | 42.0 | 75.0 | 54 | 43.6 | 28.2 | 60.3 | 41 | 31.0 | 18.7 | 46.8 | 49 | 43.9 |
| Ghana | 2011 | 96.4 | 90.7 | 98.6 | 241 | 93.7 | 81.4 | 98.0 | 95 | 86.3 | 78.2 | 91.7 | 153 | 90.7 | 64.9 | 53.6 | 74.7 | 235 | 38.1 | 24.4 | 54.0 | 85 | 17.4 | 11.8 | 24.9 | 135 | 37.4 |
| Ghana | 2014 | 98.0 | 92.4 | 99.5 | 120 | 95.1 | 83.8 | 98.6 | 69 | 92.9 | 86.2 | 96.5 | 173 | 94.6 | 68.3 | 57.9 | 77.1 | 129 | 60.9 | 46.8 | 73.4 | 72 | 34.7 | 25.9 | 44.6 | 136 | 50.1 |
| Ghana | 2017 | 93.2 | 82.7 | 97.5 | 137 | 91.0 | 85.7 | 94.5 | 314 | 84.6 | 70.6 | 92.7 | 92 | 90.4 | 61.1 | 49.6 | 71.4 | 157 | 39.2 | 30.1 | 49.1 | 286 | 22.7 | 12.7 | 37.1 | 96 | 41.5 |
| Guatemala | 1995 | 90.1 | 84.5 | 93.8 | 292 | 76.6 | 69.0 | 82.8 | 337 | 46.8 | 32.6 | 61.6 | 53 | 77.8 | 68.4 | 59.8 | 75.9 | 211 | 51.2 | 43.7 | 58.7 | 258 | 20.9 | 10.2 | 38.1 | 41 | 54.0 |
| Guatemala | 1998 | 94.1 | 80.5 | 98.4 | 86 | 87.1 | 76.9 | 93.2 | 157 | 52.8 | 26.9 | 77.3 | 30 | 84.5 | 66.4 | 48.6 | 80.5 | 102 | 50.2 | 37.7 | 62.6 | 126 | 38.6 | 15.3 | 68.6 | 31 | 52.8 |
| Guatemala | 2014 | 94.0 | 86.8 | 97.4 | 131 | 90.5 | 87.4 | 93.0 | 462 | 70.2 | 62.2 | 77.0 | 232 | 85.3 | 70.7 | 59.9 | 79.6 | 123 | 61.4 | 55.5 | 67.0 | 378 | 41.2 | 33.7 | 49.1 | 214 | 56.8 |
| Guinea | 1999 | 96.0 | 93.3 | 97.6 | 362 | 97.4 | 83.3 | 99.7 | 39 | 100.0 | - | - | 15 | 96.2 | 80.3 | 71.7 | 86.7 | 124 | 54.5 | 25.3 | 80.9 | 11 | 53.7 | 32.0 | 74.1 | 21 | 75.0 |
| Guinea | 2005 | 96.9 | 93.6 | 98.6 | 424 | 97.1 | 89.1 | 99.3 | 43 | 80.4 | 47.9 | 94.8 | 16 | 96.4 | 72.3 | 65.3 | 78.3 | 257 | 65.8 | 37.9 | 85.8 | 20 | 46.9 | 25.5 | 69.4 | 15 | 70.6 |
| Guinea | 2012 | 94.6 | 91.1 | 96.8 | 404 | 88.4 | 75.1 | 95.0 | 72 | 84.2 | 71.7 | 91.8 | 56 | 92.7 | 71.4 | 63.5 | 78.1 | 229 | 47.1 | 28.2 | 66.9 | 31 | 46.2 | 24.2 | 69.9 | 31 | 66.0 |
| Guinea | 2016 | 95.0 | 92.1 | 96.8 | 380 | 93.3 | 84.9 | 97.2 | 71 | 85.1 | 67.7 | 94.0 | 63 | 93.5 | 72.1 | 65.8 | 77.7 | 248 | 38.9 | 26.8 | 52.6 | 66 | 41.3 | 28.8 | 55.0 | 77 | 59.8 |
| Guinea | 2018 | 86.9 | 83.5 | 89.7 | 524 | 91.6 | 82.3 | 96.2 | 78 | 77.6 | 65.9 | 86.1 | 85 | 86.3 | 72.3 | 64.7 | 78.8 | 191 | 52.3 | 34.4 | 69.6 | 33 | 23.6 | 13.0 | 38.9 | 47 | 60.3 |
| Guinea Bissau | 2006 | 93.5 | 90.1 | 95.8 | 392 | 91.9 | 84.8 | 95.8 | 123 | 90.4 | 73.8 | 96.9 | 31 | 92.8 | 70.3 | 62.3 | 77.2 | 178 | 54.4 | 42.7 | 65.7 | 70 | 21.4 | 9.9 | 40.3 | 29 | 61.4 |
| Guinea Bissau | 2014 | 96.3 | 92.0 | 98.3 | 332 | 95.3 | 84.3 | 98.7 | 165 | 87.7 | 76.9 | 93.8 | 76 | 94.6 | 59.6 | 52.6 | 66.3 | 276 | 52.7 | 43.7 | 61.4 | 153 | 23.3 | 14.0 | 36.2 | 60 | 50.9 |
| Guyana | 2006 | 100.0 | - | - | 1 | 57.1 | 36.0 | 76.0 | 42 | 68.7 | 54.8 | 79.9 | 94 | 65.3 | . | . | . | . | 33.8 | 17.1 | 55.8 | 35 | 51.3 | 38.8 | 63.6 | 103 | 48.0 |
| Guyana | 2009 | 92.8 | 49.4 | 99.4 | 4 | 68.6 | 42.9 | 86.4 | 34 | 57.6 | 44.2 | 69.9 | 97 | 61.6 | 66.9 | 13.7 | 96.3 | 3 | 57.0 | 34.9 | 76.6 | 26 | 45.8 | 33.9 | 58.3 | 80 | 49.3 |
| Guyana | 2014 | 73.8 | 35.0 | 93.6 | 10 | 59.7 | 40.0 | 76.7 | 32 | 53.9 | 43.0 | 64.4 | 162 | 55.6 | 47.5 | 13.3 | 84.3 | 7 | 36.4 | 19.9 | 56.9 | 31 | 41.5 | 32.3 | 51.3 | 161 | 40.9 |
| Haiti | 1994 | 92.7 | 83.9 | 96.9 | 96 | 95.2 | 87.3 | 98.3 | 82 | 30.3 | 15.9 | 50.0 | 32 | 84.6 | 37.1 | 26.4 | 49.3 | 81 | 21.8 | 14.5 | 31.5 | 78 | 24.7 | 10.6 | 47.6 | 20 | 29.2 |
| Haiti | 2000 | 84.4 | 69.8 | 92.6 | 150 | 85.5 | 75.8 | 91.8 | 205 | 62.8 | 47.9 | 75.6 | 47 | 82.7 | 46.2 | 36.1 | 56.7 | 152 | 32.7 | 20.5 | 47.9 | 138 | 5.5 | 1.7 | 16.4 | 53 | 31.8 |
| Haiti | 2005 | 90.0 | 82.4 | 94.5 | 135 | 86.9 | 77.2 | 92.8 | 174 | 69.5 | 56.6 | 79.9 | 111 | 82.9 | 41.6 | 32.1 | 51.9 | 126 | 36.8 | 27.4 | 47.3 | 120 | 20.2 | 11.5 | 33.1 | 60 | 34.9 |
| Haiti | 2012 | 88.0 | 79.4 | 93.3 | 96 | 81.3 | 72.7 | 87.7 | 191 | 78.8 | 68.9 | 86.2 | 160 | 81.8 | 43.4 | 30.5 | 57.3 | 82 | 30.6 | 22.5 | 40.1 | 156 | 22.1 | 14.4 | 32.4 | 103 | 30.8 |
| Haiti | 2016 | 84.2 | 69.9 | 92.4 | 73 | 73.0 | 63.6 | 80.7 | 173 | 77.8 | 69.5 | 84.3 | 179 | 76.9 | 31.0 | 21.1 | 42.9 | 65 | 33.0 | 25.4 | 41.7 | 127 | 13.2 | 7.3 | 22.6 | 93 | 24.9 |
| Honduras | 2005 | 87.5 | 77.8 | 93.4 | 73 | 79.8 | 75.5 | 83.4 | 537 | 45.5 | 35.9 | 55.5 | 131 | 72.4 | 64.7 | 51.6 | 75.9 | 72 | 49.3 | 43.9 | 54.7 | 397 | 33.0 | 23.2 | 44.5 | 96 | 47.5 |
| Honduras | 2011 | 80.2 | 62.2 | 90.8 | 45 | 75.5 | 69.4 | 80.7 | 458 | 60.0 | 53.0 | 66.7 | 266 | 69.6 | 57.8 | 41.0 | 72.9 | 44 | 52.0 | 46.2 | 57.9 | 381 | 29.2 | 22.8 | 36.5 | 224 | 43.3 |
| India | 2005 | 92.2 | 90.1 | 93.9 | 1236 | 90.2 | 85.8 | 93.4 | 405 | 85.2 | 82.5 | 87.5 | 1491 | 89.2 | 79.2 | 75.9 | 82.2 | 966 | 77.6 | 71.7 | 82.5 | 374 | 63.1 | 59.3 | 66.8 | 1287 | 72.7 |
| India | 2015 | 88.5 | 87.2 | 89.8 | 4730 | 89.5 | 87.5 | 91.1 | 2178 | 84.4 | 83.2 | 85.4 | 9333 | 86.2 | 79.0 | 77.4 | 80.6 | 4028 | 75.6 | 72.9 | 78.1 | 2056 | 67.3 | 65.7 | 68.8 | 8195 | 71.6 |
| Indonesia | 2002 | 93.6 | 77.9 | 98.4 | 42 | 89.9 | 86.0 | 92.9 | 508 | 78.5 | 73.0 | 83.1 | 544 | 84.6 | 74.1 | 50.9 | 88.8 | 31 | 71.3 | 63.1 | 78.3 | 347 | 47.8 | 40.7 | 55.0 | 442 | 58.7 |
| Indonesia | 2007 | 90.9 | 77.8 | 96.6 | 45 | 86.1 | 80.8 | 90.1 | 460 | 75.5 | 70.7 | 79.7 | 720 | 79.9 | 52.0 | 26.6 | 76.4 | 22 | 61.0 | 53.2 | 68.4 | 414 | 41.6 | 35.4 | 48.2 | 574 | 50.3 |
| Indonesia | 2012 | 90.8 | 75.5 | 96.9 | 30 | 85.1 | 79.6 | 89.3 | 361 | 73.4 | 68.7 | 77.6 | 783 | 77.2 | 47.3 | 24.7 | 71.0 | 21 | 64.6 | 56.2 | 72.1 | 306 | 51.6 | 46.3 | 56.9 | 754 | 55.3 |
| Indonesia | 2017 | 72.4 | 40.9 | 90.9 | 15 | 85.7 | 80.1 | 90.0 | 282 | 73.5 | 69.9 | 76.8 | 905 | 76.5 | 53.7 | 23.1 | 81.8 | 8 | 64.6 | 56.4 | 72.1 | 232 | 51.7 | 47.4 | 55.9 | 826 | 54.6 |
| Iraq | 2006 | 65.1 | 58.3 | 71.3 | 281 | 69.1 | 64.4 | 73.4 | 644 | 66.5 | 60.3 | 72.1 | 384 | 67.6 | 43.6 | 36.4 | 51.1 | 236 | 32.8 | 28.0 | 38.0 | 558 | 35.7 | 29.0 | 43.0 | 334 | 35.7 |
| Iraq | 2011 | 62.8 | 57.6 | 67.7 | 714 | 51.3 | 47.1 | 55.5 | 1339 | 45.4 | 40.5 | 50.5 | 712 | 51.5 | 27.1 | 22.0 | 32.8 | 556 | 23.1 | 19.4 | 27.3 | 1141 | 19.9 | 15.3 | 25.5 | 590 | 22.7 |
| Iraq | 2018 | 46.7 | 36.3 | 57.3 | 211 | 51.0 | 41.8 | 60.1 | 494 | 35.1 | 29.5 | 41.3 | 401 | 44.8 | 31.8 | 23.8 | 41.1 | 232 | 28.1 | 22.4 | 34.5 | 410 | 22.8 | 16.1 | 31.3 | 348 | 26.7 |
| Jamaica | 2005 | . | . | . | . | 100.0 | - | - | 4 | 47.1 | 35.3 | 59.2 | 89 | 49.1 | . | . | . | . | 30.5 | 7.3 | 71.0 | 7 | 24.2 | 15.7 | 35.2 | 88 | 24.0 |
| Jamaica | 2011 | . | . | . | . | 24.0 | 2.6 | 79.1 | 3 | 44.6 | 34.2 | 55.5 | 114 | 44.4 | . | . | . | . | 0.0 | - | - | 3 | 32.1 | 22.6 | 43.5 | 86 | 31.2 |
| Jordan | 1997 | 66.3 | 44.5 | 82.8 | 27 | 48.0 | 35.6 | 60.7 | 47 | 41.8 | 36.9 | 46.9 | 325 | 43.9 | 49.5 | 30.7 | 68.5 | 28 | 23.5 | 12.2 | 40.3 | 37 | 12.7 | 9.1 | 17.5 | 265 | 16.4 |
| Jordan | 2002 | 52.4 | 32.0 | 72.0 | 26 | 68.2 | 49.1 | 82.6 | 37 | 49.7 | 43.3 | 56.2 | 322 | 51.1 | 18.0 | 5.9 | 43.6 | 13 | 17.4 | 7.5 | 35.3 | 37 | 11.8 | 8.2 | 16.6 | 259 | 12.4 |
| Jordan | 2007 | 60.1 | 36.2 | 80.0 | 27 | 58.9 | 38.4 | 76.7 | 44 | 45.0 | 38.5 | 51.6 | 555 | 46.0 | 29.6 | 11.8 | 57.0 | 32 | 24.9 | 7.0 | 59.4 | 34 | 9.4 | 6.2 | 14.0 | 447 | 10.9 |
| Jordan | 2012 | 75.0 | 54.1 | 88.5 | 19 | 44.8 | 20.7 | 71.7 | 39 | 42.9 | 36.4 | 49.7 | 578 | 43.5 | 16.2 | 6.5 | 35.0 | 26 | 26.3 | 10.3 | 52.5 | 48 | 11.7 | 7.9 | 17.1 | 505 | 12.9 |
| Jordan | 2017 | 48.7 | 20.6 | 77.6 | 12 | 54.2 | 31.8 | 75.1 | 40 | 34.7 | 28.9 | 40.9 | 529 | 36.2 | 10.0 | 1.9 | 39.3 | 13 | 25.3 | 10.4 | 49.6 | 40 | 14.3 | 10.1 | 19.7 | 483 | 14.9 |
| Kazakhstan | 1995 | . | . | . | . | . | . | . | . | 52.3 | 39.9 | 64.4 | 108 | 52.3 | . | . | . | . | . | . | . | . | 23.9 | 14.7 | 36.4 | 85 | 23.9 |
| Kazakhstan | 1999 | . | . | . | . | . | . | . | . | 61.7 | 47.9 | 73.9 | 88 | 61.7 | . | . | . | . | . | . | . | . | 19.9 | 11.3 | 32.6 | 67 | 19.9 |
| Kazakhstan | 2006 | 100.0 | - | - | 1 | 0.0 | - | - | 1 | 57.7 | 50.8 | 64.4 | 314 | 57.1 | . | . | . | . | . | . | . | . | 16.3 | 12.4 | 21.1 | 299 | 16.2 |
| Kazakhstan | 2015 | . | . | . | . | . | . | . | . | 61.6 | 55.2 | 67.5 | 372 | 59.8 | . | . | . | . | . | . | . | . | 21.8 | 17.4 | 26.9 | 355 | 21.1 |
| Kenya | 1993 | 89.7 | 78.0 | 95.5 | 67 | 92.8 | 87.4 | 95.9 | 228 | 91.5 | 82.9 | 96.0 | 85 | 91.9 | 72.2 | 57.0 | 83.7 | 48 | 56.9 | 48.4 | 65.1 | 189 | 54.7 | 43.0 | 65.9 | 81 | 58.6 |
| Kenya | 1998 | 82.1 | 58.3 | 93.8 | 44 | 89.6 | 84.2 | 93.3 | 239 | 94.7 | 86.7 | 98.0 | 84 | 89.9 | 67.6 | 50.1 | 81.3 | 46 | 64.2 | 57.1 | 70.7 | 214 | 46.0 | 33.3 | 59.3 | 69 | 60.6 |
| Kenya | 2003 | 93.9 | 87.7 | 97.0 | 73 | 94.8 | 89.8 | 97.4 | 238 | 80.2 | 66.9 | 89.1 | 72 | 92.1 | 85.3 | 71.8 | 93.0 | 60 | 54.1 | 46.5 | 61.6 | 174 | 42.8 | 32.4 | 53.9 | 64 | 57.3 |
| Kenya | 2008 | 81.1 | 47.7 | 95.3 | 73 | 88.5 | 81.9 | 92.9 | 201 | 81.0 | 68.0 | 89.6 | 72 | 86.0 | 63.0 | 48.8 | 75.2 | 49 | 56.6 | 47.8 | 65.1 | 197 | 43.9 | 27.6 | 61.7 | 69 | 53.6 |
| Kenya | 2014 | 87.4 | 80.4 | 92.2 | 161 | 92.8 | 88.8 | 95.4 | 339 | 87.4 | 78.3 | 93.0 | 166 | 90.4 | 61.2 | 48.4 | 72.7 | 89 | 52.1 | 44.7 | 59.4 | 289 | 52.7 | 41.6 | 63.5 | 145 | 53.1 |
| Kyrgyzstan | 1997 | . | . | . | . | . | . | . | . | 79.3 | 71.4 | 85.5 | 132 | 79.3 | . | . | . | . | . | . | . | . | 23.1 | 13.5 | 36.6 | 115 | 23.1 |
| Kyrgyzstan | 2005 | . | . | . | . | . | . | . | . | 68.2 | 56.9 | 77.7 | 250 | 68.2 | . | . | . | . | . | . | . | . | 27.7 | 17.4 | 41.0 | 152 | 27.7 |
| Kyrgyzstan | 2012 | 100.0 | - | - | 1 | 39.5 | 3.9 | 91.4 | 2 | 68.6 | 63.0 | 73.7 | 315 | 68.3 | . | . | . | . | . | . | . | . | 37.0 | 29.7 | 44.8 | 221 | 37.0 |
| Kyrgyzstan | 2014 | 0.0 | - | - | 2 | . | . | . | . | 62.4 | 55.7 | 68.6 | 281 | 60.7 | 0.0 | - | - | 2 | . | . | . | . | 24.7 | 18.5 | 32.1 | 279 | 22.5 |
| Kyrgyzstan | 2018 | . | . | . | . | 100.0 | - | - | 2 | 77.0 | 69.7 | 83.0 | 219 | 77.4 | . | . | . | . | . | . | . | . | 22.4 | 16.2 | 30.0 | 217 | 22.4 |
| Lao | 2006 | 84.2 | 76.0 | 89.9 | 131 | 87.6 | 78.7 | 93.1 | 139 | 60.3 | 45.8 | 73.2 | 50 | 81.7 | 65.4 | 54.5 | 74.9 | 98 | 43.2 | 33.4 | 53.5 | 127 | 18.7 | 8.4 | 36.5 | 36 | 48.4 |
| Lao | 2011 | 85.9 | 79.7 | 90.4 | 256 | 72.2 | 66.3 | 77.3 | 339 | 60.6 | 52.8 | 67.9 | 207 | 73.0 | 58.6 | 50.0 | 66.7 | 192 | 43.2 | 36.2 | 50.4 | 263 | 17.3 | 12.1 | 24.1 | 190 | 40.0 |
| Lao | 2017 | 75.2 | 67.0 | 81.9 | 177 | 67.1 | 60.4 | 73.2 | 279 | 56.6 | 50.1 | 63.0 | 299 | 64.9 | 51.0 | 40.4 | 61.6 | 133 | 31.4 | 25.6 | 37.9 | 289 | 15.3 | 10.9 | 20.9 | 347 | 27.2 |
| Lesotho | 2004 | 68.9 | 17.2 | 95.9 | 4 | 92.9 | 85.9 | 96.6 | 153 | 87.1 | 76.9 | 93.3 | 100 | 90.2 | 81.5 | 28.8 | 98.0 | 5 | 65.5 | 55.3 | 74.5 | 118 | 43.1 | 27.8 | 59.9 | 50 | 59.5 |
| Lesotho | 2009 | 87.8 | 48.3 | 98.2 | 5 | 82.1 | 75.0 | 87.5 | 176 | 69.7 | 59.3 | 78.3 | 116 | 76.5 | 65.9 | 10.6 | 96.9 | 2 | 44.7 | 35.0 | 54.9 | 122 | 25.1 | 17.2 | 35.3 | 94 | 35.1 |
| Lesotho | 2014 | 100.0 | - | - | 1 | 73.2 | 62.5 | 81.8 | 110 | 69.8 | 60.5 | 77.6 | 149 | 71.2 | 100.0 | - | - | 2 | 39.5 | 27.9 | 52.4 | 85 | 18.6 | 12.2 | 27.2 | 83 | 29.5 |
| Lesotho | 2018 | 88.9 | 32.8 | 99.2 | 2 | 60.4 | 47.8 | 71.7 | 90 | 53.8 | 42.0 | 65.2 | 125 | 56.8 | 0.0 | - | - | 4 | 14.2 | 8.2 | 23.5 | 88 | 15.0 | 9.2 | 23.4 | 146 | 14.5 |
| Liberia | 2007 | 86.1 | 76.5 | 92.1 | 132 | 91.1 | 80.9 | 96.1 | 118 | 81.2 | 67.8 | 89.8 | 59 | 86.7 | 61.7 | 47.9 | 73.9 | 128 | 41.2 | 29.4 | 54.1 | 111 | 25.9 | 12.6 | 45.8 | 55 | 47.5 |
| Liberia | 2013 | 93.4 | 86.7 | 96.8 | 189 | 91.2 | 83.1 | 95.6 | 187 | 76.1 | 64.2 | 84.9 | 96 | 87.5 | 51.2 | 42.6 | 59.8 | 199 | 35.2 | 25.5 | 46.1 | 124 | 43.1 | 28.9 | 58.6 | 95 | 44.2 |
| Madagascar | 1997 | 93.3 | 82.0 | 97.7 | 88 | 91.4 | 86.9 | 94.4 | 203 | 90.1 | 80.7 | 95.2 | 96 | 91.5 | 63.8 | 49.5 | 76.1 | 54 | 60.4 | 51.3 | 68.9 | 144 | 35.0 | 24.4 | 47.3 | 64 | 55.5 |
| Madagascar | 2003 | 93.2 | 83.3 | 97.4 | 84 | 92.2 | 84.9 | 96.1 | 168 | 84.6 | 69.4 | 93.0 | 104 | 90.9 | 80.1 | 61.2 | 91.1 | 52 | 57.7 | 45.6 | 68.9 | 124 | 63.3 | 50.4 | 74.5 | 93 | 64.1 |
| Madagascar | 2008 | 86.8 | 80.5 | 91.3 | 228 | 93.9 | 89.7 | 96.4 | 376 | 93.6 | 87.7 | 96.8 | 150 | 92.1 | 58.1 | 48.5 | 67.2 | 172 | 63.0 | 56.2 | 69.4 | 323 | 56.6 | 45.7 | 67.0 | 148 | 60.5 |
| Madagascar | 2018 | 87.8 | 82.3 | 91.8 | 250 | 90.8 | 87.2 | 93.5 | 450 | 94.4 | 90.8 | 96.7 | 234 | 91.2 | 52.4 | 43.9 | 60.9 | 187 | 63.7 | 57.6 | 69.4 | 402 | 54.9 | 46.2 | 63.3 | 175 | 58.9 |
| Malawi | 2000 | 98.7 | 96.2 | 99.6 | 211 | 97.8 | 95.4 | 98.9 | 491 | 100.0 | - | - | 75 | 98.2 | 81.6 | 74.3 | 87.2 | 173 | 76.2 | 71.1 | 80.6 | 432 | 58.0 | 42.1 | 72.4 | 50 | 76.5 |
| Malawi | 2004 | 97.9 | 94.9 | 99.1 | 217 | 97.8 | 96.2 | 98.8 | 512 | 95.6 | 86.8 | 98.6 | 68 | 97.7 | 85.8 | 77.2 | 91.6 | 158 | 79.3 | 74.4 | 83.4 | 371 | 73.5 | 60.8 | 83.3 | 82 | 80.3 |
| Malawi | 2006 | 95.2 | 92.1 | 97.1 | 387 | 98.4 | 97.3 | 99.1 | 1211 | 95.8 | 90.4 | 98.2 | 202 | 97.4 | 72.0 | 62.6 | 79.8 | 328 | 72.5 | 68.7 | 75.9 | 1095 | 71.3 | 61.0 | 79.8 | 183 | 72.2 |
| Malawi | 2010 | 97.3 | 92.6 | 99.0 | 192 | 96.4 | 94.2 | 97.8 | 789 | 94.2 | 86.9 | 97.5 | 193 | 96.1 | 75.9 | 66.9 | 83.0 | 200 | 79.5 | 75.9 | 82.7 | 894 | 64.4 | 54.6 | 73.2 | 172 | 76.8 |
| Malawi | 2013 | 97.1 | 92.5 | 98.9 | 155 | 97.9 | 96.3 | 98.8 | 991 | 94.2 | 88.2 | 97.3 | 247 | 97.2 | 79.3 | 69.7 | 86.4 | 122 | 79.5 | 75.6 | 82.9 | 821 | 58.9 | 49.7 | 67.5 | 235 | 75.4 |
| Malawi | 2015 | 92.2 | 85.2 | 96.1 | 127 | 90.3 | 87.6 | 92.4 | 765 | 95.5 | 90.3 | 98.0 | 226 | 91.6 | 86.8 | 78.9 | 92.1 | 104 | 71.9 | 67.6 | 75.8 | 702 | 61.5 | 50.9 | 71.1 | 217 | 71.5 |
| Maldives | 2009 | 71.8 | 49.9 | 86.7 | 30 | 85.0 | 74.8 | 91.5 | 101 | 73.4 | 62.1 | 82.2 | 132 | 77.3 | 65.2 | 46.1 | 80.5 | 38 | 78.0 | 66.8 | 86.3 | 92 | 63.4 | 53.0 | 72.6 | 144 | 68.4 |
| Maldives | 2016 | 100.0 | - | - | 4 | 82.9 | 48.5 | 96.2 | 26 | 77.0 | 66.4 | 85.1 | 169 | 78.2 | 100.0 | - | - | 4 | 67.1 | 41.7 | 85.3 | 35 | 61.1 | 49.5 | 71.5 | 163 | 62.7 |
| Mali | 1995 | 94.3 | 92.0 | 96.0 | 524 | 100.0 | - | - | 58 | 92.7 | 76.8 | 98.0 | 30 | 94.8 | 64.2 | 58.9 | 69.2 | 359 | 65.5 | 48.6 | 79.2 | 51 | 31.6 | 12.3 | 60.5 | 16 | 63.1 |
| Mali | 2001 | 96.5 | 94.7 | 97.7 | 828 | 89.7 | 76.6 | 95.8 | 107 | 90.1 | 78.2 | 95.9 | 49 | 95.4 | 71.1 | 63.8 | 77.4 | 389 | 65.6 | 52.1 | 76.9 | 64 | 45.9 | 28.1 | 64.9 | 22 | 69.3 |
| Mali | 2006 | 94.2 | 91.6 | 96.1 | 854 | 96.2 | 88.5 | 98.8 | 88 | 88.2 | 76.4 | 94.6 | 59 | 94.1 | 58.3 | 52.6 | 63.9 | 509 | 47.8 | 31.6 | 64.5 | 57 | 37.7 | 22.0 | 56.4 | 42 | 56.1 |
| Mali | 2009 | 90.1 | 88.1 | 91.8 | 1483 | 92.0 | 87.1 | 95.2 | 243 | 89.2 | 83.2 | 93.3 | 151 | 90.2 | 47.5 | 43.9 | 51.2 | 1270 | 43.1 | 34.4 | 52.2 | 198 | 30.4 | 21.8 | 40.7 | 114 | 45.7 |
| Mali | 2012 | 89.7 | 86.1 | 92.4 | 462 | 88.2 | 75.2 | 94.9 | 54 | 85.6 | 73.3 | 92.8 | 68 | 89.1 | 76.8 | 71.5 | 81.4 | 441 | 52.0 | 37.3 | 66.4 | 53 | 55.6 | 39.7 | 70.4 | 49 | 73.0 |
| Mali | 2015 | 92.3 | 90.0 | 94.1 | 983 | 96.7 | 92.8 | 98.5 | 149 | 91.4 | 78.4 | 96.9 | 90 | 92.3 | 55.6 | 51.3 | 59.9 | 565 | 55.9 | 44.4 | 66.8 | 101 | 41.3 | 29.3 | 54.4 | 68 | 53.4 |
| Mali | 2018 | 91.5 | 88.2 | 94.0 | 447 | 89.9 | 79.2 | 95.4 | 82 | 88.4 | 80.4 | 93.4 | 115 | 90.8 | 58.7 | 52.7 | 64.5 | 335 | 50.3 | 35.2 | 65.3 | 54 | 43.4 | 30.9 | 56.8 | 79 | 55.2 |
| Mauritania | 2007 | 85.7 | 80.7 | 89.6 | 251 | 86.0 | 79.4 | 90.7 | 211 | 80.4 | 68.8 | 88.4 | 71 | 85.1 | 37.3 | 28.1 | 47.5 | 137 | 25.7 | 18.8 | 34.0 | 136 | 23.8 | 13.6 | 38.3 | 66 | 33.1 |
| Mauritania | 2011 | 84.0 | 76.4 | 89.5 | 161 | 88.0 | 82.6 | 91.9 | 220 | 82.4 | 73.1 | 88.9 | 105 | 85.0 | 46.4 | 37.0 | 56.0 | 116 | 30.9 | 23.8 | 38.9 | 171 | 29.7 | 20.8 | 40.5 | 103 | 35.5 |
| Mauritania | 2015 | 88.1 | 82.6 | 92.0 | 238 | 83.8 | 79.0 | 87.7 | 510 | 87.0 | 78.8 | 92.3 | 134 | 85.6 | 39.1 | 28.9 | 50.4 | 134 | 44.5 | 37.8 | 51.3 | 317 | 25.3 | 16.9 | 36.1 | 109 | 39.6 |
| Moldova | 2005 | 0.0 | - | - | 1 | 35.8 | 2.5 | 92.4 | 2 | 41.1 | 33.2 | 49.4 | 130 | 40.8 | . | . | . | . | 0.0 | - | - | 1 | 2.5 | 0.5 | 10.8 | 93 | 2.4 |
| Moldova | 2012 | . | . | . | . | 100.0 | - | - | 1 | 47.7 | 37.7 | 57.9 | 111 | 48.4 | . | . | . | . | . | . | . | . | 12.3 | 6.6 | 21.6 | 130 | 12.2 |
| Mongolia | 2005 | 100.0 | - | - | 9 | 87.1 | 66.2 | 95.9 | 23 | 80.8 | 74.2 | 86.0 | 178 | 82.3 | 62.4 | 26.1 | 88.7 | 8 | 41.5 | 19.7 | 67.2 | 12 | 66.5 | 59.5 | 72.8 | 188 | 64.9 |
| Mongolia | 2010 | 97.0 | 80.5 | 99.6 | 18 | 89.3 | 71.3 | 96.5 | 28 | 81.2 | 75.6 | 85.8 | 268 | 82.4 | 61.4 | 29.5 | 85.8 | 11 | 66.1 | 40.4 | 84.9 | 20 | 64.8 | 57.1 | 71.7 | 214 | 64.7 |
| Mongolia | 2013 | 89.6 | 70.0 | 97.0 | 22 | 92.7 | 74.2 | 98.3 | 26 | 81.4 | 76.5 | 85.5 | 334 | 82.5 | 66.7 | 42.3 | 84.6 | 18 | 54.3 | 37.8 | 69.9 | 35 | 52.0 | 46.1 | 58.0 | 324 | 52.9 |
| Mongolia | 2018 | 84.9 | 58.2 | 95.8 | 15 | 95.6 | 80.7 | 99.1 | 15 | 80.0 | 74.1 | 84.9 | 349 | 80.7 | 69.1 | 34.8 | 90.3 | 20 | 64.6 | 38.1 | 84.4 | 23 | 44.3 | 36.3 | 52.6 | 281 | 46.3 |
| Montenegro | 2005 | . | . | . | . | 30.3 | 12.2 | 57.5 | 14 | 22.2 | 12.5 | 36.2 | 48 | 23.9 | . | . | . | . | 22.4 | 5.1 | 60.7 | 14 | 10.3 | 4.3 | 22.5 | 47 | 13.1 |
| Montenegro | 2013 | 100.0 | - | - | 1 | 38.8 | 16.8 | 66.6 | 16 | 17.2 | 9.0 | 30.4 | 65 | 23.9 | 100.0 | - | - | 1 | 23.2 | 9.5 | 46.6 | 15 | 4.7 | 1.7 | 12.6 | 69 | 9.0 |
| Montenegro | 2018 | 74.4 | 34.6 | 94.1 | 4 | 26.0 | 5.6 | 67.6 | 6 | 32.8 | 19.8 | 49.2 | 55 | 34.5 | 33.3 | . | . | 3 | 38.8 | 10.5 | 77.5 | 8 | 5.1 | 1.8 | 13.7 | 62 | 13.8 |
| Mozambique | 2003 | 96.6 | 93.0 | 98.4 | 278 | 94.9 | 90.6 | 97.2 | 343 | 67.4 | 48.0 | 82.3 | 42 | 94.4 | 66.6 | 58.0 | 74.2 | 207 | 65.4 | 58.2 | 71.9 | 305 | 27.3 | 10.6 | 54.3 | 26 | 64.7 |
| Mozambique | 2008 | 92.2 | 87.5 | 95.2 | 233 | 92.8 | 88.8 | 95.4 | 552 | 74.5 | 59.3 | 85.4 | 94 | 91.2 | 70.0 | 61.3 | 77.4 | 186 | 49.5 | 42.4 | 56.5 | 418 | 22.9 | 12.7 | 37.8 | 66 | 54.0 |
| Mozambique | 2011 | 92.5 | 86.7 | 95.9 | 251 | 92.2 | 89.0 | 94.6 | 381 | 80.2 | 71.6 | 86.7 | 138 | 90.6 | 60.2 | 51.1 | 68.7 | 193 | 51.4 | 44.0 | 58.7 | 324 | 27.5 | 16.7 | 41.8 | 88 | 51.5 |
| Namibia | 2006 | 76.1 | 62.4 | 85.9 | 45 | 76.8 | 62.3 | 86.9 | 94 | 63.4 | 53.1 | 72.6 | 206 | 68.5 | 40.6 | 25.2 | 58.2 | 39 | 42.9 | 32.1 | 54.4 | 81 | 18.6 | 12.1 | 27.7 | 131 | 28.4 |
| Namibia | 2013 | 80.1 | 55.6 | 92.8 | 21 | 83.9 | 74.7 | 90.2 | 82 | 55.6 | 48.1 | 62.8 | 208 | 64.4 | 34.9 | 16.8 | 58.8 | 21 | 25.3 | 15.8 | 37.9 | 63 | 18.0 | 12.0 | 26.1 | 155 | 21.0 |
| Nepal | 1996 | 97.0 | 94.8 | 98.3 | 408 | 97.0 | 87.1 | 99.4 | 52 | 95.3 | 82.6 | 98.9 | 45 | 96.9 | 93.6 | 90.2 | 95.9 | 337 | 94.0 | 80.0 | 98.4 | 41 | 81.7 | 61.6 | 92.5 | 30 | 92.8 |
| Nepal | 2006 | 98.2 | 92.9 | 99.6 | 181 | 97.6 | 88.0 | 99.5 | 67 | 95.8 | 87.0 | 98.7 | 83 | 97.5 | 95.5 | 89.3 | 98.2 | 200 | 93.3 | 82.5 | 97.6 | 59 | 94.8 | 86.0 | 98.1 | 80 | 95.0 |
| Nepal | 2011 | 88.0 | 77.0 | 94.1 | 130 | 94.2 | 82.4 | 98.2 | 60 | 96.7 | 91.2 | 98.8 | 126 | 92.5 | 94.2 | 85.8 | 97.8 | 125 | 89.7 | 73.7 | 96.4 | 53 | 92.0 | 80.6 | 97.0 | 95 | 92.6 |
| Nepal | 2014 | 96.3 | 89.2 | 98.8 | 135 | 92.5 | 62.4 | 98.9 | 54 | 91.8 | 81.2 | 96.7 | 145 | 93.6 | 82.0 | 72.0 | 89.0 | 143 | 92.5 | 75.6 | 98.0 | 59 | 89.5 | 78.8 | 95.2 | 119 | 86.7 |
| Nepal | 2016 | 98.6 | 95.2 | 99.6 | 95 | 97.5 | 89.6 | 99.4 | 74 | 98.1 | 92.5 | 99.5 | 171 | 98.1 | 86.0 | 78.1 | 91.4 | 100 | 83.5 | 69.5 | 91.9 | 68 | 91.9 | 83.6 | 96.2 | 179 | 88.5 |
| Niger | 1998 | 95.9 | 93.2 | 97.5 | 469 | 94.3 | 81.7 | 98.4 | 49 | 96.7 | 78.9 | 99.6 | 22 | 95.8 | 52.9 | 46.7 | 59.0 | 280 | 53.4 | 38.9 | 67.3 | 47 | 35.2 | 16.9 | 59.1 | 24 | 52.1 |
| Niger | 2006 | 95.9 | 93.8 | 97.3 | 566 | 93.9 | 82.5 | 98.1 | 73 | 91.8 | 76.1 | 97.5 | 35 | 95.6 | 66.0 | 60.1 | 71.5 | 325 | 57.0 | 41.1 | 71.6 | 50 | 16.9 | 5.8 | 40.1 | 29 | 62.3 |
| Niger | 2012 | 92.5 | 90.0 | 94.5 | 781 | 92.3 | 83.8 | 96.5 | 87 | 93.2 | 81.3 | 97.7 | 57 | 92.6 | 50.6 | 45.2 | 56.0 | 416 | 53.0 | 39.4 | 66.2 | 71 | 36.7 | 22.2 | 54.2 | 46 | 50.1 |
| Nigeria | 1999 | 90.5 | 86.6 | 93.4 | 354 | 78.6 | 69.5 | 85.5 | 114 | 72.8 | 64.4 | 79.9 | 110 | 84.7 | 57.5 | 47.2 | 67.2 | 89 | 25.9 | 16.6 | 38.1 | 69 | 21.8 | 14.0 | 32.2 | 84 | 35.7 |
| Nigeria | 2003 | 94.9 | 90.8 | 97.2 | 197 | 92.6 | 82.7 | 97.0 | 96 | 75.9 | 63.9 | 84.8 | 98 | 89.9 | 49.2 | 38.0 | 60.5 | 118 | 32.1 | 20.7 | 46.2 | 69 | 12.1 | 6.5 | 21.5 | 80 | 34.1 |
| Nigeria | 2007 | 79.6 | 75.1 | 83.4 | 1074 | 81.3 | 75.1 | 86.2 | 291 | 69.1 | 62.8 | 74.8 | 324 | 77.8 | 49.6 | 41.8 | 57.5 | 242 | 30.9 | 22.4 | 40.9 | 178 | 12.9 | 8.4 | 19.3 | 231 | 30.5 |
| Nigeria | 2008 | 91.6 | 89.7 | 93.2 | 1141 | 88.6 | 85.0 | 91.5 | 432 | 71.8 | 67.2 | 76.0 | 512 | 85.4 | 54.7 | 49.7 | 59.7 | 484 | 24.1 | 19.0 | 30.1 | 258 | 11.0 | 7.8 | 15.3 | 352 | 32.3 |
| Nigeria | 2011 | 88.3 | 85.1 | 91.0 | 928 | 79.6 | 72.7 | 85.0 | 361 | 68.4 | 62.7 | 73.6 | 598 | 79.3 | 58.2 | 51.9 | 64.3 | 670 | 31.6 | 22.7 | 42.0 | 336 | 12.3 | 9.1 | 16.4 | 523 | 34.5 |
| Nigeria | 2013 | 89.9 | 87.6 | 91.7 | 1140 | 86.9 | 83.1 | 90.0 | 437 | 72.2 | 67.8 | 76.1 | 727 | 84.0 | 57.1 | 52.6 | 61.4 | 644 | 23.7 | 17.9 | 30.6 | 298 | 14.1 | 11.0 | 17.9 | 524 | 35.3 |
| Nigeria | 2016 | 89.4 | 85.8 | 92.1 | 530 | 85.1 | 78.5 | 90.0 | 318 | 80.1 | 75.8 | 83.7 | 583 | 85.9 | 54.0 | 48.6 | 59.3 | 388 | 38.6 | 31.5 | 46.2 | 252 | 15.7 | 11.8 | 20.5 | 499 | 37.1 |
| Nigeria | 2018 | 92.3 | 90.2 | 94.0 | 1057 | 84.7 | 80.0 | 88.4 | 339 | 70.9 | 67.1 | 74.4 | 870 | 82.9 | 52.2 | 47.1 | 57.3 | 562 | 24.5 | 18.7 | 31.3 | 226 | 8.6 | 6.3 | 11.5 | 657 | 27.8 |
| North Macedonia | 2005 | 81.0 | 52.9 | 94.2 | 35 | 43.4 | 29.4 | 58.6 | 195 | 29.7 | 19.9 | 41.7 | 87 | 44.8 | 47.9 | 23.1 | 73.7 | 26 | 27.5 | 18.1 | 39.6 | 163 | 8.1 | 3.6 | 17.1 | 82 | 21.9 |
| North Macedonia | 2011 | 100.0 | - | - | 1 | 27.8 | 14.1 | 47.4 | 31 | 36.4 | 24.8 | 49.9 | 55 | 33.8 | 64.0 | 12.9 | 95.5 | 3 | 15.8 | 6.1 | 35.3 | 27 | 8.4 | 3.1 | 20.7 | 58 | 12.8 |
| Pakistan | 2012 | 85.7 | 80.9 | 89.4 | 413 | 78.7 | 68.2 | 86.3 | 123 | 72.8 | 65.6 | 79.1 | 266 | 80.6 | 58.5 | 49.9 | 66.6 | 225 | 68.4 | 50.4 | 82.1 | 66 | 45.7 | 35.1 | 56.8 | 141 | 56.1 |
| Pakistan | 2017 | 80.8 | 74.7 | 85.7 | 350 | 62.4 | 48.7 | 74.5 | 94 | 59.6 | 50.9 | 67.8 | 257 | 69.6 | 58.1 | 50.0 | 65.9 | 232 | 45.3 | 29.5 | 62.1 | 49 | 50.2 | 40.0 | 60.3 | 141 | 53.4 |
| Peru | 1996 | 94.0 | 88.2 | 97.1 | 112 | 87.2 | 83.1 | 90.5 | 498 | 71.4 | 65.7 | 76.4 | 502 | 79.6 | 71.1 | 59.3 | 80.7 | 100 | 46.8 | 40.9 | 52.9 | 434 | 42.8 | 36.6 | 49.2 | 435 | 47.0 |
| Peru | 2000 | 97.5 | 91.9 | 99.3 | 79 | 91.6 | 87.2 | 94.6 | 344 | 77.1 | 71.1 | 82.1 | 421 | 83.9 | 68.6 | 55.5 | 79.4 | 72 | 50.1 | 43.5 | 56.7 | 339 | 46.0 | 39.4 | 52.7 | 367 | 49.3 |
| Peru | 2004 | 100.0 | - | - | 7 | 82.8 | 70.2 | 90.8 | 67 | 63.9 | 47.7 | 77.5 | 99 | 71.6 | 44.0 | 12.7 | 81.0 | 8 | 47.1 | 31.1 | 63.6 | 60 | 37.2 | 26.3 | 49.5 | 91 | 40.6 |
| Peru | 2005 | 100.0 | - | - | 6 | 95.4 | 85.2 | 98.7 | 69 | 72.8 | 59.2 | 83.2 | 109 | 80.8 | 93.8 | 62.4 | 99.3 | 6 | 48.5 | 35.1 | 62.1 | 81 | 43.2 | 29.3 | 58.2 | 99 | 46.9 |
| Peru | 2006 | 89.3 | 48.8 | 98.6 | 10 | 95.7 | 81.1 | 99.1 | 78 | 73.6 | 58.0 | 85.0 | 110 | 82.7 | 85.3 | 57.8 | 96.1 | 13 | 58.8 | 44.5 | 71.8 | 71 | 49.5 | 36.0 | 63.0 | 97 | 54.5 |
| Peru | 2007 | 100.0 | - | - | 4 | 83.0 | 64.5 | 92.9 | 68 | 72.6 | 57.4 | 83.9 | 90 | 77.1 | 100.0 | - | - | 7 | 65.3 | 55.4 | 74.0 | 77 | 44.1 | 30.4 | 58.7 | 94 | 54.0 |
| Peru | 2008 | 95.6 | 73.5 | 99.4 | 14 | 92.0 | 85.4 | 95.8 | 132 | 73.5 | 65.3 | 80.4 | 267 | 79.9 | 70.0 | 45.0 | 87.0 | 18 | 50.9 | 39.6 | 62.2 | 127 | 43.2 | 33.5 | 53.5 | 234 | 46.9 |
| Peru | 2009 | 98.7 | 91.3 | 99.8 | 27 | 88.6 | 81.7 | 93.1 | 224 | 76.3 | 70.2 | 81.5 | 448 | 80.5 | 75.8 | 51.8 | 90.1 | 25 | 58.8 | 49.5 | 67.4 | 191 | 43.0 | 36.8 | 49.3 | 398 | 48.3 |
| Peru | 2010 | 100.0 | - | - | 12 | 88.9 | 80.9 | 93.8 | 223 | 83.2 | 76.6 | 88.2 | 368 | 85.5 | 82.6 | 47.9 | 96.1 | 13 | 60.6 | 52.7 | 68.0 | 194 | 51.4 | 44.1 | 58.5 | 367 | 54.8 |
| Peru | 2011 | 100.0 | - | - | 18 | 92.7 | 88.0 | 95.7 | 208 | 75.8 | 69.1 | 81.4 | 363 | 81.2 | 60.9 | 37.1 | 80.4 | 24 | 54.4 | 45.4 | 63.1 | 197 | 46.3 | 39.1 | 53.5 | 335 | 49.2 |
| Peru | 2012 | 92.0 | 65.5 | 98.6 | 18 | 90.0 | 82.2 | 94.6 | 191 | 78.9 | 72.5 | 84.1 | 391 | 82.3 | 56.5 | 29.0 | 80.5 | 17 | 53.1 | 44.5 | 61.5 | 199 | 45.9 | 38.7 | 53.3 | 403 | 48.1 |
| Peru | 2013 | 94.5 | 69.0 | 99.2 | 18 | 87.5 | 80.3 | 92.4 | 163 | 81.6 | 75.1 | 86.7 | 400 | 83.2 | 68.3 | 39.5 | 87.7 | 17 | 49.5 | 40.5 | 58.6 | 173 | 47.7 | 40.7 | 54.8 | 361 | 48.7 |
| Peru | 2014 | 100.0 | - | - | 3 | 90.6 | 82.1 | 95.3 | 166 | 81.0 | 75.6 | 85.5 | 501 | 82.9 | 86.0 | 60.4 | 96.1 | 17 | 55.7 | 46.1 | 64.8 | 154 | 43.5 | 37.1 | 50.0 | 406 | 47.3 |
| Peru | 2015 | 98.4 | 89.1 | 99.8 | 29 | 90.5 | 85.9 | 93.6 | 365 | 80.4 | 76.9 | 83.4 | 1254 | 82.7 | 56.2 | 30.8 | 78.8 | 19 | 55.5 | 49.0 | 61.8 | 357 | 42.3 | 38.3 | 46.4 | 1173 | 45.3 |
| Peru | 2016 | 92.7 | 62.6 | 99.0 | 20 | 87.7 | 82.3 | 91.5 | 284 | 78.8 | 75.1 | 82.1 | 1101 | 80.7 | 71.2 | 50.7 | 85.6 | 28 | 53.2 | 46.0 | 60.3 | 284 | 49.3 | 45.3 | 53.3 | 1040 | 50.4 |
| Peru | 2017 | 96.0 | 75.9 | 99.5 | 21 | 89.4 | 84.7 | 92.7 | 270 | 80.9 | 77.6 | 83.9 | 1191 | 82.6 | 60.8 | 39.0 | 79.1 | 25 | 50.8 | 43.1 | 58.5 | 254 | 46.5 | 42.7 | 50.5 | 1155 | 47.5 |
| Peru | 2018 | 100.0 | - | - | 24 | 93.5 | 89.5 | 96.1 | 294 | 81.0 | 77.8 | 83.8 | 1163 | 83.6 | 58.4 | 31.2 | 81.3 | 15 | 43.4 | 36.4 | 50.6 | 251 | 51.7 | 47.9 | 55.4 | 1141 | 50.3 |
| Philippines | 1993 | 83.0 | 60.5 | 94.0 | 19 | 71.1 | 64.6 | 76.9 | 240 | 48.1 | 42.1 | 54.3 | 313 | 58.6 | 36.4 | 15.2 | 64.7 | 9 | 33.1 | 26.2 | 40.9 | 172 | 18.3 | 13.9 | 23.7 | 255 | 24.3 |
| Philippines | 1998 | 81.7 | 47.6 | 95.7 | 10 | 75.9 | 67.5 | 82.6 | 185 | 37.1 | 31.2 | 43.4 | 301 | 50.8 | 58.4 | 28.7 | 83.0 | 11 | 45.5 | 35.7 | 55.6 | 136 | 20.6 | 15.8 | 26.5 | 221 | 29.5 |
| Philippines | 2003 | 73.1 | 41.0 | 91.4 | 8 | 69.6 | 60.5 | 77.3 | 144 | 49.2 | 43.2 | 55.2 | 293 | 56.0 | 53.8 | 24.3 | 80.9 | 9 | 42.9 | 33.2 | 53.1 | 101 | 28.4 | 23.1 | 34.4 | 289 | 32.3 |
| Philippines | 2008 | 76.7 | 45.5 | 92.8 | 13 | 78.7 | 68.3 | 86.4 | 96 | 51.2 | 45.4 | 56.9 | 325 | 57.7 | 69.0 | 29.4 | 92.2 | 7 | 43.8 | 32.9 | 55.3 | 100 | 30.3 | 24.6 | 36.6 | 277 | 34.2 |
| Rwanda | 2000 | 97.4 | 93.5 | 99.0 | 147 | 97.6 | 95.1 | 98.9 | 297 | 81.3 | 69.1 | 89.5 | 67 | 95.7 | 79.2 | 68.6 | 86.9 | 77 | 69.0 | 59.4 | 77.2 | 130 | 57.8 | 42.0 | 72.0 | 42 | 70.8 |
| Rwanda | 2005 | 96.1 | 91.4 | 98.3 | 144 | 97.0 | 94.7 | 98.4 | 393 | 91.4 | 80.3 | 96.5 | 53 | 96.4 | 85.5 | 77.9 | 90.8 | 121 | 73.5 | 67.2 | 79.0 | 286 | 76.7 | 59.4 | 88.1 | 35 | 77.1 |
| Rwanda | 2010 | 95.2 | 87.9 | 98.2 | 87 | 95.4 | 92.6 | 97.2 | 377 | 90.1 | 78.3 | 95.9 | 42 | 95.0 | 82.9 | 73.5 | 89.4 | 96 | 84.4 | 80.6 | 87.6 | 375 | 74.3 | 58.1 | 85.8 | 39 | 83.5 |
| Rwanda | 2014 | 97.4 | 83.2 | 99.6 | 72 | 96.0 | 93.3 | 97.6 | 367 | 90.7 | 76.4 | 96.7 | 63 | 95.6 | 92.2 | 82.5 | 96.7 | 68 | 88.3 | 84.0 | 91.6 | 314 | 76.7 | 62.5 | 86.7 | 71 | 87.2 |
| Sao Tome and Principe | 2008 | 100.0 | - | - | 3 | 93.0 | 86.6 | 96.4 | 79 | 89.5 | 70.5 | 96.8 | 27 | 91.9 | 25.3 | 6.6 | 61.8 | 9 | 17.9 | 9.4 | 31.5 | 87 | 27.1 | 11.3 | 52.0 | 22 | 20.0 |
| Sao Tome and Principe | 2014 | 100.0 | - | - | 10 | 85.3 | 74.4 | 92.1 | 86 | 84.8 | 67.7 | 93.7 | 41 | 85.9 | 3.3 | 0.4 | 22.7 | 7 | 26.8 | 16.3 | 40.7 | 80 | 21.7 | 10.5 | 39.7 | 42 | 24.1 |
| Senegal | 2005 | 92.6 | 89.8 | 94.8 | 515 | 96.8 | 92.2 | 98.7 | 143 | 90.0 | 73.2 | 96.8 | 43 | 93.2 | 43.0 | 36.6 | 49.7 | 407 | 36.5 | 25.9 | 48.7 | 93 | 41.0 | 18.8 | 67.5 | 26 | 41.8 |
| Senegal | 2010 | 97.9 | 96.4 | 98.8 | 659 | 94.6 | 87.4 | 97.8 | 184 | 97.4 | 92.2 | 99.2 | 69 | 97.1 | 55.3 | 49.1 | 61.3 | 430 | 43.2 | 30.5 | 56.8 | 123 | 36.3 | 18.8 | 58.3 | 41 | 50.8 |
| Senegal | 2012 | 98.2 | 96.0 | 99.2 | 306 | 98.8 | 91.7 | 99.8 | 102 | 91.1 | 70.8 | 97.7 | 52 | 97.4 | 49.9 | 42.2 | 57.6 | 266 | 39.9 | 25.0 | 57.0 | 67 | 45.4 | 25.6 | 66.7 | 23 | 47.1 |
| Senegal | 2014 | 93.7 | 85.2 | 97.4 | 284 | 90.8 | 72.1 | 97.4 | 85 | 100.0 | - | - | 34 | 93.7 | 45.4 | 37.7 | 53.3 | 286 | 40.2 | 26.4 | 55.8 | 75 | 11.7 | 4.6 | 26.6 | 36 | 41.0 |
| Senegal | 2015 | 98.4 | 96.0 | 99.4 | 298 | 94.2 | 73.3 | 99.0 | 98 | 93.2 | 72.4 | 98.6 | 38 | 96.9 | 49.8 | 41.8 | 57.9 | 267 | 49.5 | 32.5 | 66.6 | 62 | 34.6 | 18.2 | 55.9 | 48 | 47.7 |
| Senegal | 2016 | 96.5 | 92.9 | 98.3 | 322 | 99.0 | 92.9 | 99.9 | 67 | 97.4 | 83.1 | 99.6 | 40 | 97.1 | 54.7 | 46.2 | 63.0 | 262 | 30.6 | 18.4 | 46.3 | 93 | 20.3 | 10.7 | 35.2 | 41 | 45.0 |
| Senegal | 2017 | 94.6 | 91.8 | 96.5 | 467 | 93.7 | 86.4 | 97.2 | 156 | 93.9 | 79.4 | 98.4 | 111 | 94.3 | 43.2 | 37.7 | 48.9 | 479 | 38.3 | 28.3 | 49.3 | 138 | 30.5 | 21.1 | 41.9 | 135 | 40.0 |
| Serbia | 2005 | . | . | . | . | 25.8 | 16.6 | 37.8 | 130 | 21.9 | 15.9 | 29.2 | 147 | 22.8 | . | . | . | . | 14.4 | 7.8 | 25.1 | 129 | 5.9 | 3.0 | 11.4 | 130 | 8.2 |
| Serbia | 2010 | 0.0 | - | - | 1 | 17.8 | 9.3 | 31.3 | 34 | 19.0 | 11.8 | 29.2 | 206 | 18.4 | 0.0 | - | - | 2 | 13.0 | 2.3 | 48.9 | 26 | 15.9 | 7.9 | 29.3 | 195 | 15.3 |
| Serbia | 2014 | 100.0 | - | - | 1 | 30.6 | 11.0 | 61.2 | 17 | 23.2 | 15.1 | 34.0 | 128 | 24.6 | 40.8 | 4.0 | 92.0 | 2 | 11.8 | 4.0 | 29.7 | 16 | 8.2 | 4.6 | 14.2 | 165 | 8.9 |
| Sierra Leone | 2005 | 88.4 | 83.8 | 91.8 | 304 | 82.1 | 67.1 | 91.1 | 40 | 84.8 | 67.8 | 93.7 | 31 | 87.5 | 61.5 | 54.5 | 68.0 | 208 | 42.7 | 25.5 | 61.9 | 26 | 34.4 | 20.8 | 51.3 | 32 | 56.8 |
| Sierra Leone | 2008 | 82.8 | 77.5 | 87.1 | 324 | 81.1 | 69.0 | 89.2 | 67 | 76.2 | 60.4 | 87.1 | 52 | 81.9 | 56.6 | 47.6 | 65.2 | 167 | 42.2 | 21.0 | 66.7 | 26 | 16.8 | 6.3 | 37.4 | 25 | 50.2 |
| Sierra Leone | 2010 | 82.7 | 77.3 | 86.9 | 319 | 87.7 | 75.0 | 94.4 | 66 | 84.6 | 74.5 | 91.2 | 78 | 83.8 | 52.1 | 45.7 | 58.5 | 321 | 52.1 | 38.4 | 65.6 | 59 | 29.9 | 20.4 | 41.5 | 88 | 48.2 |
| Sierra Leone | 2013 | 84.4 | 80.6 | 87.6 | 547 | 88.8 | 79.2 | 94.3 | 118 | 89.9 | 81.7 | 94.7 | 138 | 86.0 | 54.5 | 47.4 | 61.5 | 269 | 50.8 | 35.9 | 65.5 | 57 | 32.0 | 22.2 | 43.8 | 109 | 47.9 |
| Sierra Leone | 2017 | 86.8 | 83.2 | 89.7 | 499 | 85.2 | 73.9 | 92.1 | 115 | 81.1 | 73.9 | 86.6 | 194 | 85.0 | 48.2 | 42.9 | 53.5 | 414 | 47.7 | 35.8 | 59.9 | 104 | 18.0 | 12.3 | 25.6 | 198 | 38.2 |
| South Africa | 1998 | 78.5 | 52.7 | 92.3 | 30 | 72.0 | 59.8 | 81.7 | 89 | 66.0 | 56.9 | 74.2 | 176 | 69.4 | 47.8 | 27.3 | 69.0 | 21 | 42.6 | 30.3 | 55.9 | 70 | 33.3 | 25.2 | 42.4 | 179 | 36.4 |
| South Africa | 2016 | 53.9 | 7.8 | 94.2 | 3 | 54.7 | 30.2 | 77.1 | 21 | 51.1 | 41.5 | 60.6 | 179 | 51.4 | 100.0 | - | - | 1 | 7.9 | 1.8 | 28.4 | 18 | 13.1 | 7.0 | 23.2 | 153 | 13.0 |
| Suriname | 2006 | 58.2 | 32.4 | 80.2 | 22 | 49.2 | 32.9 | 65.7 | 42 | 24.7 | 16.3 | 35.8 | 69 | 38.7 | 6.3 | 1.5 | 23.2 | 29 | 12.2 | 4.6 | 28.5 | 34 | 18.6 | 10.8 | 30.3 | 81 | 14.9 |
| Suriname | 2010 | 22.0 | 12.1 | 36.6 | 35 | 30.5 | 18.9 | 45.2 | 68 | 18.5 | 10.8 | 29.8 | 82 | 22.7 | 3.5 | 0.8 | 14.2 | 66 | 19.7 | 11.9 | 30.9 | 106 | 15.2 | 9.6 | 23.3 | 120 | 14.9 |
| Suriname | 2018 | 62.8 | 25.9 | 89.1 | 11 | 42.1 | 25.3 | 60.9 | 50 | 29.3 | 21.0 | 39.2 | 185 | 34.7 | 43.8 | 14.7 | 77.8 | 10 | 3.1 | 0.8 | 10.9 | 29 | 12.9 | 8.2 | 19.9 | 197 | 13.4 |
| Tajikistan | 2005 | 25.3 | 2.9 | 79.3 | 3 | 85.0 | 38.6 | 98.1 | 6 | 75.5 | 69.6 | 80.6 | 302 | 74.9 | 39.3 | 3.8 | 91.4 | 2 | 100.0 | - | - | 1 | 34.0 | 27.7 | 40.8 | 241 | 34.2 |
| Tajikistan | 2012 | 93.6 | 63.8 | 99.2 | 8 | 71.3 | 40.6 | 90.0 | 17 | 76.6 | 70.9 | 81.5 | 321 | 76.7 | 79.2 | 28.3 | 97.3 | 5 | 62.9 | 36.4 | 83.4 | 16 | 48.2 | 41.6 | 54.8 | 265 | 49.5 |
| Tajikistan | 2017 | 62.2 | 25.7 | 88.7 | 8 | 63.2 | 38.3 | 82.6 | 19 | 73.0 | 67.7 | 77.6 | 389 | 72.2 | 61.9 | 26.9 | 87.7 | 8 | 23.7 | 9.5 | 48.0 | 18 | 37.7 | 31.9 | 43.9 | 329 | 37.6 |
| Tanzania | 1996 | 96.1 | 89.4 | 98.7 | 111 | 93.7 | 90.2 | 96.1 | 318 | 83.8 | 61.2 | 94.4 | 29 | 93.9 | 65.1 | 54.9 | 74.1 | 100 | 56.9 | 49.2 | 64.3 | 241 | 30.1 | 12.5 | 56.4 | 19 | 58.1 |
| Tanzania | 2004 | 91.9 | 87.0 | 95.1 | 152 | 90.5 | 85.4 | 94.0 | 324 | 90.9 | 52.1 | 98.9 | 46 | 91.0 | 60.4 | 51.4 | 68.8 | 131 | 53.1 | 46.9 | 59.2 | 308 | 65.8 | 41.7 | 83.8 | 35 | 55.4 |
| Tanzania | 2010 | 94.0 | 86.5 | 97.4 | 128 | 94.3 | 90.6 | 96.6 | 321 | 91.2 | 73.3 | 97.5 | 59 | 94.0 | 55.4 | 42.9 | 67.2 | 120 | 49.3 | 41.8 | 56.8 | 266 | 50.1 | 30.4 | 69.8 | 47 | 51.0 |
| Tanzania | 2015 | 91.0 | 83.2 | 95.4 | 146 | 92.9 | 89.6 | 95.3 | 425 | 89.8 | 83.0 | 94.1 | 153 | 92.1 | 54.2 | 44.0 | 64.1 | 133 | 43.1 | 37.0 | 49.4 | 344 | 29.1 | 20.3 | 39.7 | 110 | 43.4 |
| Thailand | 2005 | 54.4 | 28.2 | 78.4 | 26 | 33.9 | 27.4 | 41.2 | 304 | 27.2 | 20.6 | 35.0 | 330 | 31.6 | 34.6 | 15.7 | 60.1 | 22 | 21.1 | 15.5 | 28.2 | 256 | 14.3 | 9.6 | 20.7 | 294 | 18.7 |
| Thailand | 2012 | 68.1 | 41.4 | 86.6 | 22 | 29.6 | 20.1 | 41.4 | 227 | 31.9 | 25.1 | 39.6 | 471 | 32.4 | 12.0 | 2.5 | 41.8 | 17 | 20.3 | 12.8 | 30.7 | 207 | 16.4 | 11.3 | 23.1 | 407 | 17.8 |
| Thailand | 2015 | 79.5 | 47.0 | 94.4 | 25 | 28.1 | 15.7 | 45.3 | 219 | 33.0 | 24.4 | 43.1 | 558 | 33.3 | 26.9 | 10.0 | 55.0 | 27 | 18.2 | 8.9 | 33.7 | 258 | 14.3 | 10.1 | 19.8 | 562 | 15.6 |
| Timor-Leste | 2009 | 78.0 | 70.5 | 84.1 | 203 | 71.2 | 63.5 | 77.9 | 182 | 64.3 | 57.7 | 70.4 | 246 | 70.6 | 41.1 | 33.1 | 49.7 | 152 | 35.2 | 26.5 | 44.9 | 118 | 26.1 | 19.1 | 34.7 | 174 | 33.4 |
| Timor-Leste | 2016 | 71.8 | 62.0 | 80.0 | 120 | 70.3 | 59.1 | 79.5 | 92 | 56.0 | 48.1 | 63.6 | 279 | 62.4 | 60.9 | 47.4 | 72.9 | 70 | 36.7 | 24.4 | 51.1 | 61 | 33.5 | 26.8 | 40.9 | 212 | 39.8 |
| Togo | 1998 | 99.0 | 96.8 | 99.7 | 254 | 97.8 | 85.3 | 99.7 | 102 | 94.1 | 67.4 | 99.2 | 27 | 98.3 | 82.8 | 76.0 | 88.0 | 234 | 73.1 | 64.3 | 80.3 | 126 | 67.0 | 46.1 | 82.8 | 22 | 78.6 |
| Togo | 2006 | 68.6 | 58.2 | 77.4 | 154 | 72.2 | 62.0 | 80.5 | 121 | 71.2 | 55.2 | 83.2 | 49 | 70.2 | 43.4 | 33.9 | 53.4 | 148 | 52.3 | 40.1 | 64.1 | 86 | 33.3 | 19.3 | 51.0 | 47 | 44.2 |
| Togo | 2010 | 92.4 | 83.9 | 96.6 | 181 | 92.1 | 84.4 | 96.2 | 102 | 93.8 | 78.5 | 98.4 | 40 | 92.5 | 68.8 | 59.1 | 77.0 | 157 | 65.7 | 54.7 | 75.3 | 98 | 46.8 | 30.5 | 63.8 | 44 | 63.8 |
| Togo | 2013 | 93.0 | 88.4 | 95.8 | 231 | 97.8 | 93.9 | 99.2 | 154 | 90.2 | 81.7 | 95.0 | 92 | 94.0 | 75.7 | 68.2 | 81.9 | 182 | 59.0 | 48.9 | 68.5 | 130 | 43.9 | 34.5 | 53.8 | 104 | 61.4 |
| Togo | 2017 | 96.5 | 90.6 | 98.8 | 109 | 89.9 | 79.6 | 95.3 | 127 | 92.4 | 84.9 | 96.3 | 109 | 92.8 | 78.8 | 68.3 | 86.5 | 121 | 47.6 | 34.7 | 60.8 | 113 | 44.0 | 31.4 | 57.5 | 67 | 58.7 |
| Tunisia | 2011 | 72.9 | 47.5 | 88.9 | 24 | 47.1 | 32.5 | 62.3 | 55 | 45.6 | 34.7 | 56.9 | 97 | 49.2 | 31.1 | 15.0 | 53.6 | 26 | 17.3 | 9.1 | 30.4 | 58 | 18.0 | 10.6 | 28.8 | 99 | 19.1 |
| Tunisia | 2018 | 74.3 | 47.5 | 90.3 | 15 | 55.8 | 41.3 | 69.4 | 48 | 39.0 | 30.3 | 48.5 | 135 | 45.4 | 42.7 | 21.8 | 66.6 | 18 | 20.4 | 10.6 | 35.6 | 47 | 15.2 | 10.1 | 22.1 | 169 | 18.2 |
| Turkey | 1993 | 59.4 | 47.4 | 70.4 | 78 | 48.3 | 40.7 | 56.0 | 135 | 23.7 | 12.8 | 39.6 | 43 | 47.5 | 37.8 | 22.1 | 56.6 | 45 | 12.2 | 6.8 | 21.1 | 101 | 3.3 | 0.4 | 21.5 | 32 | 17.9 |
| Turkey | 1998 | 74.3 | 61.3 | 84.0 | 56 | 52.0 | 42.0 | 61.8 | 157 | 32.9 | 19.3 | 50.0 | 60 | 51.6 | 33.4 | 16.3 | 56.4 | 26 | 24.0 | 14.2 | 37.6 | 102 | 19.4 | 8.5 | 38.5 | 42 | 24.1 |
| Turkey | 2003 | 61.4 | 47.8 | 73.4 | 78 | 55.2 | 45.4 | 64.6 | 124 | 50.5 | 35.9 | 65.0 | 69 | 55.2 | 31.8 | 17.3 | 50.9 | 42 | 25.3 | 16.6 | 36.4 | 107 | 17.6 | 9.1 | 31.4 | 58 | 24.3 |
| Turkey | 2013 | 80.4 | 58.7 | 92.2 | 29 | 73.0 | 60.8 | 82.5 | 85 | 62.8 | 52.6 | 72.0 | 127 | 68.5 | 28.1 | 14.6 | 47.2 | 29 | 37.7 | 24.4 | 53.2 | 78 | 32.6 | 23.0 | 43.9 | 88 | 34.2 |
| Turkmenistan | 2006 | . | . | . | . | 100.0 | - | - | 1 | 71.4 | 62.8 | 78.7 | 135 | 71.5 | 100.0 | - | - | 1 | 0.0 | - | - | 1 | 36.3 | 27.9 | 45.8 | 145 | 36.7 |
| Turkmenistan | 2015 | . | . | . | . | . | . | . | . | 64.1 | 57.1 | 70.6 | 270 | 64.1 | . | . | . | . | . | . | . | . | 19.5 | 14.8 | 25.4 | 240 | 19.5 |
| Uganda | 1995 | 91.2 | 83.6 | 95.4 | 130 | 88.8 | 84.0 | 92.4 | 295 | 83.5 | 72.6 | 90.7 | 72 | 89.0 | 63.8 | 55.0 | 71.7 | 128 | 37.6 | 30.3 | 45.6 | 229 | 27.8 | 15.6 | 44.5 | 48 | 45.8 |
| Uganda | 2000 | 90.2 | 82.5 | 94.7 | 104 | 89.8 | 85.1 | 93.1 | 260 | 86.0 | 74.9 | 92.6 | 77 | 89.3 | 57.5 | 47.2 | 67.1 | 98 | 46.7 | 39.0 | 54.5 | 238 | 52.4 | 36.8 | 67.5 | 53 | 50.0 |
| Uganda | 2006 | 93.2 | 86.1 | 96.8 | 115 | 92.3 | 88.5 | 94.9 | 337 | 82.6 | 72.6 | 89.5 | 73 | 91.1 | 62.9 | 52.2 | 72.4 | 99 | 53.7 | 47.7 | 59.6 | 304 | 45.2 | 32.1 | 59.1 | 57 | 54.4 |
| Uganda | 2011 | 92.4 | 81.9 | 97.0 | 71 | 88.8 | 83.7 | 92.5 | 266 | 78.9 | 69.0 | 86.3 | 120 | 86.9 | 61.9 | 44.8 | 76.5 | 58 | 47.0 | 39.5 | 54.6 | 259 | 34.2 | 23.3 | 47.0 | 93 | 45.8 |
| Uganda | 2016 | 96.3 | 88.0 | 98.9 | 93 | 87.6 | 84.2 | 90.4 | 556 | 82.7 | 76.3 | 87.7 | 234 | 86.9 | 62.2 | 51.6 | 71.7 | 110 | 45.1 | 40.3 | 49.9 | 522 | 33.1 | 26.6 | 40.3 | 208 | 43.2 |
| Ukraine | 2005 | . | . | . | . | . | . | . | . | 23.2 | 17.4 | 30.2 | 204 | 23.2 | . | . | . | . | . | . | . | . | 11.0 | 7.3 | 16.3 | 191 | 11.0 |
| Ukraine | 2007 | . | . | . | . | . | . | . | . | 25.6 | 16.7 | 37.2 | 85 | 25.6 | . | . | . | . | . | . | . | . | 6.5 | 2.4 | 16.3 | 50 | 6.5 |
| Ukraine | 2012 | . | . | . | . | . | . | . | . | 37.9 | 29.6 | 46.9 | 310 | 37.9 | . | . | . | . | . | . | . | . | 22.0 | 13.5 | 33.9 | 276 | 22.0 |
| Uzbekistan | 1996 | . | . | . | . | . | . | . | . | 68.8 | 60.1 | 76.3 | 147 | 68.8 | . | . | . | . | . | . | . | . | 38.5 | 28.4 | 49.7 | 128 | 38.5 |
| Uzbekistan | 2006 | . | . | . | . | . | . | . | . | 78.3 | 72.8 | 83.0 | 352 | 78.3 | . | . | . | . | . | . | . | . | 37.9 | 32.1 | 43.9 | 373 | 37.9 |
| Vietnam | 1997 | 93.9 | 63.6 | 99.3 | 17 | 82.0 | 58.9 | 93.6 | 44 | 80.8 | 71.1 | 87.8 | 126 | 82.3 | 37.8 | 15.5 | 66.9 | 10 | 32.5 | 17.7 | 52.0 | 69 | 20.7 | 13.7 | 30.0 | 157 | 25.0 |
| Vietnam | 2002 | 58.7 | 24.3 | 86.3 | 17 | 78.8 | 69.0 | 86.1 | 44 | 85.6 | 77.1 | 91.2 | 107 | 81.4 | 24.2 | 4.3 | 69.2 | 9 | 33.4 | 16.8 | 55.5 | 34 | 26.3 | 16.5 | 39.2 | 82 | 27.9 |
| Vietnam | 2006 | 89.8 | 65.5 | 97.6 | 24 | 79.3 | 67.0 | 87.8 | 79 | 74.2 | 63.7 | 82.5 | 97 | 77.7 | 54.7 | 31.2 | 76.3 | 19 | 21.3 | 13.4 | 32.1 | 80 | 19.3 | 12.1 | 29.4 | 86 | 22.9 |
| Vietnam | 2010 | 79.8 | 52.1 | 93.5 | 17 | 76.1 | 58.6 | 87.7 | 41 | 73.3 | 66.0 | 79.4 | 218 | 73.9 | 27.3 | 9.9 | 56.3 | 15 | 15.5 | 6.0 | 34.7 | 30 | 19.4 | 13.8 | 26.6 | 196 | 19.4 |
| Vietnam | 2013 | 90.1 | 64.4 | 97.9 | 15 | 76.7 | 55.0 | 89.8 | 30 | 62.8 | 55.4 | 69.6 | 220 | 65.6 | 43.0 | 20.2 | 69.2 | 20 | 16.9 | 7.3 | 34.6 | 42 | 21.1 | 14.7 | 29.3 | 184 | 21.8 |
| Zambia | 1996 | 94.1 | 85.1 | 97.8 | 65 | 96.6 | 94.2 | 98.1 | 327 | 93.8 | 88.1 | 96.9 | 103 | 95.7 | 59.8 | 49.5 | 69.4 | 56 | 48.1 | 40.8 | 55.5 | 263 | 34.9 | 25.5 | 45.7 | 74 | 47.1 |
| Zambia | 2001 | 98.1 | 91.7 | 99.6 | 74 | 96.0 | 93.1 | 97.7 | 270 | 97.9 | 89.8 | 99.6 | 91 | 96.8 | 53.4 | 41.4 | 65.0 | 73 | 66.6 | 59.6 | 73.0 | 251 | 40.1 | 30.9 | 50.1 | 85 | 58.4 |
| Zambia | 2007 | 93.6 | 83.5 | 97.7 | 57 | 95.2 | 90.9 | 97.6 | 263 | 89.2 | 76.7 | 95.4 | 91 | 93.8 | 59.7 | 42.9 | 74.5 | 43 | 40.9 | 34.0 | 48.2 | 267 | 34.1 | 24.8 | 44.7 | 87 | 41.7 |
| Zambia | 2013 | 92.0 | 81.5 | 96.8 | 91 | 94.9 | 91.8 | 96.9 | 471 | 88.2 | 82.4 | 92.2 | 301 | 92.2 | 62.8 | 50.5 | 73.6 | 79 | 48.5 | 42.8 | 54.2 | 430 | 24.3 | 18.8 | 30.9 | 263 | 41.8 |
| Zambia | 2018 | 89.1 | 74.8 | 95.8 | 65 | 92.6 | 87.2 | 95.8 | 334 | 86.7 | 80.3 | 91.2 | 237 | 89.8 | 41.1 | 28.3 | 55.2 | 61 | 32.7 | 26.2 | 40.0 | 323 | 24.6 | 16.4 | 35.1 | 219 | 30.4 |
| Zimbabwe | 1994 | 90.3 | 75.5 | 96.5 | 37 | 93.1 | 85.0 | 97.0 | 116 | 88.4 | 74.1 | 95.3 | 74 | 91.2 | 53.9 | 37.2 | 69.7 | 36 | 27.7 | 19.7 | 37.6 | 109 | 15.6 | 8.0 | 28.3 | 65 | 28.3 |
| Zimbabwe | 1999 | 91.6 | 57.1 | 98.9 | 15 | 95.5 | 90.7 | 97.9 | 124 | 94.9 | 88.8 | 97.7 | 108 | 95.0 | 59.2 | 38.0 | 77.4 | 23 | 45.5 | 33.8 | 57.7 | 96 | 25.9 | 17.5 | 36.4 | 96 | 37.3 |
| Zimbabwe | 2005 | 100.0 | - | - | 10 | 88.7 | 75.1 | 95.3 | 141 | 89.9 | 86.4 | 92.5 | 228 | 89.9 | 52.4 | 40.0 | 64.4 | 10 | 34.3 | 21.7 | 49.6 | 90 | 22.4 | 15.9 | 30.6 | 167 | 28.4 |
| Zimbabwe | 2009 | 57.9 | 31.7 | 80.3 | 16 | 86.7 | 78.6 | 92.0 | 154 | 82.8 | 77.6 | 86.9 | 299 | 83.2 | 43.1 | 21.3 | 67.9 | 19 | 26.3 | 19.9 | 33.8 | 193 | 15.4 | 11.1 | 20.8 | 265 | 20.5 |
| Zimbabwe | 2010 | 76.9 | 25.2 | 97.0 | 4 | 87.8 | 80.1 | 92.8 | 130 | 87.1 | 82.1 | 90.9 | 278 | 87.3 | 34.1 | 5.3 | 82.8 | 5 | 25.1 | 15.6 | 37.7 | 77 | 16.7 | 11.1 | 24.3 | 173 | 19.5 |
| Zimbabwe | 2014 | 61.4 | 30.5 | 85.2 | 12 | 83.0 | 76.3 | 88.1 | 201 | 85.8 | 81.7 | 89.0 | 428 | 84.4 | 20.4 | 6.8 | 47.5 | 24 | 20.5 | 15.3 | 27.0 | 236 | 15.0 | 11.8 | 19.0 | 437 | 17.1 |
| Zimbabwe | 2015 | 100.0 | - | - | 4 | 95.6 | 88.6 | 98.4 | 118 | 88.7 | 83.7 | 92.3 | 257 | 91.1 | 18.1 | 2.4 | 66.6 | 5 | 19.2 | 11.7 | 29.8 | 96 | 11.7 | 7.9 | 17.0 | 227 | 14.2 |
| Zimbabwe | 2019 | 49.8 | 9.0 | 90.8 | 4 | 88.1 | 80.3 | 93.0 | 106 | 81.8 | 75.7 | 86.7 | 287 | 83.1 | 12.0 | 1.6 | 54.1 | 7 | 16.4 | 10.2 | 25.2 | 109 | 11.3 | 7.6 | 16.4 | 271 | 12.8 |

^1^CAR: Central African Republic; ^2^CDR: Congo Democratic Republic.

**Supplementary table 4.** Percentage of children under 6 months and between 6-23 months of age who were fed formula by mother’s formal education level. Source: Demographic Health Survey and Multiple Indicator Cluster Survey.

| Country | Year | Consumption of formula under 6 months | | | | | | | | | | | | National prevalence (%) | Consumption of formula between 6-23 months | | | | | | | | | | | | National prevalence (%) |
| --- | --- | --- | --- | --- | --- | --- | --- | --- | --- | --- | --- | --- | --- | --- | --- | --- | --- | --- | --- | --- | --- | --- | --- | --- | --- | --- | --- |
|  |  | None | | | | Primary | | | | Secondary or higher | | | |  | None | | | | Primary | | | | Secondary or higher | | | |  |
|  |  | % | 95% CI | | N | % | 95% CI | | N | % | 95% CI | | N |  | % | 95% CI | | N | % | 95% CI | | N | % | 95% CI | | N |  |
| Afghanistan | 2010 | 15.1 | 12.2 | 18.4 | 1075 | 14.8 | 8.6 | 24.2 | 89 | 24.7 | 16.3 | 35.5 | 106 | 15.7 | 17.3 | 15.4 | 19.4 | 3166 | 27.7 | 22.1 | 34.1 | 242 | 40.0 | 31.3 | 49.4 | 227 | 19.0 |
| Afghanistan | 2015 | 9.3 | 7.3 | 11.7 | 2681 | 6.0 | 3.1 | 11.3 | 227 | 5.1 | 2.9 | 9.0 | 295 | 8.5 | 11.9 | 10.6 | 13.4 | 6805 | 10.5 | 6.9 | 15.8 | 594 | 17.0 | 12.2 | 23.2 | 679 | 12.4 |
| Albania | 2005 | . | . | . | . | . | . | . | . | 1.9 | 0.4 | 7.7 | 98 | 1.9 | 0.0 | - | - | 3 | 0.0 | - | - | 6 | 2.6 | 1.4 | 4.9 | 260 | 2.5 |
| Albania | 2008 | 0.0 | - | - | 2 | 13.9 | 6.4 | 27.7 | 86 | 21.7 | 12.9 | 34.1 | 50 | 16.3 | 13.7 | 1.6 | 60.9 | 6 | 10.5 | 6.2 | 17.0 | 228 | 19.9 | 13.1 | 29.0 | 152 | 13.9 |
| Albania | 2017 | 67.3 | 11.3 | 97.1 | 2 | 18.6 | 11.0 | 29.8 | 139 | 27.4 | 17.6 | 40.1 | 144 | 24.0 | 0.0 | - | - | 5 | 9.8 | 6.3 | 14.9 | 402 | 14.3 | 9.9 | 20.4 | 359 | 12.2 |
| Armenia | 2000 | . | . | . | . | 0.0 | - | - | 1 | 13.3 | 8.0 | 21.4 | 154 | 13.2 | . | . | . | . | . | . | . | . | 8.4 | 5.7 | 12.3 | 435 | 8.4 |
| Armenia | 2005 | . | . | . | . | 0.0 | - | - | 1 | 14.4 | 8.3 | 23.8 | 156 | 14.3 | . | . | . | . | 0.0 | - | - | 1 | 19.8 | 15.1 | 25.6 | 390 | 19.5 |
| Armenia | 2010 | . | . | . | . | . | . | . | . | 15.6 | 9.9 | 23.8 | 156 | 15.6 | . | . | . | . | . | . | . | . | 10.0 | 7.1 | 13.9 | 464 | 10.0 |
| Armenia | 2015 | . | . | . | . | 0.0 | - | - | 1 | 3.4 | 1.6 | 6.9 | 176 | 3.4 | . | . | . | . | 0.0 | - | - | 1 | 5.0 | 3.1 | 7.9 | 498 | 5.0 |
| Bangladesh | 1993 | 14.1 | 10.4 | 19.0 | 319 | 14.9 | 10.2 | 21.3 | 197 | 16.4 | 10.0 | 25.8 | 100 | 14.7 | 19.8 | 17.5 | 22.3 | 945 | 23.4 | 19.2 | 28.2 | 448 | 30.7 | 25.2 | 36.8 | 295 | 22.5 |
| Bangladesh | 1996 | 8.9 | 6.2 | 12.8 | 321 | 16.4 | 11.5 | 22.8 | 170 | 18.9 | 12.3 | 28.1 | 121 | 12.9 | 13.5 | 11.1 | 16.2 | 887 | 15.7 | 12.2 | 20.1 | 443 | 40.1 | 34.4 | 46.2 | 263 | 18.4 |
| Bangladesh | 1999 | 2.8 | 1.4 | 5.7 | 290 | 4.2 | 2.1 | 8.0 | 227 | 11.6 | 7.9 | 16.7 | 229 | 5.8 | 2.2 | 1.4 | 3.4 | 795 | 7.0 | 5.0 | 9.8 | 508 | 17.5 | 14.2 | 21.5 | 483 | 7.4 |
| Bangladesh | 2004 | 5.3 | 2.7 | 10.1 | 186 | 11.0 | 7.2 | 16.4 | 230 | 14.9 | 11.0 | 20.0 | 263 | 10.8 | 2.2 | 1.2 | 3.8 | 585 | 5.3 | 3.7 | 7.6 | 552 | 16.9 | 14.0 | 20.2 | 654 | 8.2 |
| Bangladesh | 2006 | 5.2 | 3.6 | 7.6 | 710 | 5.3 | 3.4 | 8.0 | 659 | 9.8 | 7.6 | 12.6 | 924 | 7.1 | 2.4 | 1.8 | 3.2 | 3071 | 4.2 | 3.4 | 5.2 | 2743 | 16.1 | 14.5 | 17.9 | 3611 | 8.2 |
| Bangladesh | 2007 | 6.2 | 2.4 | 15.1 | 108 | 5.2 | 2.4 | 11.2 | 152 | 13.6 | 9.2 | 19.5 | 246 | 9.4 | 4.9 | 2.8 | 8.4 | 395 | 5.2 | 3.4 | 7.9 | 505 | 17.5 | 14.5 | 21.0 | 813 | 11.0 |
| Bangladesh | 2011 | 6.7 | 3.2 | 13.6 | 126 | 8.9 | 5.3 | 14.6 | 217 | 14.1 | 10.7 | 18.3 | 446 | 11.4 | 1.0 | 0.4 | 2.4 | 376 | 3.9 | 2.6 | 6.0 | 696 | 9.3 | 7.6 | 11.3 | 1284 | 6.3 |
| Bangladesh | 2012 | 4.3 | 2.6 | 7.1 | 393 | 7.2 | 5.2 | 10.1 | 582 | 10.8 | 8.4 | 13.6 | 984 | 8.6 | 3.4 | 2.3 | 5.1 | 1284 | 6.3 | 4.8 | 8.2 | 1738 | 17.1 | 15.2 | 19.2 | 2946 | 11.5 |
| Bangladesh | 2014 | 12.4 | 5.1 | 27.1 | 84 | 10.9 | 6.5 | 17.6 | 165 | 9.1 | 5.9 | 13.9 | 383 | 10.1 | 2.6 | 1.1 | 6.0 | 296 | 2.2 | 1.1 | 4.3 | 646 | 10.2 | 8.1 | 12.6 | 1394 | 6.9 |
| Bangladesh | 2019 | 11.6 | 7.1 | 18.5 | 179 | 11.0 | 8.3 | 14.3 | 585 | 14.4 | 12.5 | 16.5 | 1606 | 13.3 | 5.7 | 3.9 | 8.3 | 681 | 6.1 | 4.8 | 7.8 | 1505 | 12.4 | 11.2 | 13.6 | 4505 | 10.3 |
| Belize | 2006 | 63.6 | 9.1 | 96.8 | 2 | 45.6 | 31.7 | 60.2 | 54 | 67.3 | 46.3 | 83.0 | 29 | 54.0 | 33.1 | 11.4 | 65.6 | 11 | 59.1 | 50.2 | 67.4 | 135 | 66.5 | 54.7 | 76.5 | 82 | 59.9 |
| Belize | 2011 | 45.6 | 16.8 | 77.6 | 7 | 52.8 | 40.7 | 64.5 | 63 | 65.4 | 52.2 | 76.6 | 70 | 57.4 | 21.4 | 10.0 | 40.0 | 36 | 30.2 | 24.8 | 36.2 | 299 | 47.6 | 40.7 | 54.6 | 258 | 37.0 |
| Belize | 2015 | 51.0 | 5.9 | 94.5 | 2 | 41.4 | 28.2 | 55.9 | 75 | 43.2 | 30.3 | 57.2 | 86 | 42.0 | 25.6 | 13.7 | 42.7 | 25 | 32.2 | 26.9 | 37.9 | 329 | 58.7 | 51.8 | 65.2 | 370 | 45.5 |
| Benin | 1996 | 2.3 | 1.1 | 4.7 | 423 | 6.4 | 1.9 | 19.0 | 74 | 14.6 | 5.3 | 34.3 | 21 | 3.5 | 3.3 | 2.2 | 5.0 | 1097 | 11.6 | 6.8 | 19.1 | 215 | 23.0 | 12.5 | 38.4 | 58 | 5.6 |
| Benin | 2001 | 22.6 | 18.3 | 27.6 | 384 | 17.3 | 11.6 | 25.1 | 99 | 21.7 | 11.5 | 37.1 | 44 | 21.5 | 19.4 | 16.3 | 23.0 | 1033 | 13.2 | 9.7 | 17.8 | 286 | 15.0 | 9.8 | 22.2 | 117 | 17.8 |
| Benin | 2006 | 12.4 | 10.5 | 14.8 | 1127 | 14.3 | 10.7 | 19.0 | 274 | 23.0 | 16.4 | 31.2 | 124 | 13.7 | 19.6 | 17.7 | 21.6 | 3385 | 23.6 | 20.4 | 27.2 | 847 | 25.5 | 21.5 | 30.1 | 361 | 20.8 |
| Benin | 2011 | 4.6 | 3.1 | 6.8 | 854 | 13.2 | 8.5 | 20.0 | 198 | 16.3 | 10.7 | 24.1 | 143 | 7.6 | 3.8 | 3.0 | 4.8 | 2691 | 8.9 | 6.4 | 12.2 | 593 | 19.8 | 16.1 | 24.2 | 455 | 6.8 |
| Benin | 2014 | 0.8 | 0.4 | 1.9 | 717 | 0.6 | 0.2 | 1.9 | 320 | 2.6 | 1.1 | 6.1 | 180 | 1.2 | 3.1 | 2.2 | 4.4 | 2118 | 4.1 | 2.7 | 6.2 | 832 | 6.2 | 4.0 | 9.4 | 457 | 4.3 |
| Benin | 2017 | 2.9 | 1.9 | 4.4 | 854 | 2.9 | 1.4 | 5.8 | 260 | 6.1 | 3.7 | 10.0 | 267 | 3.5 | 3.7 | 2.7 | 5.1 | 2446 | 4.7 | 3.3 | 6.7 | 717 | 9.8 | 7.6 | 12.4 | 721 | 5.0 |
| Bolivia | 1994 | 0.0 | - | - | 73 | 5.6 | 3.0 | 10.0 | 268 | 14.2 | 9.6 | 20.3 | 215 | 8.0 | 0.8 | 0.1 | 5.8 | 231 | 2.3 | 1.2 | 4.5 | 756 | 4.8 | 3.3 | 7.0 | 635 | 3.1 |
| Bolivia | 1998 | 2.6 | 0.8 | 8.0 | 79 | 7.2 | 4.2 | 11.9 | 289 | 18.7 | 14.0 | 24.5 | 261 | 12.0 | 2.3 | 0.9 | 6.0 | 250 | 5.5 | 4.0 | 7.5 | 975 | 11.8 | 9.1 | 15.1 | 759 | 7.7 |
| Bolivia | 2003 | 4.6 | 1.0 | 18.5 | 74 | 12.5 | 9.3 | 16.7 | 476 | 32.5 | 25.9 | 39.8 | 317 | 18.8 | 2.4 | 0.8 | 6.7 | 203 | 9.5 | 7.6 | 11.7 | 1574 | 22.6 | 19.0 | 26.7 | 926 | 13.3 |
| Bolivia | 2008 | 4.7 | 0.8 | 23.0 | 42 | 12.8 | 9.2 | 17.6 | 380 | 31.1 | 25.9 | 36.8 | 369 | 20.8 | 7.5 | 3.8 | 14.2 | 113 | 22.7 | 19.8 | 25.9 | 1273 | 43.9 | 40.1 | 47.8 | 1075 | 31.2 |
| Bosnia and Herzegovina | 2006 | 0.0 | - | - | 1 | 10.9 | 5.4 | 20.8 | 62 | 40.1 | 27.6 | 54.0 | 123 | 29.9 | 33.3 | 8.3 | 73.4 | 6 | 39.5 | 30.6 | 49.0 | 280 | 53.8 | 48.0 | 59.5 | 721 | 49.7 |
| Bosnia and Herzegovina | 2011 | 0.0 | - | - | 1 | 29.2 | 9.1 | 62.9 | 23 | 34.0 | 21.3 | 49.6 | 93 | 32.1 | 0.0 | - | - | 2 | 11.0 | 5.1 | 22.1 | 138 | 16.1 | 11.6 | 22.1 | 495 | 15.0 |
| Burkina Faso | 1998 | 0.7 | 0.3 | 1.9 | 548 | 1.6 | 0.2 | 10.1 | 41 | 13.4 | 4.0 | 36.7 | 29 | 1.1 | 0.4 | 0.2 | 0.8 | 1308 | 1.8 | 0.5 | 5.7 | 116 | 5.4 | 1.6 | 16.6 | 67 | 0.6 |
| Burkina Faso | 2003 | 5.1 | 3.5 | 7.2 | 911 | 3.2 | 1.2 | 8.1 | 110 | 7.0 | 1.9 | 22.5 | 50 | 5.0 | 3.6 | 2.9 | 4.6 | 2420 | 7.5 | 4.0 | 13.6 | 248 | 13.4 | 8.0 | 21.7 | 114 | 4.3 |
| Burkina Faso | 2006 | 0.2 | 0.0 | 1.4 | 483 | 0.0 | - | - | 42 | 5.3 | 0.7 | 30.8 | 18 | 0.4 | 0.5 | 0.2 | 1.0 | 1488 | 2.8 | 0.9 | 8.5 | 157 | 1.9 | 0.4 | 8.1 | 63 | 0.8 |
| Burkina Faso | 2010 | 0.3 | 0.1 | 1.0 | 1207 | 0.0 | - | - | 162 | 9.3 | 4.0 | 20.3 | 85 | 0.8 | 0.8 | 0.5 | 1.3 | 3398 | 2.8 | 1.4 | 5.6 | 480 | 20.5 | 13.7 | 29.6 | 269 | 2.2 |
| Burundi | 2010 | 0.0 | - | - | 307 | 0.3 | 0.0 | 2.4 | 301 | 5.4 | 2.3 | 12.1 | 77 | 0.5 | 0.4 | 0.2 | 1.0 | 1064 | 0.5 | 0.2 | 1.2 | 908 | 5.6 | 3.0 | 10.3 | 224 | 0.8 |
| Burundi | 2016 | 0.0 | - | - | 496 | 0.1 | 0.0 | 0.5 | 524 | 3.1 | 1.3 | 7.3 | 216 | 0.5 | 0.0 | - | - | 1626 | 0.4 | 0.2 | 0.9 | 1685 | 0.5 | 0.2 | 1.5 | 547 | 0.2 |
| CAR^1^ | 1994 | 0.0 | - | - | 221 | 1.7 | 0.9 | 3.1 | 189 | 8.3 | 3.2 | 19.5 | 48 | 1.6 | 0.6 | 0.2 | 1.8 | 606 | 1.6 | 0.7 | 3.3 | 440 | 4.6 | 2.7 | 7.7 | 169 | 1.5 |
| CAR^1^ | 2006 | 7.0 | 4.8 | 10.1 | 585 | 7.4 | 5.2 | 10.5 | 553 | 7.6 | 3.8 | 14.6 | 125 | 7.2 | 11.1 | 8.7 | 14.1 | 1167 | 10.8 | 8.4 | 13.8 | 1218 | 11.4 | 8.1 | 15.9 | 362 | 11.0 |
| CAR^1^ | 2010 | 3.4 | 2.0 | 5.6 | 556 | 7.1 | 4.6 | 10.8 | 587 | 7.4 | 3.7 | 14.4 | 140 | 5.6 | 4.7 | 3.5 | 6.2 | 1377 | 6.4 | 4.7 | 8.8 | 1524 | 18.3 | 13.2 | 24.8 | 365 | 7.5 |
| Cambodia | 2000 | 0.7 | 0.2 | 2.5 | 326 | 2.1 | 1.0 | 4.3 | 439 | 13.3 | 7.4 | 22.6 | 108 | 3.3 | 2.1 | 1.2 | 3.8 | 762 | 4.0 | 3.0 | 5.3 | 1063 | 13.7 | 9.1 | 20.1 | 274 | 4.9 |
| Cambodia | 2005 | 1.5 | 0.4 | 5.9 | 217 | 6.5 | 4.1 | 10.0 | 451 | 15.9 | 8.7 | 27.3 | 120 | 7.1 | 3.6 | 1.9 | 6.6 | 629 | 4.5 | 3.2 | 6.3 | 1337 | 17.3 | 12.6 | 23.2 | 363 | 6.5 |
| Cambodia | 2010 | 2.8 | 0.7 | 10.4 | 153 | 4.2 | 2.4 | 7.4 | 367 | 14.7 | 9.8 | 21.5 | 197 | 6.8 | 2.4 | 1.0 | 5.5 | 454 | 4.5 | 3.4 | 6.0 | 1241 | 23.7 | 19.7 | 28.3 | 673 | 9.3 |
| Cambodia | 2014 | 4.7 | 1.4 | 14.5 | 81 | 7.7 | 4.9 | 11.8 | 313 | 18.2 | 13.4 | 24.2 | 294 | 11.5 | 5.6 | 3.1 | 9.9 | 273 | 9.1 | 7.3 | 11.4 | 1025 | 22.3 | 18.9 | 26.1 | 829 | 13.4 |
| Cameroon | 1998 | 10.3 | 5.5 | 18.5 | 135 | 2.4 | 0.7 | 7.7 | 139 | 16.5 | 10.9 | 24.2 | 115 | 8.9 | 8.4 | 5.1 | 13.4 | 291 | 5.6 | 3.8 | 8.2 | 405 | 14.8 | 11.5 | 19.0 | 313 | 8.9 |
| Cameroon | 2004 | 2.3 | 0.8 | 6.0 | 196 | 7.3 | 4.8 | 10.9 | 339 | 13.1 | 9.5 | 18.0 | 259 | 7.7 | 5.3 | 3.3 | 8.5 | 538 | 4.8 | 3.6 | 6.5 | 984 | 17.6 | 14.7 | 20.9 | 628 | 8.5 |
| Cameroon | 2006 | 0.0 | - | - | 184 | 5.3 | 2.9 | 9.5 | 283 | 9.6 | 5.3 | 16.8 | 191 | 5.0 | 1.0 | 0.2 | 4.0 | 562 | 2.8 | 1.8 | 4.5 | 870 | 4.8 | 3.2 | 7.3 | 620 | 2.9 |
| Cameroon | 2011 | 1.8 | 0.8 | 4.0 | 276 | 5.8 | 3.8 | 8.7 | 460 | 11.8 | 8.6 | 15.8 | 387 | 6.6 | 3.8 | 2.1 | 6.8 | 751 | 3.8 | 2.8 | 5.0 | 1344 | 12.3 | 10.0 | 15.0 | 1192 | 6.8 |
| Cameroon | 2014 | 0.0 | - | - | 190 | 4.0 | 2.1 | 7.5 | 225 | 20.6 | 15.3 | 27.2 | 288 | 8.7 | 0.2 | 0.0 | 0.7 | 472 | 2.4 | 1.5 | 3.7 | 818 | 15.2 | 11.5 | 19.8 | 838 | 6.3 |
| Cameroon | 2018 | 0.8 | 0.2 | 3.7 | 220 | 2.6 | 1.2 | 5.6 | 310 | 16.4 | 12.6 | 20.9 | 456 | 8.1 | 1.7 | 0.9 | 3.1 | 595 | 5.7 | 4.0 | 8.1 | 822 | 21.7 | 18.9 | 24.8 | 1159 | 11.3 |
| Chad | 1996 | 4.8 | 3.3 | 6.9 | 600 | 8.7 | 5.5 | 13.4 | 164 | 10.5 | 4.1 | 24.2 | 43 | 5.7 | 8.8 | 7.1 | 10.7 | 1307 | 17.6 | 13.3 | 22.8 | 423 | 32.5 | 23.0 | 43.7 | 83 | 11.3 |
| Chad | 2004 | 1.0 | 0.4 | 2.4 | 456 | 0.4 | 0.0 | 2.8 | 94 | 0.9 | 0.1 | 6.6 | 33 | 0.9 | 4.3 | 2.9 | 6.4 | 968 | 4.1 | 2.4 | 6.9 | 282 | 15.9 | 10.4 | 23.5 | 121 | 4.9 |
| Chad | 2010 | 2.7 | 1.8 | 4.0 | 1339 | 4.5 | 2.3 | 8.6 | 363 | 10.1 | 5.6 | 17.4 | 165 | 3.7 | 4.2 | 3.3 | 5.3 | 3290 | 4.8 | 3.3 | 6.8 | 864 | 16.7 | 12.0 | 22.7 | 423 | 5.4 |
| Chad | 2014 | 3.2 | 2.3 | 4.4 | 1253 | 2.3 | 1.3 | 4.2 | 376 | 17.2 | 11.7 | 24.5 | 194 | 4.7 | 3.7 | 3.0 | 4.6 | 3016 | 2.8 | 1.7 | 4.6 | 944 | 9.1 | 6.5 | 12.5 | 443 | 4.1 |
| Colombia | 1995 | 18.4 | 7.4 | 38.9 | 25 | 28.0 | 21.4 | 35.6 | 168 | 52.1 | 45.5 | 58.7 | 247 | 41.2 | 4.6 | 1.5 | 13.5 | 61 | 9.0 | 6.9 | 11.7 | 632 | 19.7 | 16.8 | 22.9 | 761 | 14.5 |
| Colombia | 2000 | 0.0 | - | - | 12 | 26.9 | 20.2 | 34.8 | 169 | 50.0 | 43.5 | 56.4 | 249 | 39.9 | 18.6 | 9.8 | 32.6 | 44 | 29.2 | 25.2 | 33.6 | 532 | 45.1 | 41.4 | 48.9 | 759 | 38.1 |
| Colombia | 2005 | 8.4 | 2.8 | 22.6 | 61 | 21.9 | 17.4 | 27.3 | 397 | 34.5 | 30.3 | 39.1 | 939 | 30.3 | 21.0 | 14.2 | 30.0 | 150 | 29.3 | 26.0 | 32.8 | 1357 | 44.6 | 42.0 | 47.3 | 2603 | 38.7 |
| Colombia | 2010 | 26.7 | 10.1 | 54.3 | 45 | 27.9 | 21.8 | 35.0 | 399 | 37.4 | 33.7 | 41.3 | 1071 | 35.2 | 29.1 | 19.6 | 40.9 | 128 | 28.0 | 24.9 | 31.3 | 1408 | 47.2 | 45.0 | 49.4 | 3487 | 42.5 |
| Comoros | 1996 | 25.2 | 17.7 | 34.6 | 119 | 28.3 | 16.3 | 44.3 | 46 | 23.8 | 9.4 | 48.4 | 21 | 25.8 | 20.2 | 14.9 | 26.7 | 322 | 22.3 | 16.2 | 29.9 | 121 | 27.8 | 18.1 | 40.3 | 79 | 21.8 |
| Comoros | 2012 | 14.2 | 9.1 | 21.6 | 144 | 23.4 | 13.7 | 37.1 | 72 | 47.8 | 37.3 | 58.4 | 109 | 26.5 | 10.9 | 7.3 | 16.1 | 349 | 15.0 | 10.6 | 20.8 | 219 | 24.8 | 19.4 | 31.2 | 299 | 16.6 |
| CDR^2^ | 2007 | 10.8 | 7.3 | 15.8 | 225 | 18.4 | 13.3 | 24.9 | 401 | 18.1 | 12.1 | 26.2 | 281 | 16.4 | 22.5 | 16.9 | 29.4 | 534 | 20.7 | 16.7 | 25.5 | 1021 | 26.8 | 21.5 | 32.9 | 800 | 23.3 |
| CDR^2^ | 2010 | 0.7 | 0.2 | 3.1 | 305 | 2.0 | 0.8 | 4.8 | 510 | 8.5 | 4.8 | 14.5 | 456 | 3.8 | 1.4 | 0.7 | 2.7 | 788 | 1.1 | 0.6 | 1.8 | 1512 | 6.5 | 5.1 | 8.2 | 1317 | 2.9 |
| CDR^2^ | 2013 | 0.6 | 0.1 | 2.6 | 407 | 0.7 | 0.2 | 2.2 | 830 | 6.9 | 4.8 | 9.9 | 697 | 3.2 | 0.7 | 0.1 | 4.1 | 999 | 0.6 | 0.3 | 1.2 | 2212 | 5.8 | 4.2 | 7.9 | 1780 | 2.7 |
| CDR^2^ | 2017 | 2.7 | 1.2 | 5.8 | 481 | 2.8 | 1.1 | 7.2 | 809 | 7.8 | 5.2 | 11.6 | 816 | 5.2 | 2.6 | 1.1 | 6.4 | 1404 | 3.1 | 2.0 | 4.6 | 2562 | 7.5 | 5.7 | 10.0 | 2532 | 5.2 |
| Cote d'Ivoire | 1994 | 2.2 | 1.1 | 4.5 | 446 | 5.7 | 3.0 | 10.7 | 158 | 19.7 | 10.4 | 34.0 | 54 | 4.6 | 1.9 | 1.2 | 3.0 | 1134 | 4.4 | 2.7 | 7.0 | 431 | 3.7 | 1.6 | 8.2 | 145 | 2.7 |
| Cote d'Ivoire | 1998 | 8.5 | 4.0 | 17.3 | 102 | 22.8 | 10.8 | 41.9 | 50 | 52.0 | 26.5 | 76.5 | 14 | 14.8 | 19.1 | 14.1 | 25.3 | 369 | 15.3 | 9.7 | 23.5 | 151 | 35.3 | 22.4 | 50.8 | 63 | 19.3 |
| Cote d'Ivoire | 2006 | 3.3 | 1.9 | 5.7 | 582 | 4.0 | 1.7 | 8.9 | 261 | 14.4 | 7.5 | 25.9 | 97 | 4.8 | 6.8 | 5.2 | 8.7 | 1759 | 8.9 | 6.3 | 12.3 | 738 | 25.1 | 16.3 | 36.5 | 250 | 9.3 |
| Cote d'Ivoire | 2011 | 3.6 | 2.0 | 6.4 | 531 | 4.7 | 2.3 | 9.7 | 172 | 21.5 | 13.0 | 33.5 | 72 | 5.8 | 2.8 | 1.8 | 4.4 | 1396 | 4.7 | 3.0 | 7.1 | 529 | 22.0 | 15.7 | 29.9 | 220 | 5.4 |
| Cote d'Ivoire | 2016 | 4.0 | 2.1 | 7.8 | 677 | 6.4 | 2.4 | 15.6 | 193 | 11.4 | 5.9 | 20.9 | 111 | 5.6 | 3.5 | 2.5 | 4.9 | 1737 | 5.4 | 3.3 | 8.6 | 594 | 8.4 | 5.5 | 12.5 | 337 | 4.8 |
| Dominican Republic | 1996 | 22.8 | 11.3 | 40.5 | 43 | 42.1 | 35.1 | 49.4 | 238 | 62.5 | 53.8 | 70.4 | 134 | 47.8 | 1.6 | 0.5 | 5.1 | 147 | 4.4 | 2.9 | 6.6 | 655 | 9.5 | 6.6 | 13.4 | 403 | 6.2 |
| Dominican Republic | 1999 | 0.0 | - | - | 2 | 57.0 | . | . | 27 | 68.4 | . | . | 23 | 61.8 | 0.0 | - | - | 3 | 1.0 | . | . | 80 | 12.5 | . | . | 57 | 6.2 |
| Dominican Republic | 2002 | 26.0 | 14.5 | 42.2 | 58 | 41.4 | 35.0 | 48.0 | 525 | 49.0 | 41.6 | 56.5 | 420 | 44.6 | 1.4 | 0.4 | 4.5 | 175 | 3.5 | 2.5 | 5.0 | 1619 | 6.4 | 4.9 | 8.5 | 1304 | 4.8 |
| Dominican Republic | 2007 | 61.2 | 41.7 | 77.6 | 58 | 68.1 | 60.5 | 74.8 | 407 | 80.2 | 73.9 | 85.3 | 521 | 74.5 | 67.4 | 56.9 | 76.3 | 165 | 77.5 | 74.0 | 80.6 | 1219 | 86.0 | 83.0 | 88.6 | 1531 | 82.3 |
| Dominican Republic | 2013 | 6.1 | 0.8 | 35.1 | 12 | 37.1 | 24.2 | 52.2 | 84 | 36.4 | 28.5 | 45.0 | 211 | 35.6 | 6.2 | 1.9 | 18.4 | 35 | 7.1 | 2.9 | 16.5 | 336 | 10.9 | 7.6 | 15.2 | 677 | 9.6 |
| Dominican Republic | 2014 | 13.4 | 5.8 | 27.8 | 71 | 21.7 | 15.9 | 28.8 | 460 | 33.9 | 29.0 | 39.1 | 1123 | 30.0 | 8.5 | 4.5 | 15.5 | 247 | 10.0 | 8.1 | 12.3 | 1934 | 11.1 | 9.6 | 12.9 | 4052 | 10.7 |
| Egypt | 1995 | 3.0 | 1.7 | 5.3 | 525 | 4.7 | 2.2 | 9.8 | 208 | 12.8 | 9.2 | 17.6 | 405 | 7.0 | 4.1 | 3.0 | 5.4 | 1464 | 7.3 | 5.2 | 10.1 | 620 | 16.2 | 13.7 | 19.0 | 1098 | 9.2 |
| Egypt | 2000 | 2.3 | 1.3 | 4.3 | 465 | 3.9 | 1.9 | 7.8 | 159 | 9.6 | 7.2 | 12.7 | 577 | 6.0 | 5.2 | 3.8 | 7.1 | 1151 | 11.3 | 8.5 | 14.8 | 458 | 19.7 | 17.7 | 21.9 | 1518 | 13.1 |
| Egypt | 2005 | 2.4 | 0.8 | 6.9 | 403 | 3.6 | 0.9 | 13.7 | 144 | 7.1 | 4.2 | 11.6 | 703 | 5.2 | 2.8 | 1.5 | 5.2 | 1212 | 4.4 | 1.9 | 9.6 | 443 | 6.8 | 5.1 | 8.9 | 2236 | 5.3 |
| Egypt | 2008 | 3.0 | 1.5 | 5.8 | 300 | 3.1 | 0.8 | 11.6 | 97 | 5.6 | 4.0 | 7.8 | 733 | 4.8 | 2.9 | 1.9 | 4.4 | 820 | 1.7 | 0.6 | 4.6 | 338 | 5.3 | 4.3 | 6.5 | 2166 | 4.4 |
| Egypt | 2014 | 12.7 | 8.9 | 17.9 | 254 | 13.5 | 7.9 | 22.1 | 122 | 16.5 | 13.8 | 19.4 | 1111 | 15.6 | 3.1 | 2.0 | 4.8 | 712 | 6.0 | 3.8 | 9.3 | 391 | 4.8 | 4.1 | 5.7 | 3731 | 4.7 |
| Eswatini | 2006 | 17.0 | 7.1 | 35.6 | 31 | 10.6 | 5.1 | 20.8 | 82 | 36.5 | 28.9 | 44.7 | 145 | 26.3 | 1.7 | 0.2 | 11.3 | 58 | 13.6 | 9.4 | 19.2 | 285 | 24.2 | 20.6 | 28.3 | 424 | 18.6 |
| Eswatini | 2010 | 13.0 | 2.9 | 42.6 | 11 | 17.4 | 9.8 | 28.9 | 73 | 29.1 | 22.9 | 36.1 | 185 | 25.2 | 21.1 | 13.4 | 31.6 | 75 | 12.3 | 8.8 | 16.8 | 243 | 23.6 | 19.6 | 28.2 | 455 | 19.8 |
| Eswatini | 2014 | 18.6 | 4.2 | 54.0 | 10 | 12.2 | 5.7 | 24.3 | 54 | 15.8 | 9.5 | 25.2 | 170 | 15.1 | 16.3 | 7.7 | 31.4 | 60 | 14.8 | 10.3 | 20.6 | 243 | 23.3 | 19.2 | 28.0 | 486 | 20.3 |
| Ethiopia | 2011 | 1.0 | 0.2 | 4.2 | 788 | 3.3 | 1.4 | 7.5 | 330 | 19.3 | 9.1 | 36.2 | 69 | 2.6 | 0.7 | 0.3 | 1.4 | 1911 | 1.8 | 0.9 | 3.7 | 769 | 12.5 | 6.6 | 22.1 | 170 | 1.5 |
| Ethiopia | 2016 | 0.2 | 0.1 | 0.6 | 638 | 0.2 | 0.0 | 1.1 | 306 | 7.3 | 2.8 | 17.7 | 148 | 0.9 | 0.7 | 0.3 | 1.7 | 1682 | 2.0 | 0.9 | 4.6 | 783 | 6.5 | 3.6 | 11.5 | 357 | 1.6 |
| Gabon | 2000 | 10.9 | 3.8 | 27.2 | 23 | 21.2 | 15.6 | 28.1 | 237 | 32.9 | 26.1 | 40.5 | 191 | 26.3 | 20.2 | 11.0 | 34.3 | 71 | 19.8 | 15.8 | 24.6 | 565 | 36.3 | 32.3 | 40.4 | 547 | 28.4 |
| Gabon | 2012 | 23.8 | 8.5 | 51.3 | 36 | 49.3 | 39.0 | 59.7 | 244 | 74.1 | 67.0 | 80.2 | 351 | 65.3 | 25.1 | 15.0 | 38.9 | 98 | 18.7 | 13.1 | 26.1 | 690 | 41.5 | 36.4 | 46.8 | 931 | 34.5 |
| Gambia | 2005 | 11.4 | 8.7 | 14.7 | 550 | 5.7 | 2.4 | 12.9 | 87 | 10.8 | 6.6 | 17.4 | 140 | 9.8 | 14.8 | 12.6 | 17.3 | 1410 | 17.0 | 12.6 | 22.5 | 240 | 27.1 | 21.9 | 33.0 | 306 | 16.0 |
| Gambia | 2010 | 10.1 | 7.8 | 12.8 | 1073 | 16.3 | 9.6 | 26.3 | 126 | 21.2 | 15.1 | 29.1 | 213 | 13.2 | 8.9 | 7.4 | 10.5 | 2953 | 16.4 | 11.1 | 23.6 | 307 | 25.0 | 20.3 | 30.4 | 530 | 12.7 |
| Gambia | 2013 | 2.6 | 1.3 | 5.2 | 535 | 0.7 | 0.1 | 4.7 | 161 | 2.5 | 0.9 | 6.6 | 255 | 2.2 | 4.0 | 2.5 | 6.4 | 1545 | 2.4 | 1.1 | 5.1 | 331 | 7.0 | 4.5 | 10.6 | 548 | 4.6 |
| Gambia | 2018 | 7.8 | 4.8 | 12.3 | 487 | 1.8 | 0.7 | 4.6 | 166 | 3.9 | 1.8 | 8.1 | 256 | 5.4 | 2.4 | 1.6 | 3.6 | 1567 | 2.8 | 1.5 | 5.4 | 483 | 5.9 | 3.8 | 9.2 | 669 | 3.6 |
| Georgia | 2005 | . | . | . | . | . | . | . | . | 46.7 | 38.6 | 55.0 | 171 | 46.7 | . | . | . | . | 0.0 | - | - | 3 | 33.1 | 28.5 | 38.0 | 574 | 32.9 |
| Georgia | 2018 | 0.0 | - | - | 1 | 68.2 | 45.2 | 84.8 | 30 | 43.2 | 33.4 | 53.6 | 203 | 45.5 | 0.0 | - | - | 2 | 16.7 | 9.0 | 29.1 | 80 | 27.2 | 22.1 | 33.0 | 619 | 26.1 |
| Ghana | 1993 | 1.9 | 0.6 | 5.8 | 159 | 8.7 | 5.6 | 13.3 | 195 | 28.0 | 14.2 | 47.7 | 25 | 7.1 | 1.6 | 0.7 | 3.5 | 376 | 7.0 | 5.1 | 9.5 | 532 | 13.3 | 6.6 | 24.9 | 60 | 5.3 |
| Ghana | 1998 | 4.6 | 1.6 | 12.8 | 142 | 13.8 | 6.6 | 26.6 | 56 | 7.0 | 3.4 | 13.7 | 104 | 7.4 | 7.0 | 4.6 | 10.5 | 423 | 11.3 | 7.4 | 16.9 | 177 | 14.3 | 10.9 | 18.5 | 350 | 11.0 |
| Ghana | 2003 | 1.0 | 0.1 | 6.7 | 177 | 3.0 | 0.6 | 12.6 | 64 | 7.1 | 3.3 | 14.9 | 97 | 3.5 | 3.8 | 2.4 | 6.1 | 512 | 4.5 | 2.4 | 8.3 | 230 | 13.8 | 10.2 | 18.5 | 360 | 7.8 |
| Ghana | 2006 | 1.1 | 0.2 | 7.6 | 165 | 5.9 | 1.9 | 17.0 | 72 | 14.4 | 9.2 | 21.7 | 147 | 8.1 | 1.5 | 0.5 | 4.4 | 491 | 5.8 | 3.1 | 10.8 | 212 | 22.3 | 16.9 | 28.7 | 340 | 10.7 |
| Ghana | 2008 | 2.7 | 0.7 | 10.8 | 104 | 4.1 | 1.3 | 11.9 | 82 | 8.7 | 4.7 | 15.5 | 132 | 5.7 | 2.7 | 1.1 | 6.7 | 315 | 7.2 | 3.7 | 13.6 | 201 | 15.4 | 11.8 | 19.7 | 341 | 9.4 |
| Ghana | 2011 | 3.2 | 1.0 | 10.1 | 382 | 10.8 | 5.0 | 21.9 | 150 | 17.0 | 11.9 | 23.7 | 260 | 11.7 | 0.6 | 0.2 | 1.5 | 1123 | 1.7 | 0.6 | 4.6 | 419 | 9.6 | 6.5 | 13.9 | 629 | 5.2 |
| Ghana | 2014 | 2.4 | 0.8 | 6.8 | 205 | 7.1 | 3.1 | 15.4 | 128 | 14.6 | 9.8 | 21.1 | 273 | 9.7 | 0.5 | 0.2 | 1.7 | 561 | 3.3 | 1.4 | 7.4 | 329 | 7.2 | 5.3 | 9.8 | 766 | 4.7 |
| Ghana | 2017 | 7.4 | 3.5 | 14.8 | 233 | 19.6 | 14.5 | 25.9 | 482 | 24.6 | 16.4 | 35.1 | 176 | 17.8 | 2.6 | 1.4 | 4.7 | 713 | 3.4 | 2.3 | 5.0 | 1414 | 18.1 | 13.4 | 24.0 | 458 | 5.8 |
| Guatemala | 1995 | 6.8 | 4.2 | 10.7 | 401 | 9.6 | 7.0 | 13.0 | 475 | 21.4 | 13.1 | 33.0 | 93 | 10.0 | 3.5 | 2.3 | 5.3 | 1189 | 6.3 | 4.6 | 8.6 | 1390 | 16.8 | 11.0 | 24.9 | 241 | 6.6 |
| Guatemala | 1998 | 1.6 | 0.6 | 4.2 | 176 | 6.1 | 3.3 | 11.1 | 249 | 56.5 | 37.9 | 73.4 | 51 | 14.0 | 1.7 | 0.3 | 8.2 | 500 | 3.1 | 1.6 | 6.0 | 699 | 10.0 | 3.9 | 23.4 | 125 | 3.7 |
| Guatemala | 2014 | 5.6 | 2.9 | 10.7 | 179 | 8.9 | 6.5 | 11.9 | 588 | 44.1 | 39.1 | 49.4 | 408 | 20.9 | 1.7 | 0.9 | 3.2 | 573 | 4.2 | 3.1 | 5.5 | 1846 | 17.2 | 14.5 | 20.2 | 1090 | 7.8 |
| Guinea | 1999 | 2.6 | 1.5 | 4.5 | 573 | 9.8 | 4.4 | 20.3 | 58 | 16.1 | 5.5 | 38.6 | 31 | 3.8 | 1.9 | 1.2 | 3.0 | 1095 | 10.4 | 6.0 | 17.5 | 124 | 14.2 | 8.8 | 22.2 | 102 | 3.5 |
| Guinea | 2005 | 4.8 | 3.4 | 6.9 | 638 | 6.4 | 2.6 | 14.9 | 68 | 9.0 | 2.2 | 30.2 | 27 | 5.2 | 14.9 | 12.8 | 17.2 | 1428 | 15.2 | 9.3 | 23.7 | 130 | 35.8 | 25.4 | 47.8 | 92 | 16.1 |
| Guinea | 2012 | 4.2 | 2.7 | 6.5 | 548 | 4.2 | 1.4 | 12.0 | 92 | 25.0 | 16.1 | 36.6 | 78 | 6.7 | 3.4 | 2.5 | 4.6 | 1478 | 7.7 | 4.5 | 12.8 | 259 | 14.8 | 9.9 | 21.5 | 216 | 5.2 |
| Guinea | 2016 | 5.3 | 3.5 | 8.0 | 509 | 8.0 | 3.3 | 18.1 | 92 | 16.1 | 9.3 | 26.3 | 85 | 7.2 | 6.4 | 5.0 | 8.2 | 1499 | 9.6 | 5.9 | 15.2 | 302 | 18.2 | 14.4 | 22.8 | 321 | 8.8 |
| Guinea | 2018 | 5.2 | 3.6 | 7.5 | 706 | 11.5 | 6.1 | 20.5 | 103 | 22.1 | 14.7 | 31.9 | 107 | 7.9 | 5.9 | 4.6 | 7.5 | 1396 | 15.9 | 11.2 | 22.2 | 238 | 26.4 | 19.9 | 34.1 | 275 | 10.1 |
| Guinea Bissau | 2006 | 4.2 | 2.6 | 7.0 | 442 | 7.8 | 4.2 | 13.9 | 143 | 8.3 | 3.3 | 19.2 | 62 | 5.2 | 11.3 | 9.3 | 13.5 | 1260 | 22.8 | 17.7 | 28.9 | 394 | 29.7 | 22.3 | 38.3 | 155 | 15.1 |
| Guinea Bissau | 2014 | 1.4 | 0.5 | 3.5 | 488 | 0.5 | 0.1 | 1.9 | 265 | 6.1 | 1.8 | 18.6 | 77 | 1.8 | 8.6 | 6.5 | 11.3 | 1240 | 8.5 | 6.4 | 11.2 | 746 | 26.6 | 20.4 | 33.8 | 282 | 11.5 |
| Guyana | 2006 | . | . | . | . | 43.3 | 27.8 | 60.3 | 50 | 42.6 | 33.9 | 51.8 | 161 | 43.0 | 0.0 | - | - | 1 | 39.9 | 30.0 | 50.6 | 171 | 34.4 | 29.0 | 40.3 | 513 | 35.6 |
| Guyana | 2009 | 100.0 | - | - | 2 | 30.4 | 16.9 | 48.5 | 59 | 26.6 | 18.1 | 37.2 | 169 | 28.4 | 12.0 | 2.5 | 42.0 | 21 | 27.1 | 18.1 | 38.5 | 144 | 43.4 | 37.4 | 49.6 | 442 | 38.8 |
| Guyana | 2014 | 0.0 | - | - | 3 | 45.5 | 23.7 | 69.1 | 43 | 45.6 | 37.5 | 54.0 | 244 | 45.3 | 6.1 | 1.3 | 24.9 | 27 | 13.8 | 8.9 | 20.8 | 161 | 26.1 | 21.9 | 30.7 | 845 | 24.1 |
| Haiti | 1994 | 9.4 | 5.4 | 15.9 | 139 | 16.0 | 10.5 | 23.5 | 134 | 52.3 | 38.5 | 65.7 | 44 | 17.7 | 2.6 | 1.5 | 4.5 | 442 | 7.4 | 5.2 | 10.4 | 372 | 10.8 | 5.9 | 18.9 | 117 | 5.5 |
| Haiti | 2000 | 10.8 | 6.5 | 17.3 | 208 | 19.2 | 11.2 | 30.9 | 267 | 52.6 | 39.7 | 65.2 | 88 | 22.7 | 6.1 | 3.8 | 9.8 | 700 | 14.0 | 9.5 | 20.2 | 832 | 30.0 | 21.5 | 40.3 | 265 | 13.5 |
| Haiti | 2005 | 4.0 | 1.4 | 10.9 | 171 | 15.6 | 9.3 | 25.0 | 274 | 21.4 | 13.6 | 32.1 | 153 | 14.0 | 5.0 | 3.0 | 8.2 | 576 | 5.1 | 3.3 | 7.9 | 734 | 13.8 | 10.4 | 18.0 | 381 | 7.3 |
| Haiti | 2012 | 6.3 | 2.7 | 13.9 | 114 | 13.0 | 9.0 | 18.3 | 353 | 29.9 | 23.1 | 37.7 | 259 | 18.5 | 3.3 | 1.9 | 5.5 | 421 | 8.8 | 6.6 | 11.6 | 890 | 31.0 | 26.3 | 36.0 | 661 | 15.9 |
| Haiti | 2016 | 4.4 | 1.6 | 11.7 | 103 | 14.0 | 9.8 | 19.6 | 292 | 27.2 | 21.4 | 34.0 | 305 | 18.9 | 4.6 | 2.6 | 7.9 | 316 | 8.2 | 6.0 | 11.2 | 683 | 27.7 | 23.6 | 32.1 | 653 | 15.6 |
| Honduras | 2005 | 12.2 | 5.4 | 25.2 | 82 | 30.5 | 26.0 | 35.4 | 706 | 56.0 | 47.6 | 64.1 | 185 | 34.5 | 11.7 | 7.9 | 17.0 | 319 | 15.3 | 13.1 | 17.8 | 2162 | 34.0 | 29.4 | 38.9 | 585 | 19.4 |
| Honduras | 2011 | 2.9 | 0.9 | 9.0 | 45 | 18.9 | 15.4 | 23.0 | 685 | 39.6 | 33.4 | 46.0 | 354 | 26.1 | 0.3 | 0.0 | 1.9 | 178 | 2.3 | 1.6 | 3.4 | 1960 | 8.2 | 6.2 | 10.7 | 1099 | 4.5 |
| India | 2005 | 1.6 | 1.0 | 2.5 | 1806 | 3.4 | 2.1 | 5.4 | 693 | 7.0 | 5.8 | 8.5 | 2117 | 4.0 | 4.8 | 4.0 | 5.9 | 5233 | 8.7 | 7.2 | 10.5 | 1908 | 17.2 | 15.9 | 18.4 | 6762 | 10.2 |
| India | 2015 | 1.8 | 1.3 | 2.3 | 6077 | 3.3 | 2.1 | 5.1 | 3137 | 4.6 | 4.1 | 5.2 | 13412 | 3.7 | 5.8 | 5.4 | 6.4 | 20270 | 7.6 | 6.8 | 8.3 | 9963 | 13.5 | 12.9 | 14.0 | 41529 | 10.6 |
| Indonesia | 2002 | 3.8 | 1.4 | 9.8 | 64 | 14.1 | 10.5 | 18.7 | 711 | 30.3 | 25.5 | 35.6 | 866 | 21.3 | 11.9 | 6.7 | 20.4 | 173 | 20.3 | 17.1 | 24.0 | 1921 | 44.1 | 40.7 | 47.6 | 2319 | 32.2 |
| Indonesia | 2007 | 10.4 | 3.9 | 25.3 | 66 | 21.6 | 17.0 | 27.2 | 648 | 43.6 | 38.6 | 48.7 | 1088 | 34.8 | 18.8 | 11.3 | 29.5 | 151 | 25.2 | 22.3 | 28.4 | 1963 | 47.9 | 45.1 | 50.7 | 3094 | 38.3 |
| Indonesia | 2012 | 29.6 | 14.3 | 51.5 | 63 | 20.4 | 15.6 | 26.3 | 449 | 36.1 | 32.3 | 40.1 | 1174 | 31.7 | 8.8 | 4.8 | 15.4 | 140 | 22.6 | 19.7 | 25.8 | 1485 | 44.7 | 42.2 | 47.4 | 3568 | 37.5 |
| Indonesia | 2017 | 1.8 | 0.2 | 12.4 | 19 | 18.9 | 14.5 | 24.3 | 384 | 32.3 | 29.1 | 35.8 | 1263 | 28.9 | 10.1 | 4.2 | 22.3 | 62 | 20.8 | 17.9 | 24.0 | 1107 | 42.0 | 40.0 | 44.0 | 3864 | 36.9 |
| Iraq | 2006 | 23.1 | 17.7 | 29.4 | 322 | 23.0 | 19.5 | 27.0 | 785 | 32.6 | 27.5 | 38.2 | 509 | 25.9 | 23.9 | 20.7 | 27.3 | 1072 | 25.0 | 22.8 | 27.3 | 2611 | 32.1 | 29.1 | 35.2 | 1641 | 26.9 |
| Iraq | 2011 | 35.6 | 31.2 | 40.3 | 977 | 41.0 | 37.6 | 44.5 | 1945 | 51.3 | 46.2 | 56.3 | 960 | 43.5 | 29.0 | 26.4 | 31.7 | 2657 | 32.0 | 29.9 | 34.1 | 5567 | 44.2 | 41.5 | 47.0 | 2944 | 35.5 |
| Iraq | 2018 | 34.0 | 27.5 | 41.3 | 333 | 41.7 | 37.0 | 46.5 | 715 | 51.6 | 46.6 | 56.6 | 633 | 44.5 | 42.7 | 35.7 | 50.0 | 1000 | 41.1 | 37.3 | 45.0 | 2078 | 52.5 | 46.5 | 58.4 | 1708 | 45.8 |
| Jamaica | 2005 | . | . | . | . | 75.9 | 22.8 | 97.1 | 4 | 62.2 | 52.7 | 70.8 | 123 | 63.1 | . | . | . | . | 62.9 | 37.3 | 82.8 | 18 | 62.8 | 57.5 | 67.8 | 387 | 62.5 |
| Jamaica | 2011 | . | . | . | . | 48.8 | 11.5 | 87.5 | 4 | 53.0 | 44.0 | 61.8 | 163 | 52.9 | . | . | . | . | 37.6 | 11.9 | 72.9 | 10 | 58.4 | 52.2 | 64.4 | 465 | 58.2 |
| Jordan | 1997 | 27.7 | 14.3 | 46.8 | 32 | 34.1 | 22.1 | 48.6 | 50 | 30.7 | 26.5 | 35.2 | 417 | 30.9 | 4.3 | 1.8 | 10.3 | 134 | 7.5 | 4.5 | 12.4 | 208 | 9.5 | 8.0 | 11.3 | 1425 | 9.0 |
| Jordan | 2002 | 35.5 | 14.0 | 65.0 | 30 | 13.6 | 6.2 | 27.2 | 44 | 32.7 | 28.0 | 37.9 | 433 | 31.4 | 9.4 | 4.4 | 19.2 | 90 | 8.1 | 4.0 | 15.6 | 167 | 12.5 | 10.5 | 14.8 | 1471 | 12.0 |
| Jordan | 2007 | 58.6 | 36.7 | 77.6 | 53 | 37.4 | 21.7 | 56.2 | 70 | 48.9 | 43.3 | 54.5 | 994 | 48.6 | 24.3 | 15.6 | 35.7 | 121 | 33.2 | 23.7 | 44.3 | 190 | 42.3 | 39.1 | 45.6 | 2428 | 41.4 |
| Jordan | 2012 | 0.0 | - | - | 17 | 27.5 | 9.7 | 57.2 | 45 | 5.2 | 2.9 | 9.1 | 788 | 6.3 | 1.3 | 0.2 | 8.7 | 78 | 3.4 | 1.3 | 8.4 | 183 | 9.2 | 7.3 | 11.4 | 2522 | 8.7 |
| Jordan | 2017 | 38.3 | 14.6 | 69.2 | 35 | 46.2 | 32.2 | 60.9 | 95 | 50.0 | 45.5 | 54.4 | 1088 | 49.5 | 16.3 | 7.7 | 31.2 | 53 | 36.6 | 26.9 | 47.6 | 185 | 47.9 | 44.6 | 51.2 | 2442 | 46.8 |
| Kazakhstan | 1995 | . | . | . | . | . | . | . | . | 18.7 | 10.8 | 30.4 | 118 | 18.7 | . | . | . | . | 0.0 | - | - | 2 | 10.6 | 7.5 | 14.9 | 401 | 10.6 |
| Kazakhstan | 1999 | 60.8 | 7.1 | 96.9 | 2 | . | . | . | . | 7.2 | 3.1 | 15.6 | 97 | 8.2 | . | . | . | . | . | . | . | . | 6.8 | 4.3 | 10.4 | 349 | 6.8 |
| Kazakhstan | 2006 | 100.0 | - | - | 1 | . | . | . | . | 22.7 | 18.0 | 28.1 | 386 | 22.9 | 38.2 | 3.7 | 90.9 | 2 | 0.0 | - | - | 3 | 31.2 | 28.1 | 34.5 | 1419 | 31.2 |
| Kazakhstan | 2015 | 0.0 | - | - | 1 | . | . | . | . | 19.8 | 15.5 | 25.0 | 506 | 20.0 | 0.0 | - | - | 1 | . | . | . | . | 12.8 | 10.9 | 14.9 | 1611 | 12.8 |
| Kenya | 1993 | 0.9 | 0.2 | 3.8 | 86 | 2.9 | 1.6 | 5.4 | 319 | 1.3 | 0.4 | 4.3 | 113 | 2.2 | 2.3 | 0.9 | 5.6 | 271 | 2.9 | 1.8 | 4.5 | 1015 | 7.2 | 4.9 | 10.5 | 359 | 3.8 |
| Kenya | 1998 | 1.8 | 0.4 | 7.7 | 44 | 2.6 | 1.2 | 5.5 | 330 | 8.2 | 4.3 | 14.9 | 144 | 4.2 | 2.2 | 0.6 | 7.3 | 189 | 1.9 | 1.1 | 3.2 | 1060 | 7.9 | 5.2 | 11.8 | 375 | 3.4 |
| Kenya | 2003 | 6.4 | 2.7 | 14.6 | 126 | 3.8 | 2.0 | 7.3 | 358 | 6.9 | 2.9 | 15.5 | 115 | 4.8 | 4.7 | 2.4 | 8.9 | 301 | 3.7 | 2.6 | 5.3 | 952 | 6.0 | 4.0 | 8.8 | 357 | 4.3 |
| Kenya | 2008 | 0.3 | 0.0 | 1.9 | 125 | 0.5 | 0.1 | 2.2 | 311 | 8.5 | 4.2 | 16.6 | 151 | 2.5 | 3.6 | 0.7 | 16.2 | 318 | 1.7 | 1.0 | 2.8 | 970 | 6.2 | 3.7 | 10.1 | 368 | 3.0 |
| Kenya | 2014 | 0.8 | 0.2 | 2.9 | 387 | 0.2 | 0.0 | 1.1 | 943 | 0.2 | 0.1 | 0.8 | 480 | 0.3 | 1.7 | 1.0 | 3.0 | 1262 | 1.8 | 1.2 | 2.9 | 2966 | 4.5 | 3.2 | 6.3 | 1564 | 2.7 |
| Kyrgyzstan | 1997 | . | . | . | . | 0.0 | - | - | 1 | 14.2 | 8.8 | 22.1 | 176 | 14.1 | 100.0 | - | - | 1 | . | . | . | . | 24.8 | 20.3 | 29.9 | 520 | 24.9 |
| Kyrgyzstan | 2005 | . | . | . | . | . | . | . | . | 22.6 | 15.9 | 30.9 | 286 | 22.6 | . | . | . | . | 0.0 | - | - | 1 | 27.7 | 22.8 | 33.1 | 873 | 27.6 |
| Kyrgyzstan | 2012 | . | . | . | . | 0.0 | - | - | 2 | 6.8 | 4.5 | 10.1 | 445 | 6.7 | 0.0 | - | - | 1 | 0.0 | - | - | 3 | 8.8 | 7.0 | 11.1 | 1313 | 8.8 |
| Kyrgyzstan | 2014 | 0.0 | - | - | 2 | . | . | . | . | 7.9 | 5.3 | 11.7 | 429 | 7.9 | 20.0 | . | . | 5 | 0.0 | - | - | 4 | 11.3 | 9.3 | 13.7 | 1355 | 11.2 |
| Kyrgyzstan | 2018 | . | . | . | . | . | . | . | . | 13.6 | 9.8 | 18.6 | 390 | 13.6 | . | . | . | . | 0.0 | - | - | 2 | 17.1 | 14.2 | 20.4 | 990 | 17.0 |
| Lao | 2006 | 1.3 | 0.3 | 6.0 | 171 | 8.9 | 4.9 | 15.7 | 186 | 11.2 | 5.6 | 21.1 | 86 | 6.3 | 1.1 | 0.5 | 2.4 | 461 | 2.6 | 1.4 | 4.7 | 543 | 24.8 | 17.2 | 34.5 | 183 | 5.6 |
| Lao | 2011 | 2.5 | 1.1 | 5.3 | 369 | 9.4 | 6.6 | 13.3 | 454 | 16.7 | 12.4 | 22.3 | 345 | 9.7 | 6.7 | 5.0 | 9.1 | 1000 | 17.6 | 15.2 | 20.2 | 1385 | 41.5 | 37.8 | 45.4 | 880 | 21.4 |
| Lao | 2017 | 4.5 | 2.1 | 9.4 | 229 | 13.7 | 9.9 | 18.7 | 435 | 22.8 | 18.3 | 28.1 | 470 | 15.9 | 8.9 | 6.6 | 11.9 | 682 | 15.1 | 12.6 | 17.9 | 1288 | 28.4 | 25.5 | 31.5 | 1458 | 19.7 |
| Lesotho | 2004 | 0.0 | - | - | 10 | 4.8 | 2.8 | 8.2 | 274 | 12.4 | 7.5 | 19.9 | 115 | 7.1 | 5.4 | 0.8 | 28.1 | 24 | 11.4 | 9.1 | 14.3 | 632 | 22.2 | 18.1 | 26.9 | 326 | 15.1 |
| Lesotho | 2009 | 21.4 | 4.8 | 59.5 | 8 | 10.2 | 6.6 | 15.3 | 254 | 23.4 | 16.7 | 31.8 | 172 | 16.0 | 8.0 | 2.1 | 26.0 | 24 | 8.7 | 6.1 | 12.3 | 651 | 14.6 | 11.0 | 19.1 | 443 | 11.3 |
| Lesotho | 2014 | 0.0 | - | - | 2 | 7.4 | 3.6 | 14.8 | 145 | 25.3 | 17.0 | 36.0 | 180 | 17.4 | 0.0 | - | - | 7 | 5.9 | 3.3 | 10.4 | 435 | 19.3 | 15.5 | 23.6 | 506 | 13.3 |
| Lesotho | 2018 | . | . | . | . | 13.5 | 6.8 | 25.0 | 79 | 23.8 | 15.7 | 34.3 | 136 | 20.0 | 4.3 | 0.6 | 26.5 | 13 | 9.6 | 6.0 | 15.1 | 418 | 20.4 | 16.7 | 24.7 | 578 | 16.3 |
| Liberia | 2007 | 3.4 | 1.3 | 8.3 | 238 | 8.6 | 4.7 | 15.4 | 196 | 28.5 | 17.0 | 43.7 | 69 | 9.2 | 4.0 | 2.3 | 6.6 | 677 | 6.2 | 3.9 | 9.7 | 582 | 20.1 | 15.0 | 26.5 | 259 | 7.8 |
| Liberia | 2013 | 1.6 | 0.7 | 3.5 | 298 | 3.7 | 1.3 | 10.4 | 272 | 11.9 | 6.2 | 21.7 | 148 | 5.1 | 3.5 | 1.8 | 6.6 | 903 | 3.2 | 1.5 | 6.6 | 784 | 11.1 | 7.1 | 17.0 | 468 | 5.7 |
| Madagascar | 1997 | 0.0 | - | - | 153 | 0.8 | 0.2 | 3.2 | 346 | 4.3 | 2.1 | 8.8 | 129 | 1.3 | 1.1 | 0.6 | 2.0 | 372 | 2.3 | 1.3 | 3.7 | 900 | 5.4 | 3.5 | 8.1 | 366 | 2.6 |
| Madagascar | 2003 | 0.1 | 0.0 | 0.8 | 114 | 0.0 | 0.0 | 0.3 | 247 | 1.1 | 0.3 | 4.5 | 138 | 0.2 | 1.2 | 0.4 | 3.5 | 319 | 2.8 | 1.5 | 5.0 | 728 | 13.4 | 8.0 | 21.5 | 491 | 4.8 |
| Madagascar | 2008 | 7.5 | 4.6 | 11.9 | 370 | 4.1 | 2.4 | 6.7 | 598 | 4.4 | 2.4 | 7.7 | 246 | 5.1 | 10.0 | 7.9 | 12.7 | 936 | 8.6 | 6.8 | 10.7 | 1685 | 17.4 | 13.7 | 21.7 | 685 | 10.7 |
| Madagascar | 2018 | 1.2 | 0.3 | 4.7 | 362 | 1.0 | 0.4 | 2.4 | 639 | 6.0 | 3.8 | 9.2 | 382 | 2.7 | 0.1 | 0.0 | 0.6 | 979 | 0.4 | 0.2 | 0.9 | 1907 | 5.6 | 3.5 | 8.7 | 973 | 1.8 |
| Malawi | 2000 | 0.7 | 0.2 | 3.0 | 329 | 1.4 | 0.7 | 2.5 | 800 | 7.0 | 3.6 | 13.4 | 113 | 1.7 | 0.9 | 0.4 | 2.1 | 948 | 3.1 | 2.3 | 4.2 | 2085 | 14.4 | 10.6 | 19.3 | 279 | 3.3 |
| Malawi | 2004 | 1.6 | 0.4 | 5.5 | 253 | 2.5 | 1.6 | 4.1 | 726 | 8.7 | 3.9 | 18.1 | 120 | 3.0 | 1.3 | 0.7 | 2.4 | 839 | 4.0 | 2.5 | 6.4 | 2117 | 15.4 | 10.8 | 21.5 | 371 | 4.7 |
| Malawi | 2006 | 0.3 | 0.1 | 1.4 | 447 | 0.4 | 0.2 | 1.0 | 1521 | 2.0 | 0.7 | 6.1 | 326 | 0.6 | 0.3 | 0.1 | 0.9 | 1637 | 1.0 | 0.7 | 1.6 | 5171 | 3.2 | 2.0 | 5.0 | 843 | 1.1 |
| Malawi | 2010 | 1.0 | 0.1 | 6.6 | 242 | 0.9 | 0.4 | 2.0 | 1166 | 3.8 | 1.8 | 8.2 | 255 | 1.4 | 0.4 | 0.1 | 1.0 | 905 | 2.4 | 1.8 | 3.1 | 3950 | 7.3 | 5.1 | 10.3 | 833 | 2.8 |
| Malawi | 2013 | 0.9 | 0.2 | 3.9 | 194 | 1.5 | 0.6 | 3.7 | 1176 | 4.7 | 2.0 | 10.9 | 314 | 2.0 | 1.3 | 0.6 | 2.8 | 635 | 1.0 | 0.7 | 1.5 | 4016 | 6.7 | 4.7 | 9.5 | 1008 | 2.1 |
| Malawi | 2015 | 2.9 | 0.9 | 9.0 | 203 | 1.6 | 0.8 | 3.1 | 1065 | 2.3 | 0.9 | 6.3 | 368 | 1.9 | 1.4 | 0.7 | 2.9 | 515 | 1.9 | 1.4 | 2.6 | 3199 | 5.3 | 3.1 | 8.9 | 1033 | 2.6 |
| Maldives | 2009 | 36.5 | 19.2 | 58.1 | 30 | 21.2 | 14.5 | 30.0 | 151 | 22.1 | 15.7 | 30.0 | 231 | 23.0 | 42.6 | 33.8 | 52.0 | 141 | 40.2 | 34.7 | 46.0 | 408 | 51.6 | 46.6 | 56.6 | 689 | 47.4 |
| Maldives | 2016 | 38.0 | 5.2 | 87.3 | 3 | 16.1 | 5.3 | 39.8 | 47 | 17.0 | 9.5 | 28.5 | 238 | 17.0 | 20.8 | 5.1 | 56.0 | 11 | 43.7 | 33.4 | 54.5 | 128 | 45.4 | 39.5 | 51.4 | 709 | 44.9 |
| Mali | 1995 | 2.1 | 1.3 | 3.5 | 857 | 3.9 | 1.5 | 10.0 | 131 | 27.8 | 17.1 | 41.8 | 48 | 3.4 | 4.5 | 3.4 | 5.8 | 2078 | 8.4 | 5.2 | 13.2 | 277 | 40.1 | 30.1 | 51.1 | 120 | 6.6 |
| Mali | 2001 | 2.8 | 1.9 | 4.1 | 1215 | 7.1 | 3.7 | 13.5 | 150 | 17.5 | 10.4 | 27.9 | 53 | 3.8 | 11.4 | 9.7 | 13.3 | 2817 | 10.7 | 7.5 | 15.1 | 389 | 36.6 | 29.4 | 44.4 | 166 | 12.6 |
| Mali | 2006 | 2.1 | 1.4 | 3.2 | 1184 | 6.9 | 3.5 | 13.2 | 156 | 13.9 | 8.1 | 22.7 | 81 | 3.2 | 9.6 | 8.1 | 11.3 | 3160 | 13.5 | 9.6 | 18.6 | 379 | 33.9 | 27.1 | 41.3 | 241 | 11.2 |
| Mali | 2009 | 2.4 | 1.7 | 3.4 | 2299 | 3.6 | 1.9 | 6.7 | 357 | 7.8 | 4.6 | 12.9 | 257 | 3.0 | 5.5 | 4.6 | 6.5 | 6185 | 11.7 | 9.4 | 14.4 | 1023 | 21.7 | 17.9 | 26.1 | 608 | 7.6 |
| Mali | 2012 | 2.8 | 1.9 | 4.3 | 798 | 4.7 | 1.9 | 11.0 | 81 | 18.5 | 12.3 | 26.7 | 120 | 4.7 | 3.4 | 2.4 | 4.8 | 2258 | 10.3 | 6.3 | 16.3 | 265 | 25.4 | 18.7 | 33.6 | 272 | 6.0 |
| Mali | 2015 | 1.3 | 0.8 | 2.2 | 1268 | 1.6 | 0.6 | 4.2 | 184 | 2.3 | 0.6 | 8.7 | 116 | 1.5 | 3.2 | 2.5 | 4.0 | 3683 | 6.3 | 4.4 | 9.1 | 563 | 7.3 | 4.6 | 11.4 | 360 | 5.2 |
| Mali | 2018 | 2.3 | 1.3 | 3.8 | 695 | 4.2 | 1.8 | 9.6 | 129 | 17.4 | 11.8 | 24.9 | 173 | 5.2 | 4.1 | 3.2 | 5.3 | 1917 | 4.4 | 2.6 | 7.4 | 341 | 17.0 | 13.2 | 21.6 | 455 | 6.3 |
| Mauritania | 2007 | 11.0 | 7.5 | 15.8 | 248 | 18.0 | 13.4 | 23.9 | 313 | 28.5 | 21.4 | 36.9 | 127 | 15.9 | 14.2 | 11.5 | 17.6 | 830 | 18.1 | 14.8 | 22.0 | 843 | 29.2 | 23.0 | 36.4 | 351 | 16.4 |
| Mauritania | 2011 | 6.2 | 3.3 | 11.2 | 258 | 8.1 | 5.2 | 12.6 | 353 | 34.3 | 26.8 | 42.8 | 154 | 11.3 | 9.6 | 7.1 | 12.8 | 673 | 17.2 | 14.7 | 19.9 | 966 | 34.5 | 29.2 | 40.3 | 464 | 16.7 |
| Mauritania | 2015 | 7.3 | 4.0 | 12.9 | 242 | 6.7 | 4.7 | 9.5 | 519 | 29.6 | 20.9 | 40.0 | 153 | 10.7 | 4.9 | 3.3 | 7.2 | 832 | 8.4 | 6.7 | 10.6 | 1806 | 26.9 | 21.4 | 33.3 | 538 | 10.6 |
| Moldova | 2005 | . | . | . | . | 37.5 | 3.1 | 91.9 | 2 | 23.2 | 16.4 | 31.9 | 156 | 23.4 | 0.0 | - | - | 3 | 0.0 | - | - | 5 | 30.9 | 26.7 | 35.4 | 478 | 30.5 |
| Moldova | 2012 | . | . | . | . | 0.0 | - | - | 1 | 21.0 | 14.8 | 28.9 | 174 | 21.2 | . | . | . | . | 17.4 | 3.5 | 54.9 | 7 | 25.0 | 21.5 | 28.8 | 580 | 24.8 |
| Mongolia | 2005 | 4.6 | 0.7 | 23.5 | 22 | 6.0 | 1.5 | 21.6 | 33 | 17.1 | 13.0 | 22.3 | 344 | 15.5 | 8.2 | 1.8 | 30.6 | 48 | 10.3 | 5.2 | 19.5 | 87 | 34.6 | 30.7 | 38.8 | 963 | 31.6 |
| Mongolia | 2010 | 0.0 | - | - | 22 | 3.0 | 0.4 | 18.5 | 29 | 8.8 | 5.7 | 13.2 | 359 | 8.1 | 1.8 | 0.3 | 11.9 | 63 | 4.1 | 1.3 | 11.9 | 118 | 6.9 | 5.2 | 9.1 | 1142 | 6.5 |
| Mongolia | 2013 | 7.5 | 1.9 | 25.9 | 34 | 7.5 | 2.3 | 21.4 | 37 | 18.6 | 15.2 | 22.6 | 573 | 17.4 | 3.0 | 0.9 | 9.5 | 106 | 1.5 | 0.4 | 6.0 | 130 | 7.4 | 6.1 | 9.0 | 1565 | 6.8 |
| Mongolia | 2018 | 23.1 | 7.1 | 54.2 | 36 | 15.4 | 4.7 | 40.5 | 35 | 21.4 | 16.4 | 27.4 | 545 | 21.2 | 4.3 | 1.3 | 13.7 | 88 | 7.2 | 2.8 | 17.2 | 106 | 8.4 | 6.2 | 11.2 | 1480 | 8.2 |
| Montenegro | 2005 | . | . | . | . | 27.9 | 12.6 | 50.9 | 18 | 24.3 | 13.7 | 39.4 | 53 | 25.3 | . | . | . | . | 10.5 | 4.2 | 23.7 | 64 | 8.7 | 5.4 | 13.7 | 220 | 9.1 |
| Montenegro | 2013 | . | . | . | . | 43.9 | 19.5 | 71.5 | 18 | 34.2 | 20.7 | 50.8 | 90 | 36.0 | 0.0 | - | - | 3 | 1.2 | 0.2 | 8.1 | 58 | 8.0 | 5.5 | 11.7 | 327 | 6.9 |
| Montenegro | 2018 | 0.0 | - | - | 3 | 41.4 | 13.6 | 76.1 | 14 | 39.1 | 26.1 | 53.8 | 84 | 38.1 | 19.2 | 14.9 | 24.4 | 14 | 29.5 | 14.1 | 51.6 | 31 | 20.9 | 13.6 | 30.7 | 287 | 21.3 |
| Mozambique | 2003 | 1.2 | 0.5 | 2.6 | 400 | 6.0 | 4.1 | 8.5 | 575 | 41.6 | 23.8 | 61.9 | 52 | 5.2 | 2.3 | 1.5 | 3.4 | 1154 | 6.2 | 4.9 | 7.8 | 1493 | 29.2 | 19.8 | 40.7 | 158 | 5.4 |
| Mozambique | 2008 | 2.7 | 1.0 | 6.9 | 355 | 4.4 | 3.0 | 6.5 | 703 | 22.5 | 16.1 | 30.4 | 171 | 5.9 | 1.1 | 0.6 | 2.2 | 1071 | 3.7 | 2.8 | 4.9 | 2204 | 20.4 | 15.5 | 26.3 | 377 | 4.1 |
| Mozambique | 2011 | 1.9 | 0.7 | 4.8 | 319 | 2.9 | 1.5 | 5.4 | 553 | 12.9 | 7.9 | 20.3 | 172 | 3.8 | 0.4 | 0.1 | 1.6 | 1120 | 0.9 | 0.5 | 1.5 | 1687 | 7.8 | 5.6 | 10.6 | 473 | 1.5 |
| Namibia | 2006 | 2.4 | 0.3 | 15.4 | 57 | 8.0 | 3.7 | 16.3 | 149 | 26.1 | 20.8 | 32.3 | 289 | 18.5 | 1.7 | 0.7 | 4.2 | 180 | 4.7 | 3.0 | 7.3 | 410 | 26.9 | 22.7 | 31.5 | 806 | 17.9 |
| Namibia | 2013 | 0.0 | - | - | 43 | 6.3 | 2.7 | 14.0 | 112 | 19.5 | 15.2 | 24.6 | 370 | 15.6 | 6.4 | 2.4 | 16.3 | 100 | 5.0 | 2.6 | 9.4 | 327 | 14.4 | 12.0 | 17.3 | 876 | 11.7 |
| Nepal | 1996 | 0.7 | 0.2 | 2.2 | 516 | 0.0 | - | - | 91 | 4.9 | 1.8 | 13.0 | 70 | 1.0 | 0.5 | 0.2 | 1.0 | 1623 | 0.9 | 0.2 | 3.7 | 211 | 6.3 | 3.5 | 10.9 | 204 | 1.1 |
| Nepal | 2006 | 0.0 | - | - | 255 | 0.0 | - | - | 82 | 6.9 | 2.9 | 15.6 | 136 | 2.0 | 0.5 | 0.2 | 1.4 | 877 | 2.4 | 1.0 | 5.5 | 297 | 5.2 | 2.7 | 9.9 | 368 | 2.0 |
| Nepal | 2011 | 0.3 | 0.0 | 2.1 | 208 | 0.0 | - | - | 103 | 2.8 | 1.3 | 5.9 | 186 | 1.1 | 0.8 | 0.3 | 2.8 | 574 | 0.2 | 0.0 | 1.5 | 274 | 4.8 | 3.1 | 7.4 | 573 | 2.3 |
| Nepal | 2014 | 0.6 | 0.1 | 4.2 | 171 | 1.8 | 0.3 | 9.5 | 75 | 4.6 | 2.2 | 9.2 | 206 | 2.7 | 0.8 | 0.2 | 2.3 | 627 | 3.9 | 1.8 | 8.5 | 254 | 4.9 | 3.3 | 7.1 | 672 | 3.1 |
| Nepal | 2016 | 0.0 | - | - | 111 | 1.1 | 0.2 | 7.9 | 89 | 7.5 | 4.2 | 13.1 | 267 | 4.5 | 1.0 | 0.3 | 3.1 | 418 | 2.5 | 1.1 | 5.6 | 283 | 3.5 | 2.4 | 5.1 | 762 | 2.5 |
| Niger | 1998 | 1.0 | 0.5 | 2.0 | 692 | 2.5 | 0.8 | 7.4 | 88 | 20.1 | 10.5 | 35.1 | 36 | 1.7 | 0.8 | 0.5 | 1.3 | 1745 | 4.1 | 2.2 | 7.7 | 223 | 15.7 | 9.2 | 25.5 | 95 | 1.6 |
| Niger | 2006 | 8.7 | 6.8 | 11.1 | 798 | 14.5 | 7.6 | 25.7 | 110 | 19.4 | 8.2 | 39.3 | 37 | 9.5 | 25.2 | 23.3 | 27.2 | 2035 | 31.0 | 25.1 | 37.6 | 282 | 50.7 | 39.2 | 62.1 | 162 | 26.8 |
| Niger | 2012 | 0.8 | 0.4 | 1.7 | 1091 | 1.7 | 0.4 | 6.7 | 138 | 8.8 | 4.0 | 18.4 | 72 | 1.2 | 1.0 | 0.6 | 1.5 | 2629 | 1.0 | 0.4 | 2.5 | 369 | 12.7 | 8.9 | 17.9 | 257 | 1.6 |
| Nigeria | 1999 | 3.0 | 1.7 | 5.3 | 271 | 7.6 | 3.8 | 14.4 | 134 | 16.4 | 11.3 | 23.4 | 150 | 7.7 | 1.3 | 0.6 | 2.7 | 777 | 5.8 | 4.1 | 8.3 | 401 | 11.5 | 8.4 | 15.4 | 442 | 5.2 |
| Nigeria | 2003 | 5.8 | 3.5 | 9.3 | 314 | 13.8 | 8.3 | 22.1 | 139 | 25.1 | 14.9 | 39.1 | 158 | 12.7 | 4.4 | 2.9 | 6.6 | 750 | 11.9 | 8.4 | 16.5 | 401 | 19.8 | 15.5 | 24.8 | 435 | 10.2 |
| Nigeria | 2007 | 8.0 | 5.5 | 11.5 | 840 | 14.8 | 11.0 | 19.5 | 388 | 24.9 | 19.9 | 30.7 | 445 | 14.5 | 5.4 | 4.1 | 7.0 | 2282 | 12.2 | 9.3 | 15.7 | 1087 | 23.3 | 20.3 | 26.7 | 1297 | 12.4 |
| Nigeria | 2008 | 1.8 | 1.1 | 2.7 | 1395 | 7.5 | 5.5 | 10.3 | 634 | 18.3 | 15.5 | 21.4 | 860 | 8.6 | 1.1 | 0.8 | 1.5 | 3746 | 5.1 | 3.9 | 6.6 | 1679 | 15.0 | 13.2 | 16.9 | 2132 | 6.4 |
| Nigeria | 2011 | 4.4 | 3.2 | 5.9 | 1291 | 15.1 | 10.8 | 20.7 | 524 | 26.9 | 23.2 | 31.1 | 899 | 14.7 | 6.0 | 4.8 | 7.5 | 3400 | 10.6 | 8.6 | 13.0 | 1531 | 24.4 | 21.6 | 27.5 | 2595 | 14.4 |
| Nigeria | 2013 | 0.8 | 0.4 | 1.5 | 1359 | 5.5 | 3.5 | 8.6 | 549 | 10.9 | 8.7 | 13.6 | 1026 | 5.0 | 1.1 | 0.7 | 1.5 | 3921 | 3.1 | 2.3 | 4.1 | 1686 | 12.4 | 10.9 | 14.0 | 3114 | 5.4 |
| Nigeria | 2016 | 2.9 | 1.2 | 6.8 | 700 | 5.4 | 3.5 | 8.2 | 439 | 11.0 | 8.8 | 13.8 | 867 | 6.4 | 1.9 | 1.3 | 2.6 | 2184 | 4.7 | 3.4 | 6.4 | 1274 | 10.0 | 8.6 | 11.6 | 2476 | 6.6 |
| Nigeria | 2018 | 0.8 | 0.4 | 1.4 | 1404 | 3.7 | 2.3 | 6.0 | 494 | 12.6 | 10.4 | 15.1 | 1295 | 5.9 | 2.4 | 1.8 | 3.2 | 3892 | 3.5 | 2.4 | 5.3 | 1293 | 11.9 | 10.6 | 13.3 | 3698 | 6.5 |
| North Macedonia | 2005 | 17.2 | 3.3 | 56.2 | 39 | 15.9 | 5.2 | 39.3 | 130 | 43.7 | 17.3 | 74.3 | 64 | 22.8 | 24.9 | 7.8 | 56.7 | 156 | 21.3 | 13.1 | 32.7 | 696 | 7.0 | 4.2 | 11.4 | 347 | 16.1 |
| North Macedonia | 2011 | 0.0 | - | - | 3 | 41.5 | 21.7 | 64.5 | 41 | 41.3 | 27.9 | 56.2 | 68 | 40.3 | 0.0 | - | - | 6 | 31.2 | 23.2 | 40.6 | 127 | 19.5 | 14.0 | 26.4 | 277 | 23.6 |
| Pakistan | 2012 | 5.8 | 3.8 | 8.9 | 566 | 5.9 | 2.9 | 11.7 | 168 | 19.8 | 14.3 | 26.9 | 342 | 9.9 | 3.5 | 2.6 | 4.8 | 1529 | 4.6 | 2.6 | 8.1 | 425 | 11.3 | 8.7 | 14.6 | 916 | 5.9 |
| Pakistan | 2017 | 7.8 | 5.2 | 11.6 | 585 | 11.4 | 6.7 | 18.9 | 163 | 18.0 | 13.9 | 23.0 | 369 | 12.2 | 5.0 | 3.7 | 6.8 | 1344 | 5.5 | 3.2 | 9.2 | 304 | 9.2 | 7.0 | 12.0 | 918 | 6.7 |
| Peru | 1996 | 0.2 | 0.0 | 1.5 | 162 | 2.5 | 1.4 | 4.4 | 643 | 16.2 | 12.7 | 20.5 | 714 | 9.4 | 1.0 | 0.4 | 2.5 | 477 | 1.6 | 1.0 | 2.5 | 2064 | 4.4 | 3.3 | 5.8 | 2188 | 3.0 |
| Peru | 2000 | 4.7 | 1.2 | 17.1 | 88 | 5.5 | 3.1 | 9.5 | 497 | 11.6 | 8.5 | 15.6 | 577 | 8.8 | 1.2 | 0.3 | 4.6 | 314 | 1.0 | 0.5 | 1.9 | 1598 | 3.2 | 2.3 | 4.6 | 1787 | 2.2 |
| Peru | 2004 | 0.0 | - | - | 15 | 2.4 | 0.7 | 8.5 | 85 | 20.4 | 12.0 | 32.7 | 133 | 13.9 | 0.0 | - | - | 36 | 1.2 | 0.4 | 3.6 | 277 | 5.5 | 3.1 | 9.3 | 409 | 3.8 |
| Peru | 2005 | 7.3 | 1.0 | 38.7 | 13 | 4.3 | 1.8 | 10.0 | 106 | 21.2 | 13.3 | 31.9 | 154 | 14.3 | 0.0 | - | - | 34 | 0.0 | - | - | 318 | 7.5 | 4.3 | 12.6 | 449 | 4.6 |
| Peru | 2006 | 0.0 | - | - | 13 | 2.9 | 0.8 | 10.4 | 90 | 18.0 | 10.8 | 28.4 | 148 | 12.3 | 0.0 | - | - | 48 | 0.0 | - | - | 324 | 3.1 | 1.5 | 6.3 | 470 | 1.9 |
| Peru | 2007 | 0.0 | - | - | 10 | 11.4 | 1.7 | 48.7 | 84 | 15.9 | 7.2 | 31.5 | 129 | 13.7 | 0.0 | - | - | 30 | 0.6 | 0.2 | 1.9 | 292 | 8.4 | 4.8 | 14.3 | 430 | 5.3 |
| Peru | 2008 | 8.1 | 1.1 | 41.0 | 20 | 8.3 | 3.4 | 19.0 | 152 | 20.9 | 14.1 | 29.9 | 373 | 16.9 | 0.0 | - | - | 63 | 3.0 | 1.7 | 5.1 | 630 | 7.9 | 5.6 | 11.1 | 1141 | 6.1 |
| Peru | 2009 | 0.0 | - | - | 34 | 3.3 | 1.4 | 7.5 | 282 | 18.2 | 14.1 | 23.1 | 585 | 13.7 | 2.2 | 0.3 | 14.2 | 95 | 2.1 | 1.2 | 3.7 | 991 | 11.9 | 9.7 | 14.4 | 1941 | 8.9 |
| Peru | 2010 | 0.0 | - | - | 18 | 8.1 | 4.5 | 14.3 | 255 | 18.9 | 14.4 | 24.4 | 493 | 15.4 | 0.0 | - | - | 64 | 2.2 | 1.1 | 4.1 | 940 | 11.4 | 9.3 | 14.0 | 1702 | 8.4 |
| Peru | 2011 | 0.0 | - | - | 18 | 7.3 | 3.8 | 13.4 | 234 | 17.8 | 13.5 | 23.1 | 528 | 14.9 | 5.8 | 2.0 | 15.7 | 92 | 0.9 | 0.5 | 1.7 | 872 | 12.5 | 10.3 | 15.1 | 1593 | 9.0 |
| Peru | 2012 | 5.1 | 0.7 | 28.6 | 28 | 6.3 | 3.3 | 11.7 | 254 | 25.9 | 20.9 | 31.5 | 545 | 20.0 | 1.2 | 0.2 | 8.4 | 89 | 2.0 | 1.2 | 3.4 | 834 | 15.7 | 13.1 | 18.6 | 1806 | 11.7 |
| Peru | 2013 | 1.1 | 0.1 | 7.5 | 20 | 7.4 | 3.5 | 14.9 | 199 | 21.2 | 16.6 | 26.6 | 531 | 17.8 | 0.2 | 0.0 | 1.6 | 75 | 5.6 | 3.6 | 8.5 | 734 | 18.2 | 15.5 | 21.1 | 1771 | 14.8 |
| Peru | 2014 | 0.0 | - | - | 19 | 8.0 | 4.5 | 13.9 | 213 | 26.5 | 22.0 | 31.6 | 646 | 22.3 | 3.6 | 0.5 | 22.0 | 51 | 4.7 | 2.8 | 7.9 | 705 | 19.2 | 17.0 | 21.6 | 2011 | 15.9 |
| Peru | 2015 | 0.0 | - | - | 29 | 9.0 | 5.9 | 13.5 | 400 | 27.8 | 24.5 | 31.3 | 1418 | 23.6 | 0.0 | - | - | 122 | 6.1 | 4.7 | 7.8 | 1580 | 21.6 | 19.9 | 23.3 | 5337 | 17.9 |
| Peru | 2016 | 9.2 | 1.3 | 44.7 | 16 | 6.5 | 4.1 | 10.1 | 325 | 22.6 | 19.5 | 26.2 | 1105 | 19.0 | 5.9 | 2.3 | 14.0 | 108 | 7.3 | 5.6 | 9.5 | 1281 | 22.3 | 20.4 | 24.2 | 4809 | 19.1 |
| Peru | 2017 | 0.0 | - | - | 32 | 9.3 | 6.4 | 13.2 | 347 | 24.8 | 21.7 | 28.2 | 1413 | 21.7 | 0.0 | - | - | 89 | 7.8 | 6.0 | 10.2 | 1190 | 23.6 | 21.9 | 25.3 | 5301 | 20.5 |
| Peru | 2018 | 7.1 | 1.0 | 36.7 | 19 | 7.2 | 4.3 | 11.8 | 340 | 23.9 | 21.1 | 27.0 | 1378 | 20.7 | 2.7 | 0.9 | 7.8 | 93 | 7.0 | 5.4 | 9.1 | 1179 | 22.2 | 20.7 | 23.8 | 5262 | 19.4 |
| Philippines | 1993 | 7.9 | 1.9 | 27.1 | 27 | 13.9 | 10.3 | 18.4 | 336 | 25.6 | 21.7 | 29.9 | 458 | 20.5 | 8.1 | 3.6 | 17.4 | 76 | 11.6 | 9.6 | 14.0 | 1011 | 12.9 | 11.1 | 14.9 | 1394 | 12.3 |
| Philippines | 1998 | 15.7 | 6.1 | 34.7 | 26 | 24.0 | 18.5 | 30.5 | 265 | 48.8 | 43.7 | 53.9 | 466 | 40.2 | 10.1 | 4.4 | 21.6 | 53 | 19.6 | 16.7 | 22.8 | 727 | 42.5 | 39.4 | 45.7 | 1357 | 34.9 |
| Philippines | 2003 | 0.0 | - | - | 16 | 14.1 | 9.7 | 20.2 | 182 | 36.9 | 32.2 | 42.0 | 426 | 29.8 | 19.6 | 10.4 | 33.9 | 41 | 34.3 | 29.9 | 39.0 | 559 | 56.4 | 53.5 | 59.3 | 1340 | 49.6 |
| Philippines | 2008 | 0.0 | - | - | 8 | 17.0 | 11.3 | 24.8 | 169 | 44.2 | 39.2 | 49.4 | 416 | 36.3 | 2.7 | 0.4 | 17.0 | 37 | 14.8 | 11.2 | 19.2 | 422 | 44.2 | 41.1 | 47.4 | 1359 | 37.1 |
| Rwanda | 2000 | 0.4 | 0.1 | 2.9 | 227 | 0.3 | 0.1 | 0.8 | 445 | 3.5 | 1.0 | 11.3 | 85 | 0.6 | 0.3 | 0.1 | 1.1 | 645 | 0.6 | 0.3 | 1.1 | 1185 | 9.8 | 6.7 | 14.1 | 285 | 1.5 |
| Rwanda | 2005 | 4.1 | 2.0 | 8.4 | 223 | 3.5 | 2.1 | 5.7 | 585 | 9.6 | 4.0 | 21.1 | 68 | 4.1 | 54.1 | 50.0 | 58.1 | 622 | 58.9 | 56.0 | 61.8 | 1504 | 78.3 | 71.5 | 83.8 | 214 | 59.2 |
| Rwanda | 2010 | 0.0 | - | - | 118 | 0.1 | 0.0 | 0.5 | 509 | 3.6 | 1.3 | 9.7 | 82 | 0.4 | 1.5 | 0.7 | 3.2 | 399 | 0.5 | 0.2 | 0.9 | 1710 | 3.0 | 1.2 | 7.1 | 224 | 0.9 |
| Rwanda | 2014 | 0.0 | - | - | 74 | 0.3 | 0.0 | 1.9 | 490 | 2.3 | 0.9 | 5.6 | 139 | 0.6 | 0.6 | 0.2 | 2.4 | 323 | 0.9 | 0.6 | 1.5 | 1673 | 3.5 | 2.0 | 6.0 | 358 | 1.2 |
| Sao Tome and Principe | 2008 | 5.5 | 0.8 | 30.1 | 17 | 7.6 | 3.3 | 16.3 | 122 | 6.6 | 2.2 | 18.0 | 53 | 7.1 | 33.1 | 16.5 | 55.4 | 30 | 37.0 | 31.1 | 43.3 | 423 | 41.0 | 30.3 | 52.6 | 110 | 37.8 |
| Sao Tome and Principe | 2014 | 15.8 | 2.0 | 62.8 | 6 | 3.8 | 1.1 | 12.3 | 93 | 4.6 | 1.3 | 15.3 | 70 | 4.5 | 0.6 | 0.1 | 4.6 | 31 | 5.7 | 3.5 | 9.2 | 358 | 10.7 | 5.8 | 18.9 | 182 | 7.3 |
| Senegal | 2005 | 6.6 | 4.8 | 9.1 | 950 | 10.4 | 4.6 | 22.0 | 260 | 21.5 | 12.2 | 35.2 | 80 | 8.7 | 14.8 | 12.6 | 17.2 | 2256 | 23.8 | 19.6 | 28.7 | 635 | 42.3 | 31.2 | 54.2 | 169 | 18.6 |
| Senegal | 2010 | 2.3 | 1.2 | 4.5 | 972 | 7.5 | 3.8 | 14.1 | 249 | 8.6 | 3.3 | 20.3 | 113 | 3.9 | 2.2 | 1.5 | 3.2 | 2467 | 5.0 | 2.9 | 8.4 | 653 | 16.4 | 11.0 | 23.8 | 255 | 4.0 |
| Senegal | 2012 | 1.2 | 0.6 | 2.7 | 433 | 0.6 | 0.1 | 3.9 | 152 | 8.7 | 3.2 | 21.5 | 87 | 2.2 | 1.6 | 0.9 | 2.9 | 1381 | 2.2 | 0.7 | 6.9 | 386 | 12.5 | 7.1 | 20.9 | 168 | 2.8 |
| Senegal | 2014 | 1.5 | 0.7 | 3.3 | 424 | 4.8 | 0.9 | 21.8 | 107 | 4.6 | 0.8 | 22.5 | 81 | 2.7 | 0.5 | 0.2 | 1.2 | 1322 | 0.5 | 0.2 | 1.4 | 373 | 6.1 | 2.4 | 14.9 | 190 | 1.2 |
| Senegal | 2015 | 2.5 | 0.9 | 6.6 | 408 | 1.1 | 0.3 | 4.6 | 130 | 9.3 | 2.9 | 25.6 | 88 | 3.3 | 1.0 | 0.6 | 1.9 | 1385 | 3.7 | 1.7 | 8.1 | 379 | 6.3 | 2.8 | 13.6 | 220 | 2.2 |
| Senegal | 2016 | 1.5 | 0.7 | 3.0 | 398 | 0.0 | - | - | 118 | 5.3 | 1.2 | 20.0 | 89 | 2.0 | 2.9 | 1.8 | 4.8 | 1342 | 2.0 | 0.6 | 7.0 | 368 | 4.8 | 1.5 | 14.1 | 230 | 3.0 |
| Senegal | 2017 | 5.0 | 2.9 | 8.2 | 712 | 1.8 | 0.6 | 4.8 | 227 | 9.4 | 5.0 | 16.9 | 203 | 5.3 | 1.4 | 0.8 | 2.4 | 2192 | 3.9 | 2.2 | 6.8 | 695 | 13.7 | 10.0 | 18.4 | 601 | 4.2 |
| Serbia | 2005 | . | . | . | . | 26.2 | 15.8 | 40.1 | 169 | 19.6 | 14.1 | 26.6 | 165 | 21.3 | . | . | . | . | 3.6 | 1.8 | 7.1 | 514 | 12.7 | 9.8 | 16.3 | 615 | 10.7 |
| Serbia | 2010 | 7.6 | 0.7 | 48.5 | 5 | 28.8 | 12.7 | 52.9 | 37 | 43.5 | 32.5 | 55.3 | 204 | 40.2 | 4.1 | 0.4 | 30.0 | 6 | 1.6 | 0.4 | 6.2 | 126 | 10.9 | 7.7 | 15.2 | 879 | 9.4 |
| Serbia | 2014 | 0.0 | - | - | 2 | 61.9 | 31.8 | 85.0 | 15 | 40.0 | 27.8 | 53.5 | 152 | 40.9 | 0.0 | - | - | 9 | 1.0 | 0.1 | 6.5 | 83 | 10.1 | 7.9 | 12.7 | 703 | 8.9 |
| Sierra Leone | 2005 | 9.3 | 6.9 | 12.5 | 416 | 13.9 | 6.7 | 26.6 | 50 | 17.9 | 9.0 | 32.5 | 42 | 10.4 | 8.8 | 7.2 | 10.7 | 1255 | 12.5 | 7.9 | 19.1 | 170 | 31.5 | 24.2 | 39.8 | 165 | 11.4 |
| Sierra Leone | 2008 | 4.8 | 3.2 | 7.1 | 440 | 15.1 | 8.3 | 25.8 | 82 | 30.2 | 21.4 | 40.8 | 95 | 9.4 | 7.7 | 6.0 | 9.8 | 1109 | 11.2 | 6.2 | 19.2 | 212 | 28.3 | 22.0 | 35.6 | 216 | 10.7 |
| Sierra Leone | 2010 | 7.1 | 5.1 | 10.0 | 574 | 11.4 | 6.5 | 19.3 | 117 | 22.1 | 15.2 | 31.1 | 140 | 10.1 | 8.4 | 6.9 | 10.3 | 1659 | 15.7 | 11.9 | 20.3 | 356 | 31.3 | 26.5 | 36.6 | 427 | 13.2 |
| Sierra Leone | 2013 | 3.6 | 2.2 | 5.7 | 707 | 6.2 | 3.0 | 12.6 | 180 | 13.9 | 8.9 | 20.9 | 228 | 5.9 | 2.7 | 2.0 | 3.6 | 2037 | 7.6 | 5.1 | 11.2 | 426 | 13.1 | 9.5 | 17.7 | 625 | 5.5 |
| Sierra Leone | 2017 | 3.0 | 1.6 | 5.3 | 671 | 8.3 | 3.9 | 16.8 | 166 | 18.7 | 14.3 | 24.2 | 333 | 8.9 | 5.8 | 4.5 | 7.5 | 2019 | 7.5 | 4.8 | 11.5 | 507 | 20.2 | 17.1 | 23.7 | 885 | 10.4 |
| South Africa | 1998 | 34.2 | 22.8 | 47.7 | 47 | 48.3 | 38.3 | 58.6 | 124 | 55.4 | 49.2 | 61.5 | 334 | 51.5 | 26.1 | 17.4 | 37.1 | 100 | 28.4 | 23.7 | 33.7 | 403 | 46.3 | 42.5 | 50.1 | 871 | 39.8 |
| South Africa | 2016 | 47.2 | 11.6 | 86.0 | 6 | 28.3 | 11.4 | 54.6 | 25 | 31.1 | 25.5 | 37.4 | 315 | 31.4 | 45.0 | 16.2 | 77.6 | 9 | 17.4 | 10.2 | 28.1 | 86 | 42.0 | 37.4 | 46.8 | 782 | 40.2 |
| Suriname | 2006 | 26.1 | 13.0 | 45.5 | 20 | 62.0 | 47.1 | 75.0 | 54 | 65.2 | 55.6 | 73.7 | 106 | 60.4 | 38.4 | 26.6 | 51.7 | 102 | 53.9 | 46.0 | 61.6 | 189 | 66.7 | 61.4 | 71.7 | 359 | 59.4 |
| Suriname | 2010 | 61.0 | 44.0 | 75.8 | 64 | 70.7 | 59.2 | 80.1 | 106 | 78.5 | 70.1 | 85.0 | 133 | 74.1 | 51.8 | 41.7 | 61.8 | 206 | 65.4 | 58.8 | 71.4 | 390 | 81.4 | 76.7 | 85.3 | 462 | 72.7 |
| Suriname | 2018 | 51.7 | 23.3 | 79.0 | 17 | 48.3 | 31.8 | 65.2 | 55 | 61.3 | 52.7 | 69.3 | 259 | 58.9 | 46.7 | 33.8 | 60.1 | 52 | 52.7 | 43.2 | 62.1 | 193 | 68.3 | 63.4 | 72.8 | 913 | 64.9 |
| Tajikistan | 2005 | 0.0 | - | - | 5 | 10.2 | 1.4 | 48.1 | 10 | 6.8 | 4.4 | 10.5 | 373 | 6.8 | 6.6 | 0.8 | 36.9 | 12 | 15.8 | 5.3 | 38.6 | 21 | 17.9 | 15.3 | 20.9 | 1241 | 17.7 |
| Tajikistan | 2012 | 0.0 | - | - | 13 | 15.1 | 6.2 | 32.5 | 27 | 11.7 | 8.7 | 15.5 | 384 | 11.5 | 19.9 | 10.1 | 35.5 | 34 | 9.6 | 4.7 | 18.8 | 77 | 18.1 | 15.6 | 21.0 | 1356 | 17.7 |
| Tajikistan | 2017 | 0.0 | - | - | 4 | 3.3 | 0.5 | 19.4 | 28 | 10.0 | 7.7 | 13.0 | 521 | 9.7 | 13.1 | 5.8 | 26.8 | 48 | 13.0 | 7.2 | 22.3 | 91 | 11.6 | 9.9 | 13.5 | 1583 | 11.7 |
| Tanzania | 1996 | 4.0 | 2.0 | 8.1 | 188 | 3.4 | 1.9 | 6.1 | 428 | 3.4 | 0.5 | 19.7 | 38 | 3.6 | 4.7 | 3.4 | 6.4 | 519 | 11.2 | 9.4 | 13.2 | 1298 | 35.2 | 26.6 | 44.9 | 103 | 10.3 |
| Tanzania | 2004 | 0.6 | 0.1 | 2.4 | 201 | 0.7 | 0.2 | 2.3 | 551 | 3.9 | 1.9 | 7.6 | 73 | 0.8 | 2.5 | 1.5 | 4.1 | 665 | 3.8 | 2.7 | 5.3 | 1532 | 18.8 | 12.0 | 28.3 | 211 | 4.1 |
| Tanzania | 2010 | 0.1 | 0.0 | 0.6 | 207 | 0.4 | 0.1 | 1.9 | 501 | 11.1 | 3.9 | 27.9 | 95 | 1.0 | 0.3 | 0.0 | 2.0 | 554 | 0.5 | 0.2 | 1.7 | 1384 | 3.8 | 1.5 | 9.4 | 297 | 0.7 |
| Tanzania | 2015 | 0.0 | - | - | 201 | 0.7 | 0.2 | 2.4 | 618 | 1.5 | 0.4 | 5.2 | 196 | 0.7 | 0.0 | 0.0 | 0.2 | 603 | 1.2 | 0.7 | 2.0 | 1772 | 1.9 | 0.9 | 4.0 | 645 | 1.1 |
| Thailand | 2005 | 7.5 | 1.8 | 26.8 | 30 | 32.0 | 25.6 | 39.2 | 317 | 26.7 | 21.3 | 32.9 | 524 | 28.2 | 14.1 | 7.8 | 23.9 | 115 | 26.3 | 22.7 | 30.3 | 1281 | 31.6 | 28.0 | 35.4 | 1512 | 28.3 |
| Thailand | 2012 | 15.1 | 4.1 | 42.3 | 17 | 56.6 | 44.4 | 68.1 | 149 | 41.6 | 34.8 | 48.7 | 425 | 45.0 | 29.8 | 16.6 | 47.4 | 76 | 59.5 | 52.2 | 66.4 | 822 | 67.0 | 62.9 | 70.8 | 1720 | 63.0 |
| Thailand | 2015 | 12.5 | 3.1 | 38.9 | 26 | 58.6 | 41.7 | 73.7 | 126 | 43.2 | 34.5 | 52.4 | 505 | 44.9 | 23.3 | 11.5 | 41.6 | 115 | 53.7 | 46.6 | 60.7 | 898 | 56.4 | 51.2 | 61.5 | 2201 | 53.8 |
| Timor-Leste | 2009 | 13.1 | 8.9 | 18.9 | 332 | 18.4 | 13.7 | 24.4 | 264 | 32.0 | 25.6 | 39.2 | 364 | 22.1 | 14.4 | 11.7 | 17.5 | 824 | 15.3 | 12.3 | 18.9 | 743 | 27.7 | 24.1 | 31.6 | 1003 | 20.0 |
| Timor-Leste | 2016 | 1.2 | 0.4 | 3.8 | 171 | 5.4 | 2.0 | 13.8 | 96 | 13.5 | 9.9 | 18.1 | 476 | 9.5 | 4.0 | 2.5 | 6.4 | 436 | 7.2 | 4.8 | 10.5 | 372 | 16.3 | 13.9 | 19.0 | 1142 | 11.9 |
| Togo | 1998 | 8.5 | 6.0 | 12.1 | 407 | 8.5 | 5.0 | 14.2 | 232 | 20.5 | 10.5 | 36.2 | 53 | 9.7 | 8.6 | 6.9 | 10.6 | 1172 | 11.4 | 8.5 | 15.1 | 559 | 20.5 | 13.6 | 29.6 | 147 | 10.4 |
| Togo | 2006 | 22.6 | 17.3 | 29.0 | 253 | 15.4 | 9.5 | 24.1 | 134 | 14.2 | 6.5 | 28.3 | 59 | 18.8 | 54.4 | 49.3 | 59.5 | 710 | 59.7 | 53.6 | 65.4 | 433 | 66.3 | 58.3 | 73.5 | 195 | 58.2 |
| Togo | 2010 | 3.4 | 1.5 | 7.4 | 277 | 8.4 | 3.8 | 17.2 | 201 | 1.3 | 0.3 | 6.2 | 85 | 4.9 | 6.7 | 4.9 | 9.1 | 727 | 11.8 | 8.7 | 15.9 | 453 | 20.4 | 15.2 | 26.9 | 212 | 11.1 |
| Togo | 2013 | 2.3 | 0.7 | 6.9 | 258 | 1.0 | 0.3 | 3.2 | 207 | 3.2 | 1.2 | 8.5 | 138 | 2.1 | 1.4 | 0.7 | 2.6 | 929 | 0.8 | 0.4 | 1.9 | 696 | 6.2 | 3.7 | 10.1 | 445 | 2.3 |
| Togo | 2017 | 0.6 | 0.1 | 4.0 | 180 | 2.8 | 1.2 | 6.3 | 163 | 4.6 | 2.0 | 10.5 | 160 | 2.7 | 0.8 | 0.3 | 2.0 | 503 | 1.1 | 0.4 | 3.0 | 516 | 4.6 | 3.0 | 7.0 | 442 | 2.1 |
| Tunisia | 2011 | 4.0 | 0.9 | 16.1 | 44 | 7.4 | 3.4 | 15.4 | 85 | 11.2 | 6.6 | 18.3 | 177 | 9.5 | 7.9 | 3.7 | 15.7 | 129 | 10.9 | 7.3 | 16.0 | 270 | 14.6 | 11.0 | 19.2 | 455 | 12.8 |
| Tunisia | 2018 | 62.7 | 39.5 | 81.2 | 19 | 29.2 | 19.0 | 42.0 | 60 | 56.7 | 49.0 | 64.0 | 220 | 51.5 | 19.0 | 11.5 | 29.8 | 72 | 8.1 | 4.8 | 13.4 | 198 | 14.0 | 11.3 | 17.2 | 676 | 13.1 |
| Turkey | 1993 | 10.6 | 5.6 | 19.2 | 94 | 18.4 | 13.3 | 25.0 | 196 | 30.9 | 21.6 | 41.9 | 72 | 18.6 | 7.5 | 5.1 | 10.8 | 288 | 4.0 | 2.6 | 6.1 | 575 | 6.9 | 3.9 | 12.0 | 171 | 5.5 |
| Turkey | 1998 | 13.1 | 6.9 | 23.2 | 74 | 24.6 | 18.3 | 32.2 | 214 | 44.0 | 32.3 | 56.3 | 82 | 26.8 | 7.6 | 4.8 | 12.0 | 214 | 12.4 | 9.5 | 15.9 | 585 | 21.5 | 14.9 | 30.0 | 213 | 13.4 |
| Turkey | 2003 | 7.0 | 3.7 | 13.1 | 102 | 21.7 | 15.6 | 29.3 | 212 | 33.7 | 24.3 | 44.4 | 99 | 22.0 | 8.3 | 5.5 | 12.4 | 284 | 15.1 | 12.1 | 18.6 | 572 | 24.3 | 18.7 | 30.9 | 300 | 16.3 |
| Turkey | 2013 | 32.6 | 19.2 | 49.7 | 59 | 31.8 | 21.3 | 44.5 | 104 | 31.6 | 24.3 | 39.9 | 169 | 31.8 | 13.4 | 8.7 | 20.2 | 154 | 23.5 | 18.6 | 29.1 | 366 | 32.4 | 27.9 | 37.2 | 533 | 27.1 |
| Turkmenistan | 2006 | 0.0 | - | - | 1 | 100.0 | - | - | 1 | 13.9 | 9.7 | 19.5 | 235 | 14.0 | 47.1 | 7.3 | 90.9 | 3 | 50.3 | 7.8 | 92.3 | 3 | 33.5 | 28.1 | 39.3 | 618 | 33.6 |
| Turkmenistan | 2015 | 0.0 | - | - | 1 | 0.0 | - | - | 1 | 12.4 | 8.9 | 17.1 | 340 | 12.3 | . | . | . | . | 0.0 | - | - | 2 | 9.5 | 7.8 | 11.5 | 1167 | 9.5 |
| Uganda | 1995 | 0.0 | - | - | 165 | 2.7 | 1.0 | 7.1 | 375 | 3.1 | 1.1 | 8.1 | 89 | 1.9 | 1.2 | 0.5 | 3.1 | 569 | 1.9 | 1.3 | 2.7 | 1217 | 6.2 | 4.1 | 9.2 | 293 | 2.1 |
| Uganda | 2000 | 1.0 | 0.2 | 4.6 | 134 | 0.9 | 0.3 | 2.5 | 421 | 5.3 | 2.0 | 13.1 | 101 | 1.5 | 0.8 | 0.3 | 2.2 | 484 | 1.4 | 0.8 | 2.3 | 1193 | 7.3 | 4.5 | 11.5 | 317 | 1.9 |
| Uganda | 2006 | 0.0 | - | - | 176 | 1.1 | 0.3 | 3.4 | 483 | 3.0 | 1.1 | 7.8 | 141 | 1.2 | 0.3 | 0.0 | 1.8 | 518 | 0.2 | 0.1 | 0.8 | 1410 | 1.1 | 0.4 | 2.9 | 308 | 0.4 |
| Uganda | 2011 | 0.0 | - | - | 122 | 0.2 | 0.0 | 0.8 | 453 | 1.2 | 0.3 | 5.5 | 203 | 0.4 | 0.1 | 0.0 | 0.4 | 341 | 0.0 | - | - | 1251 | 2.2 | 1.1 | 4.2 | 477 | 0.5 |
| Uganda | 2016 | 0.0 | - | - | 180 | 0.4 | 0.1 | 1.2 | 938 | 1.0 | 0.3 | 3.5 | 364 | 0.5 | 0.7 | 0.2 | 2.1 | 498 | 0.1 | 0.0 | 0.3 | 2574 | 0.7 | 0.3 | 1.6 | 1088 | 0.4 |
| Ukraine | 2005 | . | . | . | . | . | . | . | . | 34.0 | 27.0 | 41.8 | 229 | 34.0 | . | . | . | . | . | . | . | . | 34.3 | 28.7 | 40.4 | 907 | 34.3 |
| Ukraine | 2007 | . | . | . | . | 100.0 | - | - | 1 | 27.3 | 18.3 | 38.6 | 97 | 28.0 | 0.0 | - | - | 1 | 40.8 | 4.1 | 91.8 | 2 | 42.4 | 36.1 | 49.0 | 335 | 42.3 |
| Ukraine | 2012 | . | . | . | . | . | . | . | . | 30.5 | 23.0 | 39.2 | 307 | 30.5 | . | . | . | . | . | . | . | . | 38.6 | 33.5 | 44.0 | 1254 | 38.8 |
| Uzbekistan | 1996 | . | . | . | . | . | . | . | . | 14.0 | 8.9 | 21.3 | 164 | 14.0 | . | . | . | . | 100.0 | - | - | 1 | 30.8 | 25.5 | 36.7 | 649 | 30.9 |
| Uzbekistan | 2006 | . | . | . | . | . | . | . | . | 11.5 | 8.5 | 15.4 | 445 | 11.5 | . | . | . | . | . | . | . | . | 21.5 | 18.9 | 24.3 | 1663 | 21.5 |
| Vietnam | 1997 | 4.4 | 0.7 | 22.1 | 27 | 0.0 | - | - | 65 | 5.5 | 2.5 | 11.5 | 145 | 3.9 | 4.3 | 1.6 | 11.3 | 67 | 5.3 | 3.0 | 9.2 | 230 | 9.5 | 7.0 | 12.9 | 576 | 7.9 |
| Vietnam | 2002 | 0.0 | - | - | 10 | 0.0 | - | - | 60 | 3.2 | 1.1 | 8.9 | 121 | 2.2 | 0.0 | - | - | 44 | 4.6 | 2.1 | 10.0 | 169 | 8.1 | 5.5 | 11.8 | 429 | 6.6 |
| Vietnam | 2006 | 1.4 | 0.2 | 9.9 | 34 | 24.6 | 15.3 | 37.2 | 88 | 47.4 | 36.7 | 58.3 | 107 | 34.1 | 4.5 | 1.1 | 16.0 | 89 | 27.7 | 21.9 | 34.3 | 369 | 46.6 | 40.3 | 53.1 | 350 | 34.7 |
| Vietnam | 2010 | 0.0 | - | - | 21 | 27.0 | 14.8 | 44.3 | 54 | 47.8 | 40.4 | 55.3 | 244 | 42.1 | 4.3 | 0.9 | 17.7 | 74 | 17.1 | 11.5 | 24.7 | 175 | 30.8 | 27.1 | 34.8 | 861 | 27.4 |
| Vietnam | 2013 | 18.1 | 5.6 | 45.3 | 21 | 28.1 | 16.6 | 43.4 | 47 | 39.8 | 33.7 | 46.3 | 290 | 37.5 | 12.9 | 5.7 | 26.6 | 63 | 26.0 | 18.1 | 35.9 | 152 | 45.7 | 41.6 | 50.0 | 903 | 41.8 |
| Zambia | 1996 | 0.0 | - | - | 93 | 2.8 | 1.5 | 5.1 | 450 | 5.8 | 3.0 | 11.3 | 132 | 3.1 | 0.0 | - | - | 275 | 1.1 | 0.6 | 1.9 | 1259 | 4.8 | 2.9 | 8.1 | 389 | 1.8 |
| Zambia | 2001 | 0.0 | - | - | 113 | 1.4 | 0.6 | 3.4 | 413 | 8.5 | 4.3 | 16.1 | 124 | 2.7 | 1.3 | 0.4 | 4.2 | 294 | 3.4 | 2.6 | 4.6 | 1219 | 7.1 | 4.8 | 10.2 | 410 | 4.0 |
| Zambia | 2007 | 0.0 | - | - | 89 | 1.2 | 0.4 | 3.4 | 373 | 6.9 | 3.6 | 12.9 | 156 | 2.5 | 0.5 | 0.1 | 3.7 | 241 | 1.8 | 1.1 | 2.8 | 1149 | 6.1 | 3.8 | 9.4 | 439 | 2.6 |
| Zambia | 2013 | 0.5 | 0.1 | 3.6 | 134 | 0.2 | 0.0 | 1.6 | 619 | 4.4 | 2.5 | 7.5 | 433 | 1.8 | 0.2 | 0.0 | 1.3 | 395 | 0.8 | 0.3 | 1.7 | 2019 | 3.2 | 2.1 | 4.7 | 1305 | 1.5 |
| Zambia | 2018 | 0.0 | - | - | 95 | 0.2 | 0.0 | 1.2 | 515 | 5.5 | 3.4 | 8.7 | 409 | 2.3 | 0.0 | - | - | 276 | 0.5 | 0.2 | 1.1 | 1459 | 3.7 | 2.5 | 5.3 | 1050 | 1.7 |
| Zimbabwe | 1994 | 0.0 | - | - | 39 | 3.5 | 1.4 | 8.9 | 194 | 7.8 | 4.1 | 14.3 | 167 | 5.0 | 1.5 | 1.2 | 1.8 | 154 | 3.0 | 1.8 | 4.8 | 544 | 7.8 | 5.1 | 11.8 | 365 | 4.5 |
| Zimbabwe | 1999 | 0.0 | - | - | 22 | 2.6 | 0.7 | 8.6 | 151 | 4.0 | 1.6 | 9.4 | 174 | 3.2 | 0.0 | - | - | 66 | 1.3 | 0.6 | 2.9 | 465 | 2.1 | 1.1 | 3.7 | 469 | 1.6 |
| Zimbabwe | 2005 | 53.8 | 25.8 | 79.6 | 15 | 45.8 | 38.5 | 53.3 | 207 | 36.0 | 30.4 | 41.9 | 312 | 40.1 | 55.9 | 42.1 | 68.9 | 41 | 62.3 | 57.2 | 67.1 | 548 | 63.1 | 59.8 | 66.3 | 856 | 62.5 |
| Zimbabwe | 2009 | 0.0 | - | - | 20 | 0.0 | - | - | 212 | 4.9 | 3.0 | 7.7 | 449 | 3.2 | 1.0 | 0.1 | 6.6 | 71 | 1.0 | 0.5 | 2.3 | 786 | 3.2 | 2.3 | 4.5 | 1290 | 2.4 |
| Zimbabwe | 2010 | 0.0 | - | - | 9 | 1.4 | 0.5 | 3.9 | 208 | 2.6 | 1.4 | 4.7 | 424 | 2.2 | 0.0 | - | - | 23 | 0.5 | 0.1 | 2.3 | 520 | 3.0 | 2.1 | 4.3 | 1106 | 2.2 |
| Zimbabwe | 2014 | 0.0 | - | - | 5 | 0.9 | 0.2 | 4.6 | 277 | 4.6 | 3.1 | 6.8 | 585 | 3.4 | 1.6 | 0.2 | 10.9 | 61 | 1.6 | 0.9 | 2.8 | 935 | 3.4 | 2.7 | 4.4 | 1969 | 2.8 |
| Zimbabwe | 2015 | 0.0 | - | - | 5 | 0.4 | 0.1 | 3.1 | 158 | 3.7 | 2.2 | 6.3 | 440 | 2.8 | 0.0 | - | - | 17 | 0.2 | 0.0 | 0.7 | 496 | 2.4 | 1.6 | 3.5 | 1115 | 1.6 |
| Zimbabwe | 2019 | 0.0 | - | - | 3 | 1.2 | 0.3 | 4.9 | 165 | 4.8 | 3.0 | 7.6 | 437 | 3.8 | 0.0 | - | - | 24 | 0.5 | 0.1 | 1.9 | 478 | 3.5 | 2.5 | 4.8 | 1235 | 2.6 |

^1^CAR: Central African Republic; ^2^CDR: Congo Democratic Republic.

**Supplementary table 5.** Annual changes in the prevalence of breast milk and formula consumption indicators by mother’s formal education level according to the regions of the world. Source: Demographic Health Survey and Multiple Indicator Cluster Survey.

| Indicator | Regions of the world | Formal education level | | | | | | | | | | | | | |
| --- | --- | --- | --- | --- | --- | --- | --- | --- | --- | --- | --- | --- | --- | --- | --- |
|  |  | None | | | |  | Primary | | | |  | Secondary or higher | | | |
|  |  | Beta | 95% CI | | P |  | Beta | 95% CI | | P |  | Beta | 95% CI | | P |
| Early initiation of breastfeeding | West & Central Africa | 0.51 | -0.19 | 1.20 | 0.156 |  | **0.56** | **0.08** | **1.05** | **0.022** |  | **0.54** | **0.21** | **0.86** | **0.001** |
|  | Eastern & Southern Africa | **0.81** | **0.32** | **1.29** | **0.001** |  | **0.82** | **0.33** | **1.31** | **0.001** |  | **0.66** | **0.17** | **1.16** | **0.008** |
|  | Middle East & North Africa | -0.31 | -0.73 | 0.11 | 0.143 |  | **-0.41** | **-0.66** | **-0.16** | **0.001** |  | **-0.50** | **-0.83** | **-0.17** | **0.003** |
|  | Eastern Europe & Central Asia | **2.37** | **1.55** | **3.20** | **<0.001** |  | **1.47** | **0.33** | **2.61** | **0.011** |  | **2.00** | **1.35** | **2.65** | **<0.001** |
|  | South Asia | **2.05** | **1.77** | **2.32** | **<0.001** |  | **1.82** | **1.55** | **2.09** | **<0.001** |  | **1.24** | **1.00** | **1.48** | **<0.001** |
|  | East Asia & the Pacific | **1.04** | **0.37** | **1.71** | **0.002** |  | **1.03** | **0.52** | **1.55** | **<0.001** |  | **0.79** | **0.19** | **1.39** | **0.010** |
|  | Latin America & Caribbean | **1.37** | **0.81** | **1.92** | **<0.001** |  | **0.95** | **0.51** | **1.39** | **<0.001** |  | 0.12 | -0.25 | 0.49 | 0.537 |
| Exclusive breastfeeding  under 6 months | West & Central Africa | **0.92** | **0.49** | **1.35** | **<0.001** |  | **0.87** | **0.32** | **1.42** | **0.002** |  | **0.90** | **0.52** | **1.29** | **<0.001** |
|  | Eastern & Southern Africa | **0.93** | **0.15** | **1.72** | **0.019** |  | **1.44** | **0.83** | **2.06** | **<0.001** |  | **1.53** | **0.90** | **2.17** | **<0.001** |
|  | Middle East & North Africa | **-0.81** | **-1.51** | **-0.11** | **0.024** |  | **-0.94** | **-1.65** | **-0.23** | **0.009** |  | **-0.33** | **-0.55** | **-0.12** | **0.003** |
|  | Eastern Europe & Central Asia | **1.76** | **0.51** | **3.01** | **0.006** |  | **0.76** | **0.13** | **1.38** | **0.018** |  | **1.34** | **1.12** | **1.57** | **<0.001** |
|  | South Asia | **0.38** | **0.22** | **0.54** | **<0.001** |  | **0.59** | **0.17** | **1.01** | **0.006** |  | **0.95** | **0.69** | **1.21** | **<0.001** |
|  | East Asia & the Pacific | 0.03 | -1.28 | 1.34 | 0.962 |  | **0.70** | **0.26** | **1.14** | **0.002** |  | **0.93** | **0.60** | **1.27** | **<0.001** |
|  | Latin America & Caribbean | **1.36** | **0.62** | **2.11** | **<0.001** |  | **0.98** | **0.45** | **1.51** | **<0.001** |  | **1.03** | **0.45** | **1.61** | **0.001** |
| Continued breastfeeding  at 1 years | West & Central Africa | -0.14 | -0.29 | 0.01 | 0.063 |  | -0.12 | -0.32 | 0.08 | 0.241 |  | 0.01 | -0.22 | 0.23 | 0.960 |
|  | Eastern & Southern Africa | -0.32 | -0.66 | 0.02 | 0.064 |  | -0.17 | -0.35 | 0.00 | 0.053 |  | -0.16 | -0.41 | 0.08 | 0.182 |
|  | Middle East & North Africa | -0.43 | -0.96 | 0.11 | 0.117 |  | **-0.71** | **-1.01** | **-0.42** | **<0.001** |  | -0.10 | -1.31 | 1.11 | 0.877 |
|  | Eastern Europe & Central Asia | 0.38 | -0.26 | 1.02 | 0.246 |  | **1.16** | **0.81** | **1.51** | **<0.001** |  | **1.31** | **0.35** | **2.26** | **0.007** |
|  | South Asia | **-0.35** | **-0.46** | **-0.24** | **<0.001** |  | -0.16 | -0.38 | 0.06 | 0.146 |  | -0.01 | -0.27 | 0.25 | 0.912 |
|  | East Asia & the Pacific | -0.40 | -1.00 | 0.20 | 0.190 |  | -0.16 | -0.44 | 0.11 | 0.247 |  | -0.29 | -0.83 | 0.25 | 0.292 |
|  | Latin America & Caribbean | 0.55 | -0.35 | 1.46 | 0.231 |  | 0.21 | -0.21 | 0.64 | 0.321 |  | **0.92** | **0.47** | **1.37** | **<0.001** |
| Continued breastfeeding  at 2 years | West & Central Africa | -0.27 | -0.63 | 0.09 | 0.136 |  | -0.18 | -0.46 | 0.11 | 0.222 |  | **-0.28** | **-0.48** | **-0.08** | **0.007** |
|  | Eastern & Southern Africa | -0.17 | -0.87 | 0.53 | 0.633 |  | -0.38 | -0.84 | 0.07 | 0.100 |  | -0.16 | -0.41 | 0.10 | 0.226 |
|  | Middle East & North Africa | **-1.04** | **-1.17** | **-0.91** | **<0.001** |  | **-1.05** | **-1.41** | **-0.68** | **<0.001** |  | **-0.52** | **-0.65** | **-0.39** | **<0.001** |
|  | Eastern Europe & Central Asia | -0.29 | -0.73 | 0.14 | 0.184 |  | **0.94** | **0.46** | **1.43** | **<0.001** |  | **0.79** | **0.09** | **1.50** | **0.027** |
|  | South Asia | **-0.29** | **-0.54** | **-0.03** | **0.027** |  | **-0.37** | **-0.68** | **-0.07** | **0.017** |  | **0.26** | **0.08** | **0.44** | **0.004** |
|  | East Asia & the Pacific | -0.21 | -1.39 | 0.96 | 0.720 |  | -0.30 | -0.89 | 0.29 | 0.317 |  | 0.34 | -0.04 | 0.73 | 0.081 |
|  | Latin America & Caribbean | 0.12 | -0.74 | 0.99 | 0.781 |  | 0.36 | -0.08 | 0.79 | 0.109 |  | **0.36** | **0.12** | **0.60** | **0.003** |
| Formula consumption  under 6 months | West & Central Africa | **-0.15** | **-0.23** | **-0.06** | **0.001** |  | **-0.27** | **-0.43** | **-0.11** | **0.001** |  | **-0.45** | **-0.65** | **-0.25** | **<0.001** |
|  | Eastern & Southern Africa | -0.02 | -0.17 | 0.14 | 0.829 |  | **-0.24** | **-0.44** | **-0.04** | **0.020** |  | -0.28 | -0.58 | 0.02 | 0.071 |
|  | Middle East & North Africa | **0.65** | **0.26** | **1.04** | **0.001** |  | **0.63** | **0.14** | **1.12** | **0.011** |  | 0.45 | -0.23 | 1.13 | 0.197 |
|  | Eastern Europe & Central Asia | 0.26 | -1.07 | 1.59 | 0.705 |  | 0.40 | -0.16 | 0.96 | 0.162 |  | -0.10 | -0.29 | 0.10 | 0.331 |
|  | South Asia | 0.02 | -0.05 | 0.08 | 0.590 |  | -0.07 | -0.25 | 0.12 | 0.477 |  | **-0.20** | **-0.26** | **-0.15** | **<0.001** |
|  | East Asia & the Pacific | 0.27 | -0.08 | 0.61 | 0.128 |  | 0.66 | -0.14 | 1.47 | 0.106 |  | 0.53 | -0.41 | 1.46 | 0.269 |
|  | Latin America & Caribbean | 0.08 | -0.10 | 0.26 | 0.375 |  | 0.02 | -0.27 | 0.30 | 0.904 |  | -0.12 | -0.89 | 0.66 | 0.769 |
| Formula consumption  between 6-23 months | West & Central Africa | **-0.18** | **-0.34** | **-0.02** | **0.029** |  | **-0.32** | **-0.45** | **-0.18** | **<0.001** |  | **-0.40** | **-0.71** | **-0.09** | **0.011** |
|  | Eastern & Southern Africa | -0.04 | -0.24 | 0.16 | 0.694 |  | **-0.24** | **-0.43** | **-0.04** | **0.016** |  | **-0.56** | **-1.05** | **-0.08** | **0.024** |
|  | Middle East & North Africa | 0.22 | -0.46 | 0.90 | 0.522 |  | 0.14 | -0.62 | 0.91 | 0.717 |  | -0.17 | -1.39 | 1.06 | 0.788 |
|  | Eastern Europe & Central Asia | -0.02 | -0.72 | 0.68 | 0.958 |  | **0.63** | **0.08** | **1.17** | **0.024** |  | 0.55 | -0.22 | 1.31 | 0.161 |
|  | South Asia | -0.20 | -0.53 | 0.13 | 0.241 |  | **-0.35** | **-0.64** | **-0.07** | **0.015** |  | **-0.69** | **-1.05** | **-0.33** | **<0.001** |
|  | East Asia & the Pacific | 0.05 | -0.31 | 0.41 | 0.796 |  | 0.51 | -0.15 | 1.18 | 0.130 |  | 0.95 | -0.08 | 1.99 | 0.072 |
|  | Latin America & Caribbean | 0.30 | -0.12 | 0.73 | 0.162 |  | **0.39** | **0.11** | **0.66** | **0.005** |  | **0.95** | **0.65** | **1.25** | **<0.001** |

*Betas represent the annual changes in the prevalence of the feeding indicators in percentage points.

**Supplementary table 6.** Annual changes in the prevalence of breast milk and formula consumption indicators by mother’s formal education level according to the World Bank income groups. Source: Demographic Health Survey and Multiple Indicator Cluster Survey.

| Indicator | Income group | Formal education level | | | | | | | | | | | | | |
| --- | --- | --- | --- | --- | --- | --- | --- | --- | --- | --- | --- | --- | --- | --- | --- |
|  |  | None | | | |  | Primary | | | |  | Secondary or higher | | | |
|  |  | β^*^ | 95% CI | | P |  | β^*^ | 95% CI | | P |  | β^*^ | 95% CI | | P |
| Early initiation of breastfeeding | Upper-middle income | **0.96** | **0.23** | **1.70** | **0.010** |  | **0.69** | **0.20** | **1.17** | **0.005** |  | 0.18 | -0.29 | 0.65 | 0.454 |
|  | Lower-middle income | 0.34 | -0.09 | 0.78 | 0.125 |  | 0.42 | -0.02 | 0.86 | 0.062 |  | 0.39 | -0.08 | 0.85 | 0.101 |
|  | Low income | **1.40** | **0.64** | **2.16** | **<0.001** |  | **1.29** | **0.63** | **1.94** | **<0.001** |  | **1.03** | **0.50** | **1.57** | **<0.001** |
| Exclusive breastfeeding  under 6 months | Upper-middle income | **1.14** | **0.12** | **2.16** | **0.029** |  | **0.91** | **0.16** | **1.65** | **0.017** |  | **1.35** | **0.41** | **2.29** | **0.005** |
|  | Lower-middle income | 0.75 | -0.08 | 1.57 | 0.077 |  | 0.65 | -0.20 | 1.50 | 0.136 |  | **1.18** | **0.48** | **1.89** | **0.001** |
|  | Low income | **0.85** | **0.38** | **1.33** | **<0.001** |  | **1.05** | **0.73** | **1.36** | **<0.001** |  | **1.12** | **0.83** | **1.42** | **<0.001** |
| Continued breastfeeding  at 1 years | Upper-middle income | 0.03 | -0.49 | 0.54 | 0.914 |  | **0.93** | **0.19** | **1.68** | **0.014** |  | **1.30** | **0.41** | **2.19** | **0.004** |
|  | Lower-middle income | -0.03 | -0.49 | 0.43 | 0.899 |  | 0.08 | -0.37 | 0.53 | 0.729 |  | **0.57** | **0.15** | **0.99** | **0.007** |
|  | Low income | **-0.20** | **-0.31** | **-0.09** | **<0.001** |  | -0.06 | -0.16 | 0.04 | 0.221 |  | 0.12 | -0.04 | 0.29 | 0.145 |
| Continued breastfeeding  at 2 years | Upper-middle income | -0.29 | -0.88 | 0.30 | 0.341 |  | 0.27 | -0.66 | 1.20 | 0.571 |  | **0.62** | **0.33** | **0.92** | **<0.001** |
|  | Lower-middle income | 0.37 | -0.35 | 1.09 | 0.316 |  | 0.13 | -0.55 | 0.81 | 0.711 |  | 0.37 | -0.13 | 0.88 | 0.146 |
|  | Low income | **-0.33** | **-0.52** | **-0.15** | **<0.001** |  | -0.20 | -0.40 | 0.01 | 0.062 |  | 0.00 | -0.25 | 0.25 | 0.990 |
| Formula consumption  under 6 months | Upper-middle income | 0.46 | -0.73 | 1.65 | 0.448 |  | **0.51** | **0.27** | **0.76** | **<0.001** |  | -0.09 | -1.00 | 0.82 | 0.847 |
|  | Lower-middle income | -0.27 | -0.58 | 0.04 | 0.091 |  | -0.12 | -0.44 | 0.21 | 0.478 |  | -0.45 | -0.92 | 0.02 | 0.058 |
|  | Low income | **-0.13** | **-0.25** | **0.00** | **0.044** |  | **-0.24** | **-0.38** | **-0.09** | **0.001** |  | **-0.26** | **-0.45** | **-0.06** | **0.009** |
| Formula consumption  between 6-23 months | Upper-middle income | 0.23 | -0.32 | 0.77 | 0.410 |  | **0.72** | **0.56** | **0.87** | **<0.001** |  | **0.85** | **0.38** | **1.32** | **<0.001** |
|  | Lower-middle income | **-0.19** | **-0.35** | **-0.03** | **0.022** |  | -0.18 | -0.46 | 0.09 | 0.189 |  | -0.20 | -0.64 | 0.23 | 0.360 |
|  | Low income | -0.24 | -0.47 | 0.00 | 0.051 |  | **-0.34** | **-0.58** | **-0.10** | **0.006** |  | **-0.61** | **-1.01** | **-0.21** | **0.003** |

*Betas represent the annual changes in the prevalence of the feeding indicators in percentage points.

**Supplementary table 7.** Average weighted prevalence of breast milk and formula consumption indicators by mother’s formal education level according to income groups^*^

| Indicator | Income group | Formal education level | | |
| --- | --- | --- | --- | --- |
|  |  | None (%) | Primary (%) | Secondary or higher (%) |
| Early initiation of breastfeeding | Upper-middle income | 61.6 | 57.5 | 54.9 |
|  | Lower-middle income | 41.7 | 42.3 | 43.2 |
|  | Low income | 57.5 | 57.4 | 56.8 |
| Exclusive breastfeeding under 6 months | Upper-middle income | 31.0 | 33.7 | 33.4 |
|  | Lower-middle income | 47.2 | 46.7 | 48.0 |
|  | Low income | 50.7 | 52.5 | 50.1 |
| Continued breastfeeding at 1 year | Upper-middle income | 69.5 | 59.1 | 50.4 |
|  | Lower-middle income | 85.6 | 84.3 | 76.1 |
|  | Low income | 89.9 | 90.9 | 87.6 |
| Continued breastfeeding at 2 years | Upper-middle income | 44.8 | 27.5 | 24.0 |
|  | Lower-middle income | 64.6 | 56.2 | 47.9 |
|  | Low income | 65.8 | 57.3 | 45.8 |
| Formula consumption under 6 months | Upper-middle income | 26.3 | 33.4 | 36.0 |
|  | Lower-middle income | 4.0 | 8.1 | 14.5 |
|  | Low income | 1.9 | 2.4 | 8.0 |
| Formula consumption between 6-23 months | Upper-middle income | 24.0 | 26.7 | 38.2 |
|  | Lower-middle income | 5.3 | 8.3 | 17.2 |
|  | Low income | 2.4 | 3.2 | 9.2 |

*Figures are based on the most recent survey included in the trend analysis weighted by the population of children in each country.

**Supplementary table 8.** Average weighted prevalence of breast milk and formula consumption indicators by mother’s formal education level according to regions of the world^*^

| Indicator | Regions of the world | Formal education level | | |
| --- | --- | --- | --- | --- |
|  |  | None (%) | Primary (%) | Secondary or higher (%) |
| Early initiation of breastfeeding | West & Central Africa | 42.8 | 45.9 | 47.9 |
|  | Eastern & Southern Africa | 69.0 | 66.9 | 65.1 |
|  | Middle East & North Africa | 36.0 | 32.1 | 29.2 |
|  | Eastern Europe & Central Asia | 70.1 | 62.4 | 67.0 |
|  | South Asia | 36.0 | 38.4 | 39.6 |
|  | East Asia & the Pacific | 56.7 | 50.6 | 48.2 |
|  | Latin America & Caribbean | 67.1 | 64.0 | 52.4 |
| Exclusive breastfeeding under 6 months | West & Central Africa | 31.4 | 35.0 | 40.5 |
|  | Eastern & Southern Africa | 52.8 | 58.0 | 57.3 |
|  | Middle East & North Africa | 37.5 | 31.7 | 33.1 |
|  | Eastern Europe & Central Asia | 45.9 | 28.3 | 32.1 |
|  | South Asia | 54.4 | 52.9 | 52.8 |
|  | East Asia & the Pacific | 40.8 | 43.6 | 40.9 |
|  | Latin America & Caribbean | 58.0 | 50.5 | 41.6 |
| Continued breastfeeding at 1 year | West & Central Africa | 90.9 | 87.1 | 78.6 |
|  | Eastern & Southern Africa | 86.6 | 89.1 | 84.6 |
|  | Middle East & North Africa | 69.9 | 67.1 | 64.1 |
|  | Eastern Europe & Central Asia | 75.5 | 71.2 | 60.0 |
|  | South Asia | 86.9 | 85.3 | 81.2 |
|  | East Asia & the Pacific | 77.0 | 78.3 | 63.4 |
|  | Latin America & Caribbean | 87.2 | 77.2 | 64.4 |
| Continued breastfeeding at 2 years | West & Central Africa | 57.2 | 37.5 | 22.8 |
|  | Eastern & Southern Africa | 66.6 | 52.8 | 45.3 |
|  | Middle East & North Africa | 32.7 | 27.1 | 18.3 |
|  | Eastern Europe & Central Asia | 33.7 | 34.1 | 28.5 |
|  | South Asia | 75.0 | 71.1 | 65.9 |
|  | East Asia & the Pacific | 53.4 | 47.8 | 38.0 |
|  | Latin America & Caribbean | 48.8 | 42.2 | 33.6 |
| Consumption of formula under 6 months | West & Central Africa | 2.2 | 4.5 | 12.7 |
|  | Eastern & Southern Africa | 4.6 | 3.2 | 7.0 |
|  | Middle East & North Africa | 22.1 | 23.3 | 29.3 |
|  | Eastern Europe & Central Asia | 19.3 | 26.5 | 24.1 |
|  | South Asia | 3.9 | 5.4 | 7.8 |
|  | East Asia & the Pacific | 5.0 | 22.3 | 36.2 |
|  | Latin America & Caribbean | 12.3 | 17.4 | 34.3 |
| Consumption of formula between 6-23 months | West & Central Africa | 2.7 | 4.0 | 12.8 |
|  | Eastern & Southern Africa | 4.5 | 2.7 | 7.8 |
|  | Middle East & North Africa | 14.9 | 16.9 | 19.9 |
|  | Eastern Europe & Central Asia | 9.7 | 17.1 | 25.1 |
|  | South Asia | 5.8 | 7.1 | 12.6 |
|  | East Asia & the Pacific | 9.6 | 22.0 | 42.9 |
|  | Latin America & Caribbean | 11.0 | 14.5 | 29.8 |

*Figures are based on the most recent survey included in the trend analysis weighted by the population of children in each country.

**Supplementary table 9.** Annual changes in the prevalence of breast milk and formula consumption indicators by mother’s formal education level for selected countries. Source: Demographic Health Survey and Multiple Indicator Cluster Survey.

| Indicator | Country | Year | Formal education level | | | | | | | | | | | |
| --- | --- | --- | --- | --- | --- | --- | --- | --- | --- | --- | --- | --- | --- | --- |
|  |  |  | None | | | | Primary | | | | Secondary | | | |
|  |  |  | β^*^ | 95% CI | | P | β^*^ | 95% CI | | P | β^*^ | 95% CI | | P |
| Early initiation of breastfeeding | Bangladesh | 2019 | **2.30** | **1.55** | **3.04** | **<0.001** | **2.04** | **1.43** | **2.64** | **<0.001** | **1.39** | **0.69** | **2.10** | **0.002** |
|  | Egypt | 2014 | -0.46 | -3.87 | 2.94 | 0.693 | -0.38 | -3.35 | 2.59 | 0.711 | -0.59 | -2.86 | 1.69 | 0.472 |
|  | Kenya | 2014 | 0.83 | -0.57 | 2.24 | 0.155 | 0.34 | -0.31 | 1.00 | 0.194 | 0.16 | -0.13 | 0.44 | 0.179 |
|  | Nigeria | 2018 | -0.09 | -0.94 | 0.77 | 0.812 | 0.18 | -0.53 | 0.88 | 0.558 | 0.33 | -0.77 | 1.43 | 0.488 |
|  | Peru | 2018 | **1.75** | **1.15** | **2.35** | **<0.001** | **1.18** | **0.83** | **1.54** | **<0.001** | -0.08 | -0.42 | 0.27 | 0.635 |
|  | Turkey | 2013 | **1.85** | **0.20** | **3.49** | **0.040** | 1.89 | -1.12 | 4.90 | 0.114 | 1.84 | -2.09 | 5.78 | 0.181 |
|  | Vietnam | 2013 | 1.41 | -2.47 | 5.29 | 0.330 | 0.34 | -2.47 | 3.15 | 0.724 | -0.70 | -4.28 | 2.88 | 0.579 |
| Exclusive breastfeeding under 6 months | Bangladesh | 2019 | 0.34 | -0.11 | 0.80 | 0.122 | **0.92** | **0.50** | **1.35** | **0.001** | **1.01** | **0.61** | **1.40** | **<0.001** |
|  | Egypt | 2014 | **-1.05** | **-1.71** | **-0.38** | **0.015** | **-1.23** | **-2.30** | **-0.17** | **0.035** | -0.39 | -1.37 | 0.59 | 0.299 |
|  | Kenya | 2014 | 1.92 | -0.54 | 4.38 | 0.089 | **2.32** | **0.47** | **4.17** | **0.028** | 2.55 | -0.35 | 5.46 | 0.068 |
|  | Nigeria | 2018 | 0.56 | -0.08 | 1.20 | 0.077 | 0.37 | -0.27 | 1.02 | 0.207 | 0.56 | -0.36 | 1.48 | 0.187 |
|  | Peru | 2018 | **0.90** | **0.06** | **1.73** | **0.037** | **0.73** | **0.45** | **1.00** | **<0.001** | 0.69 | -0.01 | 1.39 | 0.052 |
|  | Turkey | 2013 | **0.96** | **0.17** | **1.76** | **0.035** | 0.93 | 0.00 | 1.85 | 0.050 | 1.38 | -0.07 | 2.83 | 0.055 |
|  | Vietnam | 2013 | 2.31 | -0.08 | 4.69 | 0.054 | 0.35 | -0.84 | 1.54 | 0.415 | 0.35 | -0.33 | 1.04 | 0.200 |
| Continued breastfeeding at 1 year | Bangladesh | 2019 | -0.25 | -0.65 | 0.15 | 0.180 | -0.07 | -0.26 | 0.12 | 0.437 | **0.17** | **0.03** | **0.31** | **0.021** |
|  | Egypt | 2014 | -0.19 | -1.19 | 0.80 | 0.579 | **-0.62** | **-1.13** | **-0.11** | **0.031** | 0.48 | -0.34 | 1.31 | 0.157 |
|  | Kenya | 2014 | -0.10 | -0.78 | 0.59 | 0.681 | -0.02 | -0.40 | 0.36 | 0.903 | -0.40 | -1.35 | 0.54 | 0.269 |
|  | Nigeria | 2018 | 0.00 | -0.48 | 0.47 | 0.987 | 0.06 | -0.70 | 0.83 | 0.844 | 0.04 | -0.51 | 0.60 | 0.863 |
|  | Peru | 2018 | 0.04 | -0.22 | 0.29 | 0.759 | 0.07 | -0.20 | 0.34 | 0.579 | **0.56** | **0.23** | **0.89** | **0.002** |
|  | Turkey | 2013 | 0.84 | -0.43 | 2.10 | 0.104 | **1.25** | **0.46** | **2.03** | **0.021** | **2.01** | **1.20** | **2.83** | **0.009** |
|  | Vietnam | 2013 | 0.22 | -3.58 | 4.01 | 0.867 | **-0.34** | **-0.56** | **-0.12** | **0.016** | -1.16 | -2.55 | 0.24 | 0.078 |
| Continued breastfeeding at 2 years | Bangladesh | 2019 | **-0.40** | **-0.78** | **-0.01** | **0.044** | -0.24 | -0.51 | 0.04 | 0.085 | 0.17 | -0.21 | 0.55 | 0.341 |
|  | Egypt | 2014 | **-1.01** | **-1.64** | **-0.37** | **0.015** | -1.19 | -2.68 | 0.30 | 0.085 | -0.56 | -1.14 | 0.02 | 0.053 |
|  | Kenya | 2014 | -0.52 | -1.37 | 0.32 | 0.142 | -0.34 | -1.04 | 0.37 | 0.228 | -0.10 | -1.42 | 1.23 | 0.834 |
|  | Nigeria | 2018 | 0.02 | -0.51 | 0.55 | 0.921 | 0.08 | -0.75 | 0.91 | 0.820 | -0.35 | -0.97 | 0.28 | 0.224 |
|  | Peru | 2018 | -0.53 | -1.59 | 0.54 | 0.309 | 0.06 | -0.45 | 0.57 | 0.803 | 0.26 | -0.01 | 0.53 | 0.058 |
|  | Turkey | 2013 | **-0.46** | **-0.77** | **-0.15** | **0.024** | **1.18** | **0.52** | **1.85** | **0.017** | **1.31** | **0.24** | **2.38** | **0.034** |
|  | Vietnam | 2013 | 0.30 | -1.63 | 2.23 | 0.657 | **-1.23** | **-2.04** | **-0.41** | **0.017** | -0.14 | -0.76 | 0.47 | 0.510 |
| Formula consumption under 6 months | Bangladesh | 2019 | 0.01 | -0.46 | 0.48 | 0.972 | -0.15 | -0.56 | 0.25 | 0.406 | -0.19 | -0.47 | 0.09 | 0.152 |
|  | Egypt | 2014 | 0.45 | -0.41 | 1.31 | 0.194 | 0.38 | -0.53 | 1.30 | 0.275 | 0.09 | -1.11 | 1.29 | 0.823 |
|  | Kenya | 2014 | -0.04 | -0.33 | 0.26 | 0.707 | **-0.15** | **-0.26** | **-0.03** | **0.027** | -0.05 | -1.07 | 0.96 | 0.882 |
|  | Nigeria | 2018 | -0.18 | -0.48 | 0.11 | 0.176 | -0.34 | -0.96 | 0.27 | 0.222 | -0.52 | -1.37 | 0.33 | 0.187 |
|  | Peru | 2018 | 0.08 | -0.21 | 0.37 | 0.578 | **0.25** | **0.12** | **0.38** | **0.001** | **0.54** | **0.28** | **0.79** | **<0.001** |
|  | Turkey | 2013 | 1.07 | -0.76 | 2.90 | 0.128 | **0.60** | **0.17** | **1.03** | **0.027** | -0.17 | -1.87 | 1.52 | 0.703 |
|  | Vietnam | 2013 | 0.60 | -1.56 | 2.76 | 0.442 | **2.09** | **0.97** | **3.20** | **0.009** | **2.89** | **0.37** | **5.40** | **0.036** |
| Formula consumption between 6-23 months | Bangladesh | 2019 | -0.47 | -1.07 | 0.12 | 0.103 | **-0.60** | **-1.16** | **-0.04** | **0.038** | **-0.92** | **-1.57** | **-0.27** | **0.011** |
|  | Egypt | 2014 | -0.09 | -0.23 | 0.06 | 0.145 | -0.24 | -0.86 | 0.38 | 0.303 | **-0.79** | **-1.49** | **-0.09** | **0.037** |
|  | Kenya | 2014 | 0.00 | -0.23 | 0.23 | 0.998 | -0.04 | -0.12 | 0.03 | 0.140 | **-0.14** | **-0.27** | **-0.01** | **0.042** |
|  | Nigeria | 2018 | -0.03 | -0.28 | 0.22 | 0.767 | -0.28 | -0.78 | 0.22 | 0.215 | -0.23 | -1.06 | 0.60 | 0.517 |
|  | Peru | 2018 | 0.11 | -0.04 | 0.26 | 0.132 | **0.35** | **0.15** | **0.55** | **0.002** | **1.10** | **0.74** | **1.46** | **<0.001** |
|  | Turkey | 2013 | 0.31 | -0.03 | 0.64 | 0.059 | **0.92** | **0.42** | **1.42** | **0.016** | 1.16 | -0.12 | 2.44 | 0.060 |
|  | Vietnam | 2013 | 0.49 | -0.67 | 1.65 | 0.271 | **1.34** | **0.49** | **2.19** | **0.015** | **2.37** | **0.93** | **3.80** | **0.013** |

*Betas represent the annual changes in the prevalence of the feeding indicators in percentage points.
