# Supplementary material for: Maternal education and equity in breastfeeding: trends and patterns in 81 low- and middle-income countries between 2000 and 2019
Source: Int J Equity Health. 2021 Jan 7;20:20. doi: 10.1186/s12939-020-01357-3 (PMC7792102; doi:10.1186/s12939-020-01357-3)
Supplement: Supplementary file 2 — Additional file 2: Figure S1. Average absolute annual changes in breast milk and formula consumption indicators by income groups. Figure S2. Trends over the 20-years period in breast milk and formula consumption indicators by income groups. Figure S3. Trends over the 20-years period in breast milk and formula consumption indicators by regions of the world. Figure S4. Average absolute annual changes in breast milk and formula consumption indicators for selected countries. Figure S5. Per capita sales of standard (0–5 months), follow-on (6–12 months), and growing-up (13–36 months) formula from Euromonitor International for selected countries. Figure S6. Changes over the 18-year period in (A) literacy rate for women 15 years or above and (B) school enrollment in primary education for girls. [file 12939_2020_1357_MOESM2_ESM.docx]

**Supplementary figure 1.** Average absolute annual changes in breast milk and formula consumption indicators by income groups.


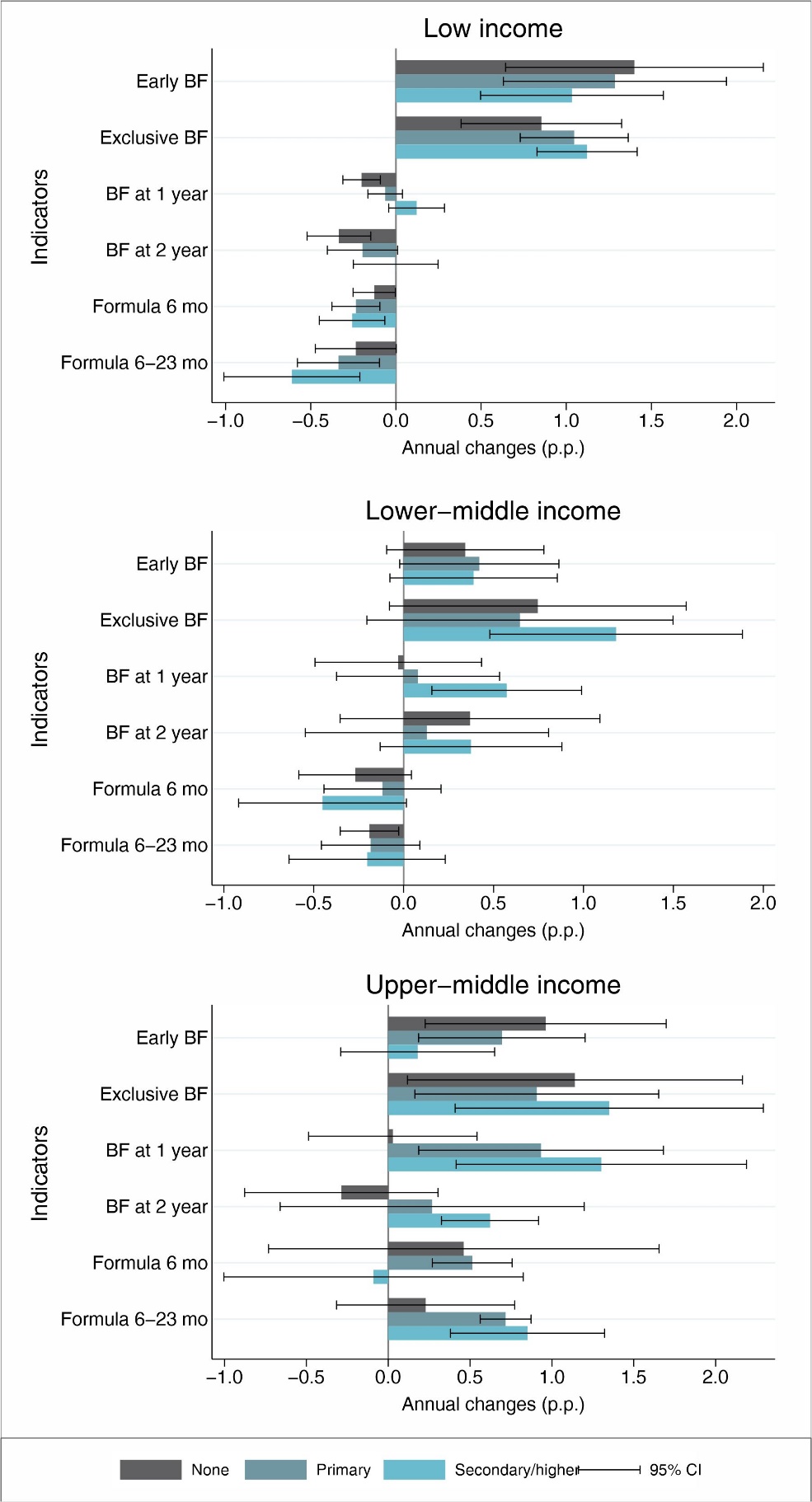


**Early BF** – Early initiation of breastfeeding; **Exclusive BF** – Exclusive breastfeeding under six months; **BF at 1 year** - Continued breastfeeding at 1 year; **BF at 2 years** – Continued breastfeeding at 2 years; **Formula 6 mo** – Consumption of formula under six months; **Formula 6-23 mo** – Consumption of formula between 6-23 months.

**Supplementary figure 2.** Trends over the 20-years period in breast milk and formula consumption indicators by income groups.


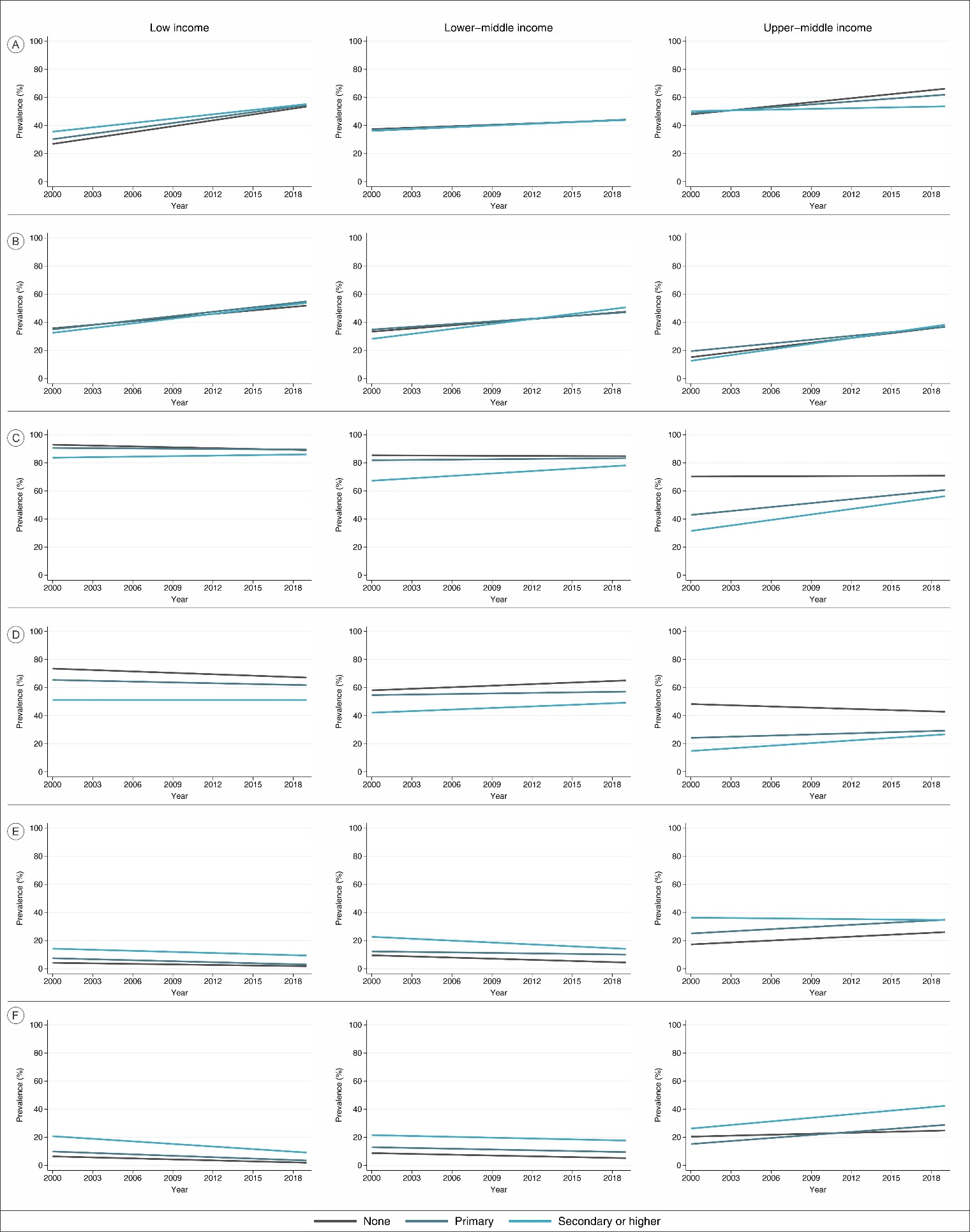


A – Early initiation of breastfeeding; B – Exclusive breastfeeding under six months; C – Continued breastfeeding at 1 year; D – Continued breastfeeding at 2 years; E – Consumption of formula under six months; F – Consumption of formula between 6-23 months

**Supplementary figure 3.** Trends over the 20-years period in breast milk and formula consumption indicators by regions of the world.


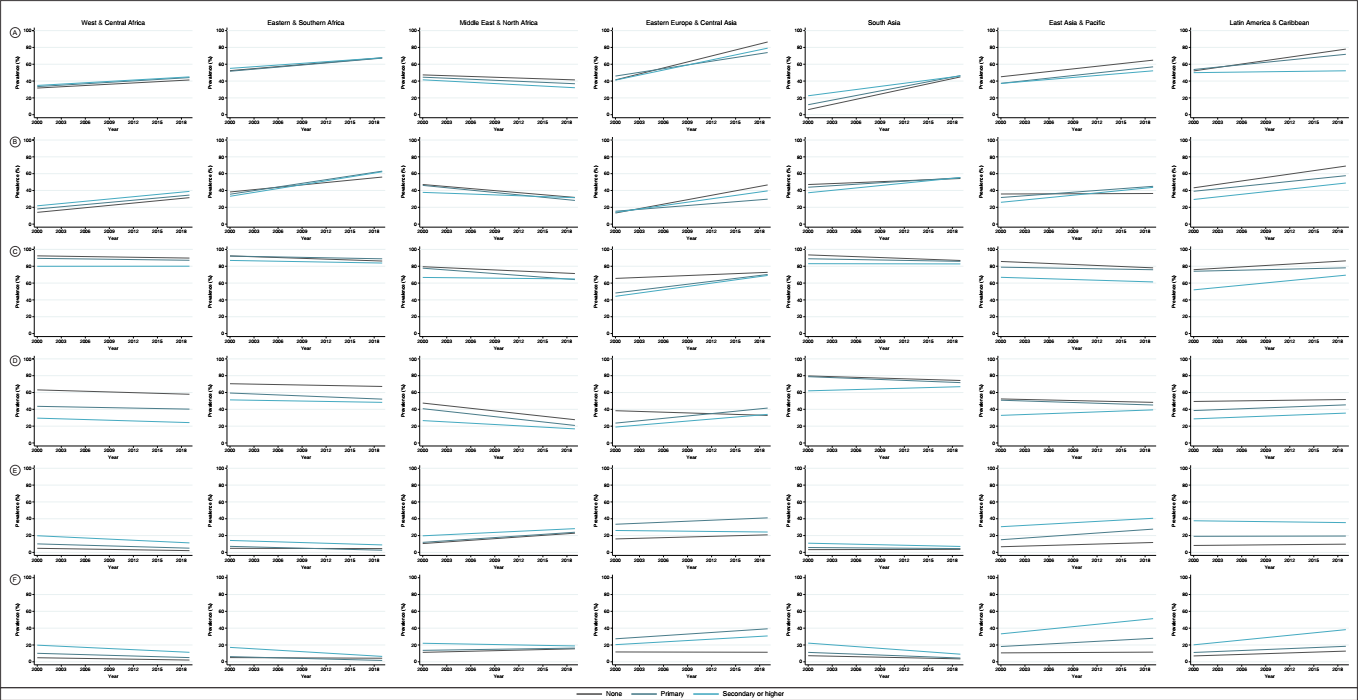


A – Early initiation of breastfeeding; B – Exclusive breastfeeding under six months; C – Continued breastfeeding at 1 year; D – Continued breastfeeding at 2 years; E – Consumption of formula under six months; F – Consumption of formula between 6-23 months

**Supplementary figure 4.** Average absolute annual changes in breast milk and formula consumption indicators for selected countries.


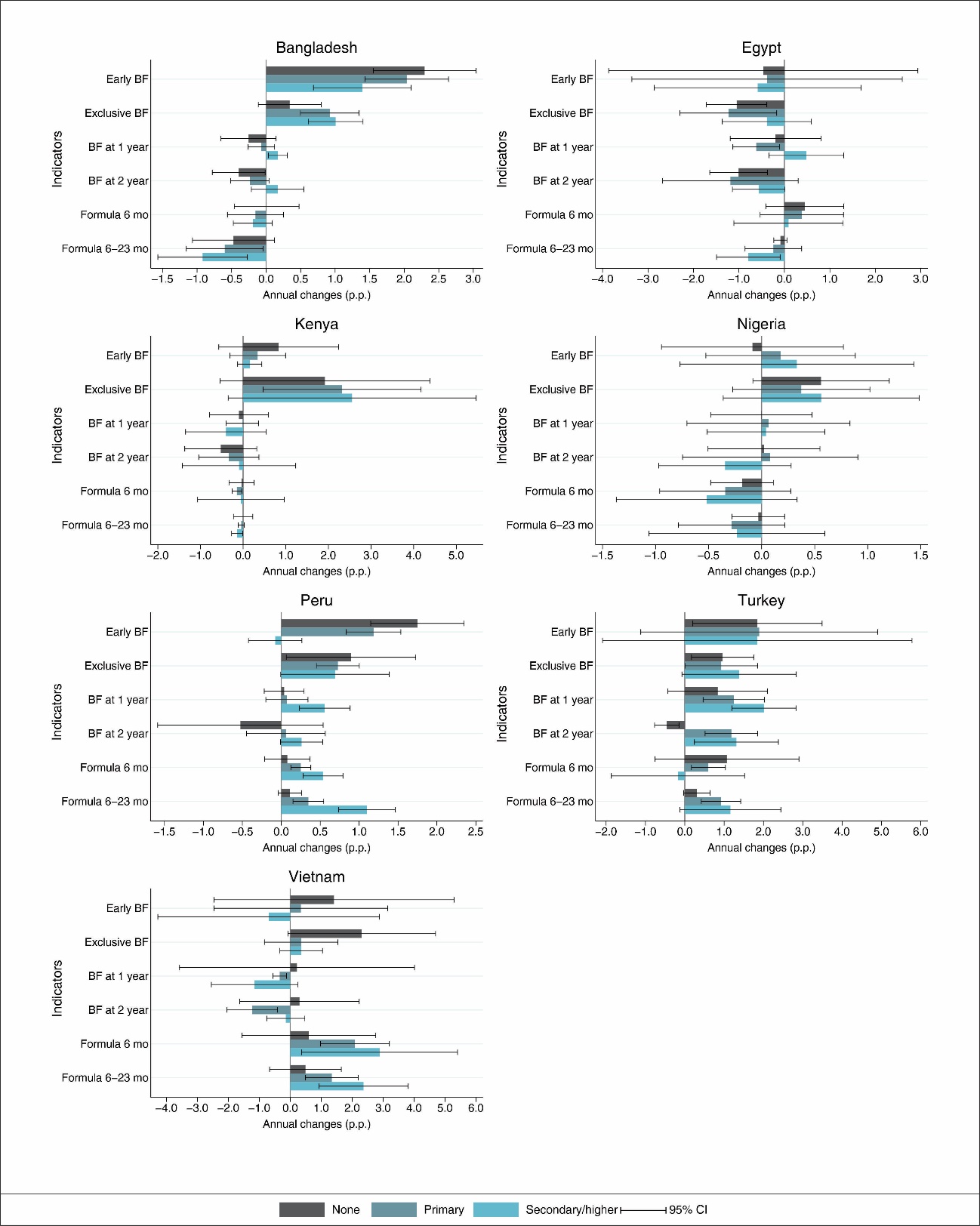


**Early BF** – Early initiation of breastfeeding; **Exclusive BF** – Exclusive breastfeeding under six months; **BF at 1 year** - Continued breastfeeding at 1 year; **BF at 2 years** – Continued breastfeeding at 2 years; **Formula 6 mo** – Consumption of formula under six months; **Formula 6-23 mo** – Consumption of formula between 6-23 months.

**Supplementary figure 5.** Per capita sales of standard (0-5 months), follow-on (6-12 months), and growing-up (13-36 months) formula from Euromonitor International for selected countries.


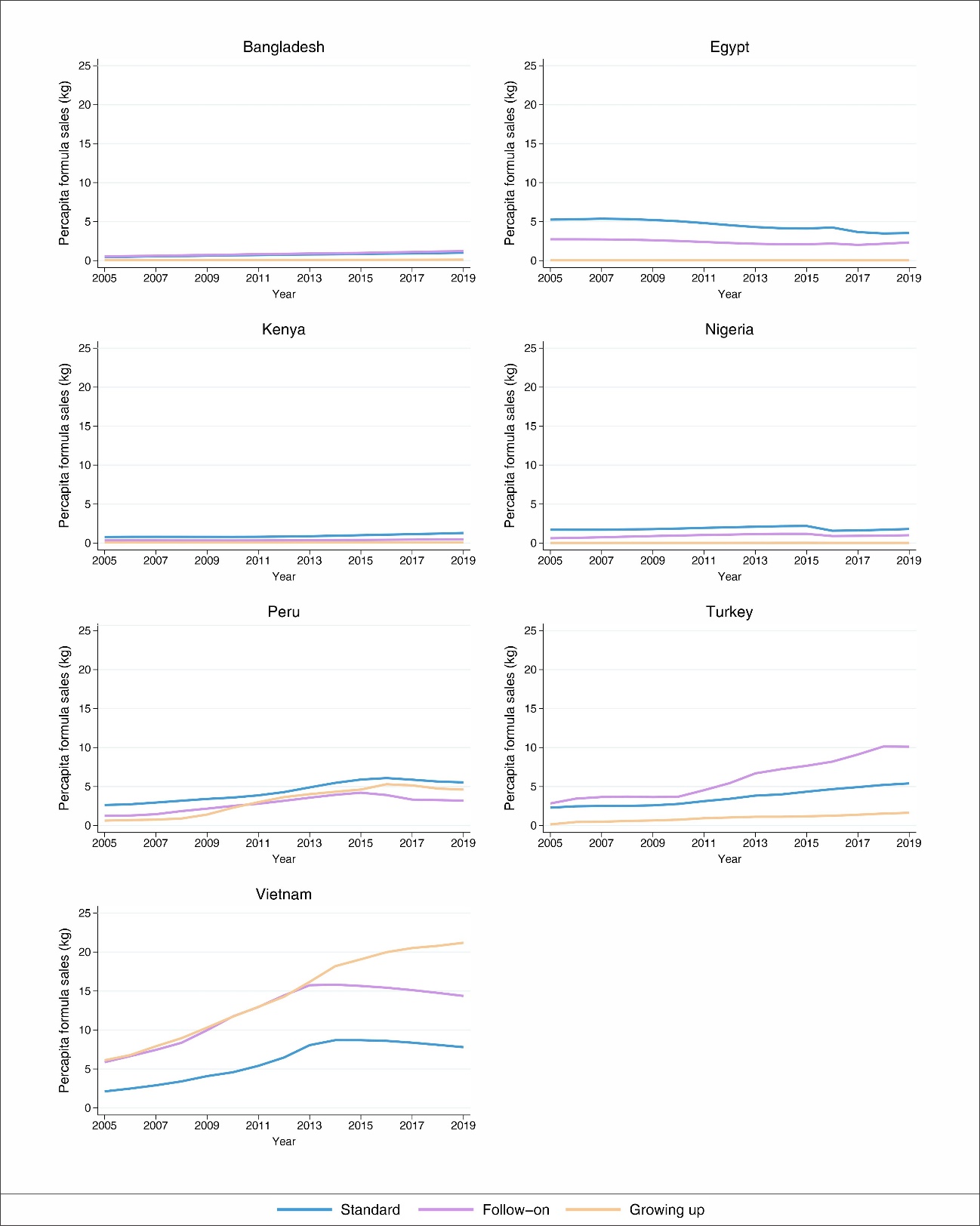


**Supplementary figure 6.** Changes over the 18-year period in (A) literacy rate for women 15 years or above and (B) school enrollment in primary education for girls.


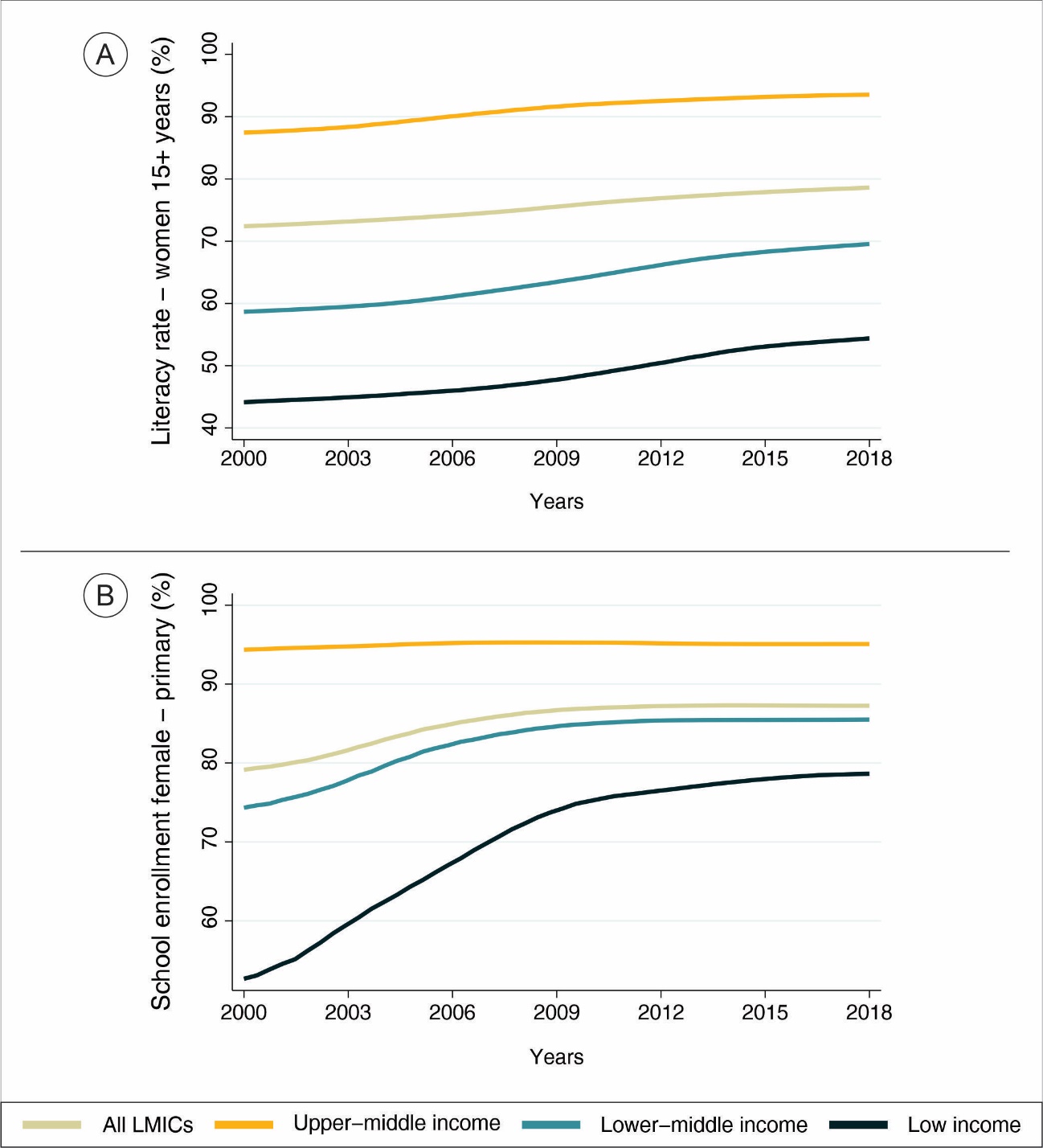


LMICs: low- and middle-income countries
